# Clinical Benefits and Risks of Antiamyloid Antibodies in Sporadic Alzheimer Disease: Systematic Review and Network Meta-Analysis With a Web Application

**DOI:** 10.2196/68454

**Published:** 2025-04-07

**Authors:** Danko Jeremic, Juan D Navarro-Lopez, Lydia Jimenez-Diaz

**Affiliations:** 1 Neurophysiology & Behavior Lab Institute of Biomedicine (IB-UCLM) and Instituto de Investigación Sanitaria de Castilla-La Mancha (IDISCAM), Faculty of Medicine of Ciudad Real University of Castilla-La Mancha Ciudad Real Spain

**Keywords:** Alzheimer disease, antibodies, donanemab, aducanumab, lecanemab

## Abstract

**Background:**

Despite the increasing approval of antiamyloid antibodies for Alzheimer disease (AD), their clinical relevance and risk-benefit profile remain uncertain. The heterogeneity of AD and the limited availability of long-term clinical data make it difficult to establish a clear rationale for selecting one treatment over another.

**Objective:**

The aim of this work was to assess and compare the efficacy and safety of antiamyloid antibodies through an interactive online meta-analytic approach by performing conventional pair-wise meta-analyses and frequentist and Bayesian network meta-analyses of phase II and III clinical trial results. To achieve this, we developed AlzMeta.app 2.0, a freely accessible web application that enables researchers and clinicians to evaluate the relative and absolute risks and benefits of these therapies in real time, incorporating different prior choices and assumptions of baseline risks of disease progression and adverse events.

**Methods:**

We adhered to PRISMA-NMA (Preferred Reporting Items for Systematic Reviews and Meta-Analyses extension for reporting of systematic reviews with network meta-analysis) and GRADE (Grading of Recommendations, Assessment, Development, and Evaluation) guidelines for reporting and rating the certainty of evidence. Clinical trial reports (until September 30, 2024) were retrieved from PubMed, Google Scholar, and clinical trial databases (including ClinicalTrials.gov). Studies with <20 sporadic AD patients and a modified Jadad score <3 were excluded. Risk of bias was assessed with the RoB-2 tool. Relative risks and benefits have been expressed as risk ratios and standardized mean differences, with confidence, credible, and prediction intervals calculated for all outcomes. For significant results, the intervention effects were ranked in frequentist and Bayesian frameworks, and their clinical relevance was determined by the absolute risk per 1000 people and number needed to treat (NNT) for a wide range of control responses.

**Results:**

Among 7 treatments tested in 21,236 patients (26 studies with low risk of bias or with some concerns), donanemab was the best-ranked treatment on cognitive and functional measures, and it was almost 2 times more effective than aducanumab and lecanemab and significantly more beneficial than other treatments on the global (cognitive and functional) Clinical Dementia Rating Scale-Sum of Boxes (NNT=10, 95% CI 8-16). Special caution is required regarding cerebral edema and microbleeding due to the clinically relevant risks of edema for donanemab (NNT=8, 95% CI 5-16), aducanumab (NNT=10, 95% CI 6-17), and lecanemab (NNT=14, 95% CI 7-31), which may outweigh the benefits.

**Conclusions:**

Our results showed that donanemab is more effective and has a safety profile similar to aducanumab and lecanemab, highlighting the need for treatment options with improved safety. Potential bias may have been introduced in the included trials due to unblinding caused by frequent cerebral edema and microbleeds, as well as the impact of the COVID-19 pandemic.

## Introduction

Until lately, the only available therapies for Alzheimer disease (AD) were related to symptomatic presentation, and this situation remains in most countries. Regulatory approvals of aducanumab (2021), lecanemab (2023), and donanemab (2024) in the United States have marked a new era in AD treatment, as these antibodies demonstrated the potential to modify disease progression by targeting amyloid β (Aβ), which involves toxic peptides deemed crucial in the AD pathophysiology [1]. While the development of aducanumab was discontinued earlier in 2024 (January 2024) [2], lecanemab and donanemab are currently under review for approval in Europe and worldwide [3], and lecanemab is already available for patients outside of the United States, including those in the United Kingdom, Japan, China, South Korea, Israel, and the United Arab Emirates [4-6].

Despite the clear advantages of anti-Aβ antibodies, substantial doubts remain about their risk-to-benefit profile and clinical relevance, and they still have to demonstrate clinically meaningful effects in real life [7-10]. There is limited understanding of their long-term effects, and the safety profile has yet to be fully explored in broader populations. Although reductions in amyloid load are well-documented and substantial, it remains uncertain whether these changes lead to significant improvements in cognition, disease progression, and daily functioning. The complexity of AD and its varying pathophysiological characteristics make it difficult to predict how these therapies will impact different patient subgroups, including those with different levels of tau pathology, disease stages, or varying risks of cognitive/functional decline and adverse events (AEs) [7,11-13].

We previously performed a web application meta-analysis of these treatments in phase III randomized placebo-controlled clinical trials (RCTs) involving sporadic AD [14]. Our analysis included aducanumab [15], lecanemab [16], bapineuzumab [17,18], and solanezumab [19,20], showing that aducanumab and lecanemab produced the most promising cognitive, functional, and biomarker results. However, these effects were achieved at the great expense of increased AEs, primarily amyloid-related imaging abnormalities (ARIAs) in the form of vasogenic cerebral edema and sulcal effusion (ARIA-E) and microhemorrhage and superficial siderosis (ARIA-H) [14]. Our study had some limitations as we analyzed only large phase III RCTs (>200 patients) with the conventional (pair-wise) meta-analysis method. This did not allow us to compare the effects of the treatments and rank their performance, and we failed to include smaller studies [21,22] and novel antibodies, including donanemab [23,24], gantenerumab [25,26], and crenezumab [27].

Unlike conventional pair-wise meta-analysis, which only compares treatments that have been directly tested against each other in clinical trials, a network meta-analysis (NMA) allows for indirect treatment comparisons. This means that even if 2 interventions have not been directly compared in a trial, a NMA can estimate their relative effectiveness based on their shared comparability to a common reference intervention. Therefore, the aim of this study was to update and expand our previous application [14] in 3 steps. First, we performed a conventional (frequentist) random effects meta-analysis with the DerSimonian and Laird method [28,29] on an extended dataset and additional outcomes in sporadic AD. Then, we conducted a frequentist [30] and Bayesian NMA [31] of phase II and III RCTs in order to compare the effectiveness and safety of anti-Αβ antibodies. Finally, we evaluated the clinical relevance of these interventions by estimating the absolute benefits and risks over an entire range of placebo responses and different prior assumptions of heterogeneity.

## Methods

### Search Strategy, Quality Assessments, and Inclusion Criteria

We followed the PRISMA-NMA (Preferred Reporting Items for Systematic Reviews and Meta-Analyses extension for reporting of systematic reviews with network meta-analysis) guidelines (Multimedia Appendix 1) [32] and GRADE (Grading of Recommendations, Assessment, Development, and Evaluation) guidelines [33] for reporting and rating the certainty (quality) of underlying evidence. Primary study sources were PubMed, Google Scholar, and multiple clinical trial databases, including the US Clinical Trials Registry (ClinicalTrials.gov), the EU Clinical Trials Registry, the Australian New Zealand Clinical Trials Registry (ANZCTR), and the International Clinical Trials Registry Platform (ICTRP) of the World Health Organization. The search terms were as follows: “Alzheimer’s,” “sporadic,” “mild cognitive impairment,” “phase 3,” “phase 2,” “monoclonal antibody,” “passive immunotherapy,” “Aducanumab,” “BIIB037,” “Gantenerumab,” “Lecanemab,” “BAN-2401,” “Solanezumab,” “LY2062430,” “Crenezumab,” “Bapineuzumab,” “AAB-001,” “Donanemab,” and “LY3002813.” No age or language restrictions were applied. We excluded studies that (1) tested <20 sporadic AD patients and (2) were not phase II/III RCTs with a report quality of ≥3 on the modified Jadad scale. The details of the electronic search strategy and modified Jadad scale assessments can be found in Multimedia Appendix 2. In brief, apart from the regular examination of study randomization, blinding, dropouts, and withdrawals [34], we evaluated whether there were clear descriptions of the inclusion and exclusion criteria, statistical analysis, and methods used to assess AEs such as ARIA. The search for eligible studies was performed by each author independently (until September 30, 2024), and any disagreements were resolved through consensus. Excel was then used to automatically eliminate duplicate studies. The study did not include a registered review protocol, and it mainly expanded our previously published protocol [14] that was initially limited in scope, focusing only on larger phase III studies with no less than 200 patients in each arm.

### Primary (Cognitive and Functional) Outcomes

The primary outcomes included cognitive and functional measures from all or most studies, and they involved mean changes from baseline in the (1) AD Assessment Scale-Cognitive Subscale (ADAS-Cog), (2) Mini-Mental State Examination (MMSE), and (3) Clinical Dementia Rating Scale-Sum of Boxes (CDR-SB). The results from the primary outcomes were evaluated to assess whether they achieved the minimal clinically important differences (MCIDs) obtained in previous studies [13,14,35].

### Secondary (Biomarker and Safety) Outcomes

The secondary outcomes were biomarker and safety measures reported by all or most trials. The biomarkers included mean changes from baseline in the amyloid burden on positron emission tomography (PET; centiloids) and the cerebrospinal fluid (CSF) biomarkers of Aβ_1-42_ and p-tau-(Thr_181_). Safety outcomes were serious AEs, tolerability (treatment discontinuation due to AEs), and total events of ARIA-E (cerebral edema and sulcal effusion), ARIA-H (cerebral microhemorrhage and superficial siderosis), headaches, dizziness, falls, arthralgia, diarrhea, urinary infection, and nasopharyngitis.

### Tertiary Outcomes and Additional Measures

Tertiary outcomes included AEs reported by few studies only: total events of ARIA-E in *APOE*-ε4 carriers and noncarriers, fatigue, nausea, back pain, and upper respiratory infections.

Apart from the outcome measures, we extracted the inclusion criteria and baseline/participant characteristics of the primary studies, including mean age (years, SD), sex/ethnicity/race, *APOE* status, baseline ADAS-Cog, MMSE, and CDR-SB scores, dosage, and administration routes.

### Data Analysis, Reporting, and Web Application Development

As in our previous study [14], in order to properly manage the differences in study-specific and patient-specific characteristics within the subgroups of the included studies, we followed the recommendation to treat each subgroup as a separate study whenever possible [36]. If it was not feasible, the subgroups from the same study were combined (pooled) [37] in order to avoid correlated effect sizes and double-counting [38-40].

Since some interventions were tested in different dose regimens, the main pair-wise meta-analysis (according to our previous methodology [14]), and frequentist and Bayesian NMAs were performed by pooling data from high-dose and low-dose regimens. Simultaneously, 2 sensitivity analyses were performed: one including only the high-dose regimen and the other including the low-dose regimen, while keeping other drugs in a single-dose regimen. If multiple low-dose regimens were available, the clinically most relevant one (with a higher dose) was selected.

The NMA results have been reported as frequentist and Bayesian estimates of the relative and absolute benefits and risks of interventions. Any discrepancy in the findings between the 2 NMA methods has been highlighted and explained in a GRADE summary of findings. Relative measures included standardized mean differences (SMDs) for benefits and risk ratios (RRs, lnRRs) for AEs. For statistically significant results with no excessive heterogeneity, relative measures have been converted to absolute risks (ARs) and benefits, expressed as AR per 1000 people [39] and numbers needed to treat (NNT) for additional harmful (NNTH, higher is better) [12,41-43] and beneficial outcomes (NNTB, lower is better) [37,41,42]. In AlzMeta.app 2.0, these calculations are allowed for a wide range of assumptions concerning the risks of decline and AEs in the control group (with no treatment). This has been implemented as it can be important for calculating the benefits and risks for patient populations with different expectations of baseline risks, based on prior history and clinical expertise in each particular case.

The web application AlzMeta.app 2.0 [44] has been mainly built in RStudio 2024.09 (R 4.4.1) by modifying and expanding the previous instance of the application, relying on key packages such as *shiny*, *tidyverse*, *dmetar*, *metafor*, *ggplot2*, *netmeta*, *rjags*, and *gemtc*. Multimedia Appendix 2 provides a detailed overview of R-based statistical packages used for data analysis (explained further) as well as quality assessments (risk of bias and modified Jadad scale). CSS and HTML have been used for further styling of the graphical interface.

#### Frequentist NMA

In the frequentist NMA, SMDs and RRs have been calculated together with their 95% CIs [30] and 95% prediction intervals (PIs) [45], and the results have been presented via forest plots and tables that separate direct and indirect estimates. The interventions have been ranked by P-score [46], representing the mean extent of certainty that a treatment is better than the competing treatments. P-score can have a value between 0 and 1 and is equivalent to the surface under the cumulative ranking curve (SUCRA) score in the Bayesian framework. A higher P-score indicates more effective or safer treatment when compared to all other treatments in the network, averaged over all competing treatments.

The values of *I*^2^ statistics (95% CI) and their statistical significance were evaluated for overall heterogeneity or inconsistency in the network, which was then split into within-design (heterogeneity) and between-design (inconsistency), and the *P*-values of within-design Q-statistics have been reported. We followed the recommendations [36,47-50] to not include surrogate (biomarker) outcomes and findings with significant and excessive heterogeneity into the summary of findings, intervention rankings, and estimations of absolute effects.

#### Bayesian NMA

The Bayesian random effects NMA was performed in the Bayesian hierarchical model with Markov Chain Monte Carlo (MCMC) estimation [51]. Model parameters included 10 thinning iterations and 100,000 simulation iterations. The convergence of the MCMC model was assessed through Gelman-Rubin plots and the potential scale reduction factor. Trace and density plots were used to decide the optimal parameters of the number of burn-in iterations, actual simulation iterations, and thinning parameters.

The relative effects (SMDs and RRs) have been presented in a forest plot for each outcome. For clinically relevant results achieved by multiple interventions, ranking was performed by SUCRA scores based on posterior probabilities in the Bayesian framework [52]. SUCRA scores range from 0% to 100% (inclusive) and can be interpreted as the estimated proportion of treatments worse than a given treatment. Values close to 100% suggest greater probability that a given treatment is the best. More information can be found in the documentation section in AlzMeta.app 2.0 [44].

The Bayesian NMA was performed by 3 different choices of heterogeneity priors in both the main and sensitivity analyses. First, we used uninformative priors (with large variance) and half-normal priors for SD, with scale factors based on overall heterogeneity measured in the frequentist NMA. Then, the estimates were obtained by half-normal priors assuming 3 times greater heterogeneity than measured. All these calculations are available for both the relative (SMDs and RRs) and absolute estimates (NNTBs, NNTHs, and ARs) in AlzMeta.app 2.0, and the corresponding code can be found in Multimedia Appendix 2.

The Bayesian network meta-regression was performed to check whether specific study characteristics influenced the effect size estimates for the primary outcomes, including the impact of the number of study sites, study duration (weeks), mean age of participants, and baseline ADAS-Cog score.

#### Rank Robustness

The robustness of SUCRA ranks was determined by excluding one study at a time in the leave-one-out session and calculating (1) the level of agreement beyond chance (interrater reliability) via quadratic weighted Cohen kappa [53], (2) how many (%) studies did not change any treatment rank, and (3) how many (%) studies displaced treatment ranks. The Cohen kappa values are often classified into levels of agreement for interpretation, for example, poor (<0%), slight (0%-20%), fair (21%-40%), moderate (41%-60%), substantial (61%-80%), and almost perfect (81%-100%) agreement. However, it should be mentioned that this is an ad hoc procedure and different interpretations are available [54], as it depends on the context and specific requirements for reliability. Even values above 90% do not necessarily indicate almost perfect agreement [53,54].

#### Rank Uncertainty

The uncertainty of SUCRA ranks has been visualized by rankograms [55], which show the distribution of ranking probabilities for each treatment to be the *n*th best option. Furthermore, we calculated Shannon normalized (information) entropy associated with SUCRA ranking probabilities, as proposed by Wu et al [56]. The authors proposed a very intuitive way to estimate uncertainty associated with ranking probabilities in NMA by applying the Shannon information entropy formula to obtain a normalized entropy score. They showed that the normalized entropy score provides a more accurate assessment of ranking uncertainty and does not depend on the number of analyzed treatments. This approach can be used to compare the uncertainty of treatment rankings within a NMA and also between different NMAs, which is crucial for the interpretation of results.

For a NMA, the most precise scenario would be absolute certainty in the ranking of treatments in our network. Therefore, each treatment would have 100% probability of being in one ranking position and 0% probability for the other positions (peaked distribution). Under this scenario, the entropy is zero bit, and normalized entropy equals zero. On the other hand, the normalized entropy reaches 1 in the least precise scenario when the ranking probabilities are the same for each rank (flat distribution).

#### Correlations

The correlation calculated among the cognitive, functional, and biomarker outcomes was the Pearson product moment correlation coefficient (*r*). For safety measures, Pearson *r* was computed only for data not significantly different from normal distribution, as determined by the Shapiro-Wilk test. The Spearman rank correlation coefficient was used for data not following normal distribution.

#### Influence and Sensitivity Analyses

Influential studies were identified initially by standard meta-analytic approaches: heterogeneity assessments, Baujat plots, influence diagnostics, and leave-one-out approach. Then, robustness was further evaluated by machine learning methods within graphic display of study heterogeneity (GOSH) plot analysis, in which the meta-analysis model was fit to all possible subsets of included studies [57,58]. Sensitivity analyses were then performed upon excluding detected influential observations. Furthermore, in frequentist NMA, the influence of each study on the treatment estimates was estimated within the main and sensitivity analyses by assessing the proportional contributions of direct comparisons [59] and the statistical importance of each study measured by the reduction of precision if removed from the analysis [60].

#### Risk of Bias and Publication Bias

The risk of bias at the study, outcome, and comparison levels was classified as “low,” “raising some concerns,” or “high,” by using the revised Cochrane risk of bias (RoB-2) approach. Summary and study-level risk of bias was visualized via the RoB-2 web application [61,62], and the risk of bias at the outcome and comparison levels has been presented within the GRADE summary of findings tables. Publication bias was statistically evaluated for continuous outcomes with >10 studies [63] using the Egger regression test [37,64,65], and funnel plots were created using the Duval and Tweedie trim-and-fill procedure (Multimedia Appendix 2).

#### Certainty of Evidence and Impact of COVID-19

The certainty of underlying evidence at the comparison level within NMAs was assessed with rigorous adherence to the GRADE approach by focusing on within-study bias, reporting bias, indirectness, imprecision, heterogeneity, and incoherence [33]. The impact of the COVID-19 pandemic was assessed by evaluating (1) the number (%) of affected patients per arm, (2) whether the impact of COVID-19 was analyzed in the primary study, and (3) how the pandemic affected each trial stage, AEs, unblinding, dropouts, delays, and missing doses.

### Role of Funding Source

The funder of the study had no role in study design, data collection, analysis, interpretation, or writing of the report.

### Ethical Considerations

This study did not require ethics board review as it did not involve human participants, identifiable patient information, or medical records. The research was based entirely on publicly available trial-level data that have been fully and irreversibly anonymized and do not contain any personally identifiable information. Therefore, no identification of individual participant information is possible from this work [66].

## Results

### Baseline Characteristics of Included Studies

**Figure 1:**
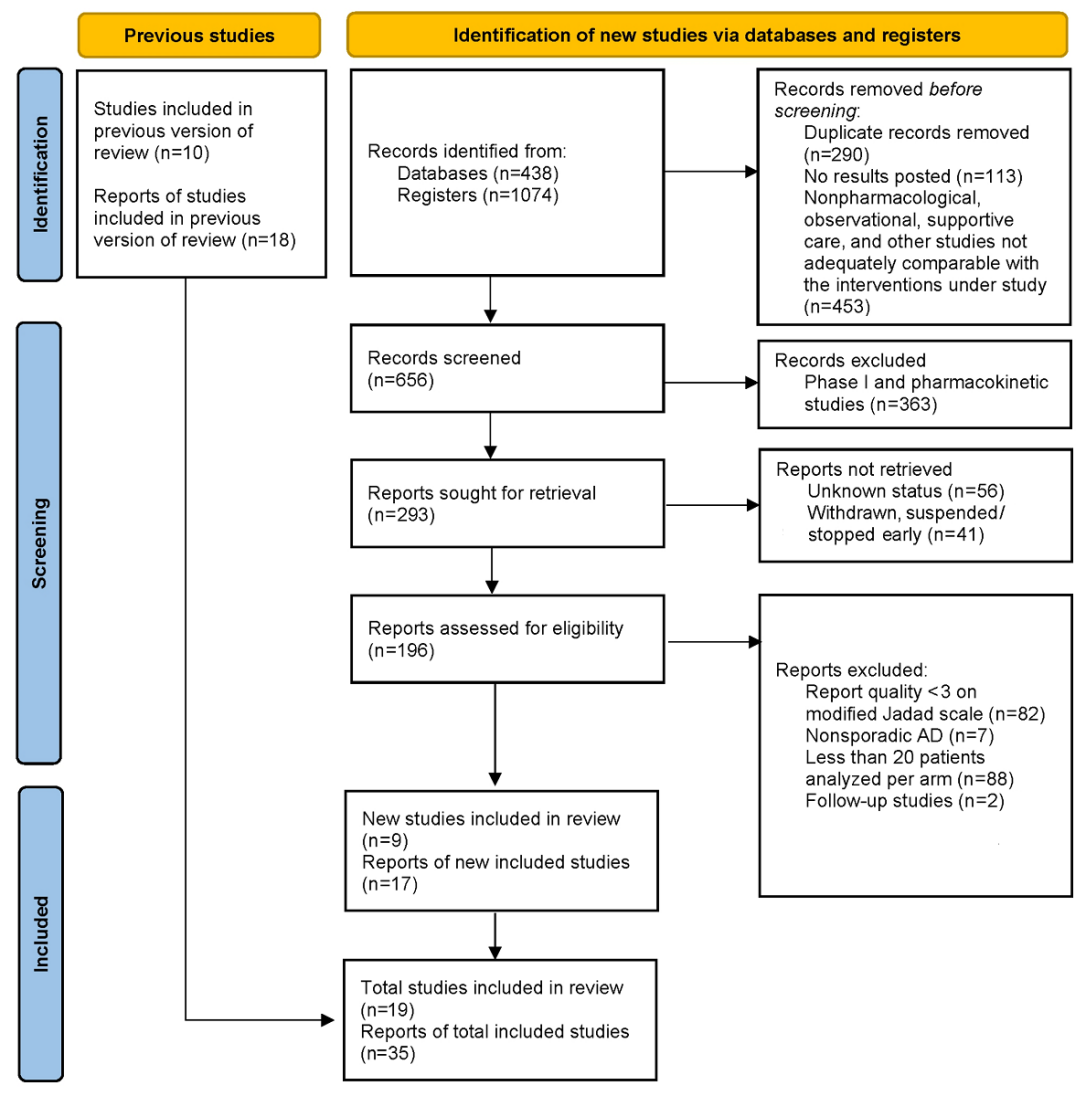
PRISMA (Preferred Reporting Items for Systematic Reviews and Meta-Analyses) flow diagram. AD: Alzheimer disease.

The meta-analysis included 21,236 patients with mild cognitive impairment (MCI) and early and mild-to-moderate sporadic AD in 26 studies (19 ClinicalTrials.gov registrations of phase II and III RCTs; Figure 1). Seven antiamyloid monoclonal antibodies were evaluated: bapineuzumab [17,18,22], gantenerumab [25,26], aducanumab [15], solanezumab [19,20], lecanemab [16,21], donanemab [23,24], and crenezumab [27,67]. The analyzed RCTs compared the interventions with placebo during 82.8 weeks on average (69-116 weeks), with acetylcholinesterase inhibitors and memantine allowed (alone or combined). Table 1 provides an overview of the baseline participant characteristics of the primary studies. Further baseline characteristics and the inclusion and exclusion criteria of the primary studies are presented in Multimedia Appendices 3 and 4, respectively. The results of the quality of report assessments (modified Jadad scale) and the specific reasons for the exclusion of each study can be found in Multimedia Appendices 5 and 6, respectively. Regarding the risk of bias, all studies were considered as having “low risk” or “raising some concerns” (Multimedia Appendix 7). For clinically relevant outcomes, summary of findings tables with rated certainty of evidence (vs placebo) according to the GRADE approach can be found in Figures S1-S8 in Multimedia Appendix 8. The complete GRADE table with certainty of evidence for each comparison within the NMA can be found in Multimedia Appendix 9.

**Table 1:** Baseline participant characteristics.

Study and dose	Dosage	Patients (placebo vs intervention), n	AD^a^ stage (MMSE^b^ score for inclusion)
Salloway et al [22], 2009; high dose	1 mg/kg q13w^c^ for 78 weeks	22 vs 26	Mild-moderate (16-26)
Salloway et al [18], 2014; Study 301; low dose	0.5 mg/kg q13w for 78 weeks	493 vs 314	Mild-moderate (16-26)
Salloway et al [18], 2014; Study 301; high dose	1 mg/kg q13w for 78 weeks	493 vs 307	Mild-moderate (16-26)
Salloway et al [18], 2014; Study 302; low dose	0.5 mg/kg q13w for 78 weeks	432 vs 658	Mild-moderate (16-26)
Doody et al [20], 2014; EXPEDITION 1	400 mg q4w for 78 weeks	506 vs 506	Mild-moderate (16-26)
Doody et al [20], 2014; EXPEDITION 2	400 mg q4w for 78 weeks	519 vs 521	Mild-moderate (16-26)
Vandenberghe et al [17], 2016; low dose	0.5 mg/kg q13w for 78 weeks	328 vs 328	Mild-moderate (16-26)
Vandenberghe et al [17], 2016; high dose	0.5 mg/kg q13w for 78 weeks	328 vs 253	Mild-moderate (16-26)
Vandenberghe et al [17], 2016; low dose	1 mg/kg q13w for 78 weeks	431 vs 650	Mild-moderate (16-26)
Honig et al [19], 2018; EXPEDITION 3	400 mg q4w for 76 weeks	1057 vs 1072	Mild (20-26)
Budd Haeberlein et al [15], 2022; EMERGE; low dose	Titrated to a target dose of 3 mg/kg (ε4^+^) or 6 mg/kg (ε4^-^) q4w over 76 weeks	548 vs 543	Early (MCI^d^ and mild) (24-30)
Budd Haeberlein et al [15], 2022; EMERGE; high dose	Titrated to a target dose of 6 mg/kg (ε4^+^) or 10 mg/kg (ε4^-^) q4w over 76 weeks	548 vs 547	Early (MCI and mild) (24-30)
Budd Haeberlein et al [15], 2022; ENGAGE; low dose	Titrated to a target dose of 3 mg/kg (ε4^+^) or 6 mg/kg (ε4^-^) q4w over 76 weeks	545 vs 547	Early (MCI and mild) (24-30)
Budd Haeberlein et al [15], 2022; ENGAGE; high dose	Titrated to a target dose of 6 mg/kg (ε4^+^) or 10 mg/kg (ε4^-^) q4w over 76 weeks	545 vs 555	Early (MCI and mild) (24-30)
van Dyck et al [16], 2023; Clarity AD	10 mg/kg every 2 weeks over 78 weeks	875 vs 859	Early (MCI and mild) (20-26)
Swanson et al [21], 2021; high dose	10 mg/kg every 2 weeks over 78 weeks	238 vs 152	Early (MCI and mild) (22-30)
Swanson et al [21], 2021; low dose	10 mg/kg every month for 78 weeks	238 vs 246	Early (MCI and mild) (22-30)
Sims et al [24], 2023; TRAILBLAZER-ALZ 2 (pooled)	700 mg for the first 3 doses and 1400 mg thereafter q4w over 72 weeks	876 vs 860	Early (MCI and mild) (20-28)
Mintun et al [23], 2021; TRAILBLAZER-ALZ	700 mg for the first 3 doses and 1400 mg thereafter q4w over 72 weeks	126 vs 131	Early (MCI and mild) (20-28)
Bateman et al [25], 2023; GRADUATE I	Minimum 3 doses at each stage: starting at 120 mg q4w and increasing to 255 mg q4w, then to 510 mg q4w, and finally to 510 mg q2w	485 vs 499	Early (MCI and mild) (≥22)
Bateman et al [25], 2023; GRADUATE II	Minimum 3 doses at each stage: starting at 120 mg q4w and increasing to 255 mg q4w, then to 510 mg q4w, and finally to 510 mg q2w	477 vs 498	Early (MCI and mild) (≥22)
Ostrowitzki et al [27], 2022; CREAD	60 mg/kg q4w for up to 100 weeks	86 vs 80	Early (prodromal-mild) (≥20)
Salloway et al [67], 2018; BLAZE (pooled)	Part 1: 300 mg subcutaneous (q2w); Part 2: 15 mg/kg intravenous (q4w)	29 vs 62	Mild-moderate (18-26)
Ostrowitzki et al [26], 2017; SCarlet RoAD I	105 mg by subcutaneous injection (q4w) for 104 weeks or nearly 2 years	133 vs 271	Early (≥24)
Ostrowitzki et al [26], 2017; SCarlet RoAD II	225 mg by subcutaneous injection (q4w) for 104 weeks or nearly 2 years	133 vs 260	Early (≥24)

^a^AD: Alzheimer disease.

^b^MMSE: Mini-Mental State Examination.

^c^qXw: once every X weeks.

^d^MCI: mild cognitive impairment.

### Primary Outcomes

Regarding the ADAS-Cog, 5 antiamyloid antibodies were significantly more effective than placebo in the frequentist NMA (Table 2; Figure S1 in Multimedia Appendix 8). These showed small effect sizes (donanemab > gantenerumab > lecanemab > aducanumab > solanezumab), with PIs for solanezumab including zero, suggesting a possible nonsignificant difference from placebo. The Bayesian NMA validated these findings, with strong support for the effects of donanemab, gantenerumab, lecanemab, and aducanumab, and a lack of strong evidence for solanezumab.

In the MMSE, donanemab and solanezumab were the only treatments superior to placebo, with small effect sizes in the frequentist NMA (Table 2; Figure S2 in Multimedia Appendix 8). Convincing evidence in the Bayesian NMA was found only for the effects of donanemab.

In the CDR-SB, both the frequentist and Bayesian NMAs conclusively showed that donanemab was significantly more effective than placebo and all the other antibodies. While aducanumab (*P=*.04) and lecanemab (*P=*.005) demonstrated relative benefits over placebo in the frequentist NMA with small effect sizes and CIs/PIs very close to zero (Table 2; Figure S3 in Multimedia Appendix 8), the Bayesian NMA results did not provide conclusive support for these effects. In other words, while donanemab was superior to placebo and other antibodies on the CDR-SB, lecanemab and aducanumab could be associated with slightly higher benefits than placebo. However, we cannot confidently rule out the possibility of no significant difference.

Heterogeneity was nonsignificant for primary outcomes (*I*^2^=0%), and inconsistency was not assessed because the networks were lacking closed loops. No evidence of publication bias was found, with Egger test *P*-values of .55, .91, and .80 for the ADAS-Cog, MMSE, and CDR-SB, respectively. Funnel plots were symmetric for primary outcomes, and the imputation of potentially “missing studies” did not change the results. The Bayesian network meta-regression revealed no impact of study characteristics (number of study sites, age of participants, and baseline ADAS-Cog score) on the effect size estimates for primary outcomes.

**Figure 2:**
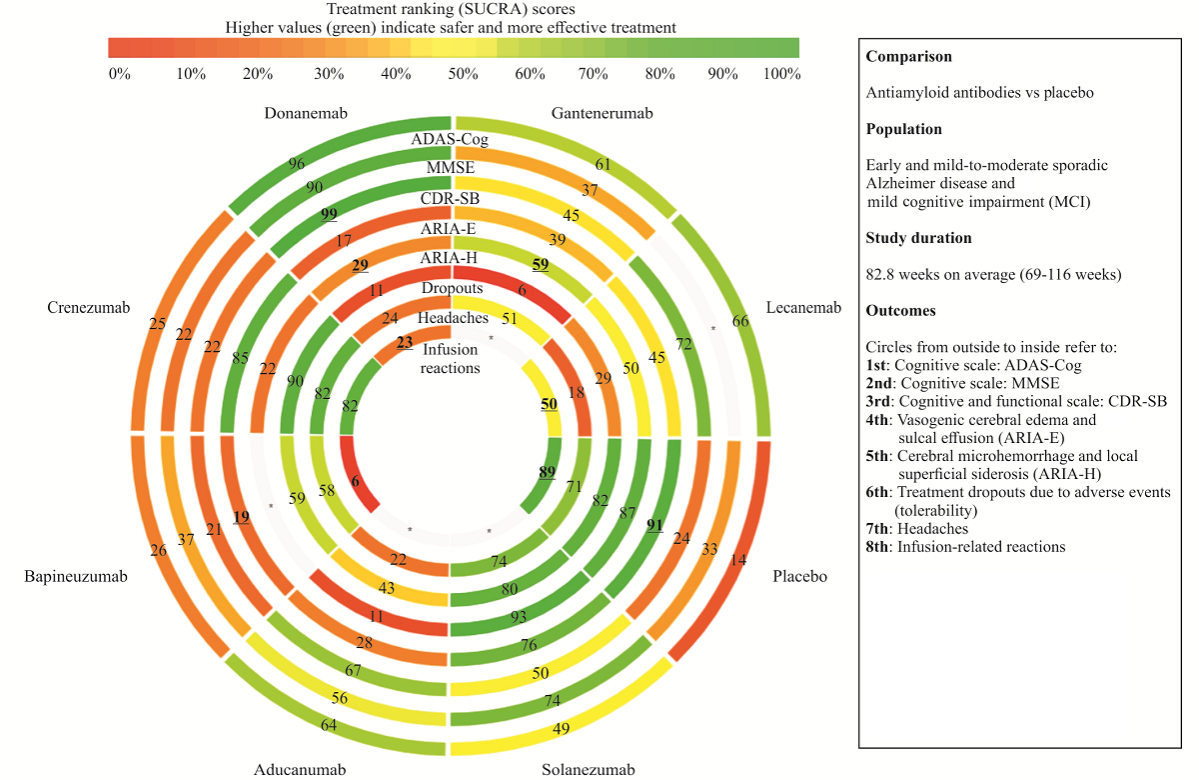
Surface under the cumulative ranking curve (SUCRA) rank-heat plot for clinically relevant results achieved by more than one intervention. Bolded and underlined SUCRA values indicate moderate-to-high certainty in the ranking probabilities, with normalized Shannon information entropy lower than 0.5. ADAS-Cog: Alzheimer Disease Assessment Scale-Cognitive Subscale; CDR-SB: Clinical Dementia Rating Scale-Sum of Boxes; MMSE: Mini-Mental State Examination. *Lack of data on the corresponding outcome within the circle.

Among all primary outcomes, donanemab outperformed other antibodies according to P-scores in the frequentist framework and SUCRA scores in the Bayesian framework. SUCRA rankings were generally robust to study exclusions (Multimedia Appendix 10), and high certainty (low information entropy) in ranking probabilities was found for the CDR-SB, where donanemab produced the clinically most relevant effects (Table 2; Figure 2).

**Table 2:** Frequentist network meta-analysis summary of findings: clinical benefits on cognitive and functional measures.

Measure and drug		RCT^a^ and patient count	Relative effect, SMD^b^ (95% CI)	Absolute effect, NNTB^c^ (95% CI)	Ranking^d^, P-score; SUCRA (rank uncertainty)	Evidence certainty^e^	Comparison to placebo
**ADAS-Cog^f^**							
	DON^g^	3 RCTs and 1469 patients	–0.22 (–0.32 to –0.12)	_14 (9 to 26)_	0.97; 96.3% (0.81)	Low^h^	Probably better
	LEC^i^	3 RCTs and 2109 patients	–0.11 (–0.19 to –0.02)	_29 (16 to 157)_	0.67; 66.0% (0.94)	Very low^j^	Probably better
	ADU^k^	4 RCTs and 2301 patients	–0.10 (–0.18 to –0.02)	_31 (17 to 157)_	0.65; 64.2% (0.95)	Very low^j^	Probably better
	GAN^l^	3 RCTs and 4172 patients	–0.10 (–0.18 to –0.01)	_33 (17 to 314)_	0.62; 60.6% (0.93)	Very low^j^	Probably better
	SOL^m^	3 RCTs and 4172 patients	–0.07 (–0.13 to –0.01)	_47 (24 to 524)_	0.48; 48.6% (0.93)	Very low^n^	Possibly better; no conclusive difference
**MMSE^o^**							
	DON	3 RCTs and 1460 patients	0.14 (0.04 to 0.25)	_21 (12 to 131)_	0.92; 90.2% (0.53)	Very low^h^	Probably better
	SOL	3 RCTs and 3821 patients	0.09 (0.02 to 0.15)	_47 (24 to 524)_	0.76; 73.8% (0.74)	Very low^n^	Possibly better; no conclusive difference
**CDR-SB^p^**							
	DON	3 RCTs and 1452 patients	–0.29 (–0.40 to –0.19)	_10 (8 to 16)_	1.00; 99.0% (0.27)	Low^h^	Probably better
	LEC	3 RCTs and 2128 patients	–0.12 (–0.21 to –0.03)	_25 (15 to 104)_	0.75; 72.46% (0.81)	Very low^n^	Possibly better; no conclusive difference
	ADU	4 RCTs and 2301 patients	–0.10 (–0.20 to –0.002)	_31 (15 to 767)_	0.68; 67.5% (0.87)	Very low^n^	Possibly better; no conclusive difference

^a^RCT: randomized controlled trial.

^b^SMD: standardized mean difference.

^c^NNTB: number needed to treat for an additional beneficial outcome (assumed control-event rate of 0.25).

^d^In the frequentist network meta-analysis, the treatments are ranked with P-scores that measure the mean extent of certainty that a given treatment is better than the competing treatments, ranging from 0 to 1 (higher is better). In the Bayesian framework, the surface under the cumulative ranking curve (SUCRA) score (0%-100%) shows the average percentage of treatments that are worse than a given treatment. The closer the SUCRA score is to 100%, the more likely it is that the treatment is the best option. Rank uncertainty is measured by the normalized Shannon entropy score of Bayesian ranking probabilities, ranging from 0 (absolute certainty) to 1 (absolute uncertainty).

^e^Grades: High, we are very confident that the true effect size lies close to that of the estimated effect; Moderate, we are moderately confident in the estimate, and the true effect size is likely to be close to our estimate, with the possibility that it is substantially different; Low, our confidence in the effect estimate is limited, and the true effect may be substantially different from the estimate of the effect; Very low, we have very little confidence in the effect estimate, and the true effect is likely to be substantially different from the estimate of the effect.

^f^ADAS-Cog: Alzheimer Disease Assessment Scale-Cognitive Subscale (lower SMD is better).

^g^DON: donanemab.

^h^Due to small effect size, imprecision, and wide prediction and credible intervals.

^i^LEC: lecanemab.

^j^Due to small effect size, imprecision, and wide prediction and credible intervals very close to zero.

^k^ADU: aducanumab.

^l^GAN: gantenerumab.

^m^SOL: solanezumab.

^n^In the frequentist network meta-analysis, the SMDs were statistically significant in the ADAS-Cog (*P*=.03) and MMSE (*P*=.007) for solanezumab, and in the CDR-SB for lecanemab (*P*=.007) and aducanumab (*P*=.046), but with confidence and prediction intervals including zero or very close to zero and large NNTB values. This suggests that these effects are unlikely to be clinically relevant. In the Bayesian framework, the interventions could be associated with a slightly higher benefit than placebo for the respective outcomes, but with credible intervals including zero; therefore, we cannot confidently rule out the possibility of no significant difference.

^o^MMSE: Mini-Mental State Examination (higher SMD is better).

^p^CDR-SB: Clinical Dementia Rating Scale-Sum of Boxes (lower SMD is better).

### Secondary Outcomes

#### Biomarkers of Aβ and p-tau

Biomarker analyses (available on AlzMeta.app) revealed more promising results of the approved antibodies. Both the frequentist and Bayesian NMAs of the PET data conclusively showed that 4 treatments significantly reduced Αβ brain burden by large effect sizes (donanemab > gantenerumab > lecanemab > aducanumab). Biomarkers of Aβ_1-42_ in the CSF were improved by 4 antibodies in the frequentist NMA (crenezumab > aducanumab > lecanemab > gantenerumab; not reported for donanemab); however, the Bayesian NMA provided strong support only for the effects of crenezumab and aducanumab. The results of amyloid biomarkers (CSF and PET) were not correlated with the mean changes observed in the primary outcomes.

CSF p-tau was significantly improved with 3 antibodies (aducanumab > gantenerumab > lecanemab; not reported for donanemab); however, the Bayesian NMA validated only the effects of aducanumab versus placebo. CSF p-tau measures were positively correlated with the effect sizes on the ADAS-Cog (*r*=0.77; *P=*.03) and CDR-SB (*r*=0.77; *P=*.03), with almost identical correlation coefficients and *P*-values for aducanumab, lecanemab, bapineuzumab, gantenerumab, and crenezumab.

#### Safety Outcomes

Antiamyloid antibodies did not increase the risk of serious AEs. However, 4 interventions (gantenerumab > donanemab > lecanemab > aducanumab) were less tolerable than placebo since they increased treatment discontinuations due to AEs by large effect sizes (Table 3; Figure S4 in Multimedia Appendix 8). Crenezumab and solanezumab were the most tolerable and safest antibodies in terms of ARIA-E and ARIA-H. All other antibodies substantially increased the risk of total ARIA-E (Table 3; Figure S5 in Multimedia Appendix 8). Heterogeneity was not significant for the risks of ARIA-E (*I*^2^=33.7%) and treatment dropouts due to AEs (*I*^2^=0%).

**Table 3:** Bayesian network meta-analysis summary of findings: clinically relevant risks.

Adverse event and drug			RCT^a^ and patient count		Relative risk, RR^b^ (95% CI)	Absolute effects^c^ (95% CI)					Ranking^f^, P-score; SUCRA (rank uncertainty)			Evidence certainty^g^			Comparison to placebo
						NNTB^d^, value (95% CI)		AR^e^ per 1000, value (95% CI)									
**ARIA-E^h^ (ACR^i^=0.01)**																	
	LEC^j^	3 RCTs and 2454 patients		8.23 (4.33-15.64)		_14 (7-31)_	_77 (42-143)_		0.46; 44.94% (0.78)			Moderate^k^			Probably worse		
	GAN^l^	4 RCTs and 2736 patients		9.36 (5.70-15.37)		_12 (7-22)_	_91 (56-143)_		0.40; 39.31% (0.78)			Moderate^k^			Probably worse		
	ADU^m^	4 RCTs and 3249 patients		11.40 (6.94-18.73)		_10 (6-17)_	_112 (67-167)_		0.27; 28.38 (0.79)			Moderate^k^			Probably worse		
	BAP^n^	6 RCTs and 4278 patients		12.97 (6.82-24.65)		_9 (5-18)_	_125 (67-200)_		0.19; 18.38% (0.72)			Moderate^k^			Probably worse		
	DON^o^	3 RCTs and 1981 patients		13.60 (7.58-24.38)		_8 (5-16)_	_125 (72-200)_		0.16; 17.71% (0.71)			Moderate^k^			Probably worse		
**ARIA-H^p^ (ACR=0.10)**																	
	GAN	3 RCTs and 1770 patients		1.70 (1.36-2.14)		_15 (9-28)_	_167 (125-200)_		0.59; 58.62% (0.85)			Moderate^k^			Probably worse		
	LEC	3 RCTs and 2454 patients		1.89 (1.48-2.41)		_12 (8-21)_	_167 (143-200)_		0.50; 49.56% (0.86)			Moderate^k^			Probably worse		
	DON	3 RCTs and 1981 patients		2.43 (2.00-2.95)		_8 (6-11)_	_200 (167-250)_		0.21; 28.57% (0.86)			Moderate^k^			Probably worse		
	ADU	4 RCTs and 3253 patients		3.39 (2.75-4.18)		_5 (4-6)_	_334 (250-334)_		0.10; 11.39% (0.82)			Moderate^k^			Probably worse		
**Dropouts (ACR=0.05)**																	
	BAP	7 RCTs and 4507 patients		1.24 (1.01-1.52)		_82 (28-1450)_	_59 (50-72)_		0.59; 59.29% (0.78)			Very low^q^			Possibly worse; no conclusive difference		
	ADU	4 RCTs and 3249 patients		1.65 (1.07-2.54)		_31 (13-283)_	_77 (53-125)_		0.43; 43.20% (0.83)			Low^r^			Probably worse		
	LEC	3 RCTs and 2454 patients		2.18 (1.54-3.07)		_17 (10-37)_	_100 (77-143)_		0.29; 28.85% (0.74)			Moderate^k^			Probably worse		
	DON	3 RCTs and 1981 patients		3.04 (2.19-4.23)		_10 (7-17)_	_143 (100-200)_		0.11; 11.29% (0.66)			Moderate^k^			Probably worse		
	GAN	4 RCTs and 2756 patients		3.44 (2.32-5.10)		_5 (3-10)_	_250 (143-334)_		0.05; 6.21% (0.38)			Moderate^k^			Probably worse		
**Infusion-related reactions (ACR=0.04)**																	
	LEC	3 RCTs and 2454 patients		4.08 (2.87-5.80)		_9 (6-14)_	_143 (112-200)_		0.50; 49.69% (0.42)			Very low^q^			Possibly worse; no conclusive difference		
	DON	3 RCTs and 1981 patients		17.65 (6.71-46.43)		_2 (1-5)_	_500 (250-1000)_		0.22; 23.29% (0.47)			Moderate^k^			Probably worse		
	BAP	1 RCT and 1121 patients		68.56 (9.39-500.85)		_1 (1-3)_	_1000 (334-1000)_		0.03; 6.17% (0.38)			Very low^s^			Probably worse		

^a^RCT: randomized controlled trial.

^b^RR: risk ratio.

^c^For safety outcomes, absolute effects are expressed as absolute risk per 1000 people and as number needed to treat for an additional beneficial outcome, with assumed control risks equal to the average control risk for each outcome, weighted by the number of participants that received placebo.

^d^NNTB: number needed to treat for an additional beneficial outcome.

^e^AR: absolute risk.

^f^In the Bayesian framework, the surface under the cumulative ranking curve (SUCRA) score shows the average percentage of treatments that are worse than a given treatment, which can range from 0% to 100%. The closer the SUCRA score is to 100%, the more likely it is that the treatment is the best option. Rank uncertainty is measured by the normalized Shannon entropy score of Bayesian ranking probabilities, ranging from 0 (absolute certainty) to 1 (absolute uncertainty).

^g^Grades: High, we are very confident that the true effect size lies close to that of the estimated effect; Moderate, we are moderately confident in the estimate, and the true effect size is likely to be close to our estimate, with the possibility that it is substantially different; Low, our confidence in the effect estimate is limited, and the true effect may be substantially different from the estimate of the effect; Very low, we have very little confidence in the effect estimate, and the true effect is likely to be substantially different from the estimate of the effect.

^h^ARIA-E: amyloid-related imaging abnormalities in the form of vasogenic cerebral edema and sulcal effusion.

^i^ACR: assumed control risk.

^j^LEC: lecanemab.

^k^Due to a large magnitude of the effect size.

^l^GAN: gantenerumab.

^m^ADU: aducanumab.

^n^BAP: bapineuzumab.

^o^DON: donanemab.

^p^ARIA-H: amyloid-related imaging abnormalities in form of cerebral microhemorrhage and local siderosis.

^q^In the frequentist network meta-analysis, statistically significant risk was found in the infusion-related reactions for lecanemab (*P*<.001) and the treatment discontinuations for bapineuzumab (*P=*.04), but with prediction intervals close to 1 or including 1 (for bapineuzumab), suggesting that these effects are unlikely to be clinically relevant. In the Bayesian framework, these treatments could be associated with a slightly higher risk than placebo on the respective outcomes, but with credible intervals including 1; thus, we cannot confidently rule out the possibility of no significant difference.

^r^Due to wide credible intervals and imprecision.

^s^Due to wide credible intervals, imprecision, and a small number of studies.

The risk of ARIA-H was significant for 4 antibodies (aducanumab > donanemab > lecanemab > gantenerumab) (Table 3; Figure S6 in Multimedia Appendix 8), demonstrated conclusively by the frequentist and Bayesian NMAs. These estimates were obtained after excluding 1 influential study of gantenerumab (GRADUATE II) [25] that induced heterogeneity in the full dataset analysis.

**Figure 3:**
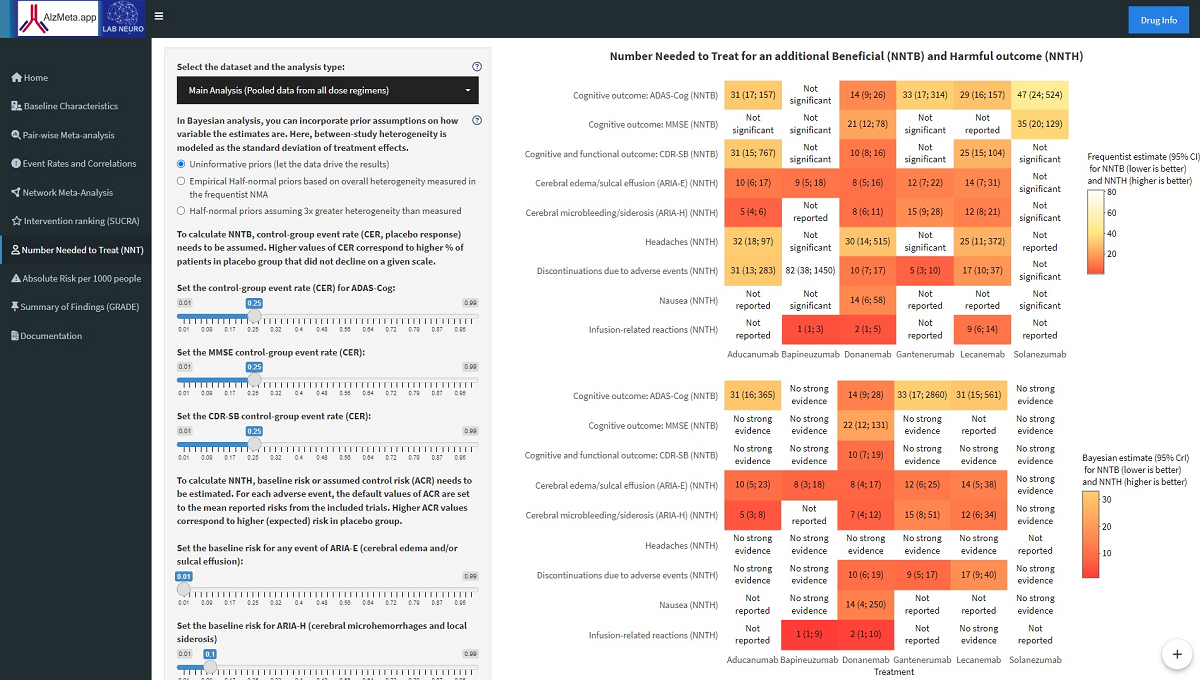
Number needed to treat (NNT) section in AlzMeta.app 2.0 showing the frequentist and Bayesian estimates of NNT for an additional beneficial (NNTB) and harmful outcome (NNTH) for statistically significant results. The estimates can be obtained over a wide range of placebo responses, including the rates of Alzheimer disease progression and adverse event risks expected in the control group. Similarly, the absolute risk per 1000 people can be calculated based on assumed control risk. Furthermore, the Bayesian network meta-analysis (NMA) can be performed with different choices of heterogeneity priors (Multimedia Appendix 2): (1) uninformative priors (with large variance), (2) half-normal priors for SD, with scale factors based on overall heterogeneity measured in the frequentist NMA, and (3) half-normal priors assuming 3 times greater heterogeneity.

Additional safety analyses showed that anti-Aβ antibodies did not raise the risks of dizziness, arthralgia, diarrhea, or urinary infections more than placebo. No significant heterogeneity was found with all interventions analyzed, except for headaches (*I*^2^=69%) and urinary infections (*I*^2^=53.3%), and excluding influential bapineuzumab [17] studies significantly reduced heterogeneity for both outcomes, leading to more certain estimates. This revealed that the risk of headaches was higher with aducanumab, lecanemab, and donanemab in the frequentist NMA (Figures 2 and 3; Figure S7 in Multimedia Appendix 8). However, the Bayesian NMA did not provide conclusive evidence for these comparisons, and frequentist PIs included 1 for all drugs, except for aducanumab. In addition, we found no enhanced risks of nasopharyngitis; however, these events were not reported for donanemab and lecanemab, which were antibodies tested during the COVID-19 pandemic (Multimedia Appendix 11).

Significant positive correlations were found between the sample sizes of the intervention and control groups and the occurrences of various treatment-related AEs and potential nocebo effects. These AEs included nausea (*r*>0.96), dizziness (*r*>0.89), headaches (*r*>0.87), serious AEs (*r*>0.76), and treatment or placebo discontinuations due to AEs (*r*>0.54). Additionally, when all interventions were analyzed, no correlation was observed between the total number of ARIA-E events and the sample size in the treatment group, while a moderate correlation was found in the control group (*r*=0.52; *P=*.03). The correlations between ARIA-E events and sample sizes became significantly stronger after excluding outlying solanezumab studies [19,20], with *r*=0.67 (*P=*.004) in the control group and *r*=0.73 (*P=*.002) in the treatment group. No significant correlations were identified between the primary outcomes and safety outcomes, including ARIA events.

The risk of ARIA-E was clinically relevant (Table 3; Figure 3) for 5 antibodies (donanemab > bapineuzumab > aducanumab > gantenerumab > lecanemab). Donanemab and bapineuzumab were the least safe treatments based on the P-score and SUCRA score (Figure 2). For all these antibodies, except for bapineuzumab, the risk of treatment discontinuation due to AEs was also clinically meaningful (Table 3; Figure 3). Gantenerumab was the least tolerable, followed by donanemab. Safety SUCRA rankings (Figure 2) remained moderately to highly stable to study exclusion, with 89% (17/19), 50% (8/16), and 82% (9/11) of studies not affecting any rank for tolerability, ARIA-E, and ARIA-H, respectively (Multimedia Appendix 10).

### Tertiary Outcomes

Given the limited information about tertiary outcomes, our results indicate that bapineuzumab, donanemab, lecanemab, aducanumab, and gantenerumab achieved clinically relevant risks of ARIA-E in both *APOE*-ε4 carriers and noncarriers (available in AlzMeta.app). The risk was higher in the carrier population, as reflected by CIs and PIs and lower *P*-values within the frequentist NMA. The higher risk in carriers was particularly noticeable for aducanumab and gantenerumab (approximately 3 times greater risk) and bapineuzumab (approximately 2 times greater risk) and less pronounced for donanemab and lecanemab, where a lower number of events was reported in noncarriers, giving more uncertain estimates.

When compared to placebo, 3 antibodies (bapineuzumab > donanemab > lecanemab) significantly increased the risk of infusion-related reactions (Table 2; Figure 3; Figure S8 in Multimedia Appendix 8), which showed mostly mild to moderate severity. These risks were all clinically relevant. Lecanemab was a relatively safer treatment option when compared to donanemab and bapineuzumab (Figure 2), with moderate uncertainty in ranking probabilities and credible intervals including 1 in the Bayesian framework. However, these estimates should be interpreted with caution due to the small number of studies and treatments included in the comparisons, leading to poor robustness of the SUCRA rankings (Multimedia Appendix 10).

Donanemab increased the risk of nausea in 2 out of 3 treatment arms [23,24]. AR measures revealed that the risk of nausea was clinically relevant, but with broad CIs (Figure 3) and overlapping prediction and credible intervals, suggesting considerable uncertainty in the estimates. Further analyses showed that the anti-Aβ antibodies did not increase the risk of fatigue, back pain, or upper respiratory infections more than placebo [24,25].

### Sensitivity Analyses

After excluding influential studies (Multimedia Appendix 12), we found greater risks and benefits of high-dose lecanemab and aducanumab (in the controversial EMERGE study [15,68]) for safety outcomes (ARIA-E, ARIA-H, and headaches), cognitive/functional outcomes (ADAS-Cog and CDR-SB), and biomarker outcomes (CSF and PET). Moreover, the risk of ARIA-E was dose-dependent for bapineuzumab, with a greater risk associated with a high dose. For low-dose aducanumab, no significant risk was found for treatment discontinuations due to AEs.

The dose-dependent efficacy of aducanumab and lecanemab was clinically meaningful on the ADAS-Cog and CDR-SB, with greater absolute benefits of high-dose regimens. However, the intervention with donanemab was still the best treatment option, being almost 2 times more effective than high-dose aducanumab and lecanemab. Further sensitivity analysis showed that donanemab had consistently greater cognitive and functional effects in patients with low or medium tau load than in the high-tau population. Still, even upon removing the low or medium tau study, donanemab remained the best-ranked treatment, outperforming the other drugs on cognitive and functional measures.

### Impact of the COVID-19 Pandemic

Among the studies included in this work, 2 gantenerumab studies [25], 1 lecanemab study [16], and 3 donanemab studies [23,24] were affected by the global outbreak of coronavirus, which caused delays in study visits and assessments, possible unblinding of some participants, and other difficulties, which have been summarized in Multimedia Appendix 11. The greatest impact was found in 2 phase III donanemab studies [24], with COVID-19 being the most commonly reported AE and being diagnosed in 16.0% (136/852) of patients in the control group and 17.6% (154/873) of patients in the group that received donanemab. A less severe impact was found in a lecanemab phase III study [16], with COVID-19 diagnosed in 7.1% (64/901) of patients in the control group and 6.7% (60/896) of patients in the intervention group. The reported impact of the pandemic was minimal in other studies.

## Discussion

### Principal Findings

The findings of this study support the effectiveness of donanemab and lecanemab, revealing small effect sizes for cognitive and functional outcomes, as well as moderate and large effect sizes for the CSF biomarkers of Aβ and p-tau, respectively. Notably, donanemab demonstrated greater benefits in patients with lower tau burden, suggesting a more pronounced effect in the earlier stages of AD. At the same time, the risks of ARIA-E, ARIA-H, and infusion-related reactions were substantial, indicating greater harm than benefit for all approved antibodies. The risks for donanemab were similar and potentially worse when compared to lecanemab and aducanumab. Lecanemab was a slightly safer and more tolerable option than donanemab and aducanumab, with lower risks of ARIA events and infusion-related reactions.

### Comparison to Prior Work

The findings presented here are consistent with our previous research [14] and other meta-analyses on anti-Aβ antibodies [8,9,35,69-72]. Based on the evidence so far, lecanemab and donanemab achieved statistical significance at attenuating cognitive and functional decline; however, the effect sizes observed in clinical trials were far below the MCID [1,35,69,71,72]. None of the studies reported so far found other clinically important results, such as a reduction in functional dependence, placement in memory care units or nursing homes, caregiver burden, or development of aggressive behaviors [69]. Although the cognitive and functional benefits of these drugs remain uncertain and show small effect sizes, they effectively demonstrated target engagement and disease-modifying potential by reducing Aβ load and improved multiple biomarker measures linked to AD pathology, including amyloid burden on PET and CSF Aβ_40_, Aβ_42_, and p-tau [16,24,71]. Therefore, direct comparative and longitudinal studies are required to fully disclose the impact of these disease-modifying effects over longer periods of time and better assess treatment-emergent risks in different populations. Exploratory post hoc modeling based on a phase III RCT of donanemab [24] suggested that Aβ levels in treated patients would remain below the positivity threshold for nearly 4 years without treatment, although it is uncertain whether the MCID would be achieved in the long term on cognitive and functional outcomes, since the effect sizes obtained so far are very low.

Drawing from our findings and earlier research, treatment initiation in earlier AD stages is expected to be more beneficial [24,71]; however, prolonged monitoring is essential to determine whether these benefits translate into sustained clinical advantages over time and whether there is a cumulative benefit that meets or exceeds the MCID. For clinicians, the MCID depends on the detected change from the last evaluation; however, a measure obtained in this way does not account for cost-effectiveness and may be variously applied across different studies, medical centers, and health care systems. Moreover, MCIDs derived from population averages may not reflect the diversity of experiences among AD patients, and small cognitive improvements observed with anti-Aβ therapies might be highly meaningful for a patient or setting but negligible for another. Ultimately, multidisciplinary evaluation integrating clinical, patient-reported, and economic outcomes is necessary to determine the true value of these treatments [13,73]. While the MCID remains useful, it should be complemented by additional clinically relevant metrics, such as NNTs, under a wide range of assumptions regarding the baseline risks of AEs and AD progression [12,74-76] and cost-effectiveness, and further modified by personalized thresholds based on real-world data addressing the complexities of AD, quality of life, and the various needs of patients, caregivers, and health care systems [13].

### Strengths and Limitations

One of the main strengths of our study is its capacity to provide real-time estimates of both relative and absolute risks and benefits of interventions within multiple datasets and dose regimens. These calculations are performed based on user-specific prior assumptions concerning heterogeneity and expected control responses (Figure 3), and the intervention rankings are accompanied by measures of their robustness and uncertainty. To the best of our knowledge, this approach was not used in previous studies, and it allows researchers and clinicians to incorporate their own expectations based on clinical expertise and novel evidence, ensuring that the findings remain relevant and adaptable to various clinical scenarios. We have combined the frequentist and Bayesian approaches to NMA, allowing users to easily obtain the results with different models and examine their discrepancies. The frequentist approach, while widely taught at all education levels and valued for offering straightforward interpretations, is often criticized for its reliance on fixed assumptions about data distributions and its inability to formally incorporate prior knowledge into the analysis. This can limit its applicability and generalizability in clinical contexts where data may be sparse or uncertain. In contrast, Bayesian methods provide a flexible framework that incorporates prior information, which can improve the robustness of estimates and more comprehensive quantification of uncertainty. While Bayesian methods have gained substantial momentum in recent decades of research, it remains less recognized than the frequentist approach and is often avoided due to perceived complexities or misconceptions. Researchers and clinicians may hesitate to engage with Bayesian methods due to computational demands or unfamiliarity with its principles and interpretation [57,75-79].

Our web application (available in English and Spanish upon peer review of this work) is very easy to use and provides extensive insights into the meta-analytic methodology, data structure, and valuable information that can serve educational purposes for anyone interested in meta-analysis and these therapies. It can be a useful tool for students, medical practitioners, and researchers to gain insights into meta-analysis techniques, including conventional pair-wise meta-analyses, and frequentist and Bayesian NMAs. Researchers could use it to facilitate their own analyses and compare results across different study types or assumptions, enabling more informed publication and policy recommendations. For health care professionals, the application provides a straightforward way to analyze clinical trial data and obtain user-defined drug effect estimates, enhancing decision-making for patient care. Furthermore, AlzMeta.app generates PIs for each outcome within the frequentist NMA. PIs account for uncertainty (heterogeneity) in the intervention estimates and aim to predict future individual observations. Therefore, when a new patient comes to the clinic, PIs (rather than CIs) should be used to predict treatment effects and recommend the optimal treatment [80,81]. Finally, the approach presented here can be easily updated and scaled up in the future by incorporating novel treatment comparisons and other findings relevant to researchers, clinicians, and the broader community. We will continue to add more information and update AlzMeta.app annually or when a large amount of new clinical trial data and drug recommendations become available.

Several limitations of this work need to be acknowledged and further resolved. First, it relied on published trial-level data without incorporating individual patient data or longitudinal findings. Second, potential bias might be introduced in primary studies due to unblinding caused by the COVID-19 pandemic and frequent ARIAs. A recent simulation study estimated that 70%-100% of ARIAs in trials led to some degree of therapeutic insight [82]. Further adaptations in trial design and more detailed reporting and analysis of unblinding events are required [82]. Third, the patients in a donanemab phase III study [24] were stratified by their brain tau levels, which was not performed in other trials, and direct head-to-head comparisons between high-clearance antibodies have not been performed yet. Therefore, our NMA only included studies where treatments were compared with placebo. Finally, clinical experience with antiamyloid monoclonal antibodies is still in its early stages and primarily limited to clinical trial populations, which may not fully represent real-world practice in the broader symptomatic AD population. The trials performed so far lacked racial and ethnic diversity (Multimedia Appendix 3), which prevented us from determining whether the risks and benefits of these treatments may vary across different populations. Similarly, patients with abnormal MRI findings, cerebrovascular damage, major depression, schizophrenia, and other serious neuropsychiatric conditions (occurring among patients with AD) were excluded from the primary studies (Multimedia Appendix 4).

### Future Directions

Further studies should integrate real-world data collected outside the controlled settings of clinical trials and include assessments on broader and more diverse populations. This will provide more robust findings and richer insights into how these treatments perform in everyday clinical practice, especially concerning their safety and long-term outcomes in different patient populations with multiple comorbidities. As the low tolerance and significant risks associated with ARIAs and infusion-related reactions loom over the approved anti-Aβ antibodies, future research is expected to support a better understanding of ARIA etiology, prevention, and management. When ARIAs were first observed in clinical trials with anti-Aβ vaccines [83] and antibodies [22], permanent discontinuation of therapy was recommended upon ARIA detection. The current practice has evolved, and most cases of asymptomatic ARIA-E meeting specific clinical and radiographic criteria can be managed with continued treatment, as ARIA-E events can resolve spontaneously with or without temporary interruption [84]. However, moderate or more severe ARIA-E cases with symptoms like headache, dizziness, or seizures, may require therapy interruption, hospitalization, enhanced monitoring, including brain imaging and electroencephalography, and administration of corticosteroids or anticonvulsants.

Careful dose titration can help mitigate the risk of ARIA-E events, and corticosteroid treatment can reduce inflammation and the radiographic severity of ARIA-E [72,84-86]. The risks can be further minimized by excluding individuals with more than four microhemorrhages at baseline and by avoiding patients on antiplatelet or anticoagulant drugs. In any case, continuation of therapy heavily relies on accurate grading and monitoring of radiological findings. Therefore, radiologists play a crucial role in closely supervising patients for any ARIA event. Permanent cessation is recommended for 10 or more severe ARIA-H cases (even if asymptomatic, with or without ARIA-E), due to the irreversibility of ARIA-H findings [72,87,88].

ARIA events are linked to inflammation, blood-brain barrier disruption, and cerebrovascular impairment, which are already common in elderly people and patients with AD [89-91]. The ARIA risk is dose-dependent and influenced by factors such as active and passive anti-Aβ immunotherapy, anticoagulant use, *APOE*-ε4 carriership, and a history of cerebrovascular events [87,92]. The *APOE*-ε4 allele dose (homozygotes > heterozygotes) is the strongest risk factor for ARIA development, treatment discontinuation, and symptom manifestation, likely due to increased vascular Aβ burden and reduced cerebrovascular integrity [16,23,72,87,93]. With that in mind, future studies need to explore the complex interplay between ARIA and these risk factors. Mechanistic research could provide insights into the underlying biological pathways and factors that contribute to ARIA development, such as anti-Aβ immunization, baseline cerebrovascular integrity, and inflammatory responses, including complement cascade and microglia response [84,94]. Longitudinal studies should examine how these factors influence ARIA evolution in diverse patient populations to account for variability in clinical presentations and responses to therapy. Likewise, animal models that mimic ARIA-like events could help in identifying molecular mechanisms, testing interventions, and developing predictive biomarkers for ARIA. These efforts will be crucial for improving patient selection, optimizing treatment protocols, and mitigating risks in the clinical use of anti-Aβ antibodies [86]. Furthermore, these studies may aid in the development of personalized approaches that integrate vascular, neuronal, glial, and inflammatory biomarkers with multi-target therapies to improve AD treatment.

In summary, our results demonstrate the small cognitive and functional benefits and substantial biomarker effects of donanemab and lecanemab, and the superiority of donanemab for cognitive and functional outcomes. Yet, these effects were achieved with significant trade-offs in the form of high risks of treatment dropout, infusion-related reactions, cerebral vasogenic edema or sulcal effusion (ARIA-E), and microbleeding and local siderosis (ARIA-H). The risks may outweigh the benefits, highlighting the need for safer therapies and further trials to better assess the risk-benefit profile and determine patient eligibility [90]. Personalized medicine and multi-target approaches will likely play an essential role in optimizing treatment for individual patients and advancing AD treatment [95].

## Appendix

Multimedia Appendix 1 PRISMA-NMA checklist.

Multimedia Appendix 2 Supplementary methods.

Multimedia Appendix 3 Additional baseline participant characteristics.

Multimedia Appendix 4 Inclusion and exclusion criteria of primary studies (clinical trials) included in this review.

Multimedia Appendix 5 Quality of the included reports assessed by the modified Jadad scale with 9 items.

Multimedia Appendix 6 Excluded studies and reasons for exclusion.

Multimedia Appendix 7 Risk of bias.

Multimedia Appendix 8 Summary of findings tables.

Multimedia Appendix 9 Summary of findings tables for each comparison within the network meta-analysis (GRADE).

Multimedia Appendix 10 Surface under the cumulative ranking curve rank robustness.

Multimedia Appendix 11 Reported impact of the COVID-19 pandemic on the primary studies.

Multimedia Appendix 12 Influential studies and their impacts on heterogeneity.

## Abbreviations

Aβ: amyloid β
AD: Alzheimer disease
ADAS-Cog: AD Assessment Scale-Cognitive Subscale
AE: adverse event
AR: absolute risk
ARIA: amyloid-related imaging abnormality
ARIA-E: amyloid-related imaging abnormalities in the form of vasogenic cerebral edema and sulcal effusion
ARIA-H: amyloid-related imaging abnormalities in the form of microhemorrhage and superficial siderosis
CDR-SB: Clinical Dementia Rating Scale-Sum of Boxes
CSF: cerebrospinal fluid
GRADE: Grading of Recommendations, Assessment, Development, and Evaluation
MCID: minimal clinically important difference
MCMC: Markov Chain Monte Carlo
MMSE: Mini-Mental State Examination
NMA: network meta-analysis
NNT: number needed to treat
PET: positron emission tomography
PI: prediction interval
PRISMA-NMA: Preferred Reporting Items for Systematic Reviews and Meta-Analyses extension for reporting of systematic reviews with network meta-analysis
RCT: randomized placebo-controlled clinical trial
SMD: standardized mean difference
SUCRA: surface under the cumulative ranking curve

## Data Availability

All data supporting this study are available on AlzMeta.app 2.0 [44].

## Disclaimer

The conclusions drawn in this study are based on the specific datasets and methodologies employed and may not fully generalize to all clinical scenarios. The findings presented here cannot be used to supersede clinical trial data or influence the approval of any medication. Rather, this study provides an objective meta-analytic comparison (at the trial level) of different treatment options in phase II and III randomized placebo-controlled clinical trials. Further research is necessary to evaluate these treatments across broader populations and varied contexts.
