# Supplementary material for: Clinical Benefits and Risks of Antiamyloid Antibodies in Sporadic Alzheimer Disease: Systematic Review and Network Meta-Analysis With a Web Application
Source: J Med Internet Res. 2025 Apr 7;27:e68454. doi: 10.2196/68454 (PMC12012406; doi:10.2196/68454)
Supplement: Multimedia Appendix 2 [file jmir_v27i1e68454_app2.docx]

## **Multimedia Appendix 2.** Supplementary methods.

# Electronic Search Strategy (PubMed example)

The search was adapted for other databases such as Google Scholar and clinical trial registries (ClinicalTrials.gov, EU Clinical Trial Registry, ANZCTR, WHO ICTRP) using equivalent terms and syntax specific to each platform.

**Search Date:** Search conducted until September 30, 2024.

**Search Terms:** The search combined terms using Boolean operators (AND, OR) and included MeSH terms, where applicable, for increased specificity. The final search string was as follows:

("Alzheimer's Disease"[MeSH Terms] OR "Alzheimer's" OR "sporadic" OR "mild cognitive impairment") AND ("phase 2" OR "phase 3") AND ("monoclonal antibody" OR "passive immunotherapy" OR "Aducanumab" OR "BIIB037" OR "Gantenerumab" OR "Lecanemab" OR "BAN-2401" OR "Solanezumab" OR "LY2062430" OR "Crenezumab" OR "Bapineuzumab" OR "AAB-001" OR "Donanemab" OR "LY3002813")

**Limits Applied:** Publication age / Language restrictions: None.

**Inclusion Criteria:** Randomized controlled trials (RCTs) in phase II or phase III; Sample size of at least 20 participants with sporadic Alzheimer's Disease (AD); Modified Jadad scale score of ≥ 3, assessing randomization, blinding, dropouts, and other quality factors (Table S1).

**Exclusion Criteria:** Studies involving fewer than 20 participants; Non-phase II or III trials; Studies not meeting the quality threshold of the modified Jadad scale.

**Search Process:** Search terms were entered into PubMed using advanced search syntax. Filters were applied manually after retrieving the search results to exclude irrelevant studies based on inclusion/exclusion criteria.

# Table S1. The overview of study methodology

| **Quality of reports** | **Jadad scale:**   1. Was the study described as randomized? 2. Was the method of randomization appropriate? 3. Was the blinding described in the study? 4. Was the method of blinding appropriate? 5. Was there a description of withdrawals and dropouts?   **Additional assessments (modifications of Jadad scale):**   1. Was there a clear description of the inclusion and exclusion criteria? 2. Were the methods used to assess adverse effects described? 3. Were the amyloid-related imaging abnormalities (ARIAs) reported depending on the APOE genotype? 4. Were the methods of statistical analysis described? |
| --- | --- |
|  | **Risk of Bias** classified as “low”, “raising some concerns”, or “high”, by using Revised Cochrane risk-of-bias (RoB-2) tool [1, 2]. |
| **Statistical methods:** Key R-based statistical packages and softwares used | **Pair-wise Meta-Analysis**   - *esc* [3] - *meta [4]* - *metafor* [5] - *dmetar [6]* |
|  | **Frequentist Network Meta-Analysis (NMA)**   - *netmeta [7]* |
|  | **Bayesian NMA and Meta-Regression**   - *dmetar [6],* - *gemtc[8],* - *rjags* [9, 10] - JAGS 4.3.0 software [11] |
|  | **Cohen’s kappa (Rank robustness)**   - *psych [12, 13]* |
|  | **Number Needed to Treat**   - *dmetar [6, 14, 15]* for calculating Number Needed to Treat for additional Beneficial / Harmful outcome |
|  | **Correlations**   - R built-in functions |
|  | **Publication bias** statistically evaluated for continuous outcomes with more than 10 studies: [16]   - *dmetar [6]* for Egger’s regression test [17] in pair-wise NMA - *metafor* [5] for funnel plots and the Duval & Tweedie’s Trim-and-Fill procedure in pair-wise NMA - *netmeta [7]* for funnel plot and Egger’s regression [17] in frequentist NMA |

# Code for Bayesian NMA Forest plots with different prior choices for each outcome

library(rjags)

library(gemtc)

if(input$prior_heter == "Uninformative priors (let the data drive the results)") {

if(input$outcome == "Cognitive outcome: ADAS-Cog"|

input$outcome == "Amyloid Burden on PET" |

input$outcome == "Cognitive and Functional outcome: CDR-SB"|

input$outcome == "CSF p-tau-181") {

data_set <- selectedDataset_bnet()

network_dataset <- mtc.network(data.re = data_set)

model_dataset_b <- mtc.model(network_dataset,

likelihood = "normal",

link = "identity",

linearModel = "random",

n.chain = 5)

mcmc2_dataset <- mtc.run(model_dataset_b, n.adapt = 5000, n.iter = 1e5, thin = 10)

gemtc::forest(relative.effect(mcmc2_dataset, t1 = "Placebo"))

} else if(input$outcome == "Cognitive outcome: MMSE"|

input$outcome == "CSF Amyloid Beta 1-42") {

data_set <- selectedDataset_bnet()

network_dataset <- mtc.network(data.re = data_set)

model_dataset_b <- mtc.model(network_dataset,

likelihood = "normal",

link = "identity",

linearModel = "random",

n.chain = 5)

mcmc2_dataset <- mtc.run(model_dataset_b, n.adapt = 5000, n.iter = 1e5, thin = 10)

gemtc::forest(relative.effect(mcmc2_dataset, t1 = "Placebo"))

} else {

data_set <- selectedDataset_bnet()

network_dataset <- mtc.network(data.re = data_set)

model_dataset_b <- mtc.model(network_dataset,

likelihood = "binom",

link = "log",

linearModel = "random",

n.chain = 5)

mcmc2_dataset <- mtc.run(model_dataset_b, n.adapt = 5000, n.iter = 1e5, thin = 10)

gemtc::forest(relative.effect(mcmc2_dataset, t1 = "Placebo"))

}

} else if(input$prior_heter == "Empirical Half-normal priors based on overall heterogeneity measured in the frequentist NMA") {

if(input$outcome == "Cognitive outcome: ADAS-Cog") {

data_set <- selectedDataset_bnet()

network_dataset <- mtc.network(data.re = data_set)

model_dataset_b <- mtc.model(

network_dataset,

likelihood = "normal",

link = "identity",

linearModel = "random",

n.chain = 5,

hy.prior = mtc.hy.prior(type="std.dev", distr="dhnorm", 0, 0.01))

mcmc2_dataset <- mtc.run(model_dataset_b, n.adapt = 5000, n.iter = 1e5, thin = 10)

gemtc::forest(relative.effect(mcmc2_dataset, t1 = "Placebo"))

} else if(input$outcome == "Amyloid Burden on PET") {

data_set <- selectedDataset_bnet()

network_dataset <- mtc.network(data.re = data_set)

model_dataset_b <- mtc.model(

network_dataset,

likelihood = "normal",

link = "identity",

linearModel = "random",

n.chain = 5,

hy.prior = mtc.hy.prior(type="std.dev", distr="dhnorm", 0, 0.01))

mcmc2_dataset <- mtc.run(model_dataset_b, n.adapt = 5000, n.iter = 1e5, thin = 10)

gemtc::forest(relative.effect(mcmc2_dataset, t1 = "Placebo"))

} else if(input$outcome == "Cognitive and Functional outcome: CDR-SB") {

data_set <- selectedDataset_bnet()

network_dataset <- mtc.network(data.re = data_set)

model_dataset_b <- mtc.model(

network_dataset,

likelihood = "normal",

link = "identity",

linearModel = "random",

n.chain = 5,

hy.prior = mtc.hy.prior(type="std.dev", distr="dhnorm", 0, 0.01))

mcmc2_dataset <- mtc.run(model_dataset_b, n.adapt = 5000, n.iter = 1e5, thin = 10)

gemtc::forest(relative.effect(mcmc2_dataset, t1 = "Placebo"))

} else if(input$outcome == "CSF p-tau-181") {

data_set <- selectedDataset_bnet()

network_dataset <- mtc.network(data.re = data_set)

model_dataset_b <- mtc.model(

network_dataset,

likelihood = "normal",

link = "identity",

linearModel = "random",

n.chain = 5,

hy.prior = mtc.hy.prior(type="std.dev", distr="dhnorm", 0, 0.3))

mcmc2_dataset <- mtc.run(model_dataset_b, n.adapt = 5000, n.iter = 1e5, thin = 10)

gemtc::forest(relative.effect(mcmc2_dataset, t1 = "Placebo"))

} else if(input$outcome == "Cognitive outcome: MMSE") {

data_set <- selectedDataset_bnet()

network_dataset <- mtc.network(data.re = data_set)

model_dataset_b <- mtc.model(

network_dataset,

likelihood = "normal",

link = "identity",

linearModel = "random",

n.chain = 5,

hy.prior = mtc.hy.prior(type="std.dev", distr="dhnorm", 0, 0.01))

mcmc2_dataset <- mtc.run(model_dataset_b, n.adapt = 5000, n.iter = 1e5, thin = 10)

gemtc::forest(relative.effect(mcmc2_dataset, t1 = "Placebo"))

} else if(input$outcome == "CSF Amyloid Beta 1-42") {

data_set <- selectedDataset_bnet()

network_dataset <- mtc.network(data.re = data_set)

model_dataset_b <- mtc.model(

network_dataset,

likelihood = "normal",

link = "identity",

linearModel = "random",

n.chain = 5,

hy.prior = mtc.hy.prior(type="std.dev", distr="dhnorm", 0, 0.01))

mcmc2_dataset <- mtc.run(model_dataset_b, n.adapt = 5000, n.iter = 1e5, thin = 10)

gemtc::forest(relative.effect(mcmc2_dataset, t1 = "Placebo"))

} else if(input$outcome == "Risk of vasogenic cerebral edema/sulcal effusion (ARIA-E)") {

data_set <- selectedDataset_bnet()

network_dataset <- mtc.network(data.re = data_set)

model_dataset_b <- mtc.model(

network_dataset,

likelihood = "binom",

link = "log",

linearModel = "random",

n.chain = 5,

hy.prior = mtc.hy.prior(type="std.dev", distr="dhnorm", 0, 0.05))

mcmc2_dataset <- mtc.run(model_dataset_b, n.adapt = 5000, n.iter = 1e5, thin = 10)

gemtc::forest(relative.effect(mcmc2_dataset, t1 = "Placebo"))

} else if(input$outcome == "Risk of cerebral microbleeding and local siderosis (ARIA-H)") {

data_set <- selectedDataset_bnet()

network_dataset <- mtc.network(data.re = data_set)

model_dataset_b <- mtc.model(

network_dataset,

likelihood = "binom",

link = "log",

linearModel = "random",

n.chain = 5,

hy.prior = mtc.hy.prior("std.dev", "dhnorm", 0, 0.3))

mcmc2_dataset <- mtc.run(model_dataset_b, n.adapt = 5000, n.iter = 1e5, thin = 10)

gemtc::forest(relative.effect(mcmc2_dataset, t1 = "Placebo"))

} else if(input$outcome == "Treatment Discontinuation due to adverse events (AEs)") {

data_set <- selectedDataset_bnet()

network_dataset <- mtc.network(data.re = data_set)

model_dataset_b <- mtc.model(

network_dataset,

likelihood = "binom",

link = "log",

linearModel = "random",

n.chain = 5,

hy.prior = mtc.hy.prior("std.dev", "dhnorm", 0, 0.01))

mcmc2_dataset <- mtc.run(model_dataset_b, n.adapt = 5000, n.iter = 1e5, thin = 10)

gemtc::forest(relative.effect(mcmc2_dataset, t1 = "Placebo"))

} else if(input$outcome == "Serious Adverse Events") {

data_set <- selectedDataset_bnet()

network_dataset <- mtc.network(data.re = data_set)

model_dataset_b <- mtc.model(

network_dataset,

likelihood = "binom",

link = "log",

linearModel = "random",

n.chain = 5,

hy.prior = mtc.hy.prior("std.dev", "dhnorm", 0, 0.01))

mcmc2_dataset <- mtc.run(model_dataset_b, n.adapt = 5000, n.iter = 1e5, thin = 10)

gemtc::forest(relative.effect(mcmc2_dataset, t1 = "Placebo"))

} else if(input$outcome == "ARIA-E in APOE-e4 non-carriers") {

data_set <- selectedDataset_bnet()

network_dataset <- mtc.network(data.re = data_set)

model_dataset_b <- mtc.model(

network_dataset,

likelihood = "binom",

link = "log",

linearModel = "random",

n.chain = 5,

hy.prior = mtc.hy.prior("std.dev", "dhnorm", 0, 0.01))

mcmc2_dataset <- mtc.run(model_dataset_b, n.adapt = 5000, n.iter = 1e5, thin = 10)

gemtc::forest(relative.effect(mcmc2_dataset, t1 = "Placebo"))

} else if(input$outcome == "ARIA-E in APOE-e4 carriers") {

data_set <- selectedDataset_bnet()

network_dataset <- mtc.network(data.re = data_set)

model_dataset_b <- mtc.model(

network_dataset,

likelihood = "binom",

link = "log",

linearModel = "random",

n.chain = 5,

hy.prior = mtc.hy.prior("std.dev", "dhnorm", 0, 0.01))

mcmc2_dataset <- mtc.run(model_dataset_b, n.adapt = 5000, n.iter = 1e5, thin = 10)

gemtc::forest(relative.effect(mcmc2_dataset, t1 = "Placebo"))

} else if(input$outcome == "Infusion-related reactions") {

data_set <- selectedDataset_bnet()

network_dataset <- mtc.network(data.re = data_set)

model_dataset_b <- mtc.model(

network_dataset,

likelihood = "binom",

link = "log",

linearModel = "random",

n.chain = 5,

hy.prior = mtc.hy.prior("std.dev", "dhnorm", 0, 0.2))

mcmc2_dataset <- mtc.run(model_dataset_b, n.adapt = 5000, n.iter = 1e5, thin = 10)

gemtc::forest(relative.effect(mcmc2_dataset, t1 = "Placebo"))

} else if(input$outcome == "Headaches") {

data_set <- selectedDataset_bnet()

network_dataset <- mtc.network(data.re = data_set)

model_dataset_b <- mtc.model(

network_dataset,

likelihood = "binom",

link = "log",

linearModel = "random",

n.chain = 5,

hy.prior = mtc.hy.prior("std.dev", "dhnorm", 0, 0.01))

mcmc2_dataset <- mtc.run(model_dataset_b, n.adapt = 5000, n.iter = 1e5, thin = 10)

gemtc::forest(relative.effect(mcmc2_dataset, t1 = "Placebo"))

} else if(input$outcome == "Nausea") {

data_set <- selectedDataset_bnet()

network_dataset <- mtc.network(data.re = data_set)

model_dataset_b <- mtc.model(

network_dataset,

likelihood = "binom",

link = "log",

linearModel = "random",

n.chain = 5,

hy.prior = mtc.hy.prior("std.dev", "dhnorm", 0, 0.01))

mcmc2_dataset <- mtc.run(model_dataset_b, n.adapt = 5000, n.iter = 1e5, thin = 10)

gemtc::forest(relative.effect(mcmc2_dataset, t1 = "Placebo"))

} else if(input$outcome == "Dizziness") {

data_set <- selectedDataset_bnet()

network_dataset <- mtc.network(data.re = data_set)

model_dataset_b <- mtc.model(

network_dataset,

likelihood = "binom",

link = "log",

linearModel = "random",

n.chain = 5,

hy.prior = mtc.hy.prior("std.dev", "dhnorm", 0, 0.01))

mcmc2_dataset <- mtc.run(model_dataset_b, n.adapt = 5000, n.iter = 1e5, thin = 10)

gemtc::forest(relative.effect(mcmc2_dataset, t1 = "Placebo"))

} else if(input$outcome == "Fall") {

data_set <- selectedDataset_bnet()

network_dataset <- mtc.network(data.re = data_set)

model_dataset_b <- mtc.model(

network_dataset,

likelihood = "binom",

link = "log",

linearModel = "random",

n.chain = 5,

hy.prior = mtc.hy.prior("std.dev", "dhnorm", 0, 0.01))

mcmc2_dataset <- mtc.run(model_dataset_b, n.adapt = 5000, n.iter = 1e5, thin = 10)

gemtc::forest(relative.effect(mcmc2_dataset, t1 = "Placebo"))

} else if(input$outcome == "Diarrhea") {

data_set <- selectedDataset_bnet()

network_dataset <- mtc.network(data.re = data_set)

model_dataset_b <- mtc.model(

network_dataset,

likelihood = "binom",

link = "log",

linearModel = "random",

n.chain = 5,

hy.prior = mtc.hy.prior("std.dev", "dhnorm", 0, 0.01))

mcmc2_dataset <- mtc.run(model_dataset_b, n.adapt = 5000, n.iter = 1e5, thin = 10)

gemtc::forest(relative.effect(mcmc2_dataset, t1 = "Placebo"))

} else if(input$outcome == "Arthralgia") {

data_set <- selectedDataset_bnet()

network_dataset <- mtc.network(data.re = data_set)

model_dataset_b <- mtc.model(

network_dataset,

likelihood = "binom",

link = "log",

linearModel = "random",

n.chain = 5,

hy.prior = mtc.hy.prior("std.dev", "dhnorm", 0, 0.15))

mcmc2_dataset <- mtc.run(model_dataset_b, n.adapt = 5000, n.iter = 1e5, thin = 10)

gemtc::forest(relative.effect(mcmc2_dataset, t1 = "Placebo"))

} else if(input$outcome == "Back Pain") {

data_set <- selectedDataset_bnet()

network_dataset <- mtc.network(data.re = data_set)

model_dataset_b <- mtc.model(

network_dataset,

likelihood = "binom",

link = "log",

linearModel = "random",

n.chain = 5,

hy.prior = mtc.hy.prior("std.dev", "dhnorm", 0, 0.01))

mcmc2_dataset <- mtc.run(model_dataset_b, n.adapt = 5000, n.iter = 1e5, thin = 10)

gemtc::forest(relative.effect(mcmc2_dataset, t1 = "Placebo"))

} else if(input$outcome == "Fatigue") {

data_set <- selectedDataset_bnet()

network_dataset <- mtc.network(data.re = data_set)

if(input$heterog_exc3 == "TRUE") {

model_dataset_b <- mtc.model(

network_dataset,

likelihood = "binom",

link = "log",

linearModel = "random",

n.chain = 5,

hy.prior = mtc.hy.prior("std.dev", "dhnorm", 0, 0.01))

} else if(input$heterog_exc3 == "FALSE") {

model_dataset_b <- mtc.model(

network_dataset,

likelihood = "binom",

link = "log",

linearModel = "random",

n.chain = 5,

hy.prior = mtc.hy.prior("std.dev", "dhnorm", 0, 0.01))

}

mcmc2_dataset <- mtc.run(model_dataset_b, n.adapt = 5000, n.iter = 1e5, thin = 10)

gemtc::forest(relative.effect(mcmc2_dataset, t1 = "Placebo"))

} else if(input$outcome == "Upper respiratory infections") {

data_set <- selectedDataset_bnet()

network_dataset <- mtc.network(data.re = data_set)

model_dataset_b <- mtc.model(

network_dataset,

likelihood = "binom",

link = "log",

linearModel = "random",

n.chain = 5,

hy.prior = mtc.hy.prior("std.dev", "dhnorm", 0, 0.01))

mcmc2_dataset <- mtc.run(model_dataset_b, n.adapt = 5000, n.iter = 1e5, thin = 10)

gemtc::forest(relative.effect(mcmc2_dataset, t1 = "Placebo"))

} else if(input$outcome == "Nasopharyngitis") {

data_set <- selectedDataset_bnet()

network_dataset <- mtc.network(data.re = data_set)

model_dataset_b <- mtc.model(

network_dataset,

likelihood = "binom",

link = "log",

linearModel = "random",

n.chain = 5,

hy.prior = mtc.hy.prior("std.dev", "dhnorm", 0, 0.02))

mcmc2_dataset <- mtc.run(model_dataset_b, n.adapt = 5000, n.iter = 1e5, thin = 10)

gemtc::forest(relative.effect(mcmc2_dataset, t1 = "Placebo"))

} else if(input$outcome == "Urinary infections") {

data_set <- selectedDataset_bnet()

network_dataset <- mtc.network(data.re = data_set)

model_dataset_b <- mtc.model(

network_dataset,

likelihood = "binom",

link = "log",

linearModel = "random",

n.chain = 5,

hy.prior = mtc.hy.prior("std.dev", "dhnorm", 0, 0.015))

mcmc2_dataset <- mtc.run(model_dataset_b, n.adapt = 5000, n.iter = 1e5, thin = 10)

gemtc::forest(relative.effect(mcmc2_dataset, t1 = "Placebo"))

}

} else if(input$prior_heter == "Half-normal priors assuming 3x greater heterogeneity than measured") {

if(input$outcome == "Cognitive outcome: ADAS-Cog") {

data_set <- selectedDataset_bnet()

network_dataset <- mtc.network(data.re = data_set)

model_dataset_b <- mtc.model(

network_dataset,

likelihood = "normal",

link = "identity",

linearModel = "random",

n.chain = 5,

hy.prior = mtc.hy.prior(type="std.dev", distr="dhnorm", 0, 0.01*3))

mcmc2_dataset <- mtc.run(model_dataset_b, n.adapt = 5000, n.iter = 1e5, thin = 10)

gemtc::forest(relative.effect(mcmc2_dataset, t1 = "Placebo"))

} else if(input$outcome == "Amyloid Burden on PET") {

data_set <- selectedDataset_bnet()

network_dataset <- mtc.network(data.re = data_set)

model_dataset_b <- mtc.model(

network_dataset,

likelihood = "normal",

link = "identity",

linearModel = "random",

n.chain = 5,

hy.prior = mtc.hy.prior(type="std.dev", distr="dhnorm", 0, 0.01*3))

mcmc2_dataset <- mtc.run(model_dataset_b, n.adapt = 5000, n.iter = 1e5, thin = 10)

gemtc::forest(relative.effect(mcmc2_dataset, t1 = "Placebo"))

} else if(input$outcome == "Cognitive and Functional outcome: CDR-SB") {

data_set <- selectedDataset_bnet()

network_dataset <- mtc.network(data.re = data_set)

model_dataset_b <- mtc.model(

network_dataset,

likelihood = "normal",

link = "identity",

linearModel = "random",

n.chain = 5,

hy.prior = mtc.hy.prior(type="std.dev", distr="dhnorm", 0, 0.01*3))

mcmc2_dataset <- mtc.run(model_dataset_b, n.adapt = 5000, n.iter = 1e5, thin = 10)

gemtc::forest(relative.effect(mcmc2_dataset, t1 = "Placebo"))

} else if(input$outcome == "CSF p-tau-181") {

data_set <- selectedDataset_bnet()

network_dataset <- mtc.network(data.re = data_set)

model_dataset_b <- mtc.model(

network_dataset,

likelihood = "normal",

link = "identity",

linearModel = "random",

n.chain = 5,

hy.prior = mtc.hy.prior(type="std.dev", distr="dhnorm", 0, 0.3*3))

mcmc2_dataset <- mtc.run(model_dataset_b, n.adapt = 5000, n.iter = 1e5, thin = 10)

gemtc::forest(relative.effect(mcmc2_dataset, t1 = "Placebo"))

} else if(input$outcome == "Cognitive outcome: MMSE") {

data_set <- selectedDataset_bnet()

network_dataset <- mtc.network(data.re = data_set)

model_dataset_b <- mtc.model(

network_dataset,

likelihood = "normal",

link = "identity",

linearModel = "random",

n.chain = 5,

hy.prior = mtc.hy.prior(type="std.dev", distr="dhnorm", 0, 0.01*3))

mcmc2_dataset <- mtc.run(model_dataset_b, n.adapt = 5000, n.iter = 1e5, thin = 10)

gemtc::forest(relative.effect(mcmc2_dataset, t1 = "Placebo"))

} else if(input$outcome == "CSF Amyloid Beta 1-42") {

data_set <- selectedDataset_bnet()

network_dataset <- mtc.network(data.re = data_set)

model_dataset_b <- mtc.model(

network_dataset,

likelihood = "normal",

link = "identity",

linearModel = "random",

n.chain = 5,

hy.prior = mtc.hy.prior(type="std.dev", distr="dhnorm", 0, 0.01*3))

mcmc2_dataset <- mtc.run(model_dataset_b, n.adapt = 5000, n.iter = 1e5, thin = 10)

gemtc::forest(relative.effect(mcmc2_dataset, t1 = "Placebo"))

} else if(input$outcome == "Risk of vasogenic cerebral edema/sulcal effusion (ARIA-E)") {

data_set <- selectedDataset_bnet()

network_dataset <- mtc.network(data.re = data_set)

model_dataset_b <- mtc.model(

network_dataset,

likelihood = "binom",

link = "log",

linearModel = "random",

n.chain = 5,

hy.prior = mtc.hy.prior(type="std.dev", distr="dhnorm", 0, 0.05*3))

mcmc2_dataset <- mtc.run(model_dataset_b, n.adapt = 5000, n.iter = 1e5, thin = 10)

gemtc::forest(relative.effect(mcmc2_dataset, t1 = "Placebo"))

} else if(input$outcome == "Risk of cerebral microbleeding and local siderosis (ARIA-H)") {

data_set <- selectedDataset_bnet()

network_dataset <- mtc.network(data.re = data_set)

model_dataset_b <- mtc.model(

network_dataset,

likelihood = "binom",

link = "log",

linearModel = "random",

n.chain = 5,

hy.prior = mtc.hy.prior("std.dev", "dhnorm", 0, 0.3*3))

mcmc2_dataset <- mtc.run(model_dataset_b, n.adapt = 5000, n.iter = 1e5, thin = 10)

gemtc::forest(relative.effect(mcmc2_dataset, t1 = "Placebo"))

} else if(input$outcome == "Treatment Discontinuation due to adverse events (AEs)"){

data_set <- selectedDataset_bnet()

network_dataset <- mtc.network(data.re = data_set)

model_dataset_b <- mtc.model(

network_dataset,

likelihood = "binom",

link = "log",

linearModel = "random",

n.chain = 5,

hy.prior = mtc.hy.prior("std.dev", "dhnorm", 0, 0.01*3))

mcmc2_dataset <- mtc.run(model_dataset_b, n.adapt = 5000, n.iter = 1e5, thin = 10)

gemtc::forest(relative.effect(mcmc2_dataset, t1 = "Placebo"))

} else if(input$outcome == "Serious Adverse Events") {

data_set <- selectedDataset_bnet()

network_dataset <- mtc.network(data.re = data_set)

model_dataset_b <- mtc.model(

network_dataset,

likelihood = "binom",

link = "log",

linearModel = "random",

n.chain = 5,

hy.prior = mtc.hy.prior("std.dev", "dhnorm", 0, 0.01*3))

mcmc2_dataset <- mtc.run(model_dataset_b, n.adapt = 5000, n.iter = 1e5, thin = 10)

gemtc::forest(relative.effect(mcmc2_dataset, t1 = "Placebo"))

} else if(input$outcome == "ARIA-E in APOE-e4 non-carriers") {

data_set <- selectedDataset_bnet()

network_dataset <- mtc.network(data.re = data_set)

model_dataset_b <- mtc.model(

network_dataset,

likelihood = "binom",

link = "log",

linearModel = "random",

n.chain = 5,

hy.prior = mtc.hy.prior("std.dev", "dhnorm", 0, 0.01*3))

mcmc2_dataset <- mtc.run(model_dataset_b, n.adapt = 5000, n.iter = 1e5, thin = 10)

gemtc::forest(relative.effect(mcmc2_dataset, t1 = "Placebo"))

} else if(input$outcome == "ARIA-E in APOE-e4 carriers") {

data_set <- selectedDataset_bnet()

network_dataset <- mtc.network(data.re = data_set)

model_dataset_b <- mtc.model(

network_dataset,

likelihood = "binom",

link = "log",

linearModel = "random",

n.chain = 5,

hy.prior = mtc.hy.prior("std.dev", "dhnorm", 0, 0.01*3))

mcmc2_dataset <- mtc.run(model_dataset_b, n.adapt = 5000, n.iter = 1e5, thin = 10)

gemtc::forest(relative.effect(mcmc2_dataset, t1 = "Placebo"))

} else if(input$outcome == "Infusion-related reactions") {

data_set <- selectedDataset_bnet()

network_dataset <- mtc.network(data.re = data_set)

model_dataset_b <- mtc.model(

network_dataset,

likelihood = "binom",

link = "log",

linearModel = "random",

n.chain = 5,

hy.prior = mtc.hy.prior("std.dev", "dhnorm", 0, 0.2*3))

mcmc2_dataset <- mtc.run(model_dataset_b, n.adapt = 5000, n.iter = 1e5, thin = 10)

gemtc::forest(relative.effect(mcmc2_dataset, t1 = "Placebo"))

} else if(input$outcome == "Headaches") {

data_set <- selectedDataset_bnet()

network_dataset <- mtc.network(data.re = data_set)

model_dataset_b <- mtc.model(

network_dataset,

likelihood = "binom",

link = "log",

linearModel = "random",

n.chain = 5,

hy.prior = mtc.hy.prior("std.dev", "dhnorm", 0, 0.01*3))

mcmc2_dataset <- mtc.run(model_dataset_b, n.adapt = 5000, n.iter = 1e5, thin = 10)

gemtc::forest(relative.effect(mcmc2_dataset, t1 = "Placebo"))

} else if(input$outcome == "Nausea") {

data_set <- selectedDataset_bnet()

network_dataset <- mtc.network(data.re = data_set)

model_dataset_b <- mtc.model(

network_dataset,

likelihood = "binom",

link = "log",

linearModel = "random",

n.chain = 5,

hy.prior = mtc.hy.prior("std.dev", "dhnorm", 0, 0.01*3))

mcmc2_dataset <- mtc.run(model_dataset_b, n.adapt = 5000, n.iter = 1e5, thin = 10)

gemtc::forest(relative.effect(mcmc2_dataset, t1 = "Placebo"))

} else if(input$outcome == "Dizziness") {

data_set <- selectedDataset_bnet()

network_dataset <- mtc.network(data.re = data_set)

model_dataset_b <- mtc.model(

network_dataset,

likelihood = "binom",

link = "log",

linearModel = "random",

n.chain = 5,

hy.prior = mtc.hy.prior("std.dev", "dhnorm", 0, 0.01*3))

mcmc2_dataset <- mtc.run(model_dataset_b, n.adapt = 5000, n.iter = 1e5, thin = 10)

gemtc::forest(relative.effect(mcmc2_dataset, t1 = "Placebo"))

} else if(input$outcome == "Fall") {

data_set <- selectedDataset_bnet()

network_dataset <- mtc.network(data.re = data_set)

model_dataset_b <- mtc.model(

network_dataset,

likelihood = "binom",

link = "log",

linearModel = "random",

n.chain = 5,

hy.prior = mtc.hy.prior("std.dev", "dhnorm", 0, 0.01*3))

mcmc2_dataset <- mtc.run(model_dataset_b, n.adapt = 5000, n.iter = 1e5, thin = 10)

gemtc::forest(relative.effect(mcmc2_dataset, t1 = "Placebo"))

} else if(input$outcome == "Diarrhea") {

data_set <- selectedDataset_bnet()

network_dataset <- mtc.network(data.re = data_set)

model_dataset_b <- mtc.model(

network_dataset,

likelihood = "binom",

link = "log",

linearModel = "random",

n.chain = 5,

hy.prior = mtc.hy.prior("std.dev", "dhnorm", 0, 0.01*3))

mcmc2_dataset <- mtc.run(model_dataset_b, n.adapt = 5000, n.iter = 1e5, thin = 10)

gemtc::forest(relative.effect(mcmc2_dataset, t1 = "Placebo"))

} else if(input$outcome == "Arthralgia") {

data_set <- selectedDataset_bnet()

network_dataset <- mtc.network(data.re = data_set)

model_dataset_b <- mtc.model(

network_dataset,

likelihood = "binom",

link = "log",

linearModel = "random",

n.chain = 5,

hy.prior = mtc.hy.prior("std.dev", "dhnorm", 0, 0.15*3))

mcmc2_dataset <- mtc.run(model_dataset_b, n.adapt = 5000, n.iter = 1e5, thin = 10)

gemtc::forest(relative.effect(mcmc2_dataset, t1 = "Placebo"))

} else if(input$outcome == "Back Pain") {

data_set <- selectedDataset_bnet()

network_dataset <- mtc.network(data.re = data_set)

model_dataset_b <- mtc.model(

network_dataset,

likelihood = "binom",

link = "log",

linearModel = "random",

n.chain = 5,

hy.prior = mtc.hy.prior("std.dev", "dhnorm", 0, 0.01*3))

mcmc2_dataset <- mtc.run(model_dataset_b, n.adapt = 5000, n.iter = 1e5, thin = 10)

gemtc::forest(relative.effect(mcmc2_dataset, t1 = "Placebo"))

} else if(input$outcome == "Fatigue") {

data_set <- selectedDataset_bnet()

network_dataset <- mtc.network(data.re = data_set)

if(input$heterog_exc3 == "TRUE") {

model_dataset_b <- mtc.model(

network_dataset,

likelihood = "binom",

link = "log",

linearModel = "random",

n.chain = 5,

hy.prior = mtc.hy.prior("std.dev", "dhnorm", 0, 0.01*3))

} else if(input$heterog_exc3 == "FALSE") {

model_dataset_b <- mtc.model(

network_dataset,

likelihood = "binom",

link = "log",

linearModel = "random",

n.chain = 5,

hy.prior = mtc.hy.prior("std.dev", "dhnorm", 0, 0.01*3))

}

mcmc2_dataset <- mtc.run(model_dataset_b, n.adapt = 5000, n.iter = 1e5, thin = 10)

gemtc::forest(relative.effect(mcmc2_dataset, t1 = "Placebo"))

} else if(input$outcome == "Upper respiratory infections") {

data_set <- selectedDataset_bnet()

network_dataset <- mtc.network(data.re = data_set)

model_dataset_b <- mtc.model(

network_dataset,

likelihood = "binom",

link = "log",

linearModel = "random",

n.chain = 5,

hy.prior = mtc.hy.prior("std.dev", "dhnorm", 0, 0.01*3))

mcmc2_dataset <- mtc.run(model_dataset_b, n.adapt = 5000, n.iter = 1e5, thin = 10)

gemtc::forest(relative.effect(mcmc2_dataset, t1 = "Placebo"))

} else if(input$outcome == "Nasopharyngitis") {

data_set <- selectedDataset_bnet()

network_dataset <- mtc.network(data.re = data_set)

model_dataset_b <- mtc.model(

network_dataset,

likelihood = "binom",

link = "log",

linearModel = "random",

n.chain = 5,

hy.prior = mtc.hy.prior("std.dev", "dhnorm", 0, 0.02*3))

mcmc2_dataset <- mtc.run(model_dataset_b, n.adapt = 5000, n.iter = 1e5, thin = 10)

gemtc::forest(relative.effect(mcmc2_dataset, t1 = "Placebo"))

} else if(input$outcome == "Urinary infections") {

data_set <- selectedDataset_bnet()

network_dataset <- mtc.network(data.re = data_set)

model_dataset_b <- mtc.model(

network_dataset,

likelihood = "binom",

link = "log",

linearModel = "random",

n.chain = 5,

hy.prior = mtc.hy.prior("std.dev", "dhnorm", 0, 0.015*3))

mcmc2_dataset <- mtc.run(model_dataset_b, n.adapt = 5000, n.iter = 1e5, thin = 10)

gemtc::forest(relative.effect(mcmc2_dataset, t1 = "Placebo"))

}

}

## REFERENCES

1. Sterne JAC, Savović J, Page MJ, Elbers RG, Blencowe NS, Boutron I, et al. RoB 2: a revised tool for assessing risk of bias in randomised trials. Bmj. 2019 Aug 28;366:l4898. PMID: 31462531. doi: 10.1136/bmj.l4898.

2. Higgins JP, Savović J, Page MJ, Elbers RG, Sterne JA. Assessing risk of bias in a randomized trial. Cochrane handbook for systematic reviews of interventions. 2019:205-28.

3. Lüdecke D, Lüdecke MD, Calculator'from David BW. Package ‘esc’. R Package Version 05. 2019;1:2019.

4. Schwarzer G. meta: An R package for meta-analysis. R news. 2007;7(3):40-5.

5. Viechtbauer W, Viechtbauer MW. Package ‘metafor’. The Comprehensive R Archive Network Package ‘metafor’ http://cran r-project org/web/packages/metafor/metafor pdf. 2015.

6. Harrer M, Cuijpers P, Furukawa T, Ebert DD. dmetar: companion R package for the guide'doing meta-analysis in R'. R package version 00. 2019;9000:2019.

7. Balduzzi S, Rücker G, Nikolakopoulou A, Papakonstantinou T, Salanti G, Efthimiou O, et al. netmeta: an R package for network meta-analysis using frequentist methods. Journal of Statistical Software. 2023;106:1-40.

8. van Valkenhoef G, Kuiper J, van Valkenhoef MG. Package ‘gemtc’. 2016.

9. Harrer M, Cuijpers P, Furukawa T, Ebert D. Doing meta-analysis with R: A hands-on guide: Chapman and Hall/CRC; 2021. ISBN: 1003107346.

10. van Valkenhoef G, Lu G, de Brock B, Hillege H, Ades AE, Welton NJ. Automating network meta-analysis. Res Synth Methods. 2012 Dec;3(4):285-99. PMID: 26053422. doi: 10.1002/jrsm.1054.

11. Plummer M, editor. JAGS: A program for analysis of Bayesian graphical models using Gibbs sampling. Proceedings of the 3rd international workshop on distributed statistical computing; 2003: Vienna, Austria.

12. Revelle W. An introduction to the psych package: Part I: data entry and data description. Northwestern University. 2019.

13. Revelle W, Revelle MW. Package ‘psych’. The comprehensive R archive network. 2015;337(338):161-5.

14. Furukawa TA. From effect size into number needed to treat. Lancet. 1999 May 15;353(9165):1680. PMID: 10335798. doi: 10.1016/s0140-6736(99)01163-0.

15. Furukawa TA, Leucht S. How to obtain NNT from Cohen's d: comparison of two methods. PLoS One. 2011 Apr 27;6(4):e19070. PMID: 21556361. doi: 10.1371/journal.pone.0019070.

16. Sterne JA, Sutton AJ, Ioannidis JP, Terrin N, Jones DR, Lau J, et al. Recommendations for examining and interpreting funnel plot asymmetry in meta-analyses of randomised controlled trials. Bmj. 2011 Jul 22;343:d4002. PMID: 21784880. doi: 10.1136/bmj.d4002.

17. Egger M, Davey Smith G, Schneider M, Minder C. Bias in meta-analysis detected by a simple, graphical test. Bmj. 1997 Sep 13;315(7109):629-34. PMID: 9310563. doi: 10.1136/bmj.315.7109.629.
