# Supplementary material for: Clinical Benefits and Risks of Antiamyloid Antibodies in Sporadic Alzheimer Disease: Systematic Review and Network Meta-Analysis With a Web Application
Source: J Med Internet Res. 2025 Apr 7;27:e68454. doi: 10.2196/68454 (PMC12012406; doi:10.2196/68454)
Supplement: Multimedia Appendix 3 [file jmir_v27i1e68454_app3.docx]

### **Multimedia Appendix 3.** Additional baseline participant characteristics.

The comparisons with “vs.” refer to comparisons between Treatment and Placebo group. **Abbreviations**: ADAS-Cog - Alzheimer's Disease Assessment Scale-Cognitive Subscale; APOE – apolipoprotein E; CDR-SB - Clinical Dementia Rating Scale–Sum of Boxes; SD – standard deviations

| **Study Name (Year) Dose** | **Study Sites (No.)** | **% of females** | **Mean age in years (SD)** | **ADAS-Cog** | **ADAS-Cog version** | **CDR-SB** | **% of APOE-ε_4_ carriers** | **% of white patients** | **% of black patients** | **% of Asian patients** | **% of Hispanic / Latino patients** |
| --- | --- | --- | --- | --- | --- | --- | --- | --- | --- | --- | --- |
| Salloway et al [1] (2009) high | 30 | 53.9 | 67.9 (0.85) vs. 70.1 (0.82) | NR | 12 | NR | 69.8 vs. 60.5 | 95.3 vs. 96.7 | NR | NR | NR |
| Salloway et al [2] 1 (2014) Study 301 low | 186 | 50.3 | 71.9 (10.1) vs. 73.1 (9.3) | 22.4 (9.7) vs. 22.2 (10.1) | 11 | NR | 0 vs. 0 | 95.1 vs. 94.9 | NR | NR | NR |
| Salloway et al [2] 2 (2014) Study 301 high | 186 | 50.3 | 71.9 (10.1) vs. 73.5 (9.1) | 22.2 (10.0) vs. 22.2 (10.1) | 11 | NR | 0 vs. 0 | 95.1 vs. 95.1 | NR | NR | NR |
| Salloway et al [2] 3 (2014) Study 302 low | 251 | 56 | 72.3 (8.4) vs. 72 (8.0) | 23.5 (9.4) vs. 23.9 (9.5) | 11 | NR | 100 vs. 100 | 97.2 vs. 94.8 | NR | NR | NR |
| Doody et al [3] 1 (2014) EXPEDITION 1 | 85 | 56.7 | 74.4 (8.0) vs. 75 (7.9) | 22.0 (8.0) vs. 22.0 (9.0) | 11 | NR | 61.3 vs. 57.3 | 84.4 vs. 83 | 4.9 vs. 4 | 9.7 vs. 12.8 | NR |
| Doody et al [3] 2 (2014) EXPEDITION 2 | 95 | 55.1 | 72.4 (7.8) vs. 72.5 (8.0) | 24.0 (9.0) vs. 23.0 (10.0) | 11 | NR | 59.5 vs. 56.8 | 77.6 vs. 76.6 | 0.4 vs. 1.3 | 22 vs .21.5 | NR |
| Vandenberghe et al [4] 1 (2016) low | 348 | 57.8 | 69.9 (9.76) vs. 71.4 (9.38) | 23.2 (10.0) vs. 22.9 (10.2) | 11 | NR | 0 vs. 0 | 80.5 vs. 79.2 | 0.6 vs. 0.8 | 17.1 vs. 17.3 | NR |
| Vandenberghe et al [4] 2 (2016) high | 348 | 57.8 | 69.9 (9.76) vs. 70.8 (9.73) | 23.5 (9.3) vs. 22.9 (10.2) | 11 | NR | 0 vs. 0 | 80.5 vs. 79.4 | 0.6 vs. 2 | 17.1 vs. 17.4 | NR |
| Vandenberghe et al [4] 3 (2016) low | 287 | 59.7 | 70.3 (7.75) vs. 71 (7.67) | 23.2 (8.9) vs. 22.6 (8.9) | 11 | NR | 100 vs. 100 | 82.6 vs. 79.5 | 0.7 vs. 0.8 | 16 vs. 17.7 | NR |
| Honig et al [5] (2018) EXPEDITION 3 | 198 | 56.76 | 73.3 (7.97) vs. 72.7 (7.81) | 28.9 (8.3) vs. 29.7 (8.5) | 14 | 3.9 (1.9) vs. 3.9 (2.0) | 66.3 vs. 69.3 | 90.5 vs. 90.7 | 1.4 vs. 1.9 | 7.7 vs. 7.2 | 5.1 vs. 5 |
| Haeberlein et al [6] (2022) EMERGE low | 180 | 52.9 | 70.8 (7.4) vs. 70.6 (7.45) | 22.5 (6.76) vs. 21.9 (6.73) | 13 | 2.46 (1.01) vs. 2.47 (1) | 67 vs. 66.7 | 78.6 vs. 79.6 | 0.2 vs. 1.1 | 8.6 vs. 7.2 | 4 vs. 4.1 |
| Haeberlein et al [6] (2022) EMERGE high | 180 | 52.9 | 70.8 (7.4) vs. 70.6 (7.47) | 22.2 (7.08) vs. 21.9 (6.73) | 13 | 2.51 (1.05) vs. 2.47 (1) | 67.1 vs. 66.7 | 78.6 vs. 77.1 | 0.2 vs. 0.7 | 8.6 vs. 7.7 | 4 vs. 4.2 |
| Haeberlein et al [6] (2022) ENGAGE low | 181 | 52.7 | 69.8 (7.72) vs. 70.4 (6.96) | 22.5 (6.30) vs. 22.5 (6.56) | 13 | 2.43 (1.01) vs. 2.40 (1) | 69.8 vs. 70.4 | 75.8 vs. 75.3 | 0.9 vs. 0.2 | 10.1 vs. 10.1 | 2.4 vs. 2 |
| Haeberlein et al [6] (2022) ENGAGE high | 181 | 52.7 | 69.8 (7.72) vs. 70 (7.65) | 22.4 (6.54) vs. 22.5 (6.56) | 13 | 2.40 (1.01) vs. 2.40 (1) | 69 vs. 68.1 | 75.8 vs. 74.4 | 0.9 vs. 0.4 | 10.1 vs. 11.7 | 2.4 vs. 2 |
| van Dyck et al [7] (2023) Clarity AD | 247 | 53 | 71 (7.8) vs. 71.4 (7.9) | 24.45 (7.08) vs. 24.37 (7.56) | 14 | 3.17 (1.34) vs. 3.22 (1.3) | 68.6 vs. 68.9 | 77.4 vs. 76.3 | 2.7 vs. 2.3 | 16.9 vs. 17.1 | NR |
| Swanson et al [8] 1 (2021) high | 117 | 50 | 72 (6.5) vs. 73 (6.17) | 22.1 (7.7) vs. 22.6 (7.7) | 14 | 3.0 (1.4) vs. 2.9 (1.5) | 70.85 vs. 29.81 | NR | NR | NR | NR |
| Swanson et al [8] 2 (2021) low | 117 | 51.5 | 72 (6.5) vs. 71 (6.17) | 21.9 (7.3) vs. 22.6 (7.7) | 14 | 2.9 (1.3) vs. 2.9 (1.5) | 70.85 vs. 88.93 | NR | NR | NR | NR |
| Sims et al [9] (2023) TRAILBLAZER-ALZ 2 | 277 | 57.37 | 73 (6.2) vs. 73 (6.2) | 29.3 (8.9) vs. 28.7 (8.8) | 13 | 4.0 (2.1) vs. 3.9 (2.1) | 68.3 vs. 67.2 | 96.4 vs. 94.7 | 3.1 vs. 4.1 | 0.5 vs. 1 | 6.3 vs. 5.8 |
| Mintun et al [10] (2021) TRAILBLAZER-ALZ | 277 | 51.75 | 75.4 (5.4) vs. 75 (5.6) | 27.6 (7.7) vs. 27.5 (7.6) | 13 | 3.6 (2.1) vs. 3.4 (1.7) | 74.2 vs. 72.5 | 95.5 vs. 94.7 | 2.5 vs. 2.9 | 0.5 vs. 1.3 | 5.7 vs. 5.7 |
| Bateman et al [11] (2023) GRADUATE I | 156 | 55.35 | 72.1 (7.8) vs. 71.1 (7.9) | 28.1 (7.1) vs. 28.1 (6.8) | 13 | 3.71 (1.67) vs. 71 (1.6) | 67.6 vs. 65.3 | 82.1 vs. 83 | 1.2 vs. 0.2 | 10.9 vs. 1.4 | 12 vs. 10.4 |
| Bateman et al [11] (2023) GRADUATE II | 152 | 58.75 | 71.8 (7.4) vs. 71.6 (7.8) | 28.1 (6.9) vs. 28.2 (7.0) | 13 | 3.67 (1.61) vs. 3.52 (1.5) | 67.3 vs. 66.9 | 80.7 vs. 85.1 | 0.8 vs. 1 | 15.7 vs. 11.2 | 24.9 vs. 22.5 |
| Ostrowitzki et al [12] (2022) CREAD | 194 | 57.6 | 70.3 (8.4) vs. 71 (7.9) | 28.9 (7.4) vs. 29.4 (7.6) | 13 | 3.79 (1.6) vs. 3.88 (1.7) | 71.7 vs. 72.7 | 88 vs. 87.1 | 0.7 vs. 1.2 | 6.8 vs. 6.9 | NR |
| Salloway et al [13] (2018) BLAZE (pooled) | 23 | 54.8 | 69.8 (7.7) vs. 71.4 (7.1) | 31.2 (9.9) vs. 34.5 (11.1) | 12 | 4.9 (2.0) vs. 5.9 (1.9) | 70.6 vs. 68.6 | NR | NR | NR | NR |
| Ostrowitzki et al [14] (2017) SCarlet RoAD I | 139 | 56.1 | 69.5 (7.5) vs. 70.3 (7.0) | 23.5 (7.2) vs. 23.1 (6.9) | 11 | 2.1 (1.0) vs. 2.2 (1.0) | 12.9 vs. 13.6 | 89.85 vs. 93 | 0.38 vs. 0.74 | 3.38 vs. 0.74 | 15.41 vs. 14.39 |
| Ostrowitzki et al [14] (2017) SCarlet RoAD II | 139 | 58.5 | 69.5 (7.5) 71.3 (7.1) | 23.5 (7.2) vs. 23.0 (6.2) | 11 | 2.1 (1.0) vs. 2.0 (0.9) | 12.9 vs. 12.5 | 89.85 vs. 91.9 | 0.38 vs. 0.77 | 3.38 vs. 0.77 | 15.41 vs. 18.08 |

**References**

1. Salloway S, Sperling R, Gilman S, Fox NC, Blennow K, Raskind M, et al. A phase 2 multiple ascending dose trial of bapineuzumab in mild to moderate Alzheimer disease. Neurology. 2009 Dec 15;73(24):2061-70. PMID: 19923550. doi: 10.1212/WNL.0b013e3181c67808.

2. Salloway S, Sperling R, Fox NC, Blennow K, Klunk W, Raskind M, et al. Two phase 3 trials of bapineuzumab in mild-to-moderate Alzheimer's disease. N Engl J Med. 2014 Jan 23;370(4):322-33. PMID: 24450891. doi: 10.1056/NEJMoa1304839.

3. Doody RS, Thomas RG, Farlow M, Iwatsubo T, Vellas B, Joffe S, et al. Phase 3 trials of solanezumab for mild-to-moderate Alzheimer's disease. N Engl J Med. 2014 Jan 23;370(4):311-21. PMID: 24450890. doi: 10.1056/NEJMoa1312889.

4. Vandenberghe R, Rinne JO, Boada M, Katayama S, Scheltens P, Vellas B, et al. Bapineuzumab for mild to moderate Alzheimer's disease in two global, randomized, phase 3 trials. Alzheimers Res Ther. 2016 May 12;8(1):18. PMID: 27176461. doi: 10.1186/s13195-016-0189-7.

5. Honig LS, Vellas B, Woodward M, Boada M, Bullock R, Borrie M, et al. Trial of Solanezumab for Mild Dementia Due to Alzheimer's Disease. N Engl J Med. 2018 Jan 25;378(4):321-30. PMID: 29365294. doi: 10.1056/NEJMoa1705971.

6. Budd Haeberlein S, Aisen PS, Barkhof F, Chalkias S, Chen T, Cohen S, et al. Two Randomized Phase 3 Studies of Aducanumab in Early Alzheimer's Disease. J Prev Alzheimers Dis. 2022;9(2):197-210. PMID: 35542991. doi: 10.14283/jpad.2022.30.

7. van Dyck CH, Swanson CJ, Aisen P, Bateman RJ, Chen C, Gee M, et al. Lecanemab in Early Alzheimer's Disease. N Engl J Med. 2023 Jan 5;388(1):9-21. PMID: 36449413. doi: 10.1056/NEJMoa2212948.

8. Swanson CJ, Zhang Y, Dhadda S, Wang J, Kaplow J, Lai RYK, et al. A randomized, double-blind, phase 2b proof-of-concept clinical trial in early Alzheimer's disease with lecanemab, an anti-Aβ protofibril antibody. Alzheimers Res Ther. 2021 Apr 17;13(1):80. PMID: 33865446. doi: 10.1186/s13195-021-00813-

9. Sims JR, Zimmer JA, Evans CD, Lu M, Ardayfio P, Sparks J, et al. Donanemab in Early Symptomatic Alzheimer Disease: The TRAILBLAZER-ALZ 2 Randomized Clinical Trial. Jama. 2023 Aug 8;330(6):512-27. PMID: 37459141. doi: 10.1001/jama.2023.13239.

10. Mintun MA, Lo AC, Duggan Evans C, Wessels AM, Ardayfio PA, Andersen SW, et al. Donanemab in Early Alzheimer's Disease. N Engl J Med. 2021 May 6;384(18):1691-704. PMID: 33720637. doi: 10.1056/NEJMoa2100708.

11. Bateman RJ, Smith J, Donohue MC, Delmar P, Abbas R, Salloway S, et al. Two Phase 3 Trials of Gantenerumab in Early Alzheimer's Disease. N Engl J Med. 2023 Nov 16;389(20):1862-76. PMID: 37966285. doi: 10.1056/NEJMoa2304430.

12. Ostrowitzki S, Bittner T, Sink KM, Mackey H, Rabe C, Honig LS, et al. Evaluating the Safety and Efficacy of Crenezumab vs Placebo in Adults With Early Alzheimer Disease: Two Phase 3 Randomized Placebo-Controlled Trials. JAMA Neurol. 2022 Nov 1;79(11):1113-21. PMID: 36121669. doi: 10.1001/jamaneurol.2022.2909.

13. Salloway S, Honigberg LA, Cho W, Ward M, Friesenhahn M, Brunstein F, et al. Amyloid positron emission tomography and cerebrospinal fluid results from a crenezumab anti-amyloid-beta antibody double-blind, placebo-controlled, randomized phase II study in mild-to-moderate Alzheimer's disease (BLAZE). Alzheimers Res Ther. 2018 Sep 19;10(1):96. PMID: 30231896. doi: 10.1186/s13195-018-0424-5.

14. Ostrowitzki S, Lasser RA, Dorflinger E, Scheltens P, Barkhof F, Nikolcheva T, et al. A phase III randomized trial of gantenerumab in prodromal Alzheimer's disease. Alzheimers Res Ther. 2017 Dec 8;9(1):95. PMID: 29221491. doi: 10.1186/s13195-017-0318-y.
