# Supplementary material for: Clinical Benefits and Risks of Antiamyloid Antibodies in Sporadic Alzheimer Disease: Systematic Review and Network Meta-Analysis With a Web Application
Source: J Med Internet Res. 2025 Apr 7;27:e68454. doi: 10.2196/68454 (PMC12012406; doi:10.2196/68454)
Supplement: Multimedia Appendix 4 [file jmir_v27i1e68454_app4.docx]

### **Multimedia Appendix 4.** Inclusion and exclusion criteria of primary studies (clinical trials) included in this review.

**Abbreviations**: ß-hCG beta-human chorionic gonadotropin assay; AD - Alzheimer's Disease; AChE - acetylcholinesterase; ADAS-Cog - Alzheimer's Disease Assessment Scale-Cognitive Subscale; APOE – apolipoprotein E; BMI - Body Mass Index; CDR-SB - Clinical Dementia Rating Scale–Sum of Boxes; CDR-GS - Clinical Dementia Rating-Global Score; CSF – cerebrospinal fluid; DSM - Diagnostic and Statistical Manual of Mental Disorders; GDS-15 - Geriatric Depression Scale; MCI – Mild Cognitive Impairment; MMSE – Mini-Mental State Examination, MRI - magnetic resonance imaging; NIA-AA - National Institute of Aging - Alzheimer's Association; PET - positron emission tomography; TIA - transient ischemic attacks

| **Study (Year)** | **Inclusion criteria** | **Exclusion criteria** |
| --- | --- | --- |
| Salloway et al [1] (2009) | Probable AD with MRI consistent with AD diagnosis and: ▪Age 50-85 ▪MMSE score o of 16–26 (inclusive) at baseline.  ▪Rosen Modified Hachinski Ischemic score ≤4 ▪Stable doses of medications ▪Fluency in English | Clinically significant neurologic disease other than AD; a major psychiatric disorder. history of stroke or seizures. a Hamilton Rating Scale score for Depression >12; current anticonvulsant. antiparkinsonian. anticoagulant. or narcotic medications; recent immunosuppressive or cancer chemotherapy medications; or cognitive enhancers other than acetylcholinesterase inhibitors or memantine at a stable dose for at least 120 days before screening. |
| Salloway et al [2] (2014) (Study 301 and Study 302) | diagnosis of probable AD. age between 50-89 and: ▪ MMSE score of 16-26 inclusive. ▪ Brain MRI scan consistent with the diagnosis of AD. ▪ Stable doses of medications (cholinesterase inhibitors and memantine allowed). ▪ Caregiver able to attend all clinic visits with patient. | Neurologic disease other than Alzheimer’s disease; a screening brain MRI scan that showed evidence of an abnormality (two or more microhemorrhages. a prior hemorrhage larger than 1 cm^3^. two or more lacunar infarcts. a prior infarct larger than 1 cm^3^. or space-occupying lesions); a major psychiatric disorder; a history of stroke or seizures; and treatment with cognitive enhancers other than stable doses of acetylcholinesterase inhibitors or memantine. |
| Vandenberghe et al [3] (2016) | ▪ Diagnosis of probable AD, with MMSE score of 16-26. and brain MRI consistent with the diagnosis of AD. ▪ Concurrent use of cholinesterase inhibitor or memantine allowed. if stable. ▪ Caregiver will participate and be able to attend clinic visits with patient | Clinically significant neurologic disease other than AD; a major psychiatric disorder. history of stroke or seizures; a Hamilton Rating Scale score for Depression >12; current anticonvulsant; antiparkinsonian; anticoagulant; or narcotic medications; recent immunosuppressive or cancer chemotherapy medications; or cognitive enhancers other than acetylcholinesterase inhibitors or memantine at a stable dose for at least 120 days before screening. |
| Doody et al [4] 1 and 2 (2014) EXPEDITION 1 and 2 | Clinical criteria for MCI due to AD or mild AD and: ▪ Meet criteria for mild to moderate AD with MMSE score of 16 through 26 at screening. ▪ Modified Hachinski Ischemia Scale score of less than or equal to 4. ▪ GDS-15 score of less than or equal to 6. ▪ Have MRI or CT scan in the last 2 years with no findings inconsistent with a diagnosis of AD. ▪ If receiving concurrent AD treatment. must be on the medication for at least 4 months at a stable dose for at least 2 months prior to randomization. | Serious or unstable illness(es); not having a reliable caregiver who is in frequent contact with patient (at least 10 hours per week); meets National Institute of Neurological Disorders and Stroke/Association Internationale pour la Recherche et l'Enseignement en Neurosciences (NINDS/AIREN) criteria for vascular dementia; not having good venous access. such that intravenous drug delivery would be difficult; multiple episodes of head trauma or history within the last 5 years of a serious infectious disease affecting the brain; allergies to humanized monoclonal antibodies; chronic alcohol and/or drug abuse within the past 5 years. has any contraindications for MRI studies or requires treatment with another monoclonal antibody |
| Honig et al [5] (2018) EXPEDITION 3 | Clinical criteria for MCI due to AD or mild AD and: ▪ Meet criteria for mild to moderate AD with MMSE score of 16-26 at screening. ▪ Modified Hachinski Ischemia Scale score of less than or equal to 4. ▪ GDS-15 score of less than or equal to 6. ▪ MRI or CT scan in the last 2 years with no findings inconsistent with a diagnosis of AD. ▪ A florbetapir PET scan or CSF result consistent with the presence of amyloid pathology at screening ▪ Concomitant therapy. including treatments for symptoms of dementia (acetylcholinesterase inhibitors and memantine. alone or in combination) and nondrug treatments. was allowed. If receiving concurrent AD treatment. must be on the medication for at least 4 months at a stable dose for at least 2 months prior to randomization. | Exclusion criteria were the same as in EXPEDITION 1 and 2 |
| Haeberlein et al [6] (2022) EMERGE and ENGAGE | Clinical criteria for MCI due to AD or mild AD and: ▪ CDR-SB of 0.5. ▪ Objective evidence of cognitive impairment at screening. ▪ MMSE score between 24 and 30 (inclusive). ▪ Positive amyloid PET scan. ▪ Consent to *APOE* genotyping. ▪ Reliable informant or caregiver. ▪ If using drugs to treat symptoms related to AD. doses must be stable for at least 8 weeks prior to screening visit. 2 ▪ Vaccinations with live or attenuated vaccines were allowed during the study. | Brain MRI was used to exclude patients with confounding pathologies. including acute or sub-acute hemorrhage. more than four microhemorrhages. cortical infarcts. >1 lacunar infarct. superficial siderosis. or a history of white matter disease as defined by protocol. or conditions that posed a risk to the patient or prevented MRI monitoring (full exclusion criteria are listed in Supplement 1). Patients with medical conditions possibly contributing to cognitive impairment were also excluded. Stable use of concomitant medications for chronic conditions was permitted during the study. except as defined in the protocol. Use of aspirin at a prophylactic dose (≤325 mg daily) was permitted. but use of any other medications with anti-platelet or anticoagulant properties was exclusionary. For cholinesterase inhibitors and memantine, patients were required to be on a stable dose before screening. with no dose adjustment during the study. Vaccinations with live or attenuated vaccines were allowed. |
| Swanson et al [7] (2021) | • Subjects who meet the NIA-AA core clinical criteria for mild cognitive impairment due to Alzheimer's disease - intermediate likelihood • Subjects who CDR score of 0.5 and a Memory Box score of 0.5 or greater at Screening and Baseline • Subjects who report a history of subjective memory decline with gradual onset and slow progression over the last one year before Screening; MUST be corroborated by an informant • Key Inclusion Criteria (Core Study) for Mild Alzheimer's Disease Dementia: • Subjects who meet the NIA-AA core clinical criteria for probable Alzheimer's disease dementia • Subjects who CDR score of 0.5-1.0 and a Memory Box score of 0.5 or greater at Screening and Baseline • Inclusion Criteria (Core Study) that must be met by all subjects: • Subjects with objective impairment in episodic memory as indicated by at least 1 standard deviation below age-adjusted mean in the Wechsler Memory Scale - IV Logical Memory II (WMS-IV LMII): - Less than or equal to 15 for age 50 to 64 years - Less than or equal to 12 for age 65 to 69 years - Less than or equal to 11 for age 70 to 74 years - Less than or equal to 9 for age 75 to 79 years - Less than or equal to 7 for age 80 to 90 years • Positive amyloid load as indicated by PET or CSF assessment • PET assessment of imaging agent uptake into brain • CSF assessment of Aβ(1-42) • Age between 50 and 90 years. inclusive • MMSE score equal to or greater than 22. and equal to or less than 30. at Screening and Baseline • BMI greater than 17 and less than 35 at Screening or Baseline • Females must not be lactating or pregnant at Screening or Baseline (negative ß-hCG). A separate baseline assessment is required if a negative pregnancy test was obtained more than 72 hours before the first dose of study drug. • Subjects on acetylcholinesterase inhibitor or memantine therapy or both for AD must be on a stable dose for at least 12 weeks prior to Baseline. Treatment naive subjects can be entered into the study. Unless otherwise stated. subjects must have been on stable doses of all other permitted concomitant non-AD medications for at least 4 weeks prior to Baseline. • Subjects must have identified caregivers/informants • Subjects must provide written informed consent ▪ If receiving concurrent AD treatment. must be on the medication for at least 4 months at a stable dose for at least 2 months prior to randomization. | Any neurological condition that may be contributing to cognitive impairment above and beyond that caused by the subject's AD; history of TIA, stroke. or seizures within 12 months of Screening; any psychiatric diagnosis or symptoms. (e.g., hallucinations. major depression. or delusions) that could interfere with study procedures in the subject GDS-15 (GDS) score ≥8 at Screening; contraindications to MRI scanning. including cardiac pacemaker/ defibrillator. Ferromagnetic implants. e.g., in skull and cardiac devices other than those approved as safe for use in MR scanners; evidence of other clinically significant lesions that could indicate a dementia diagnosis other than AD on brain MRI at Screening. or other significant pathological findings on brain MRI at Screening; prolonged QT/QTc interval (QTc greater than 450 ms) as demonstrated by a repeated electrocardiogram (ECG); Certain other specified medical conditions; Severe visual or hearing impairment that would prevent the subject from performing psychometric tests accurately |
| van Dyck et al [8] (2023) Clarity AD | MCI due to AD; age between 50-90; BMI between 17 and 35 and: ▪ Global Clinical Dementia Rating (CDR) score of 0.5 and CDR Memory Box score of 0.5 or greater at Screening and Baseline; MMSE between 22 and 30 ▪ Report a history of subjective memory decline with gradual onset and slow progression over the last 1 year before Screening. ▪ BMI greater than (>)17 and less than (<) 35 at Screening ▪ If receiving an approved treatment such as acetylcholinesterase inhibitor or memantine or both; the patient must be on a stable dose for at least 12 weeks prior to baseline. Use of memantine not allowed for participants in Japan. ▪ Have an identified person able to support the participant for the duration of the study and who spends at least 8 hours per week with the participant) ▪ Provide written informed consent | Excluded for any neurological condition that may be contributing to cognitive impairment above and beyond that caused by the participant's Alzheimer's disease; history of TIA. stroke. or seizures within 12 months of Screening; any psychiatric diagnosis or symptoms (example. hallucinations. major depression. or delusions) that could interfere with study procedures in the participant; having GDS-15 (GDS) score >=8 at Screening; contraindications to MRI scanning. including cardiac pacemaker/defibrillator. ferromagnetic metal implants (example in skull and cardiac devices other than those approved as safe for use in MRI scanners); evidence of other clinically significant lesions on brain MRI at Screening that could indicate a dementia diagnosis other than Alzheimer's disease; other significant pathological findings on brain MRI at screening. including but not limited to: more than 4 microhemorrhages (defined as 10 millimeter [mm] or less at the greatest diameter); a single macrohemorrhage >10 mm at greatest diameter; an area of superficial siderosis; evidence of vasogenic edema; evidence of cerebral contusion. encephalomalacia. aneurysms. vascular malformations. or infective lesions; evidence of multiple lacunar infarcts or stroke involving a major vascular territory. severe small vessel. or white matter disease; space occupying lesions; or brain tumors (however. lesions diagnosed as meningiomas or arachnoid cysts and <1 centimeter [cm] at their greatest diameter need not be exclusionary); any immunological disease which is not adequately controlled. or which requires treatment with immunoglobulins. systemic monoclonal antibodies (or derivatives of monoclonal antibodies); systemic immunosuppressants. or plasmapheresis during the study; participants with a bleeding disorder that is not under adequate control (including a platelet count <50.000 or international normalized ratio [INR] >1.5 for participants who are not on anticoagulant treatment. example. warfarin). Participants who are on anticoagulant therapy should have their anticoagulant status optimized and be on a stable dose for 4 weeks before Screening. Participants who are on anticoagulant therapy are not permitted to participate in CSF assessments; any other medical conditions (example. cardiac. respiratory. gastrointestinal. renal disease) which are not stably and adequately controlled. or which in the opinion of the investigator(s) could affect the participant's safety or interfere with the study assessments; participation in a clinical study involving any therapeutic monoclonal antibody. protein derived from a monoclonal antibody. immunoglobulin therapy. or vaccine within 6 months before screening unless it can be documented that the participant was randomized to placebo; participation in a clinical study involving any anti-amyloid therapies (including any monoclonal antibody therapies and any β-site amyloid precursor protein cleaving enzyme [BACE] inhibitor therapies) unless it can be documented that the participant only received placebo; have any known prior exposure to lecanemab; dosed in a clinical study involving any new chemical entities for AD within 6 months prior to screening unless it can be documented that the participant was in a placebo treatment arm |
| Sims et al [9] (2023) TRAILBLAZER-ALZ 2 | Early symptomatic AD/MCI or AD with mild dementia and: ▪ Gradual and progressive change in memory function reported by participants or informants for ≥ 6 months. ▪ MMSE score of 20 to 28 (inclusive) at baseline. ▪ Amyloid pathology (≥37 Centiloids) assessed with florbetapir or florbetaben PET ▪ Meet flortaucipir PET scan (central read) criteria - did not apply to safety cohort. ▪ study partner who will provide written informed consent to participate. ▪ Stable concomitant symptomatic AD medications and other medications that may impact cognition for at least approximately 30 days prior to randomization (did not apply to topical or discontinued medications) | Contraindication to MRI or PET scans; significant neurological disease affecting the central nervous system other than AD. that may affect cognition or ability to complete the study. including but not limited to. other - epilepsy or recurrent seizures (except febrile childhood seizures). current serious or unstable illnesses including cardiovascular, hepatic, renal, gastroenterological, respiratory, endocrinologic, neurologic (other than AD), psychiatric, immunologic or hematologic disease and other conditions that, in the opinion of the investigator, could interfere with the analyses - Has life expectancy of < 24 months or history of cancer within the last 5 years. with the exception of non-metastatic basal and/or squamous cell carcinoma of the skin. in situ cervical cancer. nonprogressive prostate cancer. or other cancers with low risk of recurrence or spread. - Participants with any current primary psychiatric diagnosis other than AD if. in the judgment of the investigator, the psychiatric disorder or symptom is likely to confound interpretation of drug effect. affect cognitive assessment. or affect the participant to complete the study. Participants with history of schizophrenia or other chronic psychosis are excluded. - Actively suicidal and therefore deemed to be at significant risk for suicide. - History of alcohol or drug use disorder (except tobacco use disorder) within 2 years before the screening visit. - History of clinically significant multiple or severe drug allergies. significant atopy. or severe posttreatment hypersensitivity reactions (including but not limited to erythema multiforme major. linear immunoglobulin A dermatosis. toxic epidermal necrolysis. and/or exfoliative dermatitis). - Current treatment with immunoglobulin G (IgG) therapy - Presence of amyloid-related imaging abnormalities of edema/effusion (ARIA-E). more than 4 cerebral microhemorrhages. more than 1 area of superficial siderosis. and any intracerebral hemorrhage > 1 cm or severe white matter disease on MRI.  - Are investigator site personnel directly affiliated with this study and/or their immediate families. Immediate family is defined as a spouse. parent. child. or sibling. whether biological or legally adopted. - Are Lilly employees or are employees of third-party organizations (TPOs) involved in study which requires exclusion of their employees. or have study partners who are Lilly employees or are employees of TPOs involved in a study which requires exclusion of their employees. - Have participated. within the last 30 days (4 months for studies conducted in Japan; 3 months for studies conducted in the United Kingdom). in a clinical trial involving an IP; if the previous IP is scientifically or medically incompatible with this study and has a long half-life. 3 months or 5 half-lives (whichever is longer) should have passed prior to screening (participation in observational studies may be permitted upon review of the observational study protocol and approval by the sponsor). - Have previously completed or withdrawn from this study or received donanemab in any prior investigational study. (This exclusion criterion does not apply to participants who are allowed to rescreen before randomization in this study). - Are currently enrolled in any other interventional clinical trial involving an IP or any other type of medical research judged not to be scientifically or medically compatible with this study. - Have had prior treatment with a passive anti-amyloid immunotherapy <5 half-lives prior to randomization.; have known allergies to donanemab. related compounds. or any components of the formulation. - Have any clinically important abnormality at screening. as determined by investigator. in physical or neurological examination. vital signs. ECG. or clinical laboratory test results that could be detrimental to the participant. could compromise the study. or show evidence of other etiologies for dementia; alanine aminotransaminase (ALT) more than 2.5X the upper limit of normal (ULN) of the performing laboratory. aspartate aminotransferase (AST more than 2.5X ULN. total bilirubin level (TBL) more than 1.5X ULN. or alkaline phosphatase (ALP) more than 2 X ULN at screening. - Screening MRI which shows evidence of significant abnormality that would suggest another potential etiology for progressive dementia or a clinically significant finding that may impact the participant  - Have any contraindications for MRI. including claustrophobia or the presence of contraindicated ferromagnetic implants or cardiac pacemakers. - centrally read MRI demonstrating presence of ARIA-E. >4 cerebral microhemorrhages. more than 1 area of superficial siderosis. any macrohemorrhages or severe white matter disease at screening. - Sensitivity to florbetapir F18 or flortaucipir F18. - Poor venous access. - Contraindication to PET. - Present or planned exposure to ionizing radiation that. in combination with the planned administration of study PET ligands. would result in a cumulative exposure that exceeds local recommended exposure limits |
| Mintun et al [10] (2021) TRAILBLAZER-ALZ | ▪Gradual and progressive change in memory function reported by participants or informants for ≥ 6 months. ▪ MMSE score of 20 to 28 (inclusive) at baseline or an acceptable historical flortaucipir PET scan within 6 months prior to baseline that meets the central read criteria. ▪ Meet 18F flortaucipir PET scan eligibility criteria. ▪ Meet 18F florbetapir PET scan (central read) eligibility criteria. | History of long QT syndrome or contraindication to MRI; have received treatment with a stable dose of an AChE inhibitor and/or memantine for less than 2 months before randomization; C; Patients with an SUVR of more than 1.46 were considered to high tau level and were excluded from the trial. Patients with an SUVR of less than 1.10 or with a deposition pattern not consistent with Alzheimer’s disease were considered to have an inadequate tau level and were excluded from the trial. except for patients with an SUVR of less than 1.10 but with a topographic deposition pattern consistent with advanced Alzheimer’s disease. who were included. The flortaucipir PET screening criteria led to the exclusion of patients with the highest tau levels. who are hypothesized to have disease that is more resistant to anti-amyloid treatments |
| Bateman et al [11] (2023) GRADUATE I and GRADUATE II | ▪ Demonstrated abnormal memory function and meets National Institute on Aging/Alzheimer's Association (NIAAA) core clinical criteria for probable AD dementia or prodromal AD (consistent with the NIAAA diagnostic criteria and guidelines for mild cognitive impairment) ▪ Evidence of the AD pathological process. as confirmed by CSF tau/A-beta42or amyloid PET scan ▪ MMSE score greater than or equal to 22 (≥ 22) ▪ CDR-GS of 0.5 or 1.0 ▪ If receiving symptomatic AD medications; the dosing regimen must have been stable for 3 months prior to screening and until randomization ▪ Availability of a reliable study partner who accepts to participate in study procedures throughout the 2 years duration of study. Furthermore. for women of childbearing potential: agreement to remain abstinent (refrain from heterosexual intercourse) or use contraceptive methods | Patients excluded if they were taking anticoagulants or GV-971 (an oligosaccharide intended to reduce inflammation in the brain by regulating the gut microbiota); or if they had clinically significant findings on MRI at screening that could cause cognitive impairment, such as more than five microhemorrhages, more than two lacunar infarcts, or a Fazekas score of 3, indicating that confluent areas of the brain are affected by white-matter hyperintensity; any evidence of a condition other than AD that may affect cognition. including. but not limited to. frontotemporal dementia. dementia with Lewy bodies. vascular dementia. Parkinson’s disease. corticobasal syndrome. Creutzfeldt–Jakob disease. progressive supranuclear palsy. frontotemporal lobar degeneration. Huntington’s disease. normal pressure hydrocephalus. seizure disorder. delirium. or hypoxia; History or presence of clinically evident systemic vascular disease (e.g., clinically significant carotid/vertebral artery stenosis or plaque. aortic aneurysm). that in the opinion of the investigator has the potential to affect cognitive function; History or presence of clinically evident cerebrovascular disease (e.g., intracranial or cerebral vascular malformations. aneurysm. intracranial macrohemorrhages); Participants with asymptomatic developmental venous anomalies may be eligible after discussion with an approval by the medical monitor: History or presence of posterior reversible encephalopathy syndrome; History or presence of any stroke with clinical symptoms within the past 12 months. or documented history within the last 6 months of an acute event that is consistent. in the opinion of the investigator. with a transient ischemic attack; History of severe. clinically significant (persistent neurologic deficit or structural brain damage) CNS trauma (e.g., cerebral contusion); History or presence of intracranial mass (e.g., glioma. meningioma) that could potentially impair cognition; Presence of infections that affect brain function or history of infections that resulted in neurologic sequelae (e.g., HIV. syphilis. neuroborreliosis. and viral or bacterial meningitis and encephalitis); History or presence of systemic autoimmune disorders that potentially cause progressive neurologic disease with associated cognitive deficits (e.g., multiple sclerosis. lupus erythematosus. antiphospholipid antibody syndrome. and Behçet’s disease); History of schizophrenia. schizoaffective disorder. major depression. or bipolar disorder; History of major depression is acceptable if participant has had no episode within the past year or is considered in remission or depression is controlled by treatment; At risk of suicide in the opinion of the investigator; Alcohol and/or substance abuse or dependence (according to the criteria specified in the DSM 5) within the past 2 years – Nicotine use is allowed – Marijuana use is not allowed and must be discontinued at least 3 months before screening ; According to the MRI central reader. MRI evidence of any of the following: – >2 lacunar infarcts – Any territorial infarct >1 cm^3^ – Any white matter lesion that corresponds to an overall Fazekas score of 3 that requires at least 1 confluent hyperintense lesion on the fluid-attenuated inversion recovery sequence. which is ≥20 mm in any dimension; Combined number of microbleeds and areas of leptomeningeal hemosiderosis (i.e., cumulative amyloid-related imaging abnormalities – hemosiderosis) on MRI more than 5 (and should not include any disseminated leptomeningeal hemosiderosis) based on the review performed by the central reader prior to randomization; Presence of any other significant cerebral abnormalities. including amyloid-related imaging abnormalities – edema. as assessed on MRI ; Inability to tolerate MRI procedures or contraindication to MRI. including. but not limited to. presence of pacemakers not compatible with MRI. aneurysm clips. artificial heart valves. ear implants. or foreign metal (ferromagnetic) objects in the eyes. skin. or body that would contraindicate an MRI scan. or any other clinical history or examination finding that. in the judgment of the investigator. would pose a potential hazard in combination with MRI ; History or presence of clinically symptomatic atrial fibrillation requiring maintenance therapy; Participants who experienced non-valvular atrial fibrillation that resolved more than 1 year ago and was not associated with prior stroke are eligible; Within the last year. unstable or clinically significant cardiovascular disease (e.g., myocardial infarction); Heart failure classified as New York Heart Association Class I or II cardiac disease is allowed. if not associated with hospitalization within the previous 12 months; Uncontrolled hypertension (e.g., blood pressure generally >160 mmHg systolic or >95 mmHg diastolic); chronic kidney disease. indicated by creatinine clearance <30 ml/min to be calculated by the central laboratory using the Cockcroft–Gault formula at screening. which remains <30 ml/min if retested; Confirmed and unexplained impaired hepatic function as indicated by screening aspartate aminotransferase (AST) or alanine transaminase (ALT) ≥3x the upper limit of normal (ULN) or total bilirubin ≥2x ULN ; History of. or known to currently have HIV infection. or hepatitis B or hepatitis C infection that has not been adequately treated in the opinion of the investigator. or history of spirochete infection of the CNS (e.g., syphilis. Lyme disease. or borreliosis); Systemically. clinically. significantly immunocompromised participants. owing to continuing effects of immune-suppressing medication; Abnormal thyroid function as indicated by abnormal screening tests that are judged to be clinically significant by the investigator. or abnormal thyroid function that requires a new treatment or an adjustment of current treatment; A participant may be rescreened if there is no improvement in cognition in the investigator's judgment after 3 months of adequate treatment for thyroid function; Participants with evidence of folic acid deficiency (as indicated by folic acid level below the lower limit of normal) or vitamin B12 deficiency (as indicated by vitamin B12 level below the lower limit of normal and/or methylmalonic acid levels above ULN); A participant may be rescreened if there is no improvement in cognition after 3 months of adequate treatment for folic acid or vitamin B12 deficiency; Screening hemoglobin A1c (HbA1C) > 8% (retesting is permitted if slightly elevated) or poorly controlled insulin-dependent diabetes (including hypoglycemic episodes) – A participant may be rescreened after 3 months to allow optimization of diabetic control ; Any previous administration of gantenerumab or active immunotherapy (vaccine) that is being evaluated to prevent or postpone cognitive decline; Any passive immunotherapy (immunoglobulin) or other long-acting biologic agent to prevent or postpone cognitive decline within 1 year of screening ; Any other investigational treatment within 5 half-lives or 4 months prior to screening. whichever is longer; Any previous administration of GV-971; Any previous treatment with medications specifically intended to treat Parkinson’s symptoms or any other neurodegenerative disorder within 1 year of screening. with no plans to initiate such medications prior to randomization; Certain medications are acceptable if the participant is taking the medicine for a non-neurodegenerative disorder. such as restless leg disorder (e.g., pramipexole); Typical antipsychotic or neuroleptic medication within 6 months of screening and with no planned changes prior to randomization except as brief treatment for a nonpsychiatric indication (e.g., emesis) – Atypical antipsychotic medications are not allowed except for intermittent short-term use. and will need to be discontinued 2 days or at least 5 half-lives (whichever is longer) prior to any neurocognitive assessment; Anticoagulation medications within 3 months of screening with no plans to initiate any prior to randomization – Anti-platelet treatments (e.g., aspirin. clopidogrel. and dipyridamole) are permitted – Short-term. perioperative use of anticoagulant medications will not result in permanent discontinuation from the study; however. for any such use it is recommended to prospectively seek advice from the medical monitor and temporary study drug interruption may be required; Chronic use of opiates or opioids (including long-acting opioid medication) within 3 months of screening with no plans to initiate any. prior to randomization – Intermittent short-term use of short-acting opioid medications for pain is permitted except within 2 days or 5 half-lives (whichever is longer) prior to any neurocognitive assessment; Stimulant medications (amphetamine. methylphenidate preparations. or modafinil) within 1 month of screening and throughout the study; Chronic use of benzodiazepines. barbiturates. or hypnotics from 3 months before screening and with no plans to initiate any. prior to randomization – Intermittent short-term use of benzodiazepines. buspirone. or short-acting hypnotic medication for sleep or anxiety is allowed except within 2 days or 5 half-lives (whichever is longer) prior to any neurocognitive assessment; Other causes of intellectual disability that may account for cognitive deficits observed at screening (e.g., static encephalopathy. closed brain injury. mental retardation); For example, this may be based on the participant’s sufficient education or work experience; Pregnancy or breastfeeding. or intention of becoming pregnant during the study; Deformity of the lumbosacral region of the spine that. in the opinion of the investigator. would contraindicate lumbar puncture in participants who will have lumbar puncture; Clinically significant abnormal screening blood. CSF (if applicable). or urine results that remain abnormal at retest; Impaired coagulation (screening prothrombin time >1.2x the ULN that remains abnormal on retest); History of cancer. with the following exceptions: – If considered to be cured – If not being actively treated with anticancer therapy or radiotherapy and. in the opinion of the investigator. is not likely to require treatment in the ensuing 5 years; For prostate cancer or basal cell carcinoma. no significant progression over the previous 2 years; Known history of severe allergic. anaphylactic. or other hypersensitivity reactions to chimeric. human. or humanized antibodies or fusion proteins. including gantenerumab; Hypersensitivity to any of gantenerumab excipients; Any other severe or unstable medical condition that. in the opinion of the investigator or sponsor. could be expected to progress. recur. or change to such an extent that it could put the participant at special risk. bias the assessment of the clinical or mental status of the participant to a significant degree. interfere with the participant’s ability to complete the study assessments. or would require the equivalent of institutional or hospital care; Residence in a skilled nursing facility such as a convalescent home or long-term care facility; participants who subsequently require residence in such facilities during the study may continue in the study and be followed for efficacy and safety. provided that they have a study partner who meets the minimum requirement |
| Ostrowitzki et al [12] (2022) CREAD | • Weight between 40 and 120 kg inclusive • Availability of a person (referred to as the "caregiver") who in the investigator's judgment: • Has frequent and sufficient contact with the participant to be able to provide accurate information regarding the participant's cognitive and functional abilities. agrees to provide information at clinic visits (which require partner input for scale completion), signs the necessary consent form. and has sufficient cognitive capacity to accurately report upon the participant's behavior and cognitive and functional abilities • Fluency in the language of the tests used at the study site • Adequate visual and auditory acuity. in the investigator's judgment. sufficient to perform the neuropsychological testing (eyeglasses and hearing aids are permitted) • Evidence of the AD pathological process. by a positive amyloid assessment either on CSF amyloid beta 1-42 levels as measured on the Elecsys beta-amyloid(1-42) test system or amyloid PET scan by qualitative read by the core/central PET laboratory • Demonstrated abnormal memory function at screening (up to 4 weeks before screening begins) or screening (FCSRT cueing index =<0.67 AND free recall =<27) • Screening MMSE score of greater than or equal to (>=) 22 points and CDR-GS of 0.5 or 1.0 • Meets National Institute on Aging/Alzheimer's Association (NIAAA) core clinical criteria for probable AD dementia or prodromal AD (consistent with the NIAAA diagnostic criteria and guidelines for MCI • If receiving symptomatic AD medications. the dosing regimen must have been stable for 3 months prior to screening • Participant must have completed at least 6 years of formal education after the age of 5 years | Any evidence of a condition other than AD that may affect cognition such as other dementias. stroke. brain damage. autoimmune disorders (e.g. multiple sclerosis) or infections with neurological sequelae; known history of severe allergic. anaphylactic. or other hypersensitivity reactions to chimeric. human. or humanized antibodies or fusion proteins history of major psychiatric illness such as schizophrenia or major depression (if not considered in remission); at risk of suicide in the opinion of the investigator; any abnormal MRI findings. such as presence of cerebral vascular pathology. cortical stroke. etc., or inability to tolerate MRI procedures or contraindication to MRI; unstable or clinically significant cardiovascular (e.g., myocardial infarction). kidney or liver disease; uncontrolled hypertension; screening hemoglobin A1c (HbA1C) >8%; poor peripheral venous access; history of cancer except if considered to be cured or if not being actively treated with anti-cancer therapy or radiotherapy. |
| Salloway et al [13] (2018) BLAZE (pooled) | • Diagnosis of probable AD according to the NINCDS-ADRDA criteria • MMSE score of 18-26 points at screening • GDS-15score of < 6 • Completion of 6 years of education (or good work history consistent with exclusion of mental retardation or other pervasive developmental disorders) • For patients currently receiving treatment with approved AD treatments (AChE inhibitors or memantine): Treatment initiated and continued for at least the last 3 months prior to randomization. at a stable dose for at least the last 2 months prior to randomization | Severe or unstable medical condition that. in the opinion of the investigator or sponsor. would interfere with the patient's ability to complete the study assessments or would require the equivalent of institutional or hospital care; history or presence of clinically evident vascular disease potentially affecting the brain (e.g., stroke. clinically significant carotid or vertebral stenosis or plaque. aortic aneurysm. intracranial aneurysm. cerebral hemorrhage. arteriovenous malformation); history of severe. clinically significant (persistent neurologic deficit or structural brain damage) central nervous system trauma (e.g., cerebral contusion); hospitalization within 4 weeks prior to screening; previous treatment with MABT5102A or any other therapeutic that targets Abeta; treatment with any biologic therapy within 5 half-lives or 3 months prior to screening. whichever is longer. with the exception of routinely recommended vaccinations. which are allowed |
| Ostrowitzki et al [14] (2017) SCarlet RoAD I and II | Participants with 50-85 years of age and prodromal Alzheimer's disease; not receiving memantine or cholinesterase inhibitors Has a study partner who in the investigator's judgement has frequent and sufficient contact with the participant as to be able to provide accurate information as to the participant's cognitive and functional abilities. who agrees to provide information at clinic visits which require partner input for scale completion ▪Has had sufficient education or work experience to exclude mental retardation ▪ Study partner has noticed a recent gradual decrease in participant's memory (over the last 12 months). which the participant may or may not be aware of ▪ Screening MMSE score of 24 or above ▪ CSF result consistent with the presence of amyloid pathology. ▪ Availability of a person ('caregiver') who in the investigator's judgment has frequent and sufficient contact with the participant. and is able to provide accurate information regarding the participant's cognitive and functional abilities  ▪Willingness and ability to complete all aspects of the study ▪Fluency in the language of the tests used at the study site; adequate visual and auditory acuity. in the investigator's judgment. sufficient to perform the neuropsychological testing (eye glasses and hearing aids are permitted); able and willing to travel to PET imaging center and complete the planned scanning sessions; past and planned exposure to ionizing radiation not exceeding safe and permissible levels Past and planned exposure to ionizing radiation not exceeding safe and permissible levels ▪Agreement not to participate in other research studies for the duration of this trial and its associated sub-studies; ▪If currently receiving approved medications for AD. the dosing regimen must have been stable for 3 months prior to screening | Excluded patients that have planned or are planning to have exposure to ionizing radiation and patients with other prior or current neurologic or medical disorder which may currently or during the course of the study impair cognition or psychiatric functioning. and those with history of stroke or transient ischemic attack within the last 12 months. history of schizophrenia. schizoaffective or bipolar disorder; those that currently meet criteria for major depression and those that have had. within the last 2 years, unstable or clinically significant cardiovascular disease (myocardial infarction. angina pectoris) |

| **Study Name (Year) Dose** | **Clinical Trial ID** | **Phase** | **Drug** |
| --- | --- | --- | --- |
| **Salloway et al (2009) high dose** | **NCT00112073** | **II** | **Bapineuzumab** |
| **Salloway et al 1 (2014) Study 301 low dose** | **NCT00574132** | **III** | **Bapineuzumab** |
| **Salloway et al 2 (2014) Study 301 high dose** | **NCT00574132** | **III** | **Bapineuzumab** |
| **Salloway et al 3 (2014) Study 302 low dose** | **NCT00575055** | **III** | **Bapineuzumab** |
| **Doody et al 1 (2014) EXPEDITION 1** | **NCT00905372** | **III** | **Solanezumab** |
| **Doody et al 2 (2014) EXPEDITION 2** | **NCT00904683** | **III** | **Solanezumab** |
| **Vandenberghe et al 1 (2016) low dose** | **NCT00667810** | **III** | **Bapineuzumab** |
| **Vandenberghe et al 2 (2016) high dose** | **NCT00667810** | **III** | **Bapineuzumab** |
| **Vandenberghe et al 3 (2016) low dose** | **NCT00676143** | **III** | **Bapineuzumab** |
| **Honig et al (2018) EXPEDITION 3** | **NCT01900665** | **III** | **Solanezumab** |
| **Haeberlein et al (2022) EMERGE low dose** | **NCT02484547** | **III** | **Aducanumab** |
| **Haeberlein et al (2022) EMERGE high dose** | **NCT02484547** | **III** | **Aducanumab** |
| **Haeberlein et al (2022) ENGAGE low dose** | **NCT02477800** | **III** | **Aducanumab** |
| **Haeberlein et al (2022) ENGAGE high dose** | **NCT02477800** | **III** | **Aducanumab** |
| **van Dyck et al (2023) Clarity AD** | **NCT03887455** | **III** | **Lecanemab** |
| **Swanson et al.1 (2021) high dose** | **NCT01767311** | **II** | **Lecanemab** |
| **Swanson et al.2 (2021) low dose** | **NCT01767311** | **II** | **Lecanemab** |
| **Sims et al (2023) TRAILBLAZER-ALZ 2 (pooled)** | **NCT04437511** | **III** | **Donanemab** |
| **Mintun et al (2021) TRAILBLAZER-ALZ** | **NCT03367403** | **II** | **Donanemab** |
| **Bateman et al (2023) GRADUATE I** | **NCT03444870** | **III** | **Gantenerumab** |
| **Bateman et al (2023) GRADUATE II** | **NCT03443973** | **III** | **Gantenerumab** |
| **Ostrowitzki et al (2022) CREAD** | **NCT02670083** | **III** | **Crenezumab** |
| **Salloway et al (2018) BLAZE (pooled)** | **NCT01397578** | **II** | **Crenezumab** |
| **Ostrowitzki et al (2017) SCarlet RoAD I** | **NCT01224106** | **III** | **Gantenerumab** |
| **Ostrowitzki et al (2017) SCarlet RoAD II** | **NCT01224106** | **III** | **Gantenerumab** |

**References**

1. Salloway S, Sperling R, Gilman S, Fox NC, Blennow K, Raskind M, et al. A phase 2 multiple ascending dose trial of bapineuzumab in mild to moderate Alzheimer disease. Neurology. 2009 Dec 15;73(24):2061-70. PMID: 19923550. doi: 10.1212/WNL.0b013e3181c67808.

2. Salloway S, Sperling R, Fox NC, Blennow K, Klunk W, Raskind M, et al. Two phase 3 trials of bapineuzumab in mild-to-moderate Alzheimer's disease. N Engl J Med. 2014 Jan 23;370(4):322-33. PMID: 24450891. doi: 10.1056/NEJMoa1304839.

3. Doody RS, Thomas RG, Farlow M, Iwatsubo T, Vellas B, Joffe S, et al. Phase 3 trials of solanezumab for mild-to-moderate Alzheimer's disease. N Engl J Med. 2014 Jan 23;370(4):311-21. PMID: 24450890. doi: 10.1056/NEJMoa1312889.

4. Vandenberghe R, Rinne JO, Boada M, Katayama S, Scheltens P, Vellas B, et al. Bapineuzumab for mild to moderate Alzheimer's disease in two global, randomized, phase 3 trials. Alzheimers Res Ther. 2016 May 12;8(1):18. PMID: 27176461. doi: 10.1186/s13195-016-0189-7.

5. Honig LS, Vellas B, Woodward M, Boada M, Bullock R, Borrie M, et al. Trial of Solanezumab for Mild Dementia Due to Alzheimer's Disease. N Engl J Med. 2018 Jan 25;378(4):321-30. PMID: 29365294. doi: 10.1056/NEJMoa1705971.

6. Budd Haeberlein S, Aisen PS, Barkhof F, Chalkias S, Chen T, Cohen S, et al. Two Randomized Phase 3 Studies of Aducanumab in Early Alzheimer's Disease. J Prev Alzheimers Dis. 2022;9(2):197-210. PMID: 35542991. doi: 10.14283/jpad.2022.30.

7. van Dyck CH, Swanson CJ, Aisen P, Bateman RJ, Chen C, Gee M, et al. Lecanemab in Early Alzheimer's Disease. N Engl J Med. 2023 Jan 5;388(1):9-21. PMID: 36449413. doi: 10.1056/NEJMoa2212948.

8. Swanson CJ, Zhang Y, Dhadda S, Wang J, Kaplow J, Lai RYK, et al. A randomized, double-blind, phase 2b proof-of-concept clinical trial in early Alzheimer's disease with lecanemab, an anti-Aβ protofibril antibody. Alzheimers Res Ther. 2021 Apr 17;13(1):80. PMID: 33865446. doi: 10.1186/s13195-021-00813-

9. Sims JR, Zimmer JA, Evans CD, Lu M, Ardayfio P, Sparks J, et al. Donanemab in Early Symptomatic Alzheimer Disease: The TRAILBLAZER-ALZ 2 Randomized Clinical Trial. Jama. 2023 Aug 8;330(6):512-27. PMID: 37459141. doi: 10.1001/jama.2023.13239.

10. Mintun MA, Lo AC, Duggan Evans C, Wessels AM, Ardayfio PA, Andersen SW, et al. Donanemab in Early Alzheimer's Disease. N Engl J Med. 2021 May 6;384(18):1691-704. PMID: 33720637. doi: 10.1056/NEJMoa2100708.

11. Bateman RJ, Smith J, Donohue MC, Delmar P, Abbas R, Salloway S, et al. Two Phase 3 Trials of Gantenerumab in Early Alzheimer's Disease. N Engl J Med. 2023 Nov 16;389(20):1862-76. PMID: 37966285. doi: 10.1056/NEJMoa2304430.

12. Ostrowitzki S, Bittner T, Sink KM, Mackey H, Rabe C, Honig LS, et al. Evaluating the Safety and Efficacy of Crenezumab vs Placebo in Adults With Early Alzheimer Disease: Two Phase 3 Randomized Placebo-Controlled Trials. JAMA Neurol. 2022 Nov 1;79(11):1113-21. PMID: 36121669. doi: 10.1001/jamaneurol.2022.2909.

13. Salloway S, Honigberg LA, Cho W, Ward M, Friesenhahn M, Brunstein F, et al. Amyloid positron emission tomography and cerebrospinal fluid results from a crenezumab anti-amyloid-beta antibody double-blind, placebo-controlled, randomized phase II study in mild-to-moderate Alzheimer's disease (BLAZE). Alzheimers Res Ther. 2018 Sep 19;10(1):96. PMID: 30231896. doi: 10.1186/s13195-018-0424-5.

14. Ostrowitzki S, Lasser RA, Dorflinger E, Scheltens P, Barkhof F, Nikolcheva T, et al. A phase III randomized trial of gantenerumab in prodromal Alzheimer's disease. Alzheimers Res Ther. 2017 Dec 8;9(1):95. PMID: 29221491. doi: 10.1186/s13195-017-0318-y.
