# Supplementary material for: Clinical Benefits and Risks of Antiamyloid Antibodies in Sporadic Alzheimer Disease: Systematic Review and Network Meta-Analysis With a Web Application
Source: J Med Internet Res. 2025 Apr 7;27:e68454. doi: 10.2196/68454 (PMC12012406; doi:10.2196/68454)
Supplement: Multimedia Appendix 5 [file jmir_v27i1e68454_app5.docx]

**Multimedia Appendix 5.** Quality of the included reports assessed by the modified Jadad scale with 9 items.

1. Was the study described as randomized?
2. Was the method of randomization appropriate?
3. Was the blinding described in the study?
4. Was the method of blinding appropriate?
5. Was there a description of withdrawals and dropouts?
6. Was there a clear description of the inclusion and exclusion criteria?
7. Were the methods used to assess adverse effects described?
8. Were the amyloid-related imaging abnormalities (ARIAs) reported depending on the APOE genotype?

(1 points if reported without allele dose, 2 points if reported separately depending on the allele dose)

1. Were the methods of statistical analysis described?

| **Author (Year): Trial Name** | **I** | **II** | **III** | **IV** | **V** | **VI** | **VII** | **VIII** | **IX** | **Total score** |
| --- | --- | --- | --- | --- | --- | --- | --- | --- | --- | --- |
| Salloway et al [1] (2009) | 1 | 1 | 1 | 1 | 1 | 1 | 1 | 1 | 1 | 9 |
| Salloway et al [2] (2014) Study 301 and 302 | 1 | 1 | 1 | 1 | 1 | 1 | 1 | 1 | 1 | 9 |
| Doody et al [3] (2014) EXPEDITION 1 and 2 | 1 | 1 | 1 | 1 | 1 | 1 | 1 | 0 | 1 | 8 |
| Vandenberghe et al [4] (2016) | 1 | 1 | 1 | 1 | 1 | 1 | 1 | 1 | 1 | 9 |
| Honig et al [5] (2018) EXPEDITION 3 | 1 | 1 | 1 | 1 | 1 | 1 | 1 | 0 | 1 | 8 |
| Haeberlein et al [6] (2022) EMERGE and ENGAGE | 1 | 1 | 1 | 1 | 1 | 1 | 1 | 1 | 1 | 9 |
| van Dyck et al [7] (2023) Clarity AD | 1 | 1 | 1 | 1 | 1 | 1 | 1 | 2 | 1 | 10 |
| Swanson et al [8] (2021) | 1 | 1 | 1 | 1 | 1 | 1 | 1 | 2 | 1 | 10 |
| Sims et al [9] (2023) TRAILBLAZER-ALZ 2 | 1 | 1 | 1 | 1 | 1 | 1 | 1 | 2 | 1 | 10 |
| Mintun et al [10] (2021) TRAILBLAZER-ALZ | 1 | 1 | 1 | 1 | 1 | 1 | 1 | 2 | 1 | 10 |
| Bateman et al [11] (2023) GRADUATE I and GRADUATE II | 1 | 1 | 1 | 1 | 1 | 1 | 1 | 2 | 1 | 10 |
| Ostrowitzki et al [12] (2022) CREAD | 1 | 1 | 1 | 1 | 1 | 1 | 1 | 2 | 1 | 10 |
| Salloway et al [13] (2018) BLAZE (pooled) | 1 | 1 | 1 | 1 | 1 | 1 | 1 | 2 | 1 | 10 |
| Ostrowitzki et al [14] (2017) SCarlet RoAD | 1 | 1 | 0 | 1 | 1 | 1 | 1 | 2 | 1 | 9 |

| **Study Name (Year) Dose** | **Clinical Trial ID** | **Phase** | **Drug** |
| --- | --- | --- | --- |
| **Salloway et al (2009) high dose** | **NCT00112073** | **II** | **Bapineuzumab** |
| **Salloway et al 1 (2014) Study 301 low dose** | **NCT00574132** | **III** | **Bapineuzumab** |
| **Salloway et al 2 (2014) Study 301 high dose** | **NCT00574132** | **III** | **Bapineuzumab** |
| **Salloway et al 3 (2014) Study 302 low dose** | **NCT00575055** | **III** | **Bapineuzumab** |
| **Doody et al 1 (2014) EXPEDITION 1** | **NCT00905372** | **III** | **Solanezumab** |
| **Doody et al 2 (2014) EXPEDITION 2** | **NCT00904683** | **III** | **Solanezumab** |
| **Vandenberghe et al 1 (2016) low dose** | **NCT00667810** | **III** | **Bapineuzumab** |
| **Vandenberghe et al 2 (2016) high dose** | **NCT00667810** | **III** | **Bapineuzumab** |
| **Vandenberghe et al 3 (2016) low dose** | **NCT00676143** | **III** | **Bapineuzumab** |
| **Honig et al (2018) EXPEDITION 3** | **NCT01900665** | **III** | **Solanezumab** |
| **Haeberlein et al (2022) EMERGE low dose** | **NCT02484547** | **III** | **Aducanumab** |
| **Haeberlein et al (2022) EMERGE high dose** | **NCT02484547** | **III** | **Aducanumab** |
| **Haeberlein et al (2022) ENGAGE low dose** | **NCT02477800** | **III** | **Aducanumab** |
| **Haeberlein et al (2022) ENGAGE high dose** | **NCT02477800** | **III** | **Aducanumab** |
| **van Dyck et al (2023) Clarity AD** | **NCT03887455** | **III** | **Lecanemab** |
| **Swanson et al.1 (2021) high dose** | **NCT01767311** | **II** | **Lecanemab** |
| **Swanson et al.2 (2021) low dose** | **NCT01767311** | **II** | **Lecanemab** |
| **Sims et al (2023) TRAILBLAZER-ALZ 2 (pooled)** | **NCT04437511** | **III** | **Donanemab** |
| **Mintun et al (2021) TRAILBLAZER-ALZ** | **NCT03367403** | **II** | **Donanemab** |
| **Bateman et al (2023) GRADUATE I** | **NCT03444870** | **III** | **Gantenerumab** |
| **Bateman et al (2023) GRADUATE II** | **NCT03443973** | **III** | **Gantenerumab** |
| **Ostrowitzki et al (2022) CREAD** | **NCT02670083** | **III** | **Crenezumab** |
| **Salloway et al (2018) BLAZE (pooled)** | **NCT01397578** | **II** | **Crenezumab** |
| **Ostrowitzki et al (2017) SCarlet RoAD I** | **NCT01224106** | **III** | **Gantenerumab** |
| **Ostrowitzki et al (2017) SCarlet RoAD II** | **NCT01224106** | **III** | **Gantenerumab** |

**References**

1. Salloway S, Sperling R, Gilman S, Fox NC, Blennow K, Raskind M, et al. A phase 2 multiple ascending dose trial of bapineuzumab in mild to moderate Alzheimer disease. Neurology. 2009 Dec 15;73(24):2061-70. PMID: 19923550. doi: 10.1212/WNL.0b013e3181c67808.

2. Salloway S, Sperling R, Fox NC, Blennow K, Klunk W, Raskind M, et al. Two phase 3 trials of bapineuzumab in mild-to-moderate Alzheimer's disease. N Engl J Med. 2014 Jan 23;370(4):322-33. PMID: 24450891. doi: 10.1056/NEJMoa1304839.

3. Doody RS, Thomas RG, Farlow M, Iwatsubo T, Vellas B, Joffe S, et al. Phase 3 trials of solanezumab for mild-to-moderate Alzheimer's disease. N Engl J Med. 2014 Jan 23;370(4):311-21. PMID: 24450890. doi: 10.1056/NEJMoa1312889.

4. Vandenberghe R, Rinne JO, Boada M, Katayama S, Scheltens P, Vellas B, et al. Bapineuzumab for mild to moderate Alzheimer's disease in two global, randomized, phase 3 trials. Alzheimers Res Ther. 2016 May 12;8(1):18. PMID: 27176461. doi: 10.1186/s13195-016-0189-7.

5. Honig LS, Vellas B, Woodward M, Boada M, Bullock R, Borrie M, et al. Trial of Solanezumab for Mild Dementia Due to Alzheimer's Disease. N Engl J Med. 2018 Jan 25;378(4):321-30. PMID: 29365294. doi: 10.1056/NEJMoa1705971.

6. Budd Haeberlein S, Aisen PS, Barkhof F, Chalkias S, Chen T, Cohen S, et al. Two Randomized Phase 3 Studies of Aducanumab in Early Alzheimer's Disease. J Prev Alzheimers Dis. 2022;9(2):197-210. PMID: 35542991. doi: 10.14283/jpad.2022.30.

7. van Dyck CH, Swanson CJ, Aisen P, Bateman RJ, Chen C, Gee M, et al. Lecanemab in Early Alzheimer's Disease. N Engl J Med. 2023 Jan 5;388(1):9-21. PMID: 36449413. doi: 10.1056/NEJMoa2212948.

8. Swanson CJ, Zhang Y, Dhadda S, Wang J, Kaplow J, Lai RYK, et al. A randomized, double-blind, phase 2b proof-of-concept clinical trial in early Alzheimer's disease with lecanemab, an anti-Aβ protofibril antibody. Alzheimers Res Ther. 2021 Apr 17;13(1):80. PMID: 33865446. doi: 10.1186/s13195-021-00813-

9. Sims JR, Zimmer JA, Evans CD, Lu M, Ardayfio P, Sparks J, et al. Donanemab in Early Symptomatic Alzheimer Disease: The TRAILBLAZER-ALZ 2 Randomized Clinical Trial. Jama. 2023 Aug 8;330(6):512-27. PMID: 37459141. doi: 10.1001/jama.2023.13239.

10. Mintun MA, Lo AC, Duggan Evans C, Wessels AM, Ardayfio PA, Andersen SW, et al. Donanemab in Early Alzheimer's Disease. N Engl J Med. 2021 May 6;384(18):1691-704. PMID: 33720637. doi: 10.1056/NEJMoa2100708.

11. Bateman RJ, Smith J, Donohue MC, Delmar P, Abbas R, Salloway S, et al. Two Phase 3 Trials of Gantenerumab in Early Alzheimer's Disease. N Engl J Med. 2023 Nov 16;389(20):1862-76. PMID: 37966285. doi: 10.1056/NEJMoa2304430.

12. Ostrowitzki S, Bittner T, Sink KM, Mackey H, Rabe C, Honig LS, et al. Evaluating the Safety and Efficacy of Crenezumab vs Placebo in Adults With Early Alzheimer Disease: Two Phase 3 Randomized Placebo-Controlled Trials. JAMA Neurol. 2022 Nov 1;79(11):1113-21. PMID: 36121669. doi: 10.1001/jamaneurol.2022.2909.

13. Salloway S, Honigberg LA, Cho W, Ward M, Friesenhahn M, Brunstein F, et al. Amyloid positron emission tomography and cerebrospinal fluid results from a crenezumab anti-amyloid-beta antibody double-blind, placebo-controlled, randomized phase II study in mild-to-moderate Alzheimer's disease (BLAZE). Alzheimers Res Ther. 2018 Sep 19;10(1):96. PMID: 30231896. doi: 10.1186/s13195-018-0424-5.

14. Ostrowitzki S, Lasser RA, Dorflinger E, Scheltens P, Barkhof F, Nikolcheva T, et al. A phase III randomized trial of gantenerumab in prodromal Alzheimer's disease. Alzheimers Res Ther. 2017 Dec 8;9(1):95. PMID: 29221491. doi: 10.1186/s13195-017-0318-y.
