# Supplementary material for: Clinical Benefits and Risks of Antiamyloid Antibodies in Sporadic Alzheimer Disease: Systematic Review and Network Meta-Analysis With a Web Application
Source: J Med Internet Res. 2025 Apr 7;27:e68454. doi: 10.2196/68454 (PMC12012406; doi:10.2196/68454)
Supplement: Multimedia Appendix 6 [file jmir_v27i1e68454_app6.docx]

### **Multimedia Appendix 6.** Excluded studies and reasons for exclusion.

**Abbreviations**: ß-hCG beta-human chorionic gonadotropin assay; AD - Alzheimer's Disease; AChE - acetylcholinesterase; ADAS-Cog - Alzheimer's Disease Assessment Scale-Cognitive Subscale; APOE – apolipoprotein E; BMI - Body Mass Index; CDR-SB - Clinical Dementia Rating Scale–Sum of Boxes; CDR-GS - Clinical Dementia Rating-Global Score; CSF – cerebrospinal fluid; DSM - Diagnostic and Statistical Manual of Mental Disorders; GDS-15 - Geriatric Depression Scale; MCI – Mild Cognitive Impairment; MMSE – Mini-Mental State Examination, MRI - magnetic resonance imaging; NIA-AA - National Institute of Aging - Alzheimer's Association; PET - positron emission tomography; TIA - transient ischemic attacks

| **Clinical Trial ID** | **Reasons for Exclusion** | **URL** |
| --- | --- | --- |
| NCT03852901 | Basic Science study | https://clinicaltrials.gov/study/NCT03852901 |
| NCT04973189 | Basic Science study | https://clinicaltrials.gov/study/NCT04973189 |
| NCT01024660 | Early Phase 1 | https://clinicaltrials.gov/study/NCT01024660 |
| NCT02045056 | Early Phase 1 | https://clinicaltrials.gov/study/NCT02045056 |
| NCT03587376 | Early Phase 1 | https://clinicaltrials.gov/study/NCT03587376 |
| NA | Follow-up study | https://onlinelibrary.wiley.com/doi/abs/10.1002/1099-1166(200112)16:1+%3C::AID-GPS569%3E3.0.CO;2-J |
| NA | Follow-up study | https://journals.sagepub.com/doi/abs/10.1177/153331750301800410 |
| 2018-002783-88 | Jadad Scale < 3 | https://www.clinicaltrialsregister.eu/ctr-search/trial/2018-002783-88/results |
| ACTRN12613000170729 | Jadad Scale < 3 | https://www.anzctr.org.au/Trial/Registration/TrialReview.aspx?id=363494&isReview=true |
| ACTRN12613000777796 | Jadad Scale < 3 | https://www.anzctr.org.au/Trial/Registration/TrialReview.aspx?id=364492&isReview=true |
| ACTRN12618001426279 | Jadad Scale < 3 | https://www.anzctr.org.au/Trial/Registration/TrialReview.aspx?id=375847&isReview=true |
| ACTRN12619000467134 | Jadad Scale < 3 | https://www.anzctr.org.au/Trial/Registration/TrialReview.aspx?id=377163&isReview=true |
| ACTRN12619000571178 | Jadad Scale < 3 | https://www.anzctr.org.au/Trial/Registration/TrialReview.aspx?id=377354&isReview=true |
| ACTRN12619001566123 | Jadad Scale < 3 | https://www.anzctr.org.au/Trial/Registration/TrialReview.aspx?id=378622&isReview=true |
| ACTRN12620000957998 | Jadad Scale < 3 | https://www.anzctr.org.au/Trial/Registration/TrialReview.aspx?id=380094&isReview=true |
| NA | Jadad Scale < 3 | https://pubmed.ncbi.nlm.nih.gov/20505438/ |
| NCT00018291 | Jadad Scale < 3 | https://clinicaltrials.gov/study/NCT00018291 |
| NCT00165724 | Jadad Scale < 3 | https://clinicaltrials.gov/study/NCT00165724 |
| NCT00174382 | Jadad Scale < 3 | https://clinicaltrials.gov/study/NCT00174382 |
| NCT00208819 | Jadad Scale < 3 | <https://www.clinicaltrials.gov/study/NCT00208819> |
| NCT00219232 | Jadad Scale < 3 | https://clinicaltrials.gov/study/NCT00219232 |
| NCT00245206 | Jadad Scale < 3 | https://clinicaltrials.gov/study/NCT00245206 |
| NCT00305903 | Jadad Scale < 3 | https://clinicaltrials.gov/study/NCT00305903 |
| NCT00380276 | Jadad Scale < 3 | https://clinicaltrials.gov/study/NCT00380276 |
| NCT00381238 | Jadad Scale < 3 | https://clinicaltrials.gov/study/NCT00381238 |
| NCT00490568 | Jadad Scale < 3 | https://clinicaltrials.gov/study/NCT00490568 |
| NCT00549601 | Jadad Scale < 3 | https://clinicaltrials.gov/study/NCT00549601 |
| NCT00561392 | Jadad Scale < 3 | https://clinicaltrials.gov/study/NCT00561392 |
| NCT00566501 | Jadad Scale < 3 | https://clinicaltrials.gov/study/NCT00566501 |
| NCT00571064 | Jadad Scale < 3 | https://clinicaltrials.gov/study/NCT00571064 |
| NCT00606476 | Jadad Scale < 3 | https://clinicaltrials.gov/study/NCT00606476 |
| NCT00622713 | Jadad Scale < 3 | https://clinicaltrials.gov/study/NCT00622713 |
| NCT00684944 | Jadad Scale < 3 | https://clinicaltrials.gov/study/NCT00684944 |
| NCT00704782 | Jadad Scale < 3 | https://clinicaltrials.gov/study/NCT00704782 |
| NCT00722046 | Jadad Scale < 3 | <https://www.clinicaltrials.gov/study/NCT00722046> |
| NCT00731224 | Jadad Scale < 3 | https://clinicaltrials.gov/study/NCT00731224 |
| NCT00800709 | Jadad Scale < 3 | https://clinicaltrials.gov/study/NCT00800709 |
| NCT00916617 | Jadad Scale < 3 | https://clinicaltrials.gov/study/NCT00916617 |
| NCT00933608 | Jadad Scale < 3 | https://clinicaltrials.gov/study/NCT00933608 |
| NCT00960531 | Jadad Scale < 3 | https://clinicaltrials.gov/study/NCT00960531 |
| NCT00998764 | Jadad Scale < 3 | https://clinicaltrials.gov/study/NCT00998764 |
| NCT01025466 | Jadad Scale < 3 | https://clinicaltrials.gov/study/NCT01025466 |
| NCT01047579 | Jadad Scale < 3 | https://clinicaltrials.gov/study/NCT01047579 |
| NCT01054976 | Jadad Scale < 3 | https://clinicaltrials.gov/study/NCT01054976 |
| NCT01127633 | Jadad Scale < 3 | https://clinicaltrials.gov/study/NCT01127633 |
| NCT01148498 | Jadad Scale < 3 | https://clinicaltrials.gov/study/NCT01148498 |
| NCT01152216 | Jadad Scale < 3 | https://clinicaltrials.gov/study/NCT01152216 |
| NCT01421056 | Jadad Scale < 3 | https://clinicaltrials.gov/study/NCT01421056 |
| NCT01478633 | Jadad Scale < 3 | https://clinicaltrials.gov/study/NCT01478633 |
| NCT01529619 | Jadad Scale < 3 | https://clinicaltrials.gov/study/NCT01529619 |
| NCT01585272 | Jadad Scale < 3 | https://clinicaltrials.gov/study/NCT01585272 |
| NCT01602393 | Jadad Scale < 3 | https://clinicaltrials.gov/study/NCT01602393 |
| NCT01614886 | Jadad Scale < 3 | https://clinicaltrials.gov/study/NCT01614886 |
| NCT01723826 | Jadad Scale < 3 | https://clinicaltrials.gov/study/NCT01723826 |
| NCT01948791 | Jadad Scale < 3 | https://clinicaltrials.gov/study/NCT01948791 |
| NCT01951118 | Jadad Scale < 3 | https://clinicaltrials.gov/study/NCT01951118 |
| NCT02051608 | Jadad Scale < 3 | <https://clinicaltrials.gov/study/NCT0205160> |
| NCT02079246 | Jadad Scale < 3 | https://clinicaltrials.gov/study/NCT02079246 |
| NCT02097056 | Jadad Scale < 3 | https://clinicaltrials.gov/study/NCT02097056 |
| NCT02245568 | Jadad Scale < 3 | https://clinicaltrials.gov/study/NCT02245568 |
| NCT02361242 | Jadad Scale < 3 | https://clinicaltrials.gov/study/NCT02361242 |
| NCT02550665 | Jadad Scale < 3 | https://clinicaltrials.gov/study/NCT02550665 |
| NCT02553928 | Jadad Scale < 3 | https://clinicaltrials.gov/study/NCT02553928 |
| NCT02586909 | Jadad Scale < 3 | https://clinicaltrials.gov/study/NCT02586909 |
| NCT02703636 | Jadad Scale < 3 | https://clinicaltrials.gov/study/NCT02703636 |
| NCT02787746 | Jadad Scale < 3 | https://clinicaltrials.gov/study/NCT02787746 |
| NCT02916056 | Jadad Scale < 3 | https://clinicaltrials.gov/study/NCT02916056 |
| NCT02921477 | Jadad Scale < 3 | https://clinicaltrials.gov/study/NCT02921477 |
| NCT03118947 | Jadad Scale < 3 | https://clinicaltrials.gov/study/NCT03118947 |
| NCT03197740 | Jadad Scale < 3 | https://clinicaltrials.gov/study/NCT03197740 |
| NCT03491150 | Jadad Scale < 3 | https://clinicaltrials.gov/study/NCT03491150 |
| NCT03594123 | Jadad Scale < 3 | https://clinicaltrials.gov/study/NCT03594123 |
| NCT04075435 | Jadad Scale < 3 | https://clinicaltrials.gov/study/NCT04075435 |
| NCT04123314 | Jadad Scale < 3 | https://clinicaltrials.gov/study/NCT04123314 |
| NCT04200911 | Jadad Scale < 3 | https://clinicaltrials.gov/study/NCT04200911 |
| NCT04263519 | Jadad Scale < 3 | https://clinicaltrials.gov/study/NCT04263519 |
| NCT04339413 | Jadad Scale < 3 | https://clinicaltrials.gov/study/NCT04339413 |
| NCT04374253 | Jadad Scale < 3 | https://clinicaltrials.gov/study/NCT04374253 |
| NCT04430517 | Jadad Scale < 3 | https://clinicaltrials.gov/study/NCT04430517 |
| NCT04552795 | Jadad Scale < 3 | https://clinicaltrials.gov/study/NCT04552795 |
| NCT04640077 | Jadad Scale < 3 | https://clinicaltrials.gov/study/NCT04640077 |
| NCT04785300 | Jadad Scale < 3 | https://clinicaltrials.gov/study/NCT04785300 |
| NCT04886063 | Jadad Scale < 3 | https://clinicaltrials.gov/study/NCT04886063 |
| NCT04947553 | Jadad Scale < 3 | https://clinicaltrials.gov/study/NCT04947553 |
| NCT05108922 | Jadad Scale < 3 | https://clinicaltrials.gov/study/NCT05108922 |
| NCT05161715 | Jadad Scale < 3 | https://clinicaltrials.gov/study/NCT05161715 |
| NCT05352763 | Jadad Scale < 3 | https://clinicaltrials.gov/study/NCT05352763 |
| NCT05575076 | Jadad Scale < 3 | https://clinicaltrials.gov/study/NCT05575076 |
| NCT06078891 | Jadad Scale < 3 | https://clinicaltrials.gov/study/NCT06078891 |
| 2020-000798-26 | No results posted | https://www.clinicaltrialsregister.eu/ctr-search/search?query=2020-000798-26 |
| ACTRN12618000761268 | No results posted | https://www.anzctr.org.au/Trial/Registration/TrialReview.aspx?id=373908&isReview=true |
| ACTRN12619000474156 | No results posted | https://www.anzctr.org.au/Trial/Registration/TrialReview.aspx?id=375128&isReview=true |
| ACTRN12619001756112 | No results posted | https://www.anzctr.org.au/Trial/Registration/TrialReview.aspx?id=378582&isReview=true |
| NCT00911807 | No results posted | https://clinicaltrials.gov/study/NCT00911807 |
| NCT00930059 | No results posted | https://clinicaltrials.gov/study/NCT00930059 |
| NCT01404169 | No results posted | https://clinicaltrials.gov/study/NCT01404169 |
| NCT01539031 | No results posted | https://clinicaltrials.gov/study/NCT01539031 |
| NCT01689233 | No results posted | https://clinicaltrials.gov/study/NCT01689233 |
| NCT01689246 | No results posted | https://clinicaltrials.gov/study/NCT01689246 |
| NCT01760005 | No results posted | https://clinicaltrials.gov/study/NCT01760005 |
| NCT01767311 | No results posted | <https://clinicaltrials.gov/study/NCT02434718> |
| NCT01841125 | No results posted | https://clinicaltrials.gov/study/NCT01841125 |
| NCT02085265 | No results posted | https://clinicaltrials.gov/study/NCT02085265 |
| NCT02327182 | No results posted | https://clinicaltrials.gov/study/NCT02327182 |
| NCT02380573 | No results posted | https://clinicaltrials.gov/study/NCT02380573 |
| NCT02444637 | No results posted | https://clinicaltrials.gov/study/NCT02444637 |
| NCT02446132 | No results posted | https://clinicaltrials.gov/study/NCT02446132 |
| NCT02471196 | No results posted | https://clinicaltrials.gov/study/NCT02471196 |
| NCT02547818 | No results posted | https://clinicaltrials.gov/study/NCT02547818 |
| NCT02792257 | No results posted | https://clinicaltrials.gov/study/NCT02792257 |
| NCT02833792 | No results posted | https://clinicaltrials.gov/study/NCT02833792 |
| NCT02931136 | No results posted | https://clinicaltrials.gov/study/NCT02931136 |
| NCT02955706 | No results posted | https://clinicaltrials.gov/study/NCT02955706 |
| NCT02989402 | No results posted | https://clinicaltrials.gov/study/NCT02989402 |
| NCT03082755 | No results posted | https://clinicaltrials.gov/study/NCT03082755 |
| NCT03108846 | No results posted | https://clinicaltrials.gov/study/NCT03108846 |
| NCT03116126 | No results posted | https://clinicaltrials.gov/study/NCT03116126 |
| NCT03185208 | No results posted | https://clinicaltrials.gov/study/NCT03185208 |
| NCT03363269 | No results posted | https://clinicaltrials.gov/study/NCT03363269 |
| NCT03393520 | No results posted | https://clinicaltrials.gov/study/NCT03393520 |
| NCT03446001 | No results posted | https://clinicaltrials.gov/study/NCT03446001 |
| NCT03454646 | No results posted | https://clinicaltrials.gov/study/NCT03454646 |
| NCT03486938 | No results posted | https://clinicaltrials.gov/study/NCT03486938 |
| NCT03620981 | No results posted | https://clinicaltrials.gov/study/NCT03620981 |
| NCT03649724 | No results posted | https://clinicaltrials.gov/study/NCT03649724 |
| NCT03724136 | No results posted | https://clinicaltrials.gov/study/NCT03724136 |
| NCT03724942 | No results posted | https://clinicaltrials.gov/study/NCT03724942 |
| NCT03817684 | No results posted | https://clinicaltrials.gov/study/NCT03817684 |
| NCT03823404 | No results posted | https://clinicaltrials.gov/study/NCT03823404 |
| NCT03875638 | No results posted | https://clinicaltrials.gov/study/NCT03875638 |
| NCT03919162 | No results posted | https://clinicaltrials.gov/study/NCT03919162 |
| NCT03954899 | No results posted | https://clinicaltrials.gov/study/NCT03954899 |
| NCT04004702 | No results posted | https://clinicaltrials.gov/study/NCT04004702 |
| NCT04032626 | No results posted | https://clinicaltrials.gov/study/NCT04032626 |
| NCT04241068 | No results posted | https://clinicaltrials.gov/study/NCT04241068 |
| NCT04314934 | No results posted | https://clinicaltrials.gov/study/NCT04314934 |
| NCT04408755 | No results posted | https://clinicaltrials.gov/study/NCT04408755 |
| NCT04421014 | No results posted | https://clinicaltrials.gov/study/NCT04421014 |
| NCT04436081 | No results posted | https://clinicaltrials.gov/study/NCT04436081 |
| NCT04437511 | No results posted | https://clinicaltrials.gov/study/NCT04437511 |
| NCT04464564 | No results posted | https://clinicaltrials.gov/study/NCT04464564 |
| NCT04466735 | No results posted | https://clinicaltrials.gov/study/NCT04466735 |
| NCT04468659 | No results posted | https://clinicaltrials.gov/study/NCT04468659 |
| NCT04488419 | No results posted | https://clinicaltrials.gov/study/NCT04488419 |
| NCT04516057 | No results posted | https://clinicaltrials.gov/study/NCT04516057 |
| NCT04592874 | No results posted | https://clinicaltrials.gov/study/NCT04592874 |
| NCT04601038 | No results posted | https://clinicaltrials.gov/study/NCT04601038 |
| NCT04619420 | No results posted | https://clinicaltrials.gov/study/NCT04619420 |
| NCT04629495 | No results posted | https://clinicaltrials.gov/study/NCT04629495 |
| NCT04629547 | No results posted | https://clinicaltrials.gov/study/NCT04629547 |
| NCT04639050 | No results posted | https://clinicaltrials.gov/study/NCT04639050 |
| NCT04669028 | No results posted | https://clinicaltrials.gov/study/NCT04669028 |
| NCT04685590 | No results posted | https://clinicaltrials.gov/study/NCT04685590 |
| NCT04770220 | No results posted | https://clinicaltrials.gov/study/NCT04770220 |
| NCT04777396 | No results posted | https://clinicaltrials.gov/study/NCT04777396 |
| NCT04777409 | No results posted | https://clinicaltrials.gov/study/NCT04777409 |
| NCT04780399 | No results posted | https://clinicaltrials.gov/study/NCT04780399 |
| NCT04804241 | No results posted | https://clinicaltrials.gov/study/NCT04804241 |
| NCT04838301 | No results posted | https://clinicaltrials.gov/study/NCT04838301 |
| NCT04867616 | No results posted | https://clinicaltrials.gov/study/NCT04867616 |
| NCT04902703 | No results posted | https://clinicaltrials.gov/study/NCT04902703 |
| NCT04931459 | No results posted | https://clinicaltrials.gov/study/NCT04931459 |
| NCT04971733 | No results posted | https://clinicaltrials.gov/study/NCT04971733 |
| NCT04994483 | No results posted | https://clinicaltrials.gov/study/NCT04994483 |
| NCT05026177 | No results posted | https://clinicaltrials.gov/study/NCT05026177 |
| NCT05026866 | No results posted | https://clinicaltrials.gov/study/NCT05026866 |
| NCT05063851 | No results posted | https://clinicaltrials.gov/study/NCT05063851 |
| NCT05068830 | No results posted | https://clinicaltrials.gov/study/NCT05068830 |
| NCT05081219 | No results posted | https://clinicaltrials.gov/study/NCT05081219 |
| NCT05104463 | No results posted | https://clinicaltrials.gov/study/NCT05104463 |
| NCT05143528 | No results posted | https://clinicaltrials.gov/study/NCT05143528 |
| NCT05189210 | No results posted | https://clinicaltrials.gov/study/NCT05189210 |
| NCT05239390 | No results posted | https://clinicaltrials.gov/study/NCT05239390 |
| NCT05267535 | No results posted | https://clinicaltrials.gov/study/NCT05267535 |
| NCT05269173 | No results posted | https://clinicaltrials.gov/study/NCT05269173 |
| NCT05269394 | No results posted | https://clinicaltrials.gov/study/NCT05269394 |
| NCT05282550 | No results posted | https://clinicaltrials.gov/study/NCT05282550 |
| NCT05291234 | No results posted | https://clinicaltrials.gov/study/NCT05291234 |
| NCT05303701 | No results posted | https://clinicaltrials.gov/study/NCT05303701 |
| NCT05307692 | No results posted | https://clinicaltrials.gov/study/NCT05307692 |
| NCT05310071 | No results posted | https://clinicaltrials.gov/study/NCT05310071 |
| NCT05318976 | No results posted | https://clinicaltrials.gov/study/NCT05318976 |
| NCT05323812 | No results posted | https://clinicaltrials.gov/study/NCT05323812 |
| NCT05397639 | No results posted | https://clinicaltrials.gov/study/NCT05397639 |
| NCT05413655 | No results posted | https://clinicaltrials.gov/study/NCT05413655 |
| NCT05423522 | No results posted | https://clinicaltrials.gov/study/NCT05423522 |
| NCT05468073 | No results posted | https://clinicaltrials.gov/study/NCT05468073 |
| NCT05476783 | No results posted | https://clinicaltrials.gov/study/NCT05476783 |
| NCT05478031 | No results posted | https://clinicaltrials.gov/study/NCT05478031 |
| NCT05508789 | No results posted | https://clinicaltrials.gov/study/NCT05508789 |
| NCT05511363 | No results posted | https://clinicaltrials.gov/study/NCT05511363 |
| NCT05522387 | No results posted | https://clinicaltrials.gov/study/NCT05522387 |
| NCT05531526 | No results posted | https://clinicaltrials.gov/study/NCT05531526 |
| NCT05531656 | No results posted | https://clinicaltrials.gov/study/NCT05531656 |
| NCT05557409 | No results posted | https://clinicaltrials.gov/study/NCT05557409 |
| NCT05564169 | No results posted | https://clinicaltrials.gov/study/NCT05564169 |
| NCT05738486 | No results posted | <https://clinicaltrials.gov/study/NCT05738486> |
| NCT05741060 | No results posted | https://clinicaltrials.gov/study/NCT05741060 |
| NCT05969054 | No results posted | https://clinicaltrials.gov/study/NCT05969054 |
| NCT05986721 | No results posted | https://clinicaltrials.gov/study/NCT05986721 |
| NCT06384573 | No results posted | https://clinicaltrials.gov/study/NCT06384573 |
| ACTRN12617001159347 | Non-Pharmacological Intervention | https://pubmed.ncbi.nlm.nih.gov/34141578/ |
| ACTRN12619000738123 | Non-Pharmacological Intervention | https://www.anzctr.org.au/Trial/Registration/TrialReview.aspx?id=377470&isReview=true |
| NCT00012857 | Non-Pharmacological Intervention | https://clinicaltrials.gov/study/NCT00012857 |
| NCT02120664 | Non-Pharmacological Intervention | https://clinicaltrials.gov/study/NCT02120664 |
| NCT02884492 | Non-Pharmacological Intervention | https://clinicaltrials.gov/study/NCT02884492 |
| NCT03703856 | Non-Pharmacological Intervention | https://clinicaltrials.gov/study/NCT03703856 |
| NCT03977584 | Non-Pharmacological Intervention | https://clinicaltrials.gov/study/NCT03977584 |
| NCT04057807 | Non-Pharmacological Intervention | https://clinicaltrials.gov/study/NCT04057807 |
| NCT04129060 | Non-Pharmacological Intervention | https://clinicaltrials.gov/study/NCT04129060 |
| NCT04308304 | Non-Pharmacological Intervention | https://clinicaltrials.gov/study/NCT04308304 |
| NCT05363293 | Non-Pharmacological Intervention | https://clinicaltrials.gov/study/NCT05363293 |
| ACTRN12608000618358 | Non-sporadic AD | https://www.anzctr.org.au/Trial/Registration/TrialReview.aspx?id=83212&isReview=true |
| ACTRN12609000256279 | Non-sporadic AD | https://www.anzctr.org.au/Trial/Registration/TrialReview.aspx?id=83298&isReview=true |
| NCT00073658 | Non-sporadic AD | https://clinicaltrials.gov/study/NCT00073658 |
| NCT01998841 | Non-sporadic AD | https://clinicaltrials.gov/study/NCT01998841 |
| NCT02008357 | Non-sporadic AD | https://clinicaltrials.gov/study/NCT02008357 |
| NCT03044249 | Non-sporadic AD | https://clinicaltrials.gov/study/NCT03044249 |
| NCT04623242 | Non-sporadic AD | https://clinicaltrials.gov/study/NCT04623242 |
| ACTRN12606000324516 | Not suited for meta-analytic model (not comparable) | https://www.anzctr.org.au/Trial/Registration/TrialReview.aspx?id=1485&isReview=true |
| ACTRN12611001008910 | Not suited for meta-analytic model (not comparable) | <https://www.anzctr.org.au/Trial/Registration/TrialReview.aspx?id=343427&isReview=true> |
| ACTRN12611001200976 | Not suited for meta-analytic model (not comparable) | https://www.anzctr.org.au/Trial/Registration/TrialReview.aspx?id=83952&isReview=true |
| ACTRN12612000869875 | Not suited for meta-analytic model (not comparable) | https://www.anzctr.org.au/Trial/Registration/TrialReview.aspx?id=362812&isReview=true |
| ACTRN12613000034730 | Not suited for meta-analytic model (not comparable) | https://www.anzctr.org.au/Trial/Registration/TrialReview.aspx?id=363372&isReview=true |
| ACTRN12613000465752 | Not suited for meta-analytic model (not comparable) | https://www.anzctr.org.au/Trial/Registration/TrialReview.aspx?id=364100&isReview=true |
| ACTRN12613000967785 | Not suited for meta-analytic model (not comparable) | https://www.anzctr.org.au/Trial/Registration/TrialReview.aspx?id=364510&isReview=true |
| ACTRN12614000411640 | Not suited for meta-analytic model (not comparable) | https://www.anzctr.org.au/Trial/Registration/TrialReview.aspx?id=365697&isReview=true |
| ACTRN12614000619640 | Not suited for meta-analytic model (not comparable) | https://www.anzctr.org.au/Trial/Registration/TrialReview.aspx?id=365015&isReview=true |
| ACTRN12614001313628 | Not suited for meta-analytic model (not comparable) | https://www.anzctr.org.au/Trial/Registration/TrialReview.aspx?id=367562&isReview=true |
| ACTRN12615000291583 | Not suited for meta-analytic model (not comparable) | https://www.anzctr.org.au/Trial/Registration/TrialReview.aspx?id=367629&isReview=true |
| ACTRN12617000214336 | Not suited for meta-analytic model (not comparable) | https://www.anzctr.org.au/Trial/Registration/TrialReview.aspx?id=371713&isReview=true |
| ACTRN12617000371392 | Not suited for meta-analytic model (not comparable) | https://www.anzctr.org.au/Trial/Registration/TrialReview.aspx?id=372483&isReview=true |
| ACTRN12617001531303 | Not suited for meta-analytic model (not comparable) | https://www.anzctr.org.au/Trial/Registration/TrialReview.aspx?id=372513&isReview=true |
| ACTRN12618001993280 | Not suited for meta-analytic model (not comparable) | https://www.anzctr.org.au/Trial/Registration/TrialReview.aspx?id=376527&isReview=true |
| ACTRN12619000574145p | Not suited for meta-analytic model (not comparable) | https://www.anzctr.org.au/Trial/Registration/TrialReview.aspx?id=376885&isReview=true |
| ACTRN12619001513101 | Not suited for meta-analytic model (not comparable) | https://www.anzctr.org.au/Trial/Registration/TrialReview.aspx?id=378583&isReview=true |
| ACTRN12620000506998 | Not suited for meta-analytic model (not comparable) | https://www.anzctr.org.au/Trial/Registration/TrialReview.aspx?id=379492&isReview=true |
| ACTRN12621000121864 | Not suited for meta-analytic model (not comparable) | https://www.anzctr.org.au/Trial/Registration/TrialReview.aspx?id=380075&isReview=true |
| ACTRN12621000726853 | Not suited for meta-analytic model (not comparable) | https://www.anzctr.org.au/Trial/Registration/TrialReview.aspx?id=380685&isReview=true |
| ACTRN12621001621808 | Not suited for meta-analytic model (not comparable) | https://www.anzctr.org.au/Trial/Registration/TrialReview.aspx?id=383030&isReview=true |
| ACTRN12621001621808 | Not suited for meta-analytic model (not comparable) | https://www.anzctr.org.au/Trial/Registration/TrialReview.aspx?id=383030&isReview=true |
| ACTRN12622001576718 | Not suited for meta-analytic model (not comparable) | https://www.anzctr.org.au/Trial/Registration/TrialReview.aspx?id=384726&isReview=true |
| ACTRN12623000204640p | Not suited for meta-analytic model (not comparable) | https://www.anzctr.org.au/Trial/Registration/TrialReview.aspx?id=385327&isReview=true |
| ISRCTN72046462 | Not suited for meta-analytic model (not comparable) | https://www.psychiatrist.com/jcp/lithium-trial-alzheimers-disease-randomized-single/ |
| NA | Not suited for meta-analytic model (not comparable) | https://pubmed.ncbi.nlm.nih.gov/32966585/ |
| NA | Not suited for meta-analytic model (not comparable) | https://onlinelibrary.wiley.com/doi/full/10.1111/j.1440-1819.2007.01729.x |
| NA | Not suited for meta-analytic model (not comparable) | https://journals.sagepub.com/doi/10.1177/0269881116665357?url_ver=Z39.88-2003&rfr_id=ori:rid:crossref.org&rfr_dat=cr_pub%20%200pubmed |
| NA | Not suited for meta-analytic model (not comparable) | https://www.thelancet.com/journals/lancet/article/PIIS0140-6736(11)60830-1/abstract |
| NA | Not suited for meta-analytic model (not comparable) | https://www.thelancet.com/journals/lancet/article/PIIS0140-6736(11)60830-1/fulltext |
| NCT00000172 | Not suited for meta-analytic model (not comparable) | https://clinicaltrials.gov/study/NCT00000172 |
| NCT00000173 | Not suited for meta-analytic model (not comparable) | https://clinicaltrials.gov/study/NCT00000173 |
| NCT00000174 | Not suited for meta-analytic model (not comparable) | https://clinicaltrials.gov/study/NCT00000174 |
| NCT00000177 | Not suited for meta-analytic model (not comparable) | https://clinicaltrials.gov/study/NCT00000177 |
| NCT00000178 | Not suited for meta-analytic model (not comparable) | https://clinicaltrials.gov/study/NCT00000178 |
| NCT00001662 | Not suited for meta-analytic model (not comparable) | https://clinicaltrials.gov/study/NCT00001662 |
| NCT00001933 | Not suited for meta-analytic model (not comparable) | https://clinicaltrials.gov/study/NCT00001933 |
| NCT00006399 | Not suited for meta-analytic model (not comparable) | https://clinicaltrials.gov/study/NCT00006399 |
| NCT00009191 | Not suited for meta-analytic model (not comparable) | https://clinicaltrials.gov/study/NCT00009191 |
| NCT00009217 | Not suited for meta-analytic model (not comparable) | https://clinicaltrials.gov/study/NCT00009217 |
| NCT00018343 | Not suited for meta-analytic model (not comparable) | https://clinicaltrials.gov/study/NCT00018343 |
| NCT00018382 | Not suited for meta-analytic model (not comparable) | https://clinicaltrials.gov/study/NCT00018382 |
| NCT00021723 | Not suited for meta-analytic model (not comparable) | https://clinicaltrials.gov/study/NCT00021723 |
| NCT00024531 | Not suited for meta-analytic model (not comparable) | https://clinicaltrials.gov/study/NCT00024531 |
| NCT00034762 | Not suited for meta-analytic model (not comparable) | https://clinicaltrials.gov/study/NCT00034762 |
| NCT00035204 | Not suited for meta-analytic model (not comparable) | https://clinicaltrials.gov/study/NCT00035204 |
| NCT00036114 | Not suited for meta-analytic model (not comparable) | https://clinicaltrials.gov/study/NCT00036114 |
| NCT00040443 | Not suited for meta-analytic model (not comparable) | https://clinicaltrials.gov/study/NCT00040443 |
| NCT00041678 | Not suited for meta-analytic model (not comparable) | https://clinicaltrials.gov/study/NCT00041678 |
| NCT00042172 | Not suited for meta-analytic model (not comparable) | https://clinicaltrials.gov/study/NCT00042172 |
| NCT00046358 | Not suited for meta-analytic model (not comparable) | https://clinicaltrials.gov/study/NCT00046358 |
| NCT00053599 | Not suited for meta-analytic model (not comparable) | https://clinicaltrials.gov/study/NCT00053599 |
| NCT00056225 | Not suited for meta-analytic model (not comparable) | https://clinicaltrials.gov/study/NCT00056225 |
| NCT00063310 | Not suited for meta-analytic model (not comparable) | https://clinicaltrials.gov/study/NCT00063310 |
| NCT00065169 | Not suited for meta-analytic model (not comparable) | https://clinicaltrials.gov/study/NCT00065169 |
| NCT00069849 | Not suited for meta-analytic model (not comparable) | https://clinicaltrials.gov/study/NCT00069849 |
| NCT00071721 | Not suited for meta-analytic model (not comparable) | https://clinicaltrials.gov/study/NCT00071721 |
| NCT00074529 | Not suited for meta-analytic model (not comparable) | https://clinicaltrials.gov/study/NCT00074529 |
| NCT00076440 | Not suited for meta-analytic model (not comparable) | https://clinicaltrials.gov/study/NCT00076440 |
| NCT00082602 | Not suited for meta-analytic model (not comparable) | https://clinicaltrials.gov/study/NCT00082602 |
| NCT00083421 | Not suited for meta-analytic model (not comparable) | https://clinicaltrials.gov/study/NCT00083421 |
| NCT00083590 | Not suited for meta-analytic model (not comparable) | https://clinicaltrials.gov/study/NCT00083590 |
| NCT00087724 | Not suited for meta-analytic model (not comparable) | https://clinicaltrials.gov/study/NCT00087724 |
| NCT00090116 | Not suited for meta-analytic model (not comparable) | https://clinicaltrials.gov/study/NCT00090116 |
| NCT00093951 | Not suited for meta-analytic model (not comparable) | https://clinicaltrials.gov/study/NCT00093951 |
| NCT00096473 | Not suited for meta-analytic model (not comparable) | https://clinicaltrials.gov/study/NCT00096473 |
| NCT00097916 | Not suited for meta-analytic model (not comparable) | https://clinicaltrials.gov/study/NCT00097916 |
| NCT00099242 | Not suited for meta-analytic model (not comparable) | https://clinicaltrials.gov/study/NCT00099242 |
| NCT00099710 | Not suited for meta-analytic model (not comparable) | https://clinicaltrials.gov/study/NCT00099710 |
| NCT00103649 | Not suited for meta-analytic model (not comparable) | https://clinicaltrials.gov/study/NCT00103649 |
| NCT00104013 | Not suited for meta-analytic model (not comparable) | https://clinicaltrials.gov/study/NCT00104013 |
| NCT00104273 | Not suited for meta-analytic model (not comparable) | https://clinicaltrials.gov/study/NCT00104273 |
| NCT00104442 | Not suited for meta-analytic model (not comparable) | https://clinicaltrials.gov/study/NCT00104442 |
| NCT00105105 | Not suited for meta-analytic model (not comparable) | https://clinicaltrials.gov/study/NCT00105105 |
| NCT00105547 | Not suited for meta-analytic model (not comparable) | https://clinicaltrials.gov/study/NCT00105547 |
| NCT00130429 | Not suited for meta-analytic model (not comparable) | https://clinicaltrials.gov/study/NCT00130429 |
| NCT00141661 | Not suited for meta-analytic model (not comparable) | https://clinicaltrials.gov/study/NCT00141661 |
| NCT00142805 | Not suited for meta-analytic model (not comparable) | https://clinicaltrials.gov/study/NCT00142805 |
| NCT00151502 | Not suited for meta-analytic model (not comparable) | https://clinicaltrials.gov/study/NCT00151502 |
| NCT00153010 | Not suited for meta-analytic model (not comparable) | https://clinicaltrials.gov/study/NCT00153010 |
| NCT00160147 | Not suited for meta-analytic model (not comparable) | https://clinicaltrials.gov/study/NCT00160147 |
| NCT00165659 | Not suited for meta-analytic model (not comparable) | https://clinicaltrials.gov/study/NCT00165659 |
| NCT00177671 | Not suited for meta-analytic model (not comparable) | https://clinicaltrials.gov/study/NCT00177671 |
| NCT00205179 | Not suited for meta-analytic model (not comparable) | https://clinicaltrials.gov/study/NCT00205179 |
| NCT00211159 | Not suited for meta-analytic model (not comparable) | https://clinicaltrials.gov/study/NCT00211159 |
| NCT00216502 | Not suited for meta-analytic model (not comparable) | https://clinicaltrials.gov/study/NCT00216502 |
| NCT00216515 | Not suited for meta-analytic model (not comparable) | https://clinicaltrials.gov/study/NCT00216515 |
| NCT00216593 | Not suited for meta-analytic model (not comparable) | https://clinicaltrials.gov/study/NCT00216593 |
| NCT00224497 | Not suited for meta-analytic model (not comparable) | https://clinicaltrials.gov/study/NCT00224497 |
| NCT00230568 | Not suited for meta-analytic model (not comparable) | https://clinicaltrials.gov/study/NCT00230568 |
| NCT00231946 | Not suited for meta-analytic model (not comparable) | https://clinicaltrials.gov/study/NCT00231946 |
| NCT00234637 | Not suited for meta-analytic model (not comparable) | https://clinicaltrials.gov/study/NCT00234637 |
| NCT00235716 | Not suited for meta-analytic model (not comparable) | https://clinicaltrials.gov/study/NCT00235716 |
| NCT00236431 | Not suited for meta-analytic model (not comparable) | https://clinicaltrials.gov/study/NCT00236431 |
| NCT00236574 | Not suited for meta-analytic model (not comparable) | https://clinicaltrials.gov/study/NCT00236574 |
| NCT00240695 | Not suited for meta-analytic model (not comparable) | https://clinicaltrials.gov/study/NCT00240695 |
| NCT00244322 | Not suited for meta-analytic model (not comparable) | https://clinicaltrials.gov/study/NCT00244322 |
| NCT00249145 | Not suited for meta-analytic model (not comparable) | https://clinicaltrials.gov/study/NCT00249145 |
| NCT00249158 | Not suited for meta-analytic model (not comparable) | https://clinicaltrials.gov/study/NCT00249158 |
| NCT00253123 | Not suited for meta-analytic model (not comparable) | https://clinicaltrials.gov/study/NCT00253123 |
| NCT00253188 | Not suited for meta-analytic model (not comparable) | https://clinicaltrials.gov/study/NCT00253188 |
| NCT00253201 | Not suited for meta-analytic model (not comparable) | https://clinicaltrials.gov/study/NCT00253201 |
| NCT00253214 | Not suited for meta-analytic model (not comparable) | https://clinicaltrials.gov/study/NCT00253214 |
| NCT00253227 | Not suited for meta-analytic model (not comparable) | https://clinicaltrials.gov/study/NCT00253227 |
| NCT00254033 | Not suited for meta-analytic model (not comparable) | https://clinicaltrials.gov/study/NCT00254033 |
| NCT00257673 | Not suited for meta-analytic model (not comparable) | https://clinicaltrials.gov/study/NCT00257673 |
| NCT00257712 | Not suited for meta-analytic model (not comparable) | https://clinicaltrials.gov/study/NCT00257712 |
| NCT00261573 | Not suited for meta-analytic model (not comparable) | https://clinicaltrials.gov/study/NCT00261573 |
| NCT00265148 | Not suited for meta-analytic model (not comparable) | https://clinicaltrials.gov/study/NCT00265148 |
| NCT00276510 | Not suited for meta-analytic model (not comparable) | https://clinicaltrials.gov/study/NCT00276510 |
| NCT00285025 | Not suited for meta-analytic model (not comparable) | https://clinicaltrials.gov/study/NCT00285025 |
| NCT00285077 | Not suited for meta-analytic model (not comparable) | https://clinicaltrials.gov/study/NCT00285077 |
| NCT00287742 | Not suited for meta-analytic model (not comparable) | https://clinicaltrials.gov/study/NCT00287742 |
| NCT00301574 | Not suited for meta-analytic model (not comparable) | https://clinicaltrials.gov/study/NCT00301574 |
| NCT00303277 | Not suited for meta-analytic model (not comparable) | https://clinicaltrials.gov/study/NCT00303277 |
| NCT00304629 | Not suited for meta-analytic model (not comparable) | https://clinicaltrials.gov/study/NCT00304629 |
| NCT00309725 | Not suited for meta-analytic model (not comparable) | https://clinicaltrials.gov/study/NCT00309725 |
| NCT00322153 | Not suited for meta-analytic model (not comparable) | https://clinicaltrials.gov/study/NCT00322153 |
| NCT00325728 | Not suited for meta-analytic model (not comparable) | https://clinicaltrials.gov/study/NCT00325728 |
| NCT00334568 | Not suited for meta-analytic model (not comparable) | https://clinicaltrials.gov/study/NCT00334568 |
| NCT00334906 | Not suited for meta-analytic model (not comparable) | https://clinicaltrials.gov/study/NCT00334906 |
| NCT00338117 | Not suited for meta-analytic model (not comparable) | https://clinicaltrials.gov/study/NCT00338117 |
| NCT00348140 | Not suited for meta-analytic model (not comparable) | https://clinicaltrials.gov/study/NCT00348140 |
| NCT00348192 | Not suited for meta-analytic model (not comparable) | https://clinicaltrials.gov/study/NCT00348192 |
| NCT00348309 | Not suited for meta-analytic model (not comparable) | https://clinicaltrials.gov/study/NCT00348309 |
| NCT00357357 | Not suited for meta-analytic model (not comparable) | https://clinicaltrials.gov/study/NCT00357357 |
| NCT00359944 | Not suited for meta-analytic model (not comparable) | https://clinicaltrials.gov/study/NCT00359944 |
| NCT00368459 | Not suited for meta-analytic model (not comparable) | https://clinicaltrials.gov/study/NCT00368459 |
| NCT00377715 | Not suited for meta-analytic model (not comparable) | https://clinicaltrials.gov/study/NCT00377715 |
| NCT00381381 | Not suited for meta-analytic model (not comparable) | https://clinicaltrials.gov/study/NCT00381381 |
| NCT00384423 | Not suited for meta-analytic model (not comparable) | https://clinicaltrials.gov/study/NCT00384423 |
| NCT00403520 | Not suited for meta-analytic model (not comparable) | https://clinicaltrials.gov/study/NCT00403520 |
| NCT00417482 | Not suited for meta-analytic model (not comparable) | https://clinicaltrials.gov/study/NCT00417482 |
| NCT00420420 | Not suited for meta-analytic model (not comparable) | https://clinicaltrials.gov/study/NCT00420420 |
| NCT00423085 | Not suited for meta-analytic model (not comparable) | https://clinicaltrials.gov/study/NCT00423085 |
| NCT00423228 | Not suited for meta-analytic model (not comparable) | https://clinicaltrials.gov/study/NCT00423228 |
| NCT00428090 | Not suited for meta-analytic model (not comparable) | https://clinicaltrials.gov/study/NCT00428090 |
| NCT00428389 | Not suited for meta-analytic model (not comparable) | https://clinicaltrials.gov/study/NCT00428389 |
| NCT00432081 | Not suited for meta-analytic model (not comparable) | https://clinicaltrials.gov/study/NCT00432081 |
| NCT00438568 | Not suited for meta-analytic model (not comparable) | https://clinicaltrials.gov/study/NCT00438568 |
| NCT00439166 | Not suited for meta-analytic model (not comparable) | https://clinicaltrials.gov/study/NCT00439166 |
| NCT00440050 | Not suited for meta-analytic model (not comparable) | https://clinicaltrials.gov/study/NCT00440050 |
| NCT00443417 | Not suited for meta-analytic model (not comparable) | https://clinicaltrials.gov/study/NCT00443417 |
| NCT00454870 | Not suited for meta-analytic model (not comparable) | https://clinicaltrials.gov/study/NCT00454870 |
| NCT00469456 | Not suited for meta-analytic model (not comparable) | https://clinicaltrials.gov/study/NCT00469456 |
| NCT00470418 | Not suited for meta-analytic model (not comparable) | https://clinicaltrials.gov/study/NCT00470418 |
| NCT00471211 | Not suited for meta-analytic model (not comparable) | https://clinicaltrials.gov/study/NCT00471211 |
| NCT00476008 | Not suited for meta-analytic model (not comparable) | https://clinicaltrials.gov/study/NCT00476008 |
| NCT00477659 | Not suited for meta-analytic model (not comparable) | https://clinicaltrials.gov/study/NCT00477659 |
| NCT00478205 | Not suited for meta-analytic model (not comparable) | https://clinicaltrials.gov/study/NCT00478205 |
| NCT00479557 | Not suited for meta-analytic model (not comparable) | https://clinicaltrials.gov/study/NCT00479557 |
| NCT00480870 | Not suited for meta-analytic model (not comparable) | https://clinicaltrials.gov/study/NCT00480870 |
| NCT00481520 | Not suited for meta-analytic model (not comparable) | https://clinicaltrials.gov/study/NCT00481520 |
| NCT00483028 | Not suited for meta-analytic model (not comparable) | https://clinicaltrials.gov/study/NCT00483028 |
| NCT00486044 | Not suited for meta-analytic model (not comparable) | https://clinicaltrials.gov/study/NCT00486044 |
| NCT00488670 | Not suited for meta-analytic model (not comparable) | https://clinicaltrials.gov/study/NCT00488670 |
| NCT00495820 | Not suited for meta-analytic model (not comparable) | https://clinicaltrials.gov/study/NCT00495820 |
| NCT00498602 | Not suited for meta-analytic model (not comparable) | https://clinicaltrials.gov/study/NCT00498602 |
| NCT00500500 | Not suited for meta-analytic model (not comparable) | https://clinicaltrials.gov/study/NCT00500500 |
| NCT00501111 | Not suited for meta-analytic model (not comparable) | https://clinicaltrials.gov/study/NCT00501111 |
| NCT00505167 | Not suited for meta-analytic model (not comparable) | https://clinicaltrials.gov/study/NCT00505167 |
| NCT00506415 | Not suited for meta-analytic model (not comparable) | https://clinicaltrials.gov/study/NCT00506415 |
| NCT00515333 | Not suited for meta-analytic model (not comparable) | https://clinicaltrials.gov/study/NCT00515333 |
| NCT00539305 | Not suited for meta-analytic model (not comparable) | https://clinicaltrials.gov/study/NCT00539305 |
| NCT00548145 | Not suited for meta-analytic model (not comparable) | https://clinicaltrials.gov/study/NCT00548145 |
| NCT00550420 | Not suited for meta-analytic model (not comparable) | https://clinicaltrials.gov/study/NCT00550420 |
| NCT00555204 | Not suited for meta-analytic model (not comparable) | https://clinicaltrials.gov/study/NCT00555204 |
| NCT00566397 | Not suited for meta-analytic model (not comparable) | https://clinicaltrials.gov/study/NCT00566397 |
| NCT00568776 | Not suited for meta-analytic model (not comparable) | https://clinicaltrials.gov/study/NCT00568776 |
| NCT00582855 | Not suited for meta-analytic model (not comparable) | https://clinicaltrials.gov/study/NCT00582855 |
| NCT00594568 | Not suited for meta-analytic model (not comparable) | https://clinicaltrials.gov/study/NCT00594568 |
| NCT00596024 | Not suited for meta-analytic model (not comparable) | https://clinicaltrials.gov/study/NCT00596024 |
| NCT00608946 | Not suited for meta-analytic model (not comparable) | https://clinicaltrials.gov/study/NCT00608946 |
| NCT00620191 | Not suited for meta-analytic model (not comparable) | https://clinicaltrials.gov/study/NCT00620191 |
| NCT00621647 | Not suited for meta-analytic model (not comparable) | https://clinicaltrials.gov/study/NCT00621647 |
| NCT00624026 | Not suited for meta-analytic model (not comparable) | https://clinicaltrials.gov/study/NCT00624026 |
| NCT00627848 | Not suited for meta-analytic model (not comparable) | https://clinicaltrials.gov/study/NCT00627848 |
| NCT00628017 | Not suited for meta-analytic model (not comparable) | https://clinicaltrials.gov/study/NCT00628017 |
| NCT00630851 | Not suited for meta-analytic model (not comparable) | https://clinicaltrials.gov/study/NCT00630851 |
| NCT00645190 | Not suited for meta-analytic model (not comparable) | https://clinicaltrials.gov/study/NCT00645190 |
| NCT00663936 | Not suited for meta-analytic model (not comparable) | https://clinicaltrials.gov/study/NCT00663936 |
| NCT00672945 | Not suited for meta-analytic model (not comparable) | https://clinicaltrials.gov/study/NCT00672945 |
| NCT00675623 | Not suited for meta-analytic model (not comparable) | https://clinicaltrials.gov/study/NCT00675623 |
| NCT00678431 | Not suited for meta-analytic model (not comparable) | https://clinicaltrials.gov/study/NCT00678431 |
| NCT00679627 | Not suited for meta-analytic model (not comparable) | https://clinicaltrials.gov/study/NCT00679627 |
| NCT00693004 | Not suited for meta-analytic model (not comparable) | https://clinicaltrials.gov/study/NCT00693004 |
| NCT00702780 | Not suited for meta-analytic model (not comparable) | https://clinicaltrials.gov/study/NCT00702780 |
| NCT00708552 | Not suited for meta-analytic model (not comparable) | https://clinicaltrials.gov/study/NCT00708552 |
| NCT00710684 | Not suited for meta-analytic model (not comparable) | https://clinicaltrials.gov/study/NCT00710684 |
| NCT00742417 | Not suited for meta-analytic model (not comparable) | https://clinicaltrials.gov/study/NCT00742417 |
| NCT00744978 | Not suited for meta-analytic model (not comparable) | https://clinicaltrials.gov/study/NCT00744978 |
| NCT00751907 | Not suited for meta-analytic model (not comparable) | https://clinicaltrials.gov/study/NCT00751907 |
| NCT00752232 | Not suited for meta-analytic model (not comparable) | https://clinicaltrials.gov/study/NCT00752232 |
| NCT00762411 | Not suited for meta-analytic model (not comparable) | https://clinicaltrials.gov/study/NCT00762411 |
| NCT00804271 | Not suited for meta-analytic model (not comparable) | https://clinicaltrials.gov/study/NCT00804271 |
| NCT00810147 | Not suited for meta-analytic model (not comparable) | https://clinicaltrials.gov/study/NCT00810147 |
| NCT00812565 | Not suited for meta-analytic model (not comparable) | https://clinicaltrials.gov/study/NCT00812565 |
| NCT00814502 | Not suited for meta-analytic model (not comparable) | https://clinicaltrials.gov/study/NCT00814502 |
| NCT00814801 | Not suited for meta-analytic model (not comparable) | https://clinicaltrials.gov/study/NCT00814801 |
| NCT00818662 | Not suited for meta-analytic model (not comparable) | https://clinicaltrials.gov/study/NCT00818662 |
| NCT00829374 | Not suited for meta-analytic model (not comparable) | https://clinicaltrials.gov/study/NCT00829374 |
| NCT00838110 | Not suited for meta-analytic model (not comparable) | https://clinicaltrials.gov/study/NCT00838110 |
| NCT00842673 | Not suited for meta-analytic model (not comparable) | https://clinicaltrials.gov/study/NCT00842673 |
| NCT00842816 | Not suited for meta-analytic model (not comparable) | https://clinicaltrials.gov/study/NCT00842816 |
| NCT00857233 | Not suited for meta-analytic model (not comparable) | https://clinicaltrials.gov/study/NCT00857233 |
| NCT00857649 | Not suited for meta-analytic model (not comparable) | https://clinicaltrials.gov/study/NCT00857649 |
| NCT00862940 | Not suited for meta-analytic model (not comparable) | https://clinicaltrials.gov/study/NCT00862940 |
| NCT00876863 | Not suited for meta-analytic model (not comparable) | https://clinicaltrials.gov/study/NCT00876863 |
| NCT00880412 | Not suited for meta-analytic model (not comparable) | https://clinicaltrials.gov/study/NCT00880412 |
| NCT00884507 | Not suited for meta-analytic model (not comparable) | https://clinicaltrials.gov/study/NCT00884507 |
| NCT00890890 | Not suited for meta-analytic model (not comparable) | https://clinicaltrials.gov/study/NCT00890890 |
| NCT00895895 | Not suited for meta-analytic model (not comparable) | https://clinicaltrials.gov/study/NCT00895895 |
| NCT00912288 | Not suited for meta-analytic model (not comparable) | https://clinicaltrials.gov/study/NCT00912288 |
| NCT00934050 | Not suited for meta-analytic model (not comparable) | https://clinicaltrials.gov/study/NCT00934050 |
| NCT00937352 | Not suited for meta-analytic model (not comparable) | https://clinicaltrials.gov/study/NCT00937352 |
| NCT00939783 | Not suited for meta-analytic model (not comparable) | https://clinicaltrials.gov/study/NCT00939783 |
| NCT00939822 | Not suited for meta-analytic model (not comparable) | https://clinicaltrials.gov/study/NCT00939822 |
| NCT00940589 | Not suited for meta-analytic model (not comparable) | https://clinicaltrials.gov/study/NCT00940589 |
| NCT00948766 | Not suited for meta-analytic model (not comparable) | https://clinicaltrials.gov/study/NCT00948766 |
| NCT00948909 | Not suited for meta-analytic model (not comparable) | https://clinicaltrials.gov/study/NCT00948909 |
| NCT00951834 | Not suited for meta-analytic model (not comparable) | https://clinicaltrials.gov/study/NCT00951834 |
| NCT00954590 | Not suited for meta-analytic model (not comparable) | https://clinicaltrials.gov/study/NCT00954590 |
| NCT00955409 | Not suited for meta-analytic model (not comparable) | https://clinicaltrials.gov/study/NCT00955409 |
| NCT00956410 | Not suited for meta-analytic model (not comparable) | https://clinicaltrials.gov/study/NCT00956410 |
| NCT00980785 | Not suited for meta-analytic model (not comparable) | https://clinicaltrials.gov/study/NCT00980785 |
| NCT01009255 | Not suited for meta-analytic model (not comparable) | https://clinicaltrials.gov/study/NCT01009255 |
| NCT01018875 | Not suited for meta-analytic model (not comparable) | https://clinicaltrials.gov/study/NCT01018875 |
| NCT01019421 | Not suited for meta-analytic model (not comparable) | https://clinicaltrials.gov/study/NCT01019421 |
| NCT01023425 | Not suited for meta-analytic model (not comparable) | https://clinicaltrials.gov/study/NCT01023425 |
| NCT01023685 | Not suited for meta-analytic model (not comparable) | https://clinicaltrials.gov/study/NCT01023685 |
| NCT01023867 | Not suited for meta-analytic model (not comparable) | https://clinicaltrials.gov/study/NCT01023867 |
| NCT01029132 | Not suited for meta-analytic model (not comparable) | https://clinicaltrials.gov/study/NCT01029132 |
| NCT01035138 | Not suited for meta-analytic model (not comparable) | https://clinicaltrials.gov/study/NCT01035138 |
| NCT01039701 | Not suited for meta-analytic model (not comparable) | https://clinicaltrials.gov/study/NCT01039701 |
| NCT01044758 | Not suited for meta-analytic model (not comparable) | https://clinicaltrials.gov/study/NCT01044758 |
| NCT01047254 | Not suited for meta-analytic model (not comparable) | https://clinicaltrials.gov/study/NCT01047254 |
| NCT01068353 | Not suited for meta-analytic model (not comparable) | https://clinicaltrials.gov/study/NCT01068353 |
| NCT01073228 | Not suited for meta-analytic model (not comparable) | https://clinicaltrials.gov/study/NCT01073228 |
| NCT01082965 | Not suited for meta-analytic model (not comparable) | https://clinicaltrials.gov/study/NCT01082965 |
| NCT01097096 | Not suited for meta-analytic model (not comparable) | https://clinicaltrials.gov/study/NCT01097096 |
| NCT01117181 | Not suited for meta-analytic model (not comparable) | https://clinicaltrials.gov/study/NCT01117181 |
| NCT01117818 | Not suited for meta-analytic model (not comparable) | https://clinicaltrials.gov/study/NCT01117818 |
| NCT01117948 | Not suited for meta-analytic model (not comparable) | https://clinicaltrials.gov/study/NCT01117948 |
| NCT01119638 | Not suited for meta-analytic model (not comparable) | https://clinicaltrials.gov/study/NCT01119638 |
| NCT01137526 | Not suited for meta-analytic model (not comparable) | https://clinicaltrials.gov/study/NCT01137526 |
| NCT01145482 | Not suited for meta-analytic model (not comparable) | https://clinicaltrials.gov/study/NCT01145482 |
| NCT01172145 | Not suited for meta-analytic model (not comparable) | https://clinicaltrials.gov/study/NCT01172145 |
| NCT01183806 | Not suited for meta-analytic model (not comparable) | https://clinicaltrials.gov/study/NCT01183806 |
| NCT01245530 | Not suited for meta-analytic model (not comparable) | https://clinicaltrials.gov/study/NCT01245530 |
| NCT01249196 | Not suited for meta-analytic model (not comparable) | https://clinicaltrials.gov/study/NCT01249196 |
| NCT01254773 | Not suited for meta-analytic model (not comparable) | https://clinicaltrials.gov/study/NCT01254773 |
| NCT01266525 | Not suited for meta-analytic model (not comparable) | https://clinicaltrials.gov/study/NCT01266525 |
| NCT01276353 | Not suited for meta-analytic model (not comparable) | https://clinicaltrials.gov/study/NCT01276353 |
| NCT01303744 | Not suited for meta-analytic model (not comparable) | https://clinicaltrials.gov/study/NCT01303744 |
| NCT01320527 | Not suited for meta-analytic model (not comparable) | https://clinicaltrials.gov/study/NCT01320527 |
| NCT01324518 | Not suited for meta-analytic model (not comparable) | https://clinicaltrials.gov/study/NCT01324518 |
| NCT01350362 | Not suited for meta-analytic model (not comparable) | https://clinicaltrials.gov/study/NCT01350362 |
| NCT01354691 | Not suited for meta-analytic model (not comparable) | https://clinicaltrials.gov/study/NCT01354691 |
| NCT01362686 | Not suited for meta-analytic model (not comparable) | https://clinicaltrials.gov/study/NCT01362686 |
| NCT01374438 | Not suited for meta-analytic model (not comparable) | https://clinicaltrials.gov/study/NCT01374438 |
| NCT01380288 | Not suited for meta-analytic model (not comparable) | https://clinicaltrials.gov/study/NCT01380288 |
| NCT01399125 | Not suited for meta-analytic model (not comparable) | https://clinicaltrials.gov/study/NCT01399125 |
| NCT01409564 | Not suited for meta-analytic model (not comparable) | https://clinicaltrials.gov/study/NCT01409564 |
| NCT01409694 | Not suited for meta-analytic model (not comparable) | https://clinicaltrials.gov/study/NCT01409694 |
| NCT01409915 | Not suited for meta-analytic model (not comparable) | https://clinicaltrials.gov/study/NCT01409915 |
| NCT01428453 | Not suited for meta-analytic model (not comparable) | https://clinicaltrials.gov/study/NCT01428453 |
| NCT01429623 | Not suited for meta-analytic model (not comparable) | https://clinicaltrials.gov/study/NCT01429623 |
| NCT01436045 | Not suited for meta-analytic model (not comparable) | https://clinicaltrials.gov/study/NCT01436045 |
| NCT01438060 | Not suited for meta-analytic model (not comparable) | https://clinicaltrials.gov/study/NCT01438060 |
| NCT01439555 | Not suited for meta-analytic model (not comparable) | https://clinicaltrials.gov/study/NCT01439555 |
| NCT01453569 | Not suited for meta-analytic model (not comparable) | https://clinicaltrials.gov/study/NCT01453569 |
| NCT01463384 | Not suited for meta-analytic model (not comparable) | https://clinicaltrials.gov/study/NCT01463384 |
| NCT01466088 | Not suited for meta-analytic model (not comparable) | https://clinicaltrials.gov/study/NCT01466088 |
| NCT01469351 | Not suited for meta-analytic model (not comparable) | https://clinicaltrials.gov/study/NCT01469351 |
| NCT01504854 | Not suited for meta-analytic model (not comparable) | https://clinicaltrials.gov/study/NCT01504854 |
| NCT01524887 | Not suited for meta-analytic model (not comparable) | https://clinicaltrials.gov/study/NCT01524887 |
| NCT01527916 | Not suited for meta-analytic model (not comparable) | https://clinicaltrials.gov/study/NCT01527916 |
| NCT01547169 | Not suited for meta-analytic model (not comparable) | https://clinicaltrials.gov/study/NCT01547169 |
| NCT01548287 | Not suited for meta-analytic model (not comparable) | https://clinicaltrials.gov/study/NCT01548287 |
| NCT01549834 | Not suited for meta-analytic model (not comparable) | https://clinicaltrials.gov/study/NCT01549834 |
| NCT01554683 | Not suited for meta-analytic model (not comparable) | https://clinicaltrials.gov/study/NCT01554683 |
| NCT01561053 | Not suited for meta-analytic model (not comparable) | https://clinicaltrials.gov/study/NCT01561053 |
| NCT01594346 | Not suited for meta-analytic model (not comparable) | https://clinicaltrials.gov/study/NCT01594346 |
| NCT01595646 | Not suited for meta-analytic model (not comparable) | https://clinicaltrials.gov/study/NCT01595646 |
| NCT01608217 | Not suited for meta-analytic model (not comparable) | https://clinicaltrials.gov/study/NCT01608217 |
| NCT01609348 | Not suited for meta-analytic model (not comparable) | https://clinicaltrials.gov/study/NCT01609348 |
| NCT01661673 | Not suited for meta-analytic model (not comparable) | https://clinicaltrials.gov/study/NCT01661673 |
| NCT01676935 | Not suited for meta-analytic model (not comparable) | https://clinicaltrials.gov/study/NCT01676935 |
| NCT01677754 | Not suited for meta-analytic model (not comparable) | https://clinicaltrials.gov/study/NCT01677754 |
| NCT01690195 | Not suited for meta-analytic model (not comparable) | https://clinicaltrials.gov/study/NCT01690195 |
| NCT01696123 | Not suited for meta-analytic model (not comparable) | https://clinicaltrials.gov/study/NCT01696123 |
| NCT01712074 | Not suited for meta-analytic model (not comparable) | https://clinicaltrials.gov/study/NCT01712074 |
| NCT01715350 | Not suited for meta-analytic model (not comparable) | https://clinicaltrials.gov/study/NCT01715350 |
| NCT01735630 | Not suited for meta-analytic model (not comparable) | https://clinicaltrials.gov/study/NCT01735630 |
| NCT01739348 | Not suited for meta-analytic model (not comparable) | https://clinicaltrials.gov/study/NCT01739348 |
| NCT01741194 | Not suited for meta-analytic model (not comparable) | https://clinicaltrials.gov/study/NCT01741194 |
| NCT01764243 | Not suited for meta-analytic model (not comparable) | https://clinicaltrials.gov/study/NCT01764243 |
| NCT01766336 | Not suited for meta-analytic model (not comparable) | https://clinicaltrials.gov/study/NCT01766336 |
| NCT01767909 | Not suited for meta-analytic model (not comparable) | https://clinicaltrials.gov/study/NCT01767909 |
| NCT01811381 | Not suited for meta-analytic model (not comparable) | https://clinicaltrials.gov/study/NCT01811381 |
| NCT01843075 | Not suited for meta-analytic model (not comparable) | https://clinicaltrials.gov/study/NCT01843075 |
| NCT01852110 | Not suited for meta-analytic model (not comparable) | https://clinicaltrials.gov/study/NCT01852110 |
| NCT01872598 | Not suited for meta-analytic model (not comparable) | https://clinicaltrials.gov/study/NCT01872598 |
| NCT01921972 | Not suited for meta-analytic model (not comparable) | https://clinicaltrials.gov/study/NCT01921972 |
| NCT01931566 | Not suited for meta-analytic model (not comparable) | https://clinicaltrials.gov/study/NCT01931566 |
| NCT01953601 | Not suited for meta-analytic model (not comparable) | https://clinicaltrials.gov/study/NCT01953601 |
| NCT01955161 | Not suited for meta-analytic model (not comparable) | https://clinicaltrials.gov/study/NCT01955161 |
| NCT01965756 | Not suited for meta-analytic model (not comparable) | https://clinicaltrials.gov/study/NCT01965756 |
| NCT02006641 | Not suited for meta-analytic model (not comparable) | https://clinicaltrials.gov/study/NCT02006641 |
| NCT02006654 | Not suited for meta-analytic model (not comparable) | https://clinicaltrials.gov/study/NCT02006654 |
| NCT02008513 | Not suited for meta-analytic model (not comparable) | https://clinicaltrials.gov/study/NCT02008513 |
| NCT02017340 | Not suited for meta-analytic model (not comparable) | https://clinicaltrials.gov/study/NCT02017340 |
| NCT02033941 | Not suited for meta-analytic model (not comparable) | https://clinicaltrials.gov/study/NCT02033941 |
| NCT02035553 | Not suited for meta-analytic model (not comparable) | https://clinicaltrials.gov/study/NCT02035553 |
| NCT02063308 | Not suited for meta-analytic model (not comparable) | https://clinicaltrials.gov/study/NCT02063308 |
| NCT02064920 | Not suited for meta-analytic model (not comparable) | https://clinicaltrials.gov/study/NCT02064920 |
| NCT02079909 | Not suited for meta-analytic model (not comparable) | https://clinicaltrials.gov/study/NCT02079909 |
| NCT02080364 | Not suited for meta-analytic model (not comparable) | https://clinicaltrials.gov/study/NCT02080364 |
| NCT02087865 | Not suited for meta-analytic model (not comparable) | https://clinicaltrials.gov/study/NCT02087865 |
| NCT02103673 | Not suited for meta-analytic model (not comparable) | https://clinicaltrials.gov/study/NCT02103673 |
| NCT02167256 | Not suited for meta-analytic model (not comparable) | https://clinicaltrials.gov/study/NCT02167256 |
| NCT02185053 | Not suited for meta-analytic model (not comparable) | https://clinicaltrials.gov/study/NCT02185053 |
| NCT02210286 | Not suited for meta-analytic model (not comparable) | https://clinicaltrials.gov/study/NCT02210286 |
| NCT02240693 | Not suited for meta-analytic model (not comparable) | https://clinicaltrials.gov/study/NCT02240693 |
| NCT02245737 | Not suited for meta-analytic model (not comparable) | https://clinicaltrials.gov/study/NCT02245737 |
| NCT02248116 | Not suited for meta-analytic model (not comparable) | https://clinicaltrials.gov/study/NCT02248116 |
| NCT02249351 | Not suited for meta-analytic model (not comparable) | https://clinicaltrials.gov/study/NCT02249351 |
| NCT02260674 | Not suited for meta-analytic model (not comparable) | https://clinicaltrials.gov/study/NCT02260674 |
| NCT02273895 | Not suited for meta-analytic model (not comparable) | https://clinicaltrials.gov/study/NCT02273895 |
| NCT02279511 | Not suited for meta-analytic model (not comparable) | https://clinicaltrials.gov/study/NCT02279511 |
| NCT02284906 | Not suited for meta-analytic model (not comparable) | https://clinicaltrials.gov/study/NCT02284906 |
| NCT02292238 | Not suited for meta-analytic model (not comparable) | https://clinicaltrials.gov/study/NCT02292238 |
| NCT02293915 | Not suited for meta-analytic model (not comparable) | https://clinicaltrials.gov/study/NCT02293915 |
| NCT02322021 | Not suited for meta-analytic model (not comparable) | https://clinicaltrials.gov/study/NCT02322021 |
| NCT02337907 | Not suited for meta-analytic model (not comparable) | https://clinicaltrials.gov/study/NCT02337907 |
| NCT02346201 | Not suited for meta-analytic model (not comparable) | https://clinicaltrials.gov/study/NCT02346201 |
| NCT02351882 | Not suited for meta-analytic model (not comparable) | https://clinicaltrials.gov/study/NCT02351882 |
| NCT02359552 | Not suited for meta-analytic model (not comparable) | https://clinicaltrials.gov/study/NCT02359552 |
| NCT02361424 | Not suited for meta-analytic model (not comparable) | https://clinicaltrials.gov/study/NCT02361424 |
| NCT02389413 | Not suited for meta-analytic model (not comparable) | https://clinicaltrials.gov/study/NCT02389413 |
| NCT02406027 | Not suited for meta-analytic model (not comparable) | https://clinicaltrials.gov/study/NCT02406027 |
| NCT02431468 | Not suited for meta-analytic model (not comparable) | https://clinicaltrials.gov/study/NCT02431468 |
| NCT02434666 | Not suited for meta-analytic model (not comparable) | https://clinicaltrials.gov/study/NCT02434666 |
| NCT02503501 | Not suited for meta-analytic model (not comparable) | https://clinicaltrials.gov/study/NCT02503501 |
| NCT02551809 | Not suited for meta-analytic model (not comparable) | https://clinicaltrials.gov/study/NCT02551809 |
| NCT02565511 | Not suited for meta-analytic model (not comparable) | https://clinicaltrials.gov/study/NCT02565511 |
| NCT02576639 | Not suited for meta-analytic model (not comparable) | https://clinicaltrials.gov/study/NCT02576639 |
| NCT02579252 | Not suited for meta-analytic model (not comparable) | https://clinicaltrials.gov/study/NCT02579252 |
| NCT02585934 | Not suited for meta-analytic model (not comparable) | https://clinicaltrials.gov/study/NCT02585934 |
| NCT02615002 | Not suited for meta-analytic model (not comparable) | https://clinicaltrials.gov/study/NCT02615002 |
| NCT02626572 | Not suited for meta-analytic model (not comparable) | https://clinicaltrials.gov/study/NCT02626572 |
| NCT02646982 | Not suited for meta-analytic model (not comparable) | https://clinicaltrials.gov/study/NCT02646982 |
| NCT02702817 | Not suited for meta-analytic model (not comparable) | https://clinicaltrials.gov/study/NCT02702817 |
| NCT02709356 | Not suited for meta-analytic model (not comparable) | https://clinicaltrials.gov/study/NCT02709356 |
| NCT02720445 | Not suited for meta-analytic model (not comparable) | https://clinicaltrials.gov/study/NCT02720445 |
| NCT02727699 | Not suited for meta-analytic model (not comparable) | https://clinicaltrials.gov/study/NCT02727699 |
| NCT02750306 | Not suited for meta-analytic model (not comparable) | https://clinicaltrials.gov/study/NCT02750306 |
| NCT02756858 | Not suited for meta-analytic model (not comparable) | https://clinicaltrials.gov/study/NCT02756858 |
| NCT02783573 | Not suited for meta-analytic model (not comparable) | https://clinicaltrials.gov/study/NCT02783573 |
| NCT02788513 | Not suited for meta-analytic model (not comparable) | https://clinicaltrials.gov/study/NCT02788513 |
| NCT02791191 | Not suited for meta-analytic model (not comparable) | https://clinicaltrials.gov/study/NCT02791191 |
| NCT02886494 | Not suited for meta-analytic model (not comparable) | https://clinicaltrials.gov/study/NCT02886494 |
| NCT02910102 | Not suited for meta-analytic model (not comparable) | https://clinicaltrials.gov/study/NCT02910102 |
| NCT02912936 | Not suited for meta-analytic model (not comparable) | https://clinicaltrials.gov/study/NCT02912936 |
| NCT02947893 | Not suited for meta-analytic model (not comparable) | https://clinicaltrials.gov/study/NCT02947893 |
| NCT02956486 | Not suited for meta-analytic model (not comparable) | https://clinicaltrials.gov/study/NCT02956486 |
| NCT02972658 | Not suited for meta-analytic model (not comparable) | https://clinicaltrials.gov/study/NCT02972658 |
| NCT02997982 | Not suited for meta-analytic model (not comparable) | https://clinicaltrials.gov/study/NCT02997982 |
| NCT03001557 | Not suited for meta-analytic model (not comparable) | https://clinicaltrials.gov/study/NCT03001557 |
| NCT03036280 | Not suited for meta-analytic model (not comparable) | https://clinicaltrials.gov/study/NCT03036280 |
| NCT03055741 | Not suited for meta-analytic model (not comparable) | https://clinicaltrials.gov/study/NCT03055741 |
| NCT03061474 | Not suited for meta-analytic model (not comparable) | https://clinicaltrials.gov/study/NCT03061474 |
| NCT03062449 | Not suited for meta-analytic model (not comparable) | https://clinicaltrials.gov/study/NCT03062449 |
| NCT03073876 | Not suited for meta-analytic model (not comparable) | https://clinicaltrials.gov/study/NCT03073876 |
| NCT03075241 | Not suited for meta-analytic model (not comparable) | https://clinicaltrials.gov/study/NCT03075241 |
| NCT03094546 | Not suited for meta-analytic model (not comparable) | https://clinicaltrials.gov/study/NCT03094546 |
| NCT03131453 | Not suited for meta-analytic model (not comparable) | https://clinicaltrials.gov/study/NCT03131453 |
| NCT03184467 | Not suited for meta-analytic model (not comparable) | https://clinicaltrials.gov/study/NCT03184467 |
| NCT03250741 | Not suited for meta-analytic model (not comparable) | https://clinicaltrials.gov/study/NCT03250741 |
| NCT03282916 | Not suited for meta-analytic model (not comparable) | https://clinicaltrials.gov/study/NCT03282916 |
| NCT03328676 | Not suited for meta-analytic model (not comparable) | https://clinicaltrials.gov/study/NCT03328676 |
| NCT03402503 | Not suited for meta-analytic model (not comparable) | https://clinicaltrials.gov/study/NCT03402503 |
| NCT03402659 | Not suited for meta-analytic model (not comparable) | https://clinicaltrials.gov/study/NCT03402659 |
| NCT03435861 | Not suited for meta-analytic model (not comparable) | https://clinicaltrials.gov/study/NCT03435861 |
| NCT03461276 | Not suited for meta-analytic model (not comparable) | https://clinicaltrials.gov/study/NCT03461276 |
| NCT03462121 | Not suited for meta-analytic model (not comparable) | https://clinicaltrials.gov/study/NCT03462121 |
| NCT03507790 | Not suited for meta-analytic model (not comparable) | https://clinicaltrials.gov/study/NCT03507790 |
| NCT03514875 | Not suited for meta-analytic model (not comparable) | https://clinicaltrials.gov/study/NCT03514875 |
| NCT03520998 | Not suited for meta-analytic model (not comparable) | https://clinicaltrials.gov/study/NCT03520998 |
| NCT03531684 | Not suited for meta-analytic model (not comparable) | https://clinicaltrials.gov/study/NCT03531684 |
| NCT03533257 | Not suited for meta-analytic model (not comparable) | https://clinicaltrials.gov/study/NCT03533257 |
| NCT03538522 | Not suited for meta-analytic model (not comparable) | https://clinicaltrials.gov/study/NCT03538522 |
| NCT03560245 | Not suited for meta-analytic model (not comparable) | https://clinicaltrials.gov/study/NCT03560245 |
| NCT03605667 | Not suited for meta-analytic model (not comparable) | https://clinicaltrials.gov/study/NCT03605667 |
| NCT03625622 | Not suited for meta-analytic model (not comparable) | https://clinicaltrials.gov/study/NCT03625622 |
| NCT03639987 | Not suited for meta-analytic model (not comparable) | https://clinicaltrials.gov/study/NCT03639987 |
| NCT03752463 | Not suited for meta-analytic model (not comparable) | https://clinicaltrials.gov/study/NCT03752463 |
| NCT03765762 | Not suited for meta-analytic model (not comparable) | https://clinicaltrials.gov/study/NCT03765762 |
| NCT03790709 | Not suited for meta-analytic model (not comparable) | https://clinicaltrials.gov/study/NCT03790709 |
| NCT03790982 | Not suited for meta-analytic model (not comparable) | https://clinicaltrials.gov/study/NCT03790982 |
| NCT03806478 | Not suited for meta-analytic model (not comparable) | https://clinicaltrials.gov/study/NCT03806478 |
| NCT03824197 | Not suited for meta-analytic model (not comparable) | https://clinicaltrials.gov/study/NCT03824197 |
| NCT03978052 | Not suited for meta-analytic model (not comparable) | https://clinicaltrials.gov/study/NCT03978052 |
| NCT03980730 | Not suited for meta-analytic model (not comparable) | https://clinicaltrials.gov/study/NCT03980730 |
| NCT03991988 | Not suited for meta-analytic model (not comparable) | https://clinicaltrials.gov/study/NCT03991988 |
| NCT04044131 | Not suited for meta-analytic model (not comparable) | https://clinicaltrials.gov/study/NCT04044131 |
| NCT04052737 | Not suited for meta-analytic model (not comparable) | https://clinicaltrials.gov/study/NCT04052737 |
| NCT04079803 | Not suited for meta-analytic model (not comparable) | https://clinicaltrials.gov/study/NCT04079803 |
| NCT04191486 | Not suited for meta-analytic model (not comparable) | https://clinicaltrials.gov/study/NCT04191486 |
| NCT04213391 | Not suited for meta-analytic model (not comparable) | https://clinicaltrials.gov/study/NCT04213391 |
| NCT04251182 | Not suited for meta-analytic model (not comparable) | https://clinicaltrials.gov/study/NCT04251182 |
| NCT04322461 | Not suited for meta-analytic model (not comparable) | https://clinicaltrials.gov/study/NCT04322461 |
| NCT04341467 | Not suited for meta-analytic model (not comparable) | https://clinicaltrials.gov/study/NCT04341467 |
| NCT04388254 | Not suited for meta-analytic model (not comparable) | https://clinicaltrials.gov/study/NCT04388254 |
| NCT04396015 | Not suited for meta-analytic model (not comparable) | https://clinicaltrials.gov/study/NCT04396015 |
| NCT04449926 | Not suited for meta-analytic model (not comparable) | https://clinicaltrials.gov/study/NCT04449926 |
| NCT04491006 | Not suited for meta-analytic model (not comparable) | https://clinicaltrials.gov/study/NCT04491006 |
| NCT04498650 | Not suited for meta-analytic model (not comparable) | https://clinicaltrials.gov/study/NCT04498650 |
| NCT04507126 | Not suited for meta-analytic model (not comparable) | https://clinicaltrials.gov/study/NCT04507126 |
| NCT04538066 | Not suited for meta-analytic model (not comparable) | https://clinicaltrials.gov/study/NCT04538066 |
| NCT04693520 | Not suited for meta-analytic model (not comparable) | https://clinicaltrials.gov/study/NCT04693520 |
| NCT04735536 | Not suited for meta-analytic model (not comparable) | https://clinicaltrials.gov/study/NCT04735536 |
| NCT04797715 | Not suited for meta-analytic model (not comparable) | <https://clinicaltrials.gov/study/NCT04797715> |
| NCT05063539 | Not suited for meta-analytic model (not comparable) | https://clinicaltrials.gov/study/NCT05063539 |
| NCT06045988 | Not suited for meta-analytic model (not comparable) | https://clinicaltrials.gov/study/NCT06045988 |
| NCT06177028 | Not suited for meta-analytic model (not comparable) | https://clinicaltrials.gov/study/NCT06177028 |
| Zhong et al 2007 | Not suited for meta-analytic model (not comparable) | https://www.eurekaselect.com/article/22755 |
| NCT00106899 | Observational study | https://clinicaltrials.gov/study/NCT00106899 |
| NCT01231971 | Observational study | https://classic.clinicaltrials.gov/ct2/show/NCT01231971 |
| NCT02782975 | Pharmacokinetic study | https://clinicaltrials.gov/study/NCT02782975 |
| NCT03711825 | Pharmacokinetic study | https://clinicaltrials.gov/study/NCT03711825 |
| NCT03748303 | Pharmacokinetic study | https://clinicaltrials.gov/study/NCT03748303 |
| NCT04111666 | Pharmacokinetic study | https://clinicaltrials.gov/study/NCT04111666 |
| NCT04451408 | Pharmacokinetic study | https://clinicaltrials.gov/study/NCT04451408 |
| NCT04462029 | Pharmacokinetic study | https://clinicaltrials.gov/study/NCT04462029 |
| NCT04759365 | Pharmacokinetic study | https://clinicaltrials.gov/study/NCT04759365 |
| NCT04920903 | Pharmacokinetic study | https://clinicaltrials.gov/study/NCT04920903 |
| NCT04939792 | Pharmacokinetic study | https://clinicaltrials.gov/study/NCT04939792 |
| NCT05028114 | Pharmacokinetic study | https://clinicaltrials.gov/study/NCT05028114 |
| NCT05040321 | Pharmacokinetic study | https://clinicaltrials.gov/study/NCT05040321 |
| NCT05783830 | Pharmacokinetic study | https://clinicaltrials.gov/study/NCT05783830 |
| NCT05965414 | Pharmacokinetic study | https://clinicaltrials.gov/study/NCT05965414 |
| ACTRN12612000876897 | Phase 1 | https://www.anzctr.org.au/Trial/Registration/TrialReview.aspx?id=362840&isReview=true |
| ACTRN12615000925549 | Phase 1 | https://www.anzctr.org.au/Trial/Registration/TrialReview.aspx?id=369129&isReview=true |
| ACTRN12616000348459 | Phase 1 | https://www.anzctr.org.au/Trial/Registration/TrialReview.aspx?id=370191&isReview=true |
| ACTRN12616000349448 | Phase 1 | https://www.anzctr.org.au/Trial/Registration/TrialReview.aspx?id=369808&isReview=true |
| ACTRN12616000836437 | Phase 1 | https://www.anzctr.org.au/Trial/Registration/TrialReview.aspx?id=370936&isReview=true |
| ACTRN12619000327189 | Phase 1 | https://www.anzctr.org.au/Trial/Registration/TrialReview.aspx?id=375888&isReview=true |
| ACTRN12619001639112 | Phase 1 | https://www.anzctr.org.au/Trial/Registration/TrialReview.aspx?id=378702&isReview=true |
| ACTRN12620000199910 | Phase 1 | https://www.anzctr.org.au/Trial/Registration/TrialReview.aspx?id=378573&isReview=true |
| ACTRN12620000204943p | Phase 1 | https://www.anzctr.org.au/Trial/Registration/TrialReview.aspx?id=379123&isReview=true |
| NA | Phase 1 | https://www.ncbi.nlm.nih.gov/pmc/articles/PMC3715117/ |
| NCT00013650 | Phase 1 | https://clinicaltrials.gov/study/NCT00013650 |
| NCT00017940 | Phase 1 | https://clinicaltrials.gov/study/NCT00017940 |
| NCT00087789 | Phase 1 | https://clinicaltrials.gov/study/NCT00087789 |
| NCT00100282 | Phase 1 | https://clinicaltrials.gov/study/NCT00100282 |
| NCT00117403 | Phase 1 | https://clinicaltrials.gov/study/NCT00117403 |
| NCT00203320 | Phase 1 | https://clinicaltrials.gov/study/NCT00203320 |
| NCT00203359 | Phase 1 | https://clinicaltrials.gov/study/NCT00203359 |
| NCT00366483 | Phase 1 | https://clinicaltrials.gov/study/NCT00366483 |
| NCT00397891 | Phase 1 | https://clinicaltrials.gov/study/NCT00397891 |
| NCT00408525 | Phase 1 | https://clinicaltrials.gov/study/NCT00408525 |
| NCT00411580 | Phase 1 | https://clinicaltrials.gov/study/NCT00411580 |
| NCT00441987 | Phase 1 | https://clinicaltrials.gov/study/NCT00441987 |
| NCT00452504 | Phase 1 | https://clinicaltrials.gov/study/NCT00452504 |
| NCT00455000 | Phase 1 | https://clinicaltrials.gov/study/NCT00455000 |
| NCT00459550 | Phase 1 | https://clinicaltrials.gov/study/NCT00459550 |
| NCT00464334 | Phase 1 | https://clinicaltrials.gov/study/NCT00464334 |
| NCT00468897 | Phase 1 | https://clinicaltrials.gov/study/NCT00468897 |
| NCT00474552 | Phase 1 | https://clinicaltrials.gov/study/NCT00474552 |
| NCT00479219 | Phase 1 | https://clinicaltrials.gov/study/NCT00479219 |
| NCT00479297 | Phase 1 | https://clinicaltrials.gov/study/NCT00479297 |
| NCT00479349 | Phase 1 | https://clinicaltrials.gov/study/NCT00479349 |
| NCT00479440 | Phase 1 | https://clinicaltrials.gov/study/NCT00479440 |
| NCT00479700 | Phase 1 | https://clinicaltrials.gov/study/NCT00479700 |
| NCT00480467 | Phase 1 | https://clinicaltrials.gov/study/NCT00480467 |
| NCT00480818 | Phase 1 | https://clinicaltrials.gov/study/NCT00480818 |
| NCT00494962 | Phase 1 | https://clinicaltrials.gov/study/NCT00494962 |
| NCT00495417 | Phase 1 | https://clinicaltrials.gov/study/NCT00495417 |
| NCT00499200 | Phase 1 | https://clinicaltrials.gov/study/NCT00499200 |
| NCT00499642 | Phase 1 | https://clinicaltrials.gov/study/NCT00499642 |
| NCT00531804 | Phase 1 | https://clinicaltrials.gov/study/NCT00531804 |
| NCT00547560 | Phase 1 | https://clinicaltrials.gov/study/NCT00547560 |
| NCT00551772 | Phase 1 | https://clinicaltrials.gov/study/NCT00551772 |
| NCT00563732 | Phase 1 | https://clinicaltrials.gov/study/NCT00563732 |
| NCT00607308 | Phase 1 | https://clinicaltrials.gov/study/NCT00607308 |
| NCT00621010 | Phase 1 | https://clinicaltrials.gov/study/NCT00621010 |
| NCT00633841 | Phase 1 | https://clinicaltrials.gov/study/NCT00633841 |
| NCT00675090 | Phase 1 | https://clinicaltrials.gov/study/NCT00675090 |
| NCT00684710 | Phase 1 | https://clinicaltrials.gov/study/NCT00684710 |
| NCT00687141 | Phase 1 | https://clinicaltrials.gov/study/NCT00687141 |
| NCT00688207 | Phase 1 | https://clinicaltrials.gov/study/NCT00688207 |
| NCT00689559 | Phase 1 | https://clinicaltrials.gov/study/NCT00689559 |
| NCT00689637 | Phase 1 | https://clinicaltrials.gov/study/NCT00689637 |
| NCT00692510 | Phase 1 | https://clinicaltrials.gov/study/NCT00692510 |
| NCT00713765 | Phase 1 | https://clinicaltrials.gov/study/NCT00713765 |
| NCT00718731 | Phase 1 | https://clinicaltrials.gov/study/NCT00718731 |
| NCT00719394 | Phase 1 | https://clinicaltrials.gov/study/NCT00719394 |
| NCT00726115 | Phase 1 | https://clinicaltrials.gov/study/NCT00726115 |
| NCT00726726 | Phase 1 | https://clinicaltrials.gov/study/NCT00726726 |
| NCT00733642 | Phase 1 | https://clinicaltrials.gov/study/NCT00733642 |
| NCT00733785 | Phase 1 | https://clinicaltrials.gov/study/NCT00733785 |
| NCT00736775 | Phase 1 | https://clinicaltrials.gov/study/NCT00736775 |
| NCT00739037 | Phase 1 | https://clinicaltrials.gov/study/NCT00739037 |
| NCT00745576 | Phase 1 | https://clinicaltrials.gov/study/NCT00745576 |
| NCT00750529 | Phase 1 | https://clinicaltrials.gov/study/NCT00750529 |
| NCT00765115 | Phase 1 | https://clinicaltrials.gov/study/NCT00765115 |
| NCT00766363 | Phase 1 | https://clinicaltrials.gov/study/NCT00766363 |
| NCT00777361 | Phase 1 | https://clinicaltrials.gov/study/NCT00777361 |
| NCT00788047 | Phase 1 | https://clinicaltrials.gov/study/NCT00788047 |
| NCT00795730 | Phase 1 | https://clinicaltrials.gov/study/NCT00795730 |
| NCT00824590 | Phase 1 | https://clinicaltrials.gov/study/NCT00824590 |
| NCT00825084 | Phase 1 | https://clinicaltrials.gov/study/NCT00825084 |
| NCT00827034 | Phase 1 | https://clinicaltrials.gov/study/NCT00827034 |
| NCT00829816 | Phase 1 | https://clinicaltrials.gov/study/NCT00829816 |
| NCT00831506 | Phase 1 | https://clinicaltrials.gov/study/NCT00831506 |
| NCT00838084 | Phase 1 | https://clinicaltrials.gov/study/NCT00838084 |
| NCT00860275 | Phase 1 | https://clinicaltrials.gov/study/NCT00860275 |
| NCT00867399 | Phase 1 | https://clinicaltrials.gov/study/NCT00867399 |
| NCT00874939 | Phase 1 | https://clinicaltrials.gov/study/NCT00874939 |
| NCT00901498 | Phase 1 | https://clinicaltrials.gov/study/NCT00901498 |
| NCT00906191 | Phase 1 | https://clinicaltrials.gov/study/NCT00906191 |
| NCT00931073 | Phase 1 | https://clinicaltrials.gov/study/NCT00931073 |
| NCT00937846 | Phase 1 | https://clinicaltrials.gov/study/NCT00937846 |
| NCT00954252 | Phase 1 | https://clinicaltrials.gov/study/NCT00954252 |
| NCT00959803 | Phase 1 | https://clinicaltrials.gov/study/NCT00959803 |
| NCT00959881 | Phase 1 | https://clinicaltrials.gov/study/NCT00959881 |
| NCT00965588 | Phase 1 | https://clinicaltrials.gov/study/NCT00965588 |
| NCT00966966 | Phase 1 | https://clinicaltrials.gov/study/NCT00966966 |
| NCT00975481 | Phase 1 | https://clinicaltrials.gov/study/NCT00975481 |
| NCT00979316 | Phase 1 | https://clinicaltrials.gov/study/NCT00979316 |
| NCT00988598 | Phase 1 | https://clinicaltrials.gov/study/NCT00988598 |
| NCT00988624 | Phase 1 | https://clinicaltrials.gov/study/NCT00988624 |
| NCT00990613 | Phase 1 | https://clinicaltrials.gov/study/NCT00990613 |
| NCT01002079 | Phase 1 | https://clinicaltrials.gov/study/NCT01002079 |
| NCT01005862 | Phase 1 | https://clinicaltrials.gov/study/NCT01005862 |
| NCT01013610 | Phase 1 | https://clinicaltrials.gov/study/NCT01013610 |
| NCT01028911 | Phase 1 | https://clinicaltrials.gov/study/NCT01028911 |
| NCT01039194 | Phase 1 | https://clinicaltrials.gov/study/NCT01039194 |
| NCT01042314 | Phase 1 | https://clinicaltrials.gov/study/NCT01042314 |
| NCT01057030 | Phase 1 | https://clinicaltrials.gov/study/NCT01057030 |
| NCT01072812 | Phase 1 | https://clinicaltrials.gov/study/NCT01072812 |
| NCT01079819 | Phase 1 | https://clinicaltrials.gov/study/NCT01079819 |
| NCT01093664 | Phase 1 | https://clinicaltrials.gov/study/NCT01093664 |
| NCT01125631 | Phase 1 | https://clinicaltrials.gov/study/NCT01125631 |
| NCT01133405 | Phase 1 | https://clinicaltrials.gov/study/NCT01133405 |
| NCT01137799 | Phase 1 | https://clinicaltrials.gov/study/NCT01137799 |
| NCT01193608 | Phase 1 | https://clinicaltrials.gov/study/NCT01193608 |
| NCT01203384 | Phase 1 | https://clinicaltrials.gov/study/NCT01203384 |
| NCT01221259 | Phase 1 | https://clinicaltrials.gov/study/NCT01221259 |
| NCT01227252 | Phase 1 | https://clinicaltrials.gov/study/NCT01227252 |
| NCT01230853 | Phase 1 | https://clinicaltrials.gov/study/NCT01230853 |
| NCT01253122 | Phase 1 | https://clinicaltrials.gov/study/NCT01253122 |
| NCT01253499 | Phase 1 | https://clinicaltrials.gov/study/NCT01253499 |
| NCT01254448 | Phase 1 | https://clinicaltrials.gov/study/NCT01254448 |
| NCT01258452 | Phase 1 | https://clinicaltrials.gov/study/NCT01258452 |
| NCT01294540 | Phase 1 | https://clinicaltrials.gov/study/NCT01294540 |
| NCT01297036 | Phase 1 | https://clinicaltrials.gov/study/NCT01297036 |
| NCT01297218 | Phase 1 | https://clinicaltrials.gov/study/NCT01297218 |
| NCT01309763 | Phase 1 | https://clinicaltrials.gov/study/NCT01309763 |
| NCT01348737 | Phase 1 | https://clinicaltrials.gov/study/NCT01348737 |
| NCT01369225 | Phase 1 | https://clinicaltrials.gov/study/NCT01369225 |
| NCT01397539 | Phase 1 | https://clinicaltrials.gov/study/NCT01397539 |
| NCT01406145 | Phase 1 | https://clinicaltrials.gov/study/NCT01406145 |
| NCT01424436 | Phase 1 | https://clinicaltrials.gov/study/NCT01424436 |
| NCT01454115 | Phase 1 | https://clinicaltrials.gov/study/NCT01454115 |
| NCT01467726 | Phase 1 | https://clinicaltrials.gov/study/NCT01467726 |
| NCT01482013 | Phase 1 | https://clinicaltrials.gov/study/NCT01482013 |
| NCT01482845 | Phase 1 | https://clinicaltrials.gov/study/NCT01482845 |
| NCT01485302 | Phase 1 | https://clinicaltrials.gov/study/NCT01485302 |
| NCT01487395 | Phase 1 | https://clinicaltrials.gov/study/NCT01487395 |
| NCT01492374 | Phase 1 | https://clinicaltrials.gov/study/NCT01492374 |
| NCT01496170 | Phase 1 | https://clinicaltrials.gov/study/NCT01496170 |
| NCT01537757 | Phase 1 | https://clinicaltrials.gov/study/NCT01537757 |
| NCT01548430 | Phase 1 | https://clinicaltrials.gov/study/NCT01548430 |
| NCT01600859 | Phase 1 | https://clinicaltrials.gov/study/NCT01600859 |
| NCT01656525 | Phase 1 | https://clinicaltrials.gov/study/NCT01656525 |
| NCT01677572 | Phase 1 | https://clinicaltrials.gov/study/NCT01677572 |
| NCT01701089 | Phase 1 | https://clinicaltrials.gov/study/NCT01701089 |
| NCT01702467 | Phase 1 | https://clinicaltrials.gov/study/NCT01702467 |
| NCT01702480 | Phase 1 | https://clinicaltrials.gov/study/NCT01702480 |
| NCT01716637 | Phase 1 | https://clinicaltrials.gov/study/NCT01716637 |
| NCT01716897 | Phase 1 | https://clinicaltrials.gov/study/NCT01716897 |
| NCT01747213 | Phase 1 | https://clinicaltrials.gov/study/NCT01747213 |
| NCT01780519 | Phase 1 | https://clinicaltrials.gov/study/NCT01780519 |
| NCT01795339 | Phase 1 | https://clinicaltrials.gov/study/NCT01795339 |
| NCT01807026 | Phase 1 | https://clinicaltrials.gov/study/NCT01807026 |
| NCT01827982 | Phase 1 | https://clinicaltrials.gov/study/NCT01827982 |
| NCT01837641 | Phase 1 | https://clinicaltrials.gov/study/NCT01837641 |
| NCT01850238 | Phase 1 | https://clinicaltrials.gov/study/NCT01850238 |
| NCT01860625 | Phase 1 | https://clinicaltrials.gov/study/NCT01860625 |
| NCT01864655 | Phase 1 | https://clinicaltrials.gov/study/NCT01864655 |
| NCT01887535 | Phase 1 | https://clinicaltrials.gov/study/NCT01887535 |
| NCT01908010 | Phase 1 | https://clinicaltrials.gov/study/NCT01908010 |
| NCT01966666 | Phase 1 | https://clinicaltrials.gov/study/NCT01966666 |
| NCT01978327 | Phase 1 | https://clinicaltrials.gov/study/NCT01978327 |
| NCT01978548 | Phase 1 | https://clinicaltrials.gov/study/NCT01978548 |
| NCT02005991 | Phase 1 | https://clinicaltrials.gov/study/NCT02005991 |
| NCT02031198 | Phase 1 | https://clinicaltrials.gov/study/NCT02031198 |
| NCT02036645 | Phase 1 | https://clinicaltrials.gov/study/NCT02036645 |
| NCT02040987 | Phase 1 | https://clinicaltrials.gov/study/NCT02040987 |
| NCT02051335 | Phase 1 | https://clinicaltrials.gov/study/NCT02051335 |
| NCT02061878 | Phase 1 | https://clinicaltrials.gov/study/NCT02061878 |
| NCT02094729 | Phase 1 | https://clinicaltrials.gov/study/NCT02094729 |
| NCT02126514 | Phase 1 | https://clinicaltrials.gov/study/NCT02126514 |
| NCT02127476 | Phase 1 | https://clinicaltrials.gov/study/NCT02127476 |
| NCT02142777 | Phase 1 | https://clinicaltrials.gov/study/NCT02142777 |
| NCT02178124 | Phase 1 | https://clinicaltrials.gov/study/NCT02178124 |
| NCT02180269 | Phase 1 | https://clinicaltrials.gov/study/NCT02180269 |
| NCT02197884 | Phase 1 | https://clinicaltrials.gov/study/NCT02197884 |
| NCT02211079 | Phase 1 | https://clinicaltrials.gov/study/NCT02211079 |
| NCT02220738 | Phase 1 | https://clinicaltrials.gov/study/NCT02220738 |
| NCT02221622 | Phase 1 | https://clinicaltrials.gov/study/NCT02221622 |
| NCT02260700 | Phase 1 | https://clinicaltrials.gov/study/NCT02260700 |
| NCT02291783 | Phase 1 | https://clinicaltrials.gov/study/NCT02291783 |
| NCT02323334 | Phase 1 | https://clinicaltrials.gov/study/NCT02323334 |
| NCT02340195 | Phase 1 | https://clinicaltrials.gov/study/NCT02340195 |
| NCT02353598 | Phase 1 | https://clinicaltrials.gov/study/NCT02353598 |
| NCT02360657 | Phase 1 | https://clinicaltrials.gov/study/NCT02360657 |
| NCT02377713 | Phase 1 | https://clinicaltrials.gov/study/NCT02377713 |
| NCT02386306 | Phase 1 | https://clinicaltrials.gov/study/NCT02386306 |
| NCT02388152 | Phase 1 | https://clinicaltrials.gov/study/NCT02388152 |
| NCT02392468 | Phase 1 | https://clinicaltrials.gov/study/NCT02392468 |
| NCT02434718 | Phase 1 | https://clinicaltrials.gov/study/NCT02434718 |
| NCT02462161 | Phase 1 | https://clinicaltrials.gov/study/NCT02462161 |
| NCT02471833 | Phase 1 | https://clinicaltrials.gov/study/NCT02471833 |
| NCT02502253 | Phase 1 | https://clinicaltrials.gov/study/NCT02502253 |
| NCT02509117 | Phase 1 | https://clinicaltrials.gov/study/NCT02509117 |
| NCT02534480 | Phase 1 | https://clinicaltrials.gov/study/NCT02534480 |
| NCT02546310 | Phase 1 | https://clinicaltrials.gov/study/NCT02546310 |
| NCT02570997 | Phase 1 | https://clinicaltrials.gov/study/NCT02570997 |
| NCT02573740 | Phase 1 | https://clinicaltrials.gov/study/NCT02573740 |
| NCT02593318 | Phase 1 | https://clinicaltrials.gov/study/NCT02593318 |
| NCT02600130 | Phase 1 | https://clinicaltrials.gov/study/NCT02600130 |
| NCT02614131 | Phase 1 | https://clinicaltrials.gov/study/NCT02614131 |
| NCT02624778 | Phase 1 | https://clinicaltrials.gov/study/NCT02624778 |
| NCT02648672 | Phase 1 | https://clinicaltrials.gov/study/NCT02648672 |
| NCT02695004 | Phase 1 | https://clinicaltrials.gov/study/NCT02695004 |
| NCT02710188 | Phase 1 | https://clinicaltrials.gov/study/NCT02710188 |
| NCT02754830 | Phase 1 | https://clinicaltrials.gov/study/NCT02754830 |
| NCT02769065 | Phase 1 | https://clinicaltrials.gov/study/NCT02769065 |
| NCT02793232 | Phase 1 | https://clinicaltrials.gov/study/NCT02793232 |
| NCT02820896 | Phase 1 | https://clinicaltrials.gov/study/NCT02820896 |
| NCT02840279 | Phase 1 | https://clinicaltrials.gov/study/NCT02840279 |
| NCT02859207 | Phase 1 | https://clinicaltrials.gov/study/NCT02859207 |
| NCT02910739 | Phase 1 | https://clinicaltrials.gov/study/NCT02910739 |
| NCT02968719 | Phase 1 | https://clinicaltrials.gov/study/NCT02968719 |
| NCT03008161 | Phase 1 | https://clinicaltrials.gov/study/NCT03008161 |
| NCT03019536 | Phase 1 | https://clinicaltrials.gov/study/NCT03019536 |
| NCT03030105 | Phase 1 | https://clinicaltrials.gov/study/NCT03030105 |
| NCT03056495 | Phase 1 | https://clinicaltrials.gov/study/NCT03056495 |
| NCT03056729 | Phase 1 | https://clinicaltrials.gov/study/NCT03056729 |
| NCT03093519 | Phase 1 | https://clinicaltrials.gov/study/NCT03093519 |
| NCT03113812 | Phase 1 | https://clinicaltrials.gov/study/NCT03113812 |
| NCT03179501 | Phase 1 | https://clinicaltrials.gov/study/NCT03179501 |
| NCT03259958 | Phase 1 | https://clinicaltrials.gov/study/NCT03259958 |
| NCT03274817 | Phase 1 | https://clinicaltrials.gov/study/NCT03274817 |
| NCT03298672 | Phase 1 | https://clinicaltrials.gov/study/NCT03298672 |
| NCT03307993 | Phase 1 | https://clinicaltrials.gov/study/NCT03307993 |
| NCT03375697 | Phase 1 | https://clinicaltrials.gov/study/NCT03375697 |
| NCT03418688 | Phase 1 | https://clinicaltrials.gov/study/NCT03418688 |
| NCT03432195 | Phase 1 | https://clinicaltrials.gov/study/NCT03432195 |
| NCT03438604 | Phase 1 | https://clinicaltrials.gov/study/NCT03438604 |
| NCT03456349 | Phase 1 | https://clinicaltrials.gov/study/NCT03456349 |
| NCT03522129 | Phase 1 | https://clinicaltrials.gov/study/NCT03522129 |
| NCT03551769 | Phase 1 | https://clinicaltrials.gov/study/NCT03551769 |
| NCT03634007 | Phase 1 | https://clinicaltrials.gov/study/NCT03634007 |
| NCT03635047 | Phase 1 | https://clinicaltrials.gov/study/NCT03635047 |
| NCT03668405 | Phase 1 | https://clinicaltrials.gov/study/NCT03668405 |
| NCT03698695 | Phase 1 | https://clinicaltrials.gov/study/NCT03698695 |
| NCT03706885 | Phase 1 | https://clinicaltrials.gov/study/NCT03706885 |
| NCT03720548 | Phase 1 | https://clinicaltrials.gov/study/NCT03720548 |
| NCT03740178 | Phase 1 | https://clinicaltrials.gov/study/NCT03740178 |
| NCT03752294 | Phase 1 | https://clinicaltrials.gov/study/NCT03752294 |
| NCT03757325 | Phase 1 | https://clinicaltrials.gov/study/NCT03757325 |
| NCT03784300 | Phase 1 | https://clinicaltrials.gov/study/NCT03784300 |
| NCT03802162 | Phase 1 | https://clinicaltrials.gov/study/NCT03802162 |
| NCT03819699 | Phase 1 | https://clinicaltrials.gov/study/NCT03819699 |
| NCT03822208 | Phase 1 | https://clinicaltrials.gov/study/NCT03822208 |
| NCT03838185 | Phase 1 | https://clinicaltrials.gov/study/NCT03838185 |
| NCT03887455 | Phase 1 | https://clinicaltrials.gov/study/NCT03887455 |
| NCT03899298 | Phase 1 | https://clinicaltrials.gov/study/NCT03899298 |
| NCT03935568 | Phase 1 | https://clinicaltrials.gov/study/NCT03935568 |
| NCT03943264 | Phase 1 | https://clinicaltrials.gov/study/NCT03943264 |
| NCT03944460 | Phase 1 | https://clinicaltrials.gov/study/NCT03944460 |
| NCT03955380 | Phase 1 | https://clinicaltrials.gov/study/NCT03955380 |
| NCT03971123 | Phase 1 | https://clinicaltrials.gov/study/NCT03971123 |
| NCT03998423 | Phase 1 | https://clinicaltrials.gov/study/NCT03998423 |
| NCT04023994 | Phase 1 | https://clinicaltrials.gov/study/NCT04023994 |
| NCT04040348 | Phase 1 | https://clinicaltrials.gov/study/NCT04040348 |
| NCT04074837 | Phase 1 | https://clinicaltrials.gov/study/NCT04074837 |
| NCT04133454 | Phase 1 | https://clinicaltrials.gov/study/NCT04133454 |
| NCT04149860 | Phase 1 | https://clinicaltrials.gov/study/NCT04149860 |
| NCT04157712 | Phase 1 | https://clinicaltrials.gov/study/NCT04157712 |
| NCT04208152 | Phase 1 | https://clinicaltrials.gov/study/NCT04208152 |
| NCT04268953 | Phase 1 | https://clinicaltrials.gov/study/NCT04268953 |
| NCT04500847 | Phase 1 | https://clinicaltrials.gov/study/NCT04500847 |
| NCT04570644 | Phase 1 | https://clinicaltrials.gov/study/NCT04570644 |
| NCT04585347 | Phase 1 | https://clinicaltrials.gov/study/NCT04585347 |
| NCT04672135 | Phase 1 | https://clinicaltrials.gov/study/NCT04672135 |
| NCT04711486 | Phase 1 | https://clinicaltrials.gov/study/NCT04711486 |
| NCT04740580 | Phase 1 | https://clinicaltrials.gov/study/NCT04740580 |
| NCT04749563 | Phase 1 | https://clinicaltrials.gov/study/NCT04749563 |
| NCT04805983 | Phase 1 | https://clinicaltrials.gov/study/NCT04805983 |
| NCT04983368 | Phase 1 | https://clinicaltrials.gov/study/NCT04983368 |
| NCT05077501 | Phase 1 | https://clinicaltrials.gov/study/NCT05077501 |
| NCT05077631 | Phase 1 | https://clinicaltrials.gov/study/NCT05077631 |
| NCT05225389 | Phase 1 | https://clinicaltrials.gov/study/NCT05225389 |
| NCT05227118 | Phase 1 | https://clinicaltrials.gov/study/NCT05227118 |
| NCT05231785 | Phase 1 | https://clinicaltrials.gov/study/NCT05231785 |
| NCT05248672 | Phase 1 | https://clinicaltrials.gov/study/NCT05248672 |
| NCT05318040 | Phase 1 | https://clinicaltrials.gov/study/NCT05318040 |
| NCT05328115 | Phase 1 | https://clinicaltrials.gov/study/NCT05328115 |
| NCT05344989 | Phase 1 | https://clinicaltrials.gov/study/NCT05344989 |
| NCT05400330 | Phase 1 | https://clinicaltrials.gov/study/NCT05400330 |
| NCT05408780 | Phase 1 | https://clinicaltrials.gov/study/NCT05408780 |
| NCT05463731 | Phase 1 | https://clinicaltrials.gov/study/NCT05463731 |
| NCT05466422 | Phase 1 | https://clinicaltrials.gov/study/NCT05466422 |
| NCT05469360 | Phase 1 | https://clinicaltrials.gov/study/NCT05469360 |
| NCT05503511 | Phase 1 | https://clinicaltrials.gov/study/NCT05503511 |
| NCT05525780 | Phase 1 | https://clinicaltrials.gov/study/NCT05525780 |
| NCT05532358 | Phase 1 | https://clinicaltrials.gov/study/NCT05532358 |
| NCT05551741 | Phase 1 | https://clinicaltrials.gov/study/NCT05551741 |
| NCT05591027 | Phase 1 | https://clinicaltrials.gov/study/NCT05591027 |
| NCT05628636 | Phase 1 | https://clinicaltrials.gov/study/NCT05628636 |
| NCT05686980 | Phase 1 | https://clinicaltrials.gov/study/NCT05686980 |
| NCT05696483 | Phase 1 | https://clinicaltrials.gov/study/NCT05696483 |
| NCT05787028 | Phase 1 | https://clinicaltrials.gov/study/NCT05787028 |
| NCT05787041 | Phase 1 | https://clinicaltrials.gov/study/NCT05787041 |
| NCT05792163 | Phase 1 | https://clinicaltrials.gov/study/NCT05792163 |
| NCT05804279 | Phase 1 | https://clinicaltrials.gov/study/NCT05804279 |
| NCT05806177 | Phase 1 | https://clinicaltrials.gov/study/NCT05806177 |
| NCT05817643 | Phase 1 | https://clinicaltrials.gov/study/NCT05817643 |
| NCT05827653 | Phase 1 | https://clinicaltrials.gov/study/NCT05827653 |
| NCT05921929 | Phase 1 | https://clinicaltrials.gov/study/NCT05921929 |
| NCT06127368 | Phase 1 | https://clinicaltrials.gov/study/NCT06127368 |
| NCT06194552 | Phase 1 | https://clinicaltrials.gov/study/NCT06194552 |
| NCT06206824 | Phase 1 | https://clinicaltrials.gov/study/NCT06206824 |
| NCT00090402 | Phase 1 and 2 | https://clinicaltrials.gov/study/NCT00090402 |
| NCT00380302 | Phase 1 and 2 | https://clinicaltrials.gov/study/NCT00380302 |
| NCT00391833 | Phase 1 and 2 | https://clinicaltrials.gov/study/NCT00391833 |
| NCT00580931 | Phase 1 and 2 | https://clinicaltrials.gov/study/NCT00580931 |
| NCT00581867 | Phase 1 and 2 | https://clinicaltrials.gov/study/NCT00581867 |
| NCT00592943 | Phase 1 and 2 | https://clinicaltrials.gov/study/NCT00592943 |
| NCT00593138 | Phase 1 and 2 | https://clinicaltrials.gov/study/NCT00593138 |
| NCT00948259 | Phase 1 and 2 | https://clinicaltrials.gov/study/NCT00948259 |
| NCT01058941 | Phase 1 and 2 | https://clinicaltrials.gov/study/NCT01058941 |
| NCT01513967 | Phase 1 and 2 | https://clinicaltrials.gov/study/NCT01513967 |
| NCT01560585 | Phase 1 and 2 | https://clinicaltrials.gov/study/NCT01560585 |
| NCT01561430 | Phase 1 and 2 | https://clinicaltrials.gov/study/NCT01561430 |
| NCT01617577 | Phase 1 and 2 | https://clinicaltrials.gov/study/NCT01617577 |
| NCT01780974 | Phase 1 and 2 | https://clinicaltrials.gov/study/NCT01780974 |
| NCT02054208 | Phase 1 and 2 | https://clinicaltrials.gov/study/NCT02054208 |
| NCT02078310 | Phase 1 and 2 | https://clinicaltrials.gov/study/NCT02078310 |
| NCT02221947 | Phase 1 and 2 | https://clinicaltrials.gov/study/NCT02221947 |
| NCT02541929 | Phase 1 and 2 | https://clinicaltrials.gov/study/NCT02541929 |
| NCT02560753 | Phase 1 and 2 | https://clinicaltrials.gov/study/NCT02560753 |
| NCT02569398 | Phase 1 and 2 | https://clinicaltrials.gov/study/NCT02569398 |
| NCT02707458 | Phase 1 and 2 | https://clinicaltrials.gov/study/NCT02707458 |
| NCT02719327 | Phase 1 and 2 | https://clinicaltrials.gov/study/NCT02719327 |
| NCT02899091 | Phase 1 and 2 | https://clinicaltrials.gov/study/NCT02899091 |
| NCT02907567 | Phase 1 and 2 | https://clinicaltrials.gov/study/NCT02907567 |
| NCT02925650 | Phase 1 and 2 | https://clinicaltrials.gov/study/NCT02925650 |
| NCT03069014 | Phase 1 and 2 | https://clinicaltrials.gov/study/NCT03069014 |
| NCT03101085 | Phase 1 and 2 | https://clinicaltrials.gov/study/NCT03101085 |
| NCT03117738 | Phase 1 and 2 | https://clinicaltrials.gov/study/NCT03117738 |
| NCT03172117 | Phase 1 and 2 | https://clinicaltrials.gov/study/NCT03172117 |
| NCT03186989 | Phase 1 and 2 | https://clinicaltrials.gov/study/NCT03186989 |
| NCT03493282 | Phase 1 and 2 | https://clinicaltrials.gov/study/NCT03493282 |
| NCT03801642 | Phase 1 and 2 | https://clinicaltrials.gov/study/NCT03801642 |
| NCT03865017 | Phase 1 and 2 | https://clinicaltrials.gov/study/NCT03865017 |
| NCT04063124 | Phase 1 and 2 | https://clinicaltrials.gov/study/NCT04063124 |
| NCT04098666 | Phase 1 and 2 | https://clinicaltrials.gov/study/NCT04098666 |
| NCT04228666 | Phase 1 and 2 | https://clinicaltrials.gov/study/NCT04228666 |
| NCT04249869 | Phase 1 and 2 | https://clinicaltrials.gov/study/NCT04249869 |
| NCT04251910 | Phase 1 and 2 | https://clinicaltrials.gov/study/NCT04251910 |
| NCT04381468 | Phase 1 and 2 | https://clinicaltrials.gov/study/NCT04381468 |
| NCT04388982 | Phase 1 and 2 | https://clinicaltrials.gov/study/NCT04388982 |
| NCT04413344 | Phase 1 and 2 | https://clinicaltrials.gov/study/NCT04413344 |
| NCT04445831 | Phase 1 and 2 | https://clinicaltrials.gov/study/NCT04445831 |
| NCT04524351 | Phase 1 and 2 | https://clinicaltrials.gov/study/NCT04524351 |
| NCT04684602 | Phase 1 and 2 | https://clinicaltrials.gov/study/NCT04684602 |
| NCT05074498 | Phase 1 and 2 | https://clinicaltrials.gov/study/NCT05074498 |
| NCT05183516 | Phase 1 and 2 | https://clinicaltrials.gov/study/NCT05183516 |
| NCT05189106 | Phase 1 and 2 | https://clinicaltrials.gov/study/NCT05189106 |
| NCT05345509 | Phase 1 and 2 | https://clinicaltrials.gov/study/NCT05345509 |
| NCT05422885 | Phase 1 and 2 | https://clinicaltrials.gov/study/NCT05422885 |
| NCT05462106 | Phase 1 and 2 | https://clinicaltrials.gov/study/NCT05462106 |
| NCT00015548 | Phase I | https://www.nejm.org/doi/full/10.1056/nejmoa061240 |
| NCT00000176 | Prevention study | https://clinicaltrials.gov/study/NCT00000176 |
| NCT00007189 | Prevention study | https://clinicaltrials.gov/study/NCT00007189 |
| NCT00010803 | Prevention study | https://clinicaltrials.gov/study/NCT00010803 |
| NCT00267163 | Prevention study | https://clinicaltrials.gov/study/NCT00267163 |
| NCT02913664 | Prevention study | https://clinicaltrials.gov/study/NCT02913664 |
| NCT03461861 | Prevention study | https://clinicaltrials.gov/study/NCT03461861 |
| NCT03613844 | Prevention study | https://clinicaltrials.gov/study/NCT03613844 |
| NCT03691519 | Prevention study | https://clinicaltrials.gov/study/NCT03691519 |
| NCT03848312 | Prevention study | https://clinicaltrials.gov/study/NCT03848312 |
| NCT05586750 | Prevention study | https://clinicaltrials.gov/study/NCT05586750 |
| NA | Small number of participants in each arm (N < 20) | https://onlinelibrary.wiley.com/doi/full/10.1111/ggi.12516 |
| NA | Small number of participants in each arm (N < 20) | https://jamanetwork.com/journals/jamapsychiatry/fullarticle/481661 |
| NA | Small number of participants in each arm (N < 20) | https://www.neurology.org/doi/full/10.1212/01.wnl.0000260060.60870.89 |
| NA | Small number of participants in each arm (N < 20) | 3 |
| NA | Small number of participants in each arm (N < 20) | https://pubmed.ncbi.nlm.nih.gov/31182351/ |
| NA | Small number of participants in each arm (N < 20) | www.onlinelibrary.wiley.com/doi/10.1002/gps.636 |
| NCT00088387 | Small number of participants in each arm (N < 20) | https://clinicaltrials.gov/study/NCT00088387 |
| NCT00112073 | Small number of participants in each arm (N < 20) | <https://jamanetwork.com/journals/jamaneurology/fullarticle/1151841> |
| NCT00151333 | Small number of participants in each arm (N < 20) | https://clinicaltrials.gov/study/NCT00151333 |
| NCT00161473 | Small number of participants in each arm (N < 20) | https://clinicaltrials.gov/study/NCT00161473 |
| NCT00164749 | Small number of participants in each arm (N < 20) | https://clinicaltrials.gov/study/NCT00164749 |
| NCT00165750 | Small number of participants in each arm (N < 20) | https://clinicaltrials.gov/study/NCT00165750 |
| NCT00255086 | Small number of participants in each arm (N < 20) | https://clinicaltrials.gov/study/NCT00255086 |
| NCT00260624 | Small number of participants in each arm (N < 20) | https://clinicaltrials.gov/study/NCT00260624 |
| NCT00299988 | Small number of participants in each arm (N < 20) | https://clinicaltrials.gov/study/NCT00299988 |
| NCT00329082 | Small number of participants in each arm (N < 20) | https://clinicaltrials.gov/study/NCT00329082 |
| NCT00362024 | Small number of participants in each arm (N < 20) | https://clinicaltrials.gov/study/NCT00362024 |
| NCT00369603 | Small number of participants in each arm (N < 20) | https://clinicaltrials.gov/study/NCT00369603 |
| NCT00385684 | Small number of participants in each arm (N < 20) | https://clinicaltrials.gov/study/NCT00385684 |
| NCT00392912 | Small number of participants in each arm (N < 20) | https://clinicaltrials.gov/study/NCT00392912 |
| NCT00401167 | Small number of participants in each arm (N < 20) | https://clinicaltrials.gov/study/NCT00401167 |
| NCT00551161 | Small number of participants in each arm (N < 20) | https://clinicaltrials.gov/study/NCT00551161 |
| NCT00586430 | Small number of participants in each arm (N < 20) | https://clinicaltrials.gov/study/NCT00586430 |
| NCT00602680 | Small number of participants in each arm (N < 20) | https://clinicaltrials.gov/study/NCT00602680 |
| NCT00626210 | Small number of participants in each arm (N < 20) | https://clinicaltrials.gov/study/NCT00626210 |
| NCT00649220 | Small number of participants in each arm (N < 20) | https://clinicaltrials.gov/study/NCT00649220 |
| NCT00663026 | Small number of participants in each arm (N < 20) | <https://clinicaltrials.gov/study/NCT00663026> |
| NCT00733863 | Small number of participants in each arm (N < 20) | https://clinicaltrials.gov/study/NCT00733863 |
| NCT00749216 | Small number of participants in each arm (N < 20) | https://clinicaltrials.gov/study/NCT00749216 |
| NCT00795418 | Small number of participants in each arm (N < 20) | https://clinicaltrials.gov/study/NCT00795418 |
| NCT00809510 | Small number of participants in each arm (N < 20) | https://clinicaltrials.gov/study/NCT00809510 |
| NCT00814346 | Small number of participants in each arm (N < 20) | https://clinicaltrials.gov/study/NCT00814346 |
| NCT00945672 | Small number of participants in each arm (N < 20) | https://clinicaltrials.gov/study/NCT00945672 |
| NCT00959192 | Small number of participants in each arm (N < 20) | https://clinicaltrials.gov/study/NCT00959192 |
| NCT00976118 | Small number of participants in each arm (N < 20) | https://clinicaltrials.gov/study/NCT00976118 |
| NCT00982202 | Small number of participants in each arm (N < 20) | https://clinicaltrials.gov/study/NCT00982202 |
| NCT01001637 | Small number of participants in each arm (N < 20) | https://clinicaltrials.gov/study/NCT01001637 |
| NCT01055392 | Small number of participants in each arm (N < 20) | https://www.cambridge.org/core/journals/the-british-journal-of-psychiatry/article/diseasemodifying-properties-of-longterm-lithium-treatment-for-amnestic-mild-cognitive-impairment-randomised-controlled-trial/C673342DBC81E80462318BF208B9A8F7 |
| NCT01066546 | Small number of participants in each arm (N < 20) | https://clinicaltrials.gov/study/NCT01066546 |
| NCT01075763 | Small number of participants in each arm (N < 20) | https://clinicaltrials.gov/study/NCT01075763 |
| NCT01078168 | Small number of participants in each arm (N < 20) | https://clinicaltrials.gov/study/NCT01078168 |
| NCT01125683 | Small number of participants in each arm (N < 20) | https://clinicaltrials.gov/study/NCT01125683 |
| NCT01126099 | Small number of participants in each arm (N < 20) | https://clinicaltrials.gov/study/NCT01126099 |
| NCT01142258 | Small number of participants in each arm (N < 20) | https://clinicaltrials.gov/study/NCT01142258 |
| NCT01142336 | Small number of participants in each arm (N < 20) | https://clinicaltrials.gov/study/NCT01142336 |
| NCT01181921 | Small number of participants in each arm (N < 20) | https://clinicaltrials.gov/study/NCT01181921 |
| NCT01227564 | Small number of participants in each arm (N < 20) | https://clinicaltrials.gov/study/NCT01227564 |
| NCT01238991 | Small number of participants in each arm (N < 20) | https://clinicaltrials.gov/study/NCT01238991 |
| NCT01284387 | Small number of participants in each arm (N < 20) | https://clinicaltrials.gov/study/NCT01284387 |
| NCT01302340 | Small number of participants in each arm (N < 20) | https://www.sciencedirect.com/science/article/abs/pii/S1064748115002250?via%3Dihub |
| NCT01354444 | Small number of participants in each arm (N < 20) | https://clinicaltrials.gov/study/NCT01354444 |
| NCT01388478 | Small number of participants in each arm (N < 20) | https://clinicaltrials.gov/study/NCT01388478 |
| NCT01397578 | Small number of participants in each arm (N < 20) | <https://www.ncbi.nlm.nih.gov/pmc/articles/PMC6146627/> |
| NCT01522404 | Small number of participants in each arm (N < 20) | https://clinicaltrials.gov/study/NCT01522404 |
| NCT01600469 | Small number of participants in each arm (N < 20) | https://clinicaltrials.gov/study/NCT01600469 |
| NCT01626391 | Small number of participants in each arm (N < 20) | https://clinicaltrials.gov/study/NCT01626391 |
| NCT01703117 | Small number of participants in each arm (N < 20) | https://clinicaltrials.gov/study/NCT01703117 |
| NCT01729598 | Small number of participants in each arm (N < 20) | https://clinicaltrials.gov/study/NCT01729598 |
| NCT01736579 | Small number of participants in each arm (N < 20) | https://clinicaltrials.gov/study/NCT01736579 |
| NCT01782742 | Small number of participants in each arm (N < 20) | https://clinicaltrials.gov/study/NCT01782742 |
| NCT01832350 | Small number of participants in each arm (N < 20) | https://clinicaltrials.gov/study/NCT01832350 |
| NCT01928420 | Small number of participants in each arm (N < 20) | https://clinicaltrials.gov/study/NCT01928420 |
| NCT01982578 | Small number of participants in each arm (N < 20) | https://clinicaltrials.gov/study/NCT01982578 |
| NCT02002819 | Small number of participants in each arm (N < 20) | https://clinicaltrials.gov/study/NCT02002819 |
| NCT02129348 | Small number of participants in each arm (N < 20) | https://clinicaltrials.gov/study/NCT02129348 |
| NCT02168920 | Small number of participants in each arm (N < 20) | https://clinicaltrials.gov/study/NCT02168920 |
| NCT02244541 | Small number of participants in each arm (N < 20) | https://clinicaltrials.gov/study/NCT02244541 |
| NCT02423122 | Small number of participants in each arm (N < 20) | https://clinicaltrials.gov/study/NCT02423122 |
| NCT02423200 | Small number of participants in each arm (N < 20) | https://clinicaltrials.gov/study/NCT02423200 |
| NCT02549196 | Small number of participants in each arm (N < 20) | https://clinicaltrials.gov/study/NCT02549196 |
| NCT02760602 | Small number of participants in each arm (N < 20) | https://clinicaltrials.gov/study/NCT02760602 |
| NCT03319810 | Small number of participants in each arm (N < 20) | https://clinicaltrials.gov/study/NCT03319810 |
| NCT03417986 | Small number of participants in each arm (N < 20) | https://clinicaltrials.gov/study/NCT03417986 |
| NCT03531710 | Small number of participants in each arm (N < 20) | https://clinicaltrials.gov/study/NCT03531710 |
| NCT03656042 | Small number of participants in each arm (N < 20) | https://clinicaltrials.gov/study/NCT03656042 |
| NCT03710642 | Small number of participants in each arm (N < 20) | https://clinicaltrials.gov/study/NCT03710642 |
| NCT03748706 | Small number of participants in each arm (N < 20) | https://clinicaltrials.gov/study/NCT03748706 |
| NCT03856359 | Small number of participants in each arm (N < 20) | https://clinicaltrials.gov/study/NCT03856359 |
| NCT03867253 | Small number of participants in each arm (N < 20) | https://clinicaltrials.gov/study/NCT03867253 |
| NCT03959553 | Small number of participants in each arm (N < 20) | https://clinicaltrials.gov/study/NCT03959553 |
| NCT04070378 | Small number of participants in each arm (N < 20) | https://clinicaltrials.gov/study/NCT04070378 |
| NCT04311515 | Small number of participants in each arm (N < 20) | https://clinicaltrials.gov/study/NCT04311515 |
| NCT04795466 | Small number of participants in each arm (N < 20) | https://clinicaltrials.gov/study/NCT04795466 |
| NCT04798989 | Small number of participants in each arm (N < 20) | https://clinicaltrials.gov/study/NCT04798989 |
| NCT05004688 | Small number of participants in each arm (N < 20) | https://clinicaltrials.gov/study/NCT05004688 |
| NCT05006599 | Small number of participants in each arm (N < 20) | https://clinicaltrials.gov/study/NCT05006599 |
| NCT05194163 | Small number of participants in each arm (N < 20) | https://clinicaltrials.gov/study/NCT05194163 |
| NCT05256134 | Small number of participants in each arm (N < 20) | https://clinicaltrials.gov/study/NCT05256134 |
| NCT00676143 | Stopped Early | https://clinicaltrials.gov/study/NCT00676143 |
| NCT00996918 | Stopped Early | https://pubmed.ncbi.nlm.nih.gov/27334799/ |
| NCT00998764 | Stopped Early | https://pubmed.ncbi.nlm.nih.gov/27334799/ |
| NCT01224106 | Stopped Early | https://clinicaltrials.gov/study/NCT01224106 |
| NCT01343966 | Stopped Early | https://clinicaltrials.gov/study/NCT01343966 |
| NCT01969123 | Stopped Early | https://clinicaltrials.gov/study/NCT01969123 |
| NCT01969136 | Stopped Early | https://clinicaltrials.gov/study/NCT01969136 |
| NCT02004392 | Stopped Early | https://clinicaltrials.gov/study/NCT02004392 |
| NCT02500784 | Stopped Early | https://clinicaltrials.gov/study/NCT02500784 |
| NCT03114657 | Stopped Early | https://clinicaltrials.gov/study/NCT03114657 |
| NCT03443973 | Stopped Early | https://clinicaltrials.gov/study/NCT03443973 |
| NCT03444870 | Stopped Early | https://clinicaltrials.gov/study/NCT03444870 |
| NCT04520412 | Stopped Early | https://clinicaltrials.gov/study/NCT04520412 |
| NCT05552157 | Stopped Early | https://clinicaltrials.gov/study/NCT05552157 |
| NCT03811847 | Supportive Care Study | https://clinicaltrials.gov/study/NCT03811847 |
| NCT00088673 | Unknown Status | https://clinicaltrials.gov/study/NCT00088673 |
| NCT00142324 | Unknown Status | https://clinicaltrials.gov/study/NCT00142324 |
| NCT00154635 | Unknown Status | https://clinicaltrials.gov/study/NCT00154635 |
| NCT00174525 | Unknown Status | https://clinicaltrials.gov/study/NCT00174525 |
| NCT00190021 | Unknown Status | https://clinicaltrials.gov/study/NCT00190021 |
| NCT00217763 | Unknown Status | https://clinicaltrials.gov/study/NCT00217763 |
| NCT00229333 | Unknown Status | https://clinicaltrials.gov/study/NCT00229333 |
| NCT00232570 | Unknown Status | https://clinicaltrials.gov/study/NCT00232570 |
| NCT00239746 | Unknown Status | https://clinicaltrials.gov/study/NCT00239746 |
| NCT00242593 | Unknown Status | https://clinicaltrials.gov/study/NCT00242593 |
| NCT00306124 | Unknown Status | https://clinicaltrials.gov/study/NCT00306124 |
| NCT00314912 | Unknown Status | https://clinicaltrials.gov/study/NCT00314912 |
| NCT00523666 | Unknown Status | https://clinicaltrials.gov/study/NCT00523666 |
| NCT00606164 | Unknown Status | https://clinicaltrials.gov/study/NCT00606164 |
| NCT00626613 | Unknown Status | https://clinicaltrials.gov/study/NCT00626613 |
| NCT00703430 | Unknown Status | https://clinicaltrials.gov/study/NCT00703430 |
| NCT00715858 | Unknown Status | https://clinicaltrials.gov/study/NCT00715858 |
| NCT00866060 | Unknown Status | https://clinicaltrials.gov/study/NCT00866060 |
| NCT00927108 | Unknown Status | https://clinicaltrials.gov/study/NCT00927108 |
| NCT01094340 | Unknown Status | https://clinicaltrials.gov/study/NCT01094340 |
| NCT01120002 | Unknown Status | https://clinicaltrials.gov/study/NCT01120002 |
| NCT01163825 | Unknown Status | https://clinicaltrials.gov/study/NCT01163825 |
| NCT01255046 | Unknown Status | https://clinicaltrials.gov/study/NCT01255046 |
| NCT01282619 | Unknown Status | https://clinicaltrials.gov/study/NCT01282619 |
| NCT01547689 | Unknown Status | https://clinicaltrials.gov/study/NCT01547689 |
| NCT01569516 | Unknown Status | https://clinicaltrials.gov/study/NCT01569516 |
| NCT01849042 | Unknown Status | https://clinicaltrials.gov/study/NCT01849042 |
| NCT01867775 | Unknown Status | https://clinicaltrials.gov/study/NCT01867775 |
| NCT01940952 | Unknown Status | https://clinicaltrials.gov/study/NCT01940952 |
| NCT01953705 | Unknown Status | https://clinicaltrials.gov/study/NCT01953705 |
| NCT02063269 | Unknown Status | https://clinicaltrials.gov/study/NCT02063269 |
| NCT02288000 | Unknown Status | https://clinicaltrials.gov/study/NCT02288000 |
| NCT02648906 | Unknown Status | https://clinicaltrials.gov/study/NCT02648906 |
| NCT02672306 | Unknown Status | https://clinicaltrials.gov/study/NCT02672306 |
| NCT02820155 | Unknown Status | https://clinicaltrials.gov/study/NCT02820155 |
| NCT03038035 | Unknown Status | https://clinicaltrials.gov/study/NCT03038035 |
| NCT03038282 | Unknown Status | https://clinicaltrials.gov/study/NCT03038282 |
| NCT03038334 | Unknown Status | https://clinicaltrials.gov/study/NCT03038334 |
| NCT03090516 | Unknown Status | https://clinicaltrials.gov/study/NCT03090516 |
| NCT03151382 | Unknown Status | https://clinicaltrials.gov/study/NCT03151382 |
| NCT03168997 | Unknown Status | https://clinicaltrials.gov/study/NCT03168997 |
| NCT03234686 | Unknown Status | https://clinicaltrials.gov/study/NCT03234686 |
| NCT03277573 | Unknown Status | https://clinicaltrials.gov/study/NCT03277573 |
| NCT03283059 | Unknown Status | https://clinicaltrials.gov/study/NCT03283059 |
| NCT03489044 | Unknown Status | https://clinicaltrials.gov/study/NCT03489044 |
| NCT03625401 | Unknown Status | https://clinicaltrials.gov/study/NCT03625401 |
| NCT04121208 | Unknown Status | https://clinicaltrials.gov/study/NCT04121208 |
| NCT04229186 | Unknown Status | https://clinicaltrials.gov/study/NCT04229186 |
| NCT04229927 | Unknown Status | https://clinicaltrials.gov/study/NCT04229927 |
| NCT04305210 | Unknown Status | https://clinicaltrials.gov/study/NCT04305210 |
| NCT04476303 | Unknown Status | https://clinicaltrials.gov/study/NCT04476303 |
| NCT04604600 | Unknown Status | https://clinicaltrials.gov/study/NCT04604600 |
| NCT04663854 | Unknown Status | https://clinicaltrials.gov/study/NCT04663854 |
| NCT04842552 | Unknown Status | https://clinicaltrials.gov/study/NCT04842552 |
| NCT04920786 | Unknown Status | https://clinicaltrials.gov/study/NCT04920786 |
| NCT05164536 | Unknown Status | https://clinicaltrials.gov/study/NCT05164536 |
| NCT00375557 | Withdrawn | https://clinicaltrials.gov/study/NCT00375557 |
| NCT00706186 | Withdrawn | https://clinicaltrials.gov/study/NCT00706186 |
| NCT00711204 | Withdrawn | https://clinicaltrials.gov/study/NCT00711204 |
| NCT00743743 | Withdrawn | https://clinicaltrials.gov/study/NCT00743743 |
| NCT00792662 | Withdrawn | https://clinicaltrials.gov/study/NCT00792662 |
| NCT00884533 | Withdrawn | https://clinicaltrials.gov/study/NCT00884533 |
| NCT01066481 | Withdrawn | https://clinicaltrials.gov/study/NCT01066481 |
| NCT01211782 | Withdrawn | https://clinicaltrials.gov/study/NCT01211782 |
| NCT01255163 | Withdrawn | https://clinicaltrials.gov/study/NCT01255163 |
| NCT01636596 | Withdrawn | https://clinicaltrials.gov/study/NCT01636596 |
| NCT01723670 | Withdrawn | https://clinicaltrials.gov/study/NCT01723670 |
| NCT01822951 | Withdrawn | https://clinicaltrials.gov/study/NCT01822951 |
| NCT02130661 | Withdrawn | https://clinicaltrials.gov/study/NCT02130661 |
| NCT02246075 | Withdrawn | https://clinicaltrials.gov/study/NCT02246075 |
| NCT02467413 | Withdrawn | https://clinicaltrials.gov/study/NCT02467413 |
| NCT02667496 | Withdrawn | https://clinicaltrials.gov/study/NCT02667496 |
| NCT02860065 | Withdrawn | https://clinicaltrials.gov/study/NCT02860065 |
| NCT02912169 | Withdrawn | https://clinicaltrials.gov/study/NCT02912169 |
| NCT03316898 | Withdrawn | https://clinicaltrials.gov/study/NCT03316898 |
| NCT03441516 | Withdrawn | https://clinicaltrials.gov/study/NCT03441516 |
| NCT03585907 | Withdrawn | https://clinicaltrials.gov/study/NCT03585907 |
| NCT03635879 | Withdrawn | https://clinicaltrials.gov/study/NCT03635879 |
| NCT04094129 | Withdrawn | https://clinicaltrials.gov/study/NCT04094129 |
| NCT04187547 | Withdrawn | https://clinicaltrials.gov/study/NCT04187547 |
| NCT05321498 | Withdrawn | https://clinicaltrials.gov/study/NCT05321498 |
| NCT05332678 | Withdrawn | https://clinicaltrials.gov/study/NCT05332678 |
| NCT05728736 | Withdrawn | https://clinicaltrials.gov/study/NCT05728736 |

| **Study Name (Year) Dose** | **Clinical Trial ID** | **Phase** | **Drug** |
| --- | --- | --- | --- |
| **Salloway et al (2009) high dose** | **NCT00112073** | **II** | **Bapineuzumab** |
| **Salloway et al 1 (2014) Study 301 low dose** | **NCT00574132** | **III** | **Bapineuzumab** |
| **Salloway et al 2 (2014) Study 301 high dose** | **NCT00574132** | **III** | **Bapineuzumab** |
| **Salloway et al 3 (2014) Study 302 low dose** | **NCT00575055** | **III** | **Bapineuzumab** |
| **Doody et al 1 (2014) EXPEDITION 1** | **NCT00905372** | **III** | **Solanezumab** |
| **Doody et al 2 (2014) EXPEDITION 2** | **NCT00904683** | **III** | **Solanezumab** |
| **Vandenberghe et al 1 (2016) low dose** | **NCT00667810** | **III** | **Bapineuzumab** |
| **Vandenberghe et al 2 (2016) high dose** | **NCT00667810** | **III** | **Bapineuzumab** |
| **Vandenberghe et al 3 (2016) low dose** | **NCT00676143** | **III** | **Bapineuzumab** |
| **Honig et al (2018) EXPEDITION 3** | **NCT01900665** | **III** | **Solanezumab** |
| **Haeberlein et al (2022) EMERGE low dose** | **NCT02484547** | **III** | **Aducanumab** |
| **Haeberlein et al (2022) EMERGE high dose** | **NCT02484547** | **III** | **Aducanumab** |
| **Haeberlein et al (2022) ENGAGE low dose** | **NCT02477800** | **III** | **Aducanumab** |
| **Haeberlein et al (2022) ENGAGE high dose** | **NCT02477800** | **III** | **Aducanumab** |
| **van Dyck et al (2023) Clarity AD** | **NCT03887455** | **III** | **Lecanemab** |
| **Swanson et al.1 (2021) high dose** | **NCT01767311** | **II** | **Lecanemab** |
| **Swanson et al.2 (2021) low dose** | **NCT01767311** | **II** | **Lecanemab** |
| **Sims et al (2023) TRAILBLAZER-ALZ 2 (pooled)** | **NCT04437511** | **III** | **Donanemab** |
| **Mintun et al (2021) TRAILBLAZER-ALZ** | **NCT03367403** | **II** | **Donanemab** |
| **Bateman et al (2023) GRADUATE I** | **NCT03444870** | **III** | **Gantenerumab** |
| **Bateman et al (2023) GRADUATE II** | **NCT03443973** | **III** | **Gantenerumab** |
| **Ostrowitzki et al (2022) CREAD** | **NCT02670083** | **III** | **Crenezumab** |
| **Salloway et al (2018) BLAZE (pooled)** | **NCT01397578** | **II** | **Crenezumab** |
| **Ostrowitzki et al (2017) SCarlet RoAD I** | **NCT01224106** | **III** | **Gantenerumab** |
| **Ostrowitzki et al (2017) SCarlet RoAD II** | **NCT01224106** | **III** | **Gantenerumab** |
