# Supplementary figures and images for: Clinical Benefits and Risks of Antiamyloid Antibodies in Sporadic Alzheimer Disease: Systematic Review and Network Meta-Analysis With a Web Application
Source: J Med Internet Res. 2025 Apr 7;27:e68454. doi: 10.2196/68454 (PMC12012406; doi:10.2196/68454)

## Slide 1
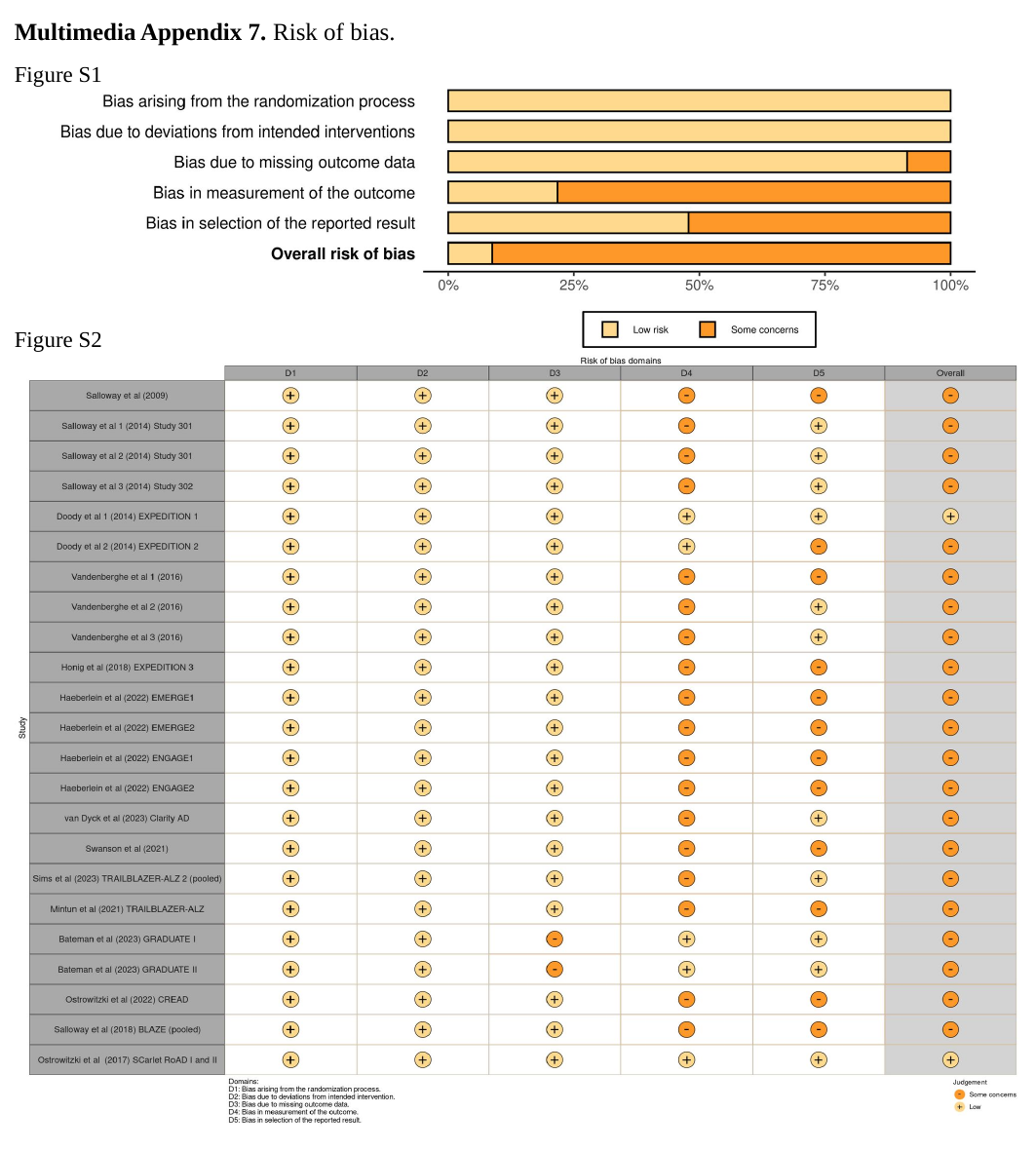

Multimedia Appendix 7. Risk of bias.
Figure S1
Figure S2

Supplement: Multimedia Appendix 7 [file jmir_v27i1e68454_app7.pptx]
