# Supplementary material for: Clinical Benefits and Risks of Antiamyloid Antibodies in Sporadic Alzheimer Disease: Systematic Review and Network Meta-Analysis With a Web Application
Source: J Med Internet Res. 2025 Apr 7;27:e68454. doi: 10.2196/68454 (PMC12012406; doi:10.2196/68454)
Supplement: Multimedia Appendix 8 [file jmir_v27i1e68454_app8.pptx]

## Slide 1
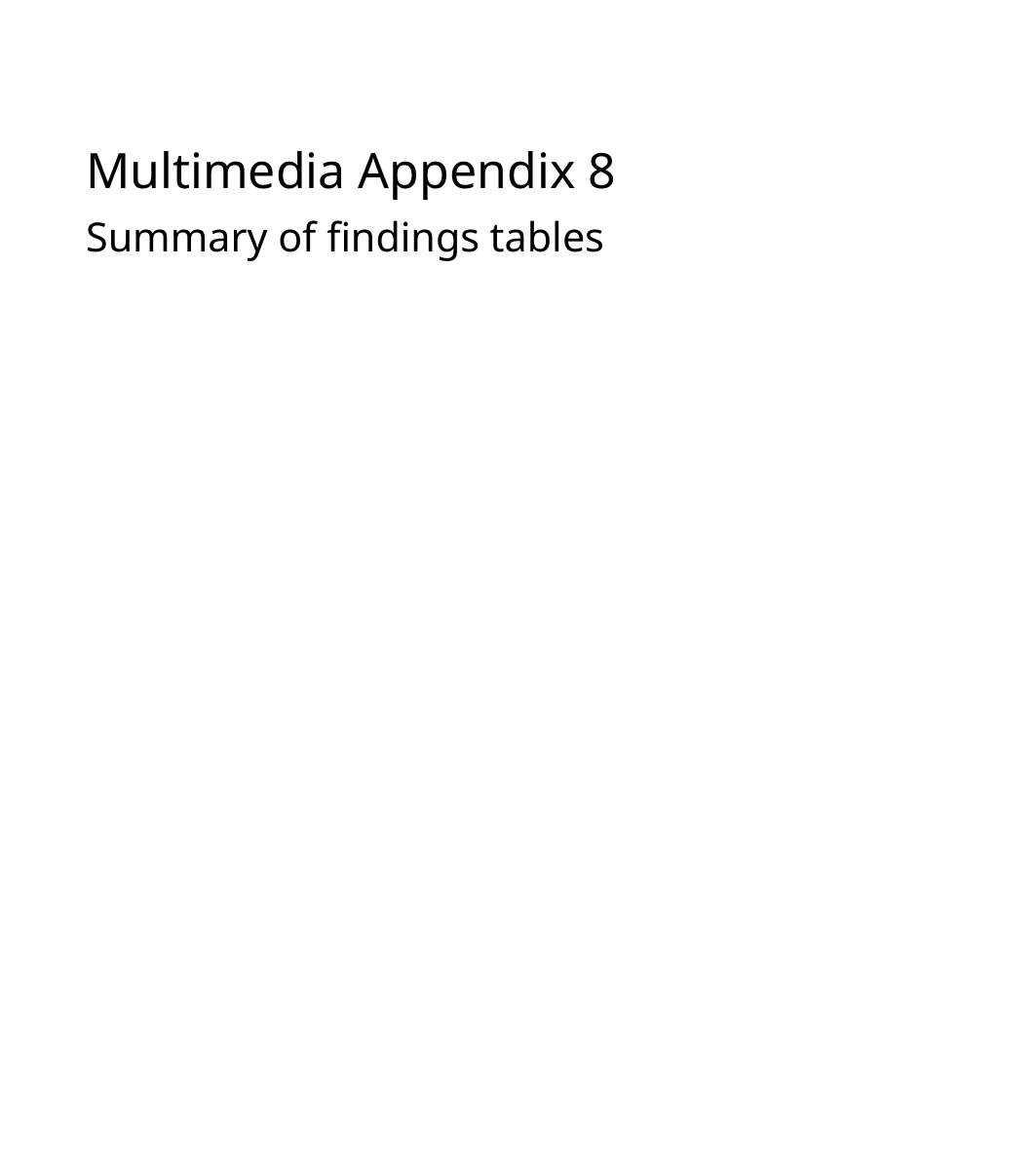

# Multimedia Appendix 8
Summary of findings tables

## Slide 2
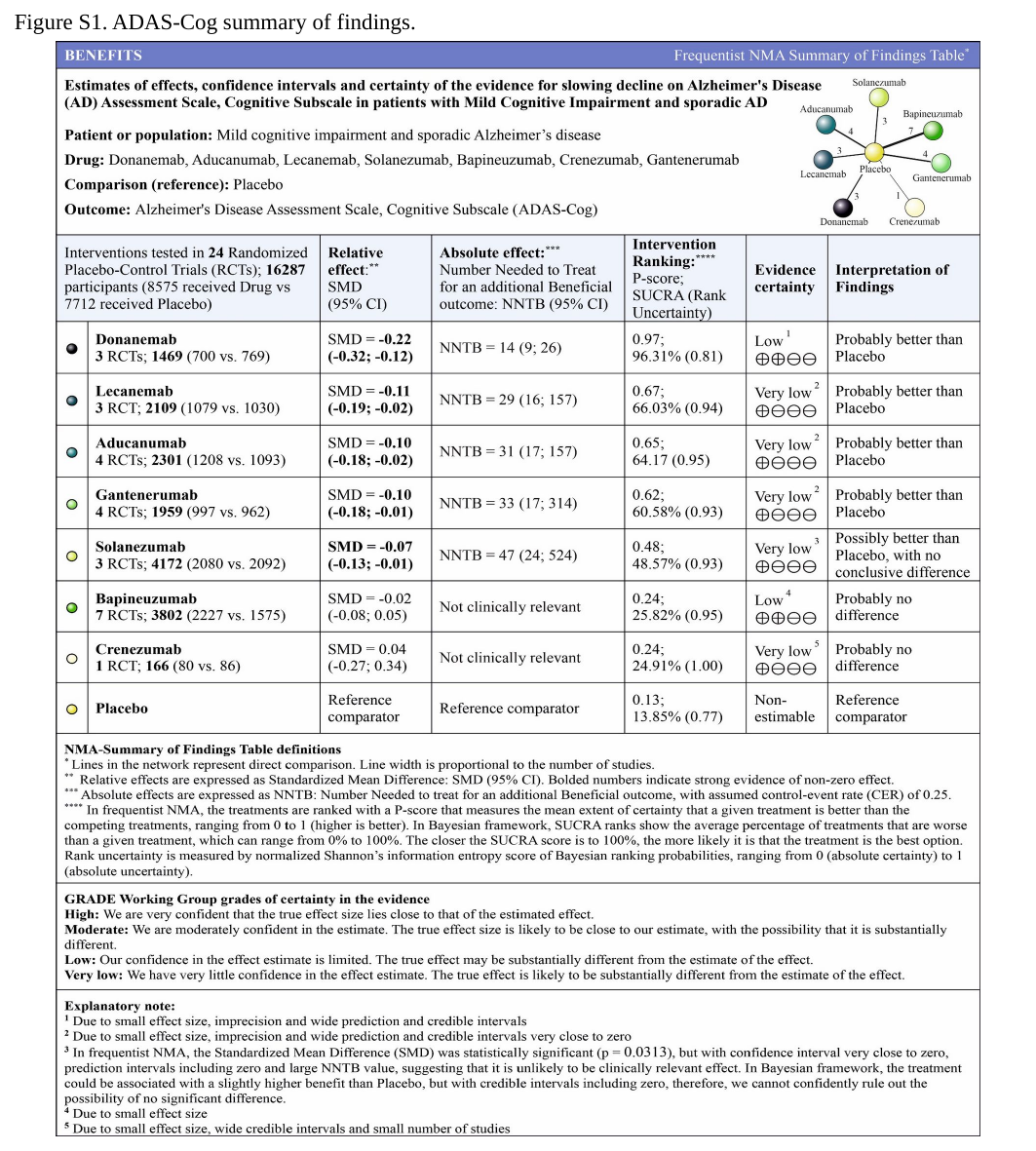

Figure S1. ADAS-Cog summary of findings.
#

## Slide 3
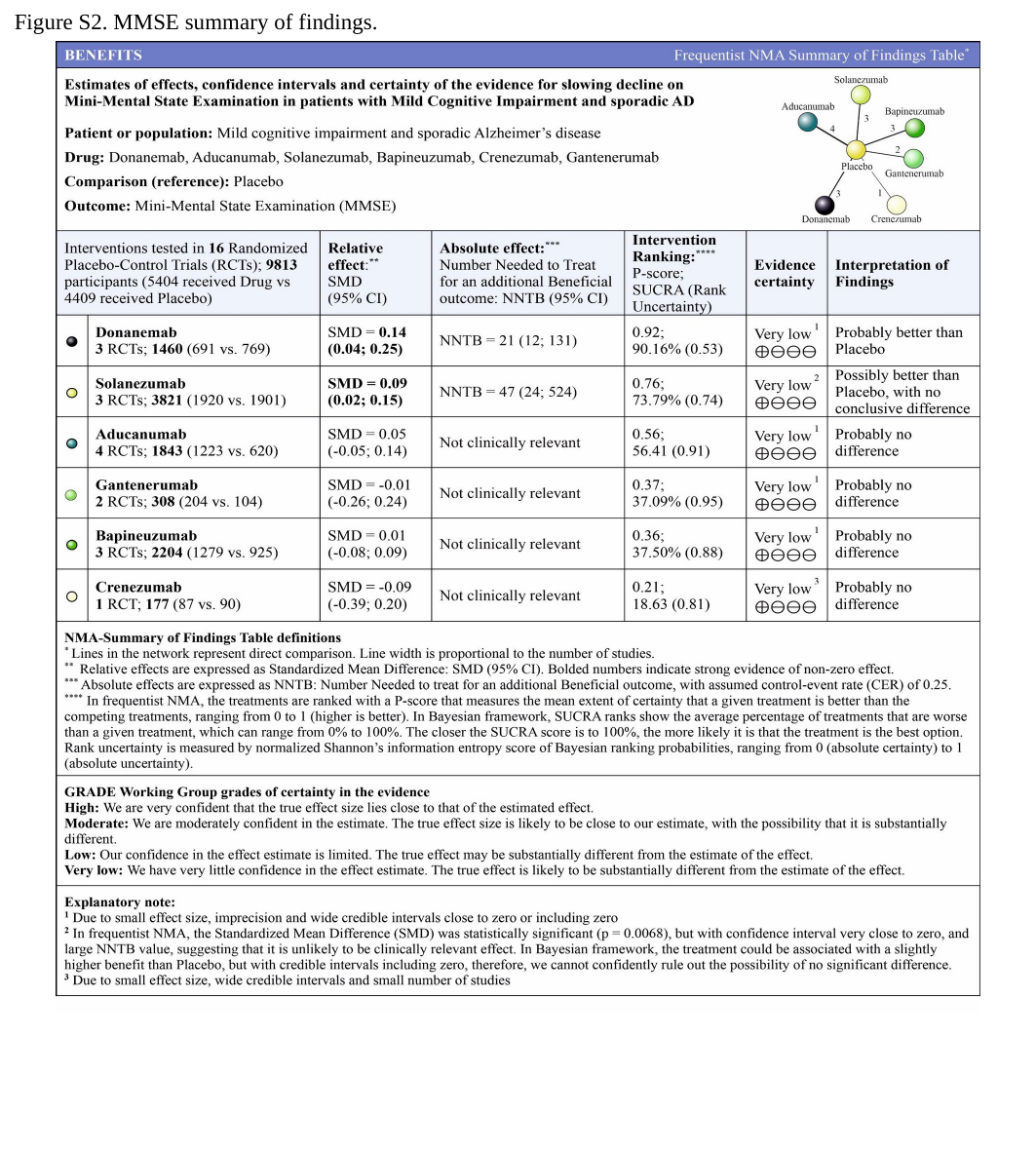

Figure S2. MMSE summary of findings.
#

## Slide 4
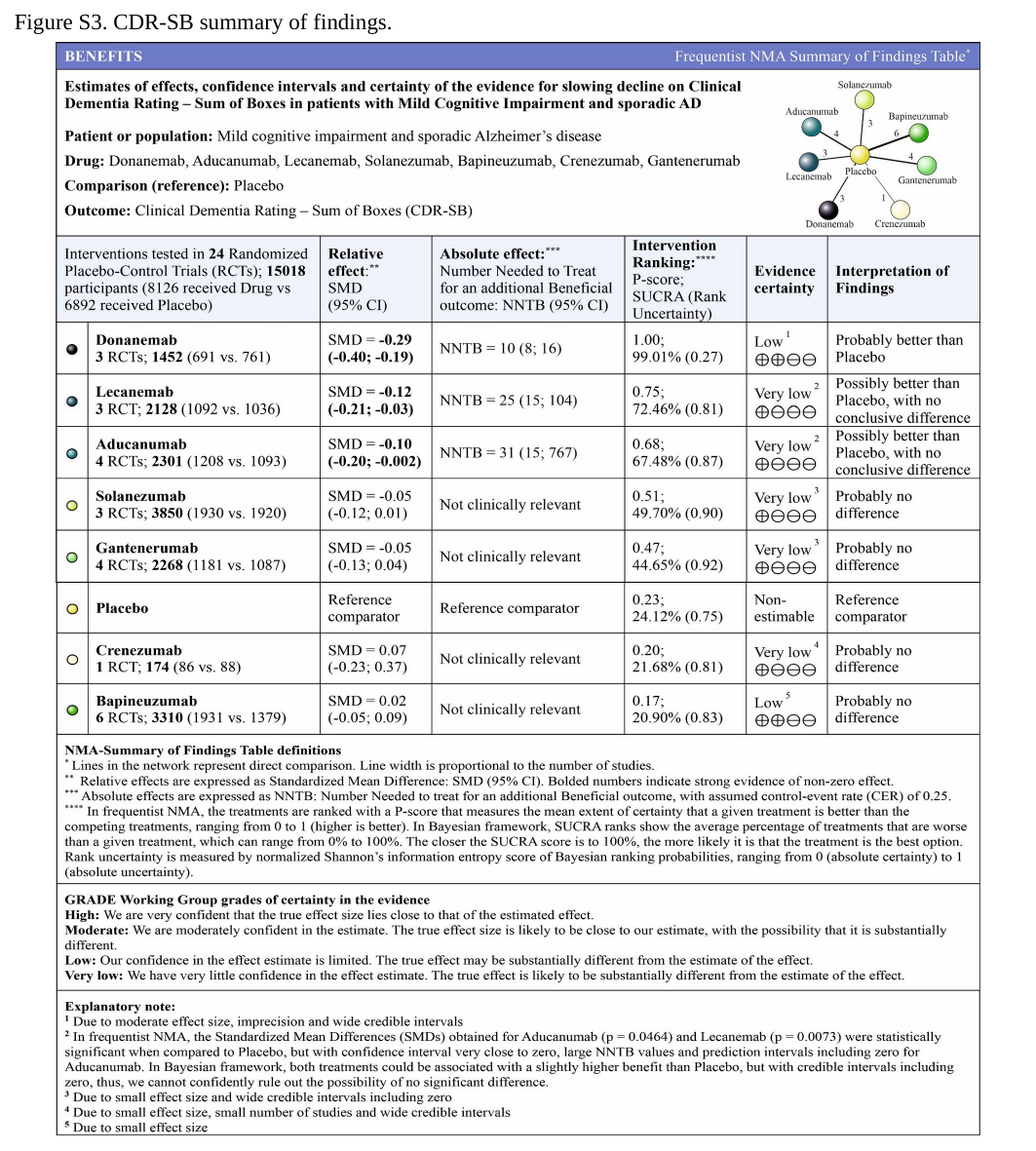

Figure S3. CDR-SB summary of findings.
#

## Slide 5
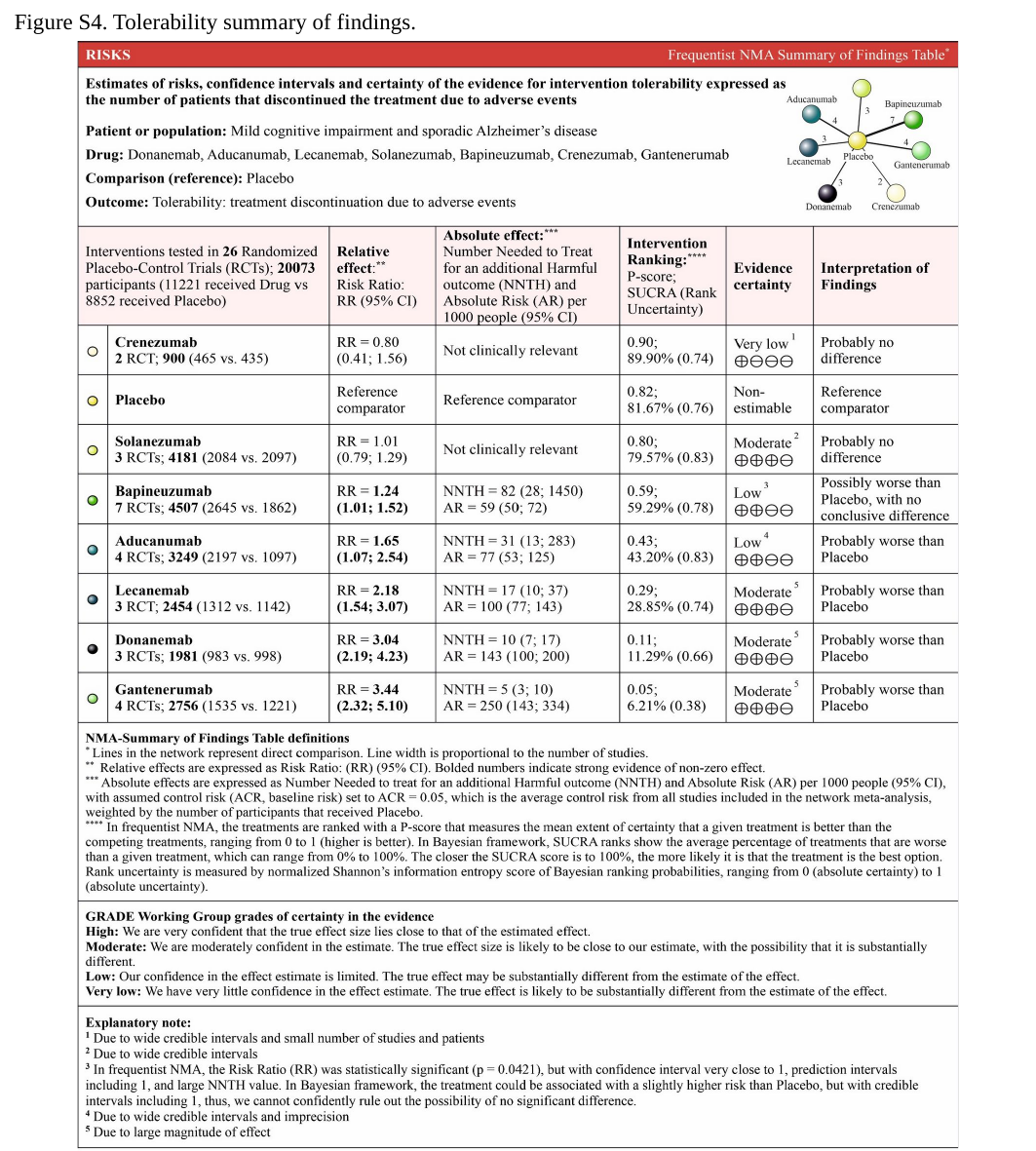

Figure S4. Tolerability summary of findings.
#

## Slide 6
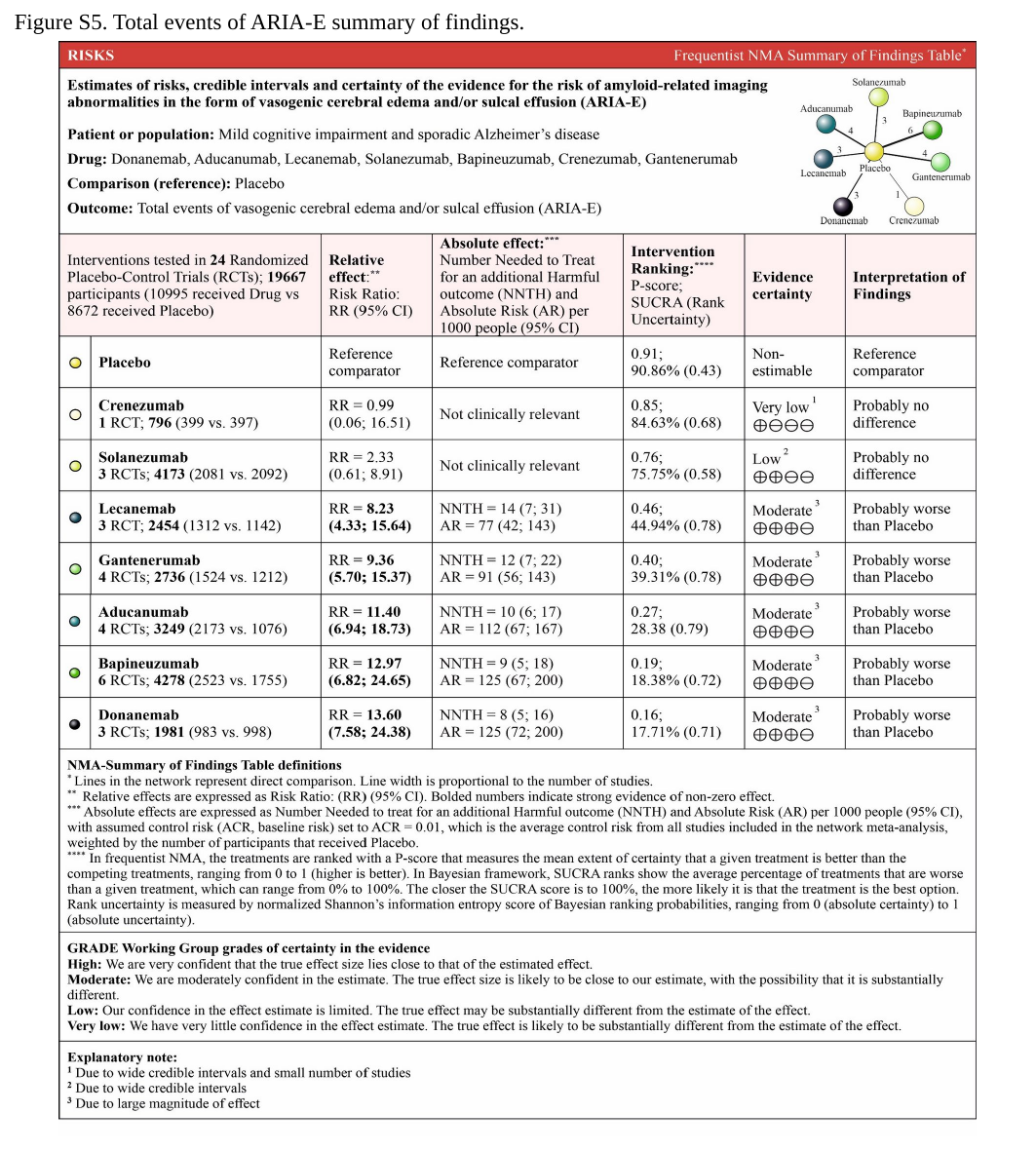

Figure S5. Total events of ARIA-E summary of findings.
#

## Slide 7
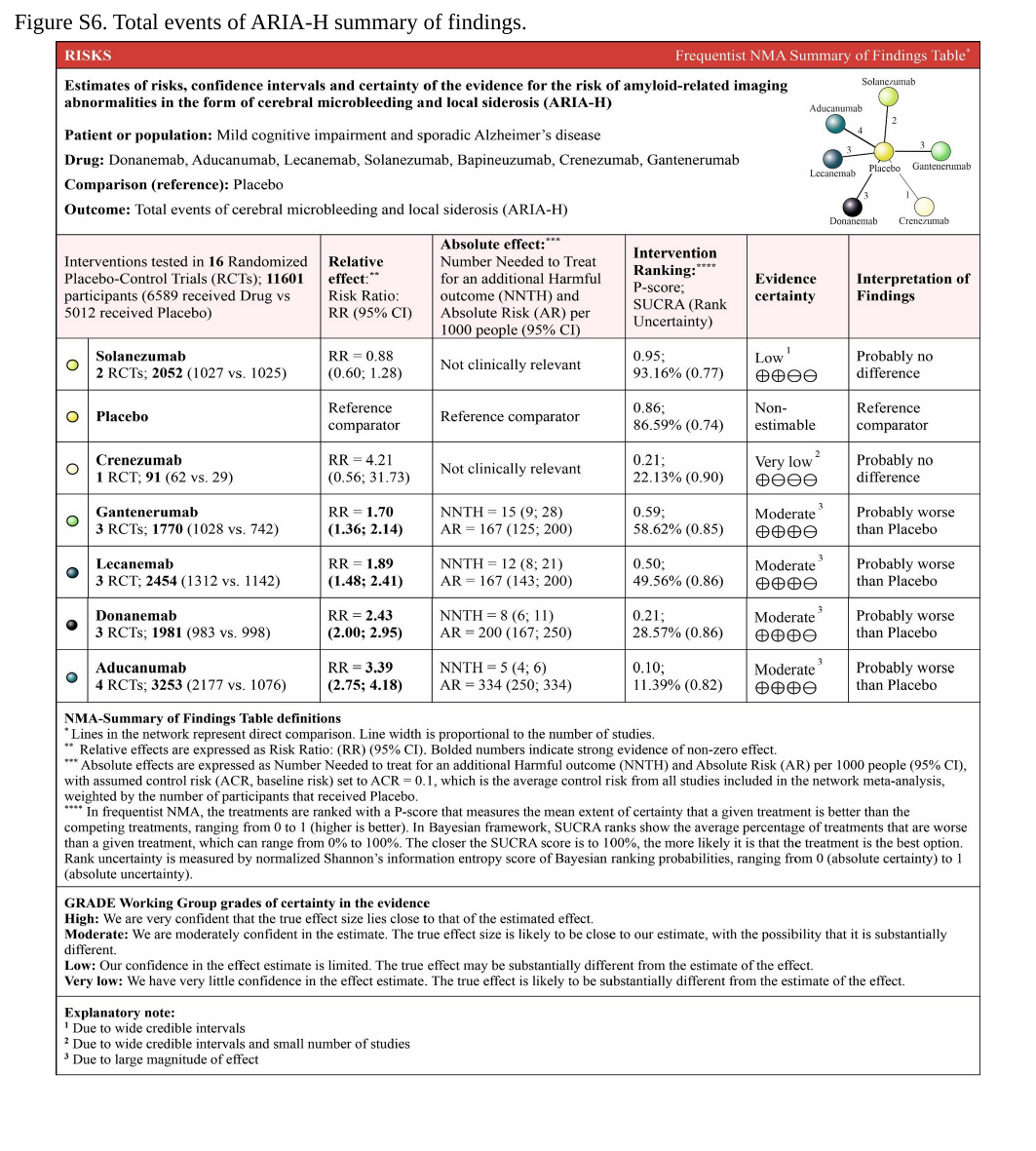

Figure S6. Total events of ARIA-H summary of findings.
#

## Slide 8
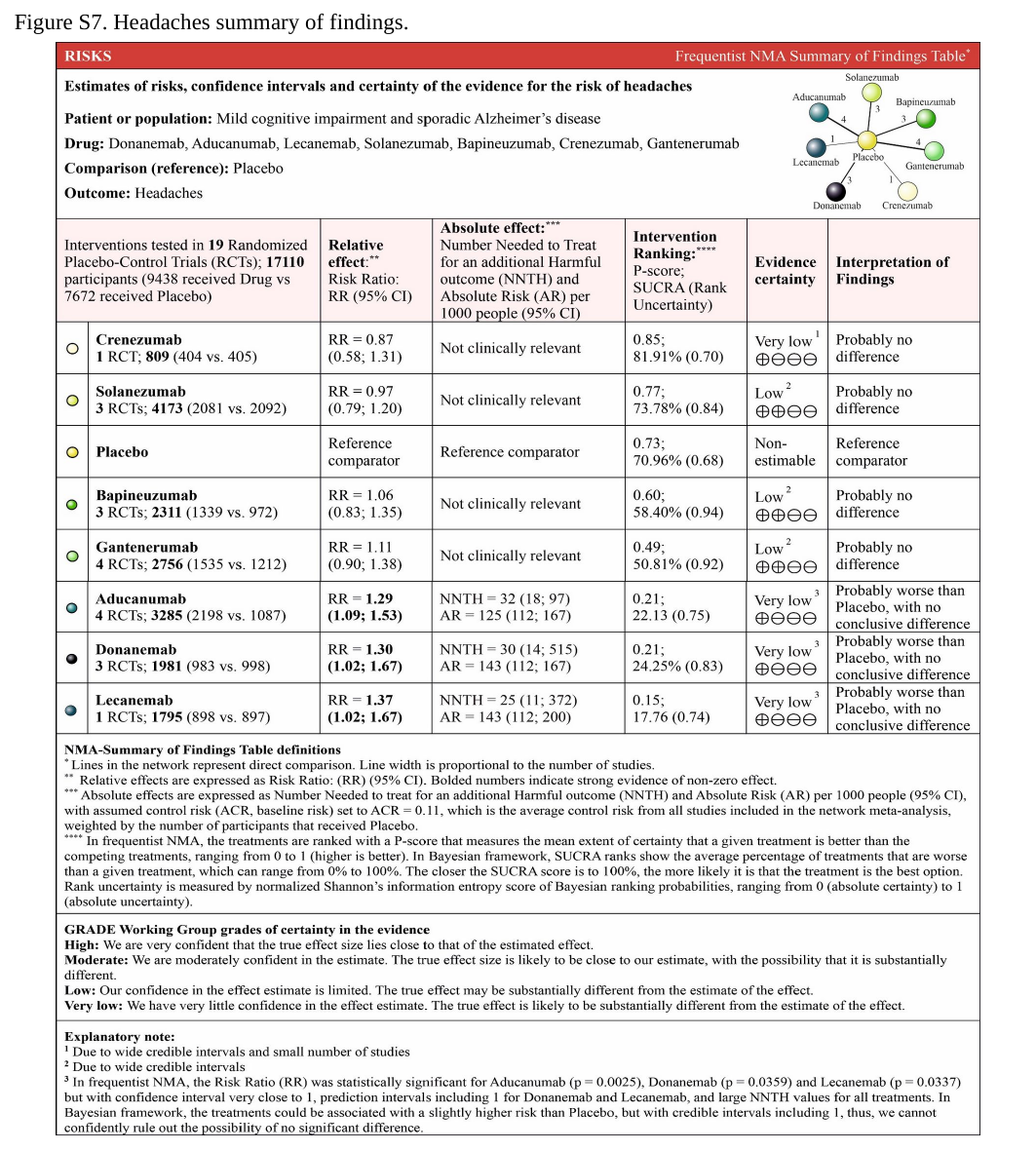

Figure S7. Headaches summary of findings.
#

## Slide 9
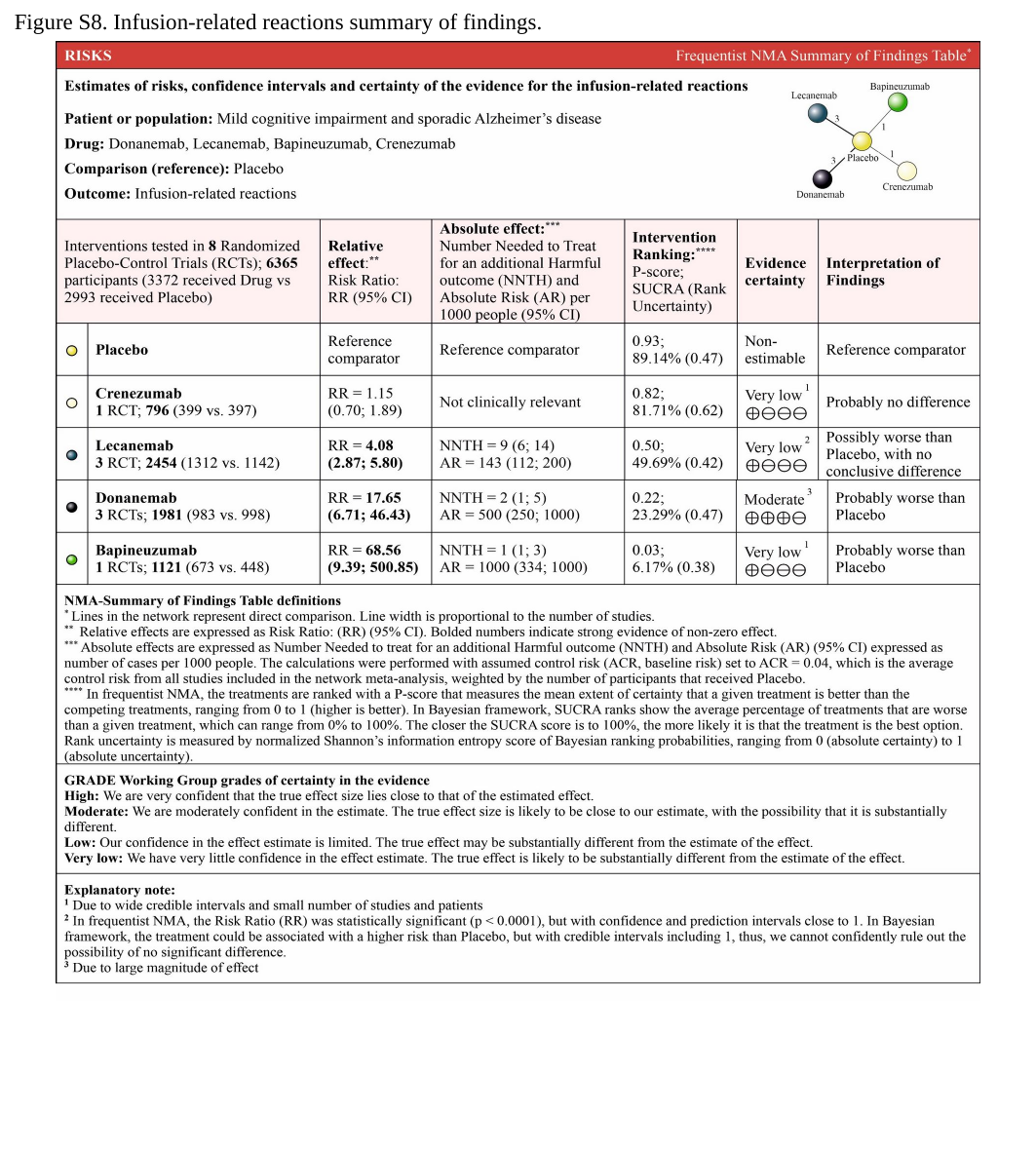

Figure S8. Infusion-related reactions summary of findings.
#
