# Supplementary material for: Clinical Benefits and Risks of Antiamyloid Antibodies in Sporadic Alzheimer Disease: Systematic Review and Network Meta-Analysis With a Web Application
Source: J Med Internet Res. 2025 Apr 7;27:e68454. doi: 10.2196/68454 (PMC12012406; doi:10.2196/68454)
Supplement: Multimedia Appendix 9 [file jmir_v27i1e68454_app9.docx]

### **Multimedia Appendix 9.** Summary of findings tables for each comparison within the network meta-analysis (GRADE).

The certainty of evidence is evaluated following the methodology outlined by the Grading of Recommendations, Assessment, Development, and Evaluation (GRADE) Working Group, focusing on the following items:

1. Number of studies (direct comparison)
2. Risk of bias
3. Inconsistency
4. Indirectness
5. Imprecision
6. Publication bias

| **Outcome** | **Comparison** | **I** | **II** | **III** | **IV** | **V** | **VI** | **Rating** | **Reason(s) for downgrading** | **Reason for upgrading** |
| --- | --- | --- | --- | --- | --- | --- | --- | --- | --- | --- |
| Tolerability | Bapineuzumab vs. Placebo | 7 | Some concerns | No concerns | No concerns | No concerns | No concerns | Low | Reporting Bias; Imprecision | Not upgraded |
| ADAS-Cog | Bapineuzumab vs. Placebo | 7 | Some concerns | No concerns | No concerns | Major concerns | No concerns | Low | Reporting Bias; Imprecision | Not upgraded |
| Serious Side Effects | Bapineuzumab vs. Placebo | 7 | Some concerns | No concerns | No concerns | No concerns | No concerns | Very Low | Reporting Bias; Imprecision; Heterogeneity | Not upgraded |
| ARIA-E | Bapineuzumab vs. Placebo | 6 | Some concerns | No concerns | No concerns | No concerns | No concerns | Moderate | Reporting Bias; Heterogeneity | Due to large effect size |
| Arthralgia | Bapineuzumab vs. Placebo | 6 | Some concerns | Some concerns | No concerns | No concerns | No concerns | Low | Reporting Bias; Indirectness | Not upgraded |
| Urinary Infections | Bapineuzumab vs. Placebo | 6 | Some concerns | Some concerns | No concerns | No concerns | No concerns | Low | Reporting Bias; Imprecision; Heterogeneity | Not upgraded |
| Headaches | Bapineuzumab vs. Placebo | 6 | Some concerns | Major concerns | No concerns | No concerns | No concerns | Very Low | Reporting Bias; Heterogeneity | Not upgraded |
| Fall | Bapineuzumab vs. Placebo | 6 | Some concerns | No concerns | No concerns | No concerns | No concerns | Low | Reporting Bias; Imprecision | Not upgraded |
| CDR-SB | Bapineuzumab vs. Placebo | 6 | Some concerns | No concerns | No concerns | Major concerns | No concerns | Low | Reporting Bias; Imprecision | Not upgraded |
| ARIA-E | Aducanumab vs. Placebo | 4 | Some concerns | No concerns | No concerns | No concerns | No concerns | Moderate | Reporting Bias | Due to large effect size |
| ARIA-E in APOE-ε4 Carriers | Aducanumab vs. Placebo | 4 | Some concerns | No concerns | No concerns | No concerns | No concerns | Moderate | Reporting Bias | Due to large effect size |
| ARIA-E in APOE-ε4 Non-Carriers | Aducanumab vs. Placebo | 4 | Some concerns | No concerns | No concerns | No concerns | No concerns | Moderate | Reporting Bias | Due to large effect size |
| ARIA-E in APOE-ε4 Carriers | Gantenerumab vs. Placebo | 4 | Some concerns | No concerns | No concerns | No concerns | No concerns | Moderate | Reporting Bias | Due to large effect size |
| ARIA-E | Gantenerumab vs. Placebo | 4 | Some concerns | No concerns | No concerns | No concerns | No concerns | Moderate | Reporting Bias | Due to large effect size |
| ARIA-E in APOE-ε4 Non-Carriers | Gantenerumab vs. Placebo | 4 | Some concerns | No concerns | No concerns | No concerns | No concerns | Moderate | Reporting Bias | Due to large effect size |
| CSF Aβ 1-42 | Aducanumab vs. Placebo | 4 | Some concerns | No concerns | No concerns | Major concerns | No concerns | Low | Reporting Bias; Imprecision | Not upgraded |
| Amyloid Burden on PET | Aducanumab vs. Placebo | 4 | Some concerns | No concerns | No concerns | Major concerns | No concerns | Low | Reporting Bias; Imprecision | Not upgraded |
| ARIA-H | Aducanumab vs. Placebo | 4 | Some concerns | No concerns | No concerns | No concerns | No concerns | Low | Reporting Bias; Imprecision | Not upgraded |
| Diarrhea | Aducanumab vs. Placebo | 4 | Some concerns | No concerns | No concerns | No concerns | No concerns | Low | Reporting Bias; Imprecision | Not upgraded |
| Dizziness | Aducanumab vs. Placebo | 4 | Some concerns | No concerns | No concerns | No concerns | No concerns | Low | Reporting Bias; Imprecision | Not upgraded |
| Headaches | Aducanumab vs. Placebo | 4 | Some concerns | No concerns | No concerns | No concerns | No concerns | Low | Reporting Bias; Imprecision | Not upgraded |
| Serious Side Effects | Aducanumab vs. Placebo | 4 | Some concerns | No concerns | No concerns | No concerns | No concerns | Low | Reporting Bias; Imprecision | Not upgraded |
| ADAS-Cog | Aducanumab vs. Placebo | 4 | Some concerns | No concerns | No concerns | Major concerns | No concerns | Very Low | Reporting Bias; Imprecision | Not upgraded |
| MMSE | Aducanumab vs. Placebo | 4 | Some concerns | No concerns | No concerns | Major concerns | No concerns | Very Low | Reporting Bias; Imprecision | Not upgraded |
| Nasopharynghitis | Aducanumab vs. Placebo | 4 | Some concerns | No concerns | No concerns | Major concerns | No concerns | Very Low | Reporting Bias; Imprecision | Not upgraded |
| Fall | Aducanumab vs. Placebo | 4 | Some concerns | No concerns | No concerns | No concerns | No concerns | Low | Reporting Bias; Heterogeneity | Not upgraded |
| Tolerability | Aducanumab vs. Placebo | 4 | Some concerns | No concerns | No concerns | No concerns | No concerns | Low | Reporting Bias | Not upgraded |
| CDR-SB | Aducanumab vs. Placebo | 4 | Some concerns | No concerns | No concerns | Major concerns | No concerns | Very Low | Reporting Bias; Imprecision | Not upgraded |
| CSF p-Tau | Aducanumab vs. Placebo | 4 | Some concerns | No concerns | No concerns | Major concerns | No concerns | Very Low | Reporting Bias; Imprecision; Heterogeneity | Not upgraded |
| Diarrhea | Bapineuzumab vs. Placebo | 4 | Some concerns | No concerns | No concerns | No concerns | No concerns | Low | Reporting Bias; Imprecision | Not upgraded |
| Dizziness | Bapineuzumab vs. Placebo | 4 | Some concerns | No concerns | No concerns | No concerns | No concerns | Low | Reporting Bias; Imprecision | Not upgraded |
| Nasopharynghitis | Bapineuzumab vs. Placebo | 4 | Some concerns | No concerns | No concerns | No concerns | No concerns | Low | Reporting Bias; Imprecision; Heterogeneity | Not upgraded |
| Urinary Infections | Gantenerumab vs. Placebo | 4 | Some concerns | Some concerns | No concerns | No concerns | No concerns | Very Low | Reporting Bias; Imprecision; Heterogeneity | Not upgraded |
| Arthralgia | Gantenerumab vs. Placebo | 4 | Some concerns | No concerns | No concerns | No concerns | No concerns | Low | Reporting Bias | Not upgraded |
| Diarrhea | Gantenerumab vs. Placebo | 4 | Some concerns | No concerns | No concerns | No concerns | No concerns | Low | Reporting Bias | Not upgraded |
| Dizziness | Gantenerumab vs. Placebo | 4 | Some concerns | No concerns | No concerns | No concerns | No concerns | Low | Reporting Bias | Not upgraded |
| Fall | Gantenerumab vs. Placebo | 4 | Some concerns | No concerns | No concerns | No concerns | No concerns | Low | Reporting Bias | Not upgraded |
| Headaches | Gantenerumab vs. Placebo | 4 | Some concerns | No concerns | No concerns | No concerns | No concerns | Low | Reporting Bias | Not upgraded |
| Serious Side Effects | Gantenerumab vs. Placebo | 4 | Some concerns | No concerns | No concerns | No concerns | No concerns | Low | Reporting Bias | Not upgraded |
| ADAS-Cog | Gantenerumab vs. Placebo | 4 | Some concerns | No concerns | No concerns | Major concerns | No concerns | Very Low | Reporting Bias; Imprecision | Not upgraded |
| ARIA-H | Gantenerumab vs. Placebo | 4 | Some concerns | No concerns | No concerns | No concerns | No concerns | Very Low | Reporting Bias; Small Number of studies; Heterogeneity | Not upgraded |
| CDR-SB | Gantenerumab vs. Placebo | 4 | Some concerns | No concerns | No concerns | Major concerns | No concerns | Very Low | Reporting Bias; Imprecision | Not upgraded |
| Nasopharynghitis | Gantenerumab vs. Placebo | 4 | Some concerns | No concerns | Major concerns | No concerns | No concerns | Very Low | Reporting Bias; Imprecision | Not upgraded |
| Infusion-related reactions | Donanemab vs. Placebo | 3 | Some concerns | No concerns | No concerns | No concerns | No concerns | Moderate | Imprecision | Due to large effect size |
| ARIA-E | Donanemab vs. Placebo | 3 | Some concerns | No concerns | No concerns | No concerns | No concerns | Moderate | Reporting Bias; Small Number of Studies | Due to large effect size |
| ARIA-E in APOE-ε4 Carriers | Donanemab vs. Placebo | 3 | Some concerns | No concerns | No concerns | No concerns | No concerns | Moderate | Reporting Bias; Small Number of Patients | Due to large effect size |
| Tolerability | Donanemab vs. Placebo | 3 | Some concerns | No concerns | No concerns | No concerns | No concerns | Moderate | Reporting Bias; Small Number of Studies | Due to large effect size |
| Tolerability | Gantenerumab vs. Placebo | 3 | Some concerns | No concerns | No concerns | No concerns | No concerns | Moderate | Reporting Bias | Due to large effect size |
| CDR-SB | Donanemab vs. Placebo | 3 | Some concerns | No concerns | No concerns | Major concerns | No concerns | Moderate | Reporting Bias; Imprecision | Due to moderate effect size |
| CSF p-Tau | Bapineuzumab vs. Placebo | 3 | Some concerns | No concerns | No concerns | Major concerns | No concerns | Low | Reporting Bias; Imprecision | Not upgraded |
| MMSE | Bapineuzumab vs. Placebo | 3 | Some concerns | No concerns | No concerns | Major concerns | No concerns | Very Low | Reporting Bias; Imprecision | Not upgraded |
| Amyloid Burden on PET | Donanemab vs. Placebo | 3 | Some concerns | Major concerns | No concerns | Major concerns | No concerns | Very Low | Reporting Bias; Imprecision; Heterogeneity | Not upgraded |
| ARIA-H | Donanemab vs. Placebo | 3 | Some concerns | No concerns | No concerns | No concerns | No concerns | Low | Reporting Bias; Small Number of Studies | Not upgraded |
| Arthralgia | Donanemab vs. Placebo | 3 | Some concerns | No concerns | No concerns | No concerns | No concerns | Low | Reporting Bias; Imprecision | Not upgraded |
| Diarrhea | Donanemab vs. Placebo | 3 | Some concerns | No concerns | No concerns | No concerns | No concerns | Low | Reporting Bias; Small Number of Studies | Not upgraded |
| Dizziness | Donanemab vs. Placebo | 3 | Some concerns | No concerns | No concerns | No concerns | No concerns | Low | Reporting Bias; Small Number of Studies | Not upgraded |
| Fall | Donanemab vs. Placebo | 3 | Some concerns | No concerns | No concerns | No concerns | No concerns | Low | Reporting Bias; Imprecision | Not upgraded |
| Serious Side Effects | Donanemab vs. Placebo | 3 | Some concerns | No concerns | No concerns | No concerns | No concerns | Low | Reporting Bias; Small Number of Studies | Not upgraded |
| MMSE | Donanemab vs. Placebo | 3 | Some concerns | No concerns | No concerns | Major concerns | No concerns | Very Low | Reporting Bias; Imprecision; Small Number of Studies | Not upgraded |
| Headaches | Donanemab vs. Placebo | 3 | Some concerns | No concerns | No concerns | No concerns | No concerns | Low | Reporting Bias; Small Number of Studies | Not upgraded |
| ADAS-Cog | Donanemab vs. Placebo | 3 | Some concerns | No concerns | No concerns | Major concerns | No concerns | Low | Reporting Bias; Imprecision | Not upgraded |
| Infusion-related reactions | Lecanemab vs. Placebo | 3 | Some concerns | No concerns | No concerns | No concerns | No concerns | Low | Imprecision | Not upgraded |
| Upper respiratory Infections | Solanezumab vs. Placebo | 3 | Low | No concerns | No concerns | No concerns | No concerns | Low | Reporting Bias; Heterogeneity | Not upgraded |
| ARIA-E | Solanezumab vs. Placebo | 3 | Low | No concerns | No concerns | No concerns | No concerns | Moderate | Reporting Bias; Wide credible intervals | Not upgraded |
| Back Pain | Solanezumab vs. Placebo | 3 | Low | No concerns | No concerns | No concerns | No concerns | Moderate | Reporting Bias | Not upgraded |
| Dizziness | Solanezumab vs. Placebo | 3 | Low | No concerns | No concerns | No concerns | No concerns | Moderate | Reporting Bias | Not upgraded |
| Fall | Solanezumab vs. Placebo | 3 | Low | No concerns | No concerns | No concerns | No concerns | Moderate | Reporting Bias | Not upgraded |
| Headaches | Solanezumab vs. Placebo | 3 | Low | No concerns | No concerns | No concerns | No concerns | Moderate | Reporting Bias | Not upgraded |
| Nasopharynghitis | Solanezumab vs. Placebo | 3 | Low | No concerns | No concerns | Major concerns | No concerns | Moderate | Reporting Bias | Not upgraded |
| Nausea | Solanezumab vs. Placebo | 3 | Low | No concerns | No concerns | No concerns | No concerns | Moderate | Reporting Bias | Not upgraded |
| Serious Side Effects | Solanezumab vs. Placebo | 3 | Low | No concerns | No concerns | No concerns | No concerns | Moderate | Reporting Bias | Not upgraded |
| Tolerability | Solanezumab vs. Placebo | 3 | Low | No concerns | No concerns | No concerns | No concerns | Moderate | Reporting Bias; Wide credible intervals | Not upgraded |
| Urinary Infections | Solanezumab vs. Placebo | 3 | Low | No concerns | No concerns | No concerns | No concerns | Moderate | Reporting Bias | Not upgraded |
| ADAS-Cog | Solanezumab vs. Placebo | 3 | Low | No concerns | No concerns | Major concerns | No concerns | Very Low | Reporting Bias; Imprecision | Not upgraded |
| MMSE | Solanezumab vs. Placebo | 3 | Low | No concerns | No concerns | Major concerns | No concerns | Very Low | Reporting Bias; Imprecision | Not upgraded |
| Fatigue | Solanezumab vs. Placebo | 3 | Low | No concerns | No concerns | No concerns | No concerns | Low | Reporting Bias; Heterogeneity | Not upgraded |
| CDR-SB | Solanezumab vs. Placebo | 3 | Low | No concerns | No concerns | Major concerns | No concerns | Moderate | Reporting Bias; Imprecision | Not upgraded |
| Diarrhea | Solanezumab vs. Placebo | 3 | Low | No concerns | No concerns | No concerns | No concerns | Moderate | Reporting Bias | Not upgraded |
| ARIA-E in APOE-ε4 Non-Carriers | Bapineuzumab vs. Placebo | 2 | Some concerns | No concerns | No concerns | No concerns | No concerns | Moderate | Reporting Bias | Due to large effect size |
| ARIA-E in APOE-ε4 Non-Carriers | Donanemab vs. Placebo | 2 | Some concerns | No concerns | No concerns | No concerns | No concerns | Moderate | Reporting Bias; Small Number of Patients | Due to large effect size |
| ARIA-E | Lecanemab vs. Placebo | 2 | Some concerns | No concerns | No concerns | No concerns | No concerns | Moderate | Reporting Bias; Small Number of Studies | Due to large effect size |
| Tolerability | Lecanemab vs. Placebo | 2 | Some concerns | No concerns | No concerns | No concerns | No concerns | Moderate | Reporting Bias; Wide credible intervals; Imprecision | Due to large effect size |
| Nasopharynghitis | Crenezumab vs. Placebo | 2 | Some concerns | No concerns | No concerns | No concerns | No concerns | Very Low | Reporting Bias; Imprecision; Heterogeneity | Not upgraded |
| Tolerability | Crenezumab vs. Placebo | 2 | Some concerns | No concerns | No concerns | No concerns | No concerns | Low | Reporting Bias; Wide credible intervals; Small Number of patients | Not upgraded |
| ADAS-Cog | Crenezumab vs. Placebo | 2 | Some concerns | No concerns | No concerns | Major concerns | No concerns | Very Low | Reporting Bias; Imprecision | Not upgraded |
| Back Pain | Crenezumab vs. Placebo | 2 | Some concerns | No concerns | No concerns | No concerns | No concerns | Very Low | Reporting Bias; Imprecision | Not upgraded |
| Serious Side Effects | Crenezumab vs. Placebo | 2 | Some concerns | No concerns | No concerns | No concerns | No concerns | Very Low | Reporting Bias | Not upgraded |
| Urinary Infections | Donanemab vs. Placebo | 2 | Some concerns | Some concerns | No concerns | No concerns | No concerns | Low | Reporting Bias; Small Number of Studies; Heterogeneity | Not upgraded |
| Back Pain | Donanemab vs. Placebo | 2 | Some concerns | No concerns | No concerns | No concerns | No concerns | Low | Reporting Bias; Small Number of Studies | Not upgraded |
| Fatigue | Donanemab vs. Placebo | 2 | Some concerns | No concerns | No concerns | No concerns | No concerns | Low | Reporting Bias; Small Number of Studies | Not upgraded |
| Nausea | Donanemab vs. Placebo | 2 | Some concerns | No concerns | No concerns | No concerns | No concerns | Low | Reporting Bias; Small Number of Studies | Not upgraded |
| MMSE | Gantenerumab vs. Placebo | 2 | Some concerns | No concerns | No concerns | No concerns | No concerns | Very Low | Reporting Bias; Small Number of studies | Not upgraded |
| Amyloid Burden on PET | Gantenerumab vs. Placebo | 2 | Some concerns | No concerns | No concerns | Major concerns | No concerns | Low | Reporting Bias; Imprecision | Not upgraded |
| CSF Aβ 1-42 | Lecanemab vs. Placebo | 2 | Some concerns | Major concerns | No concerns | Major concerns | No concerns | Very Low | Reporting Bias; Imprecision; Indirectness; Small Number of studies; Heterogeneity | Not upgraded |
| ADAS-Cog | Lecanemab vs. Placebo | 2 | Some concerns | No concerns | No concerns | Major concerns | No concerns | Very Low | Reporting Bias; Imprecision | Not upgraded |
| CDR-SB | Lecanemab vs. Placebo | 2 | Some concerns | No concerns | No concerns | Major concerns | No concerns | Very Low | Reporting Bias; Imprecision; Small Number of Studies | Not upgraded |
| Serious Side Effects | Lecanemab vs. Placebo | 2 | Some concerns | No concerns | No concerns | No concerns | No concerns | Very Low | Reporting Bias; Small Number of Studies; Heterogeneity | Not upgraded |
| CSF p-Tau | Lecanemab vs. Placebo | 2 | Some concerns | No concerns | No concerns | Major concerns | No concerns | Low | Reporting Bias; Imprecision | Not upgraded |
| ARIA-H | Solanezumab vs. Placebo | 2 | Low | No concerns | No concerns | No concerns | No concerns | Moderate | Reporting Bias | Not upgraded |
| CSF Aβ 1-42 | Solanezumab vs. Placebo | 2 | Low | No concerns | No concerns | Major concerns | No concerns | Moderate | Reporting Bias; Imprecision | Not upgraded |
| ARIA-E in APOE-ε4 Carriers | Bapineuzumab vs. Placebo | 1 | Some concerns | No concerns | No concerns | No concerns | No concerns | Moderate | Reporting Bias; Small Number of Patients | Due to large effect size |
| ARIA-E in APOE-ε4 Carriers | Lecanemab vs. Placebo | 1 | Some concerns | No concerns | No concerns | No concerns | No concerns | Moderate | Reporting Bias; Small Number of Patients | Due to large effect size |
| ARIA-E in APOE-ε4 Non-Carriers | Lecanemab vs. Placebo | 1 | Some concerns | No concerns | No concerns | No concerns | No concerns | Moderate | Reporting Bias; Small Number of Patients | Due to large effect size |
| Infusion-related reactions | Bapineuzumab vs. Placebo | 1 | Some concerns | No concerns | Major concerns | No concerns | No concerns | Low | Small Number of Studies, Imprecision | Not upgraded |
| Back Pain | Bapineuzumab vs. Placebo | 1 | Some concerns | No concerns | No concerns | No concerns | No concerns | Low | Reporting Bias; Imprecision | Not upgraded |
| Nausea | Bapineuzumab vs. Placebo | 1 | Some concerns | No concerns | No concerns | No concerns | No concerns | Low | Reporting Bias; Imprecision | Not upgraded |
| Upper respiratory Infections | Bapineuzumab vs. Placebo | 1 | Some concerns | No concerns | No concerns | No concerns | No concerns | Low | Reporting Bias; Imprecision | Not upgraded |
| Infusion-related reactions | Crenezumab vs. Placebo | 1 | Some concerns | No concerns | Major concerns | No concerns | No concerns | Low | Small Number of Studies, Imprecision | Not upgraded |
| ARIA-E | Crenezumab vs. Placebo | 1 | Some concerns | No concerns | No concerns | No concerns | No concerns | Very Low | Reporting Bias; Small Number of studies; Imprecision | Not upgraded |
| ARIA-H | Crenezumab vs. Placebo | 1 | Some concerns | No concerns | No concerns | No concerns | No concerns | Very Low | Reporting Bias; Small Number of studies | Not upgraded |
| CDR-SB | Crenezumab vs. Placebo | 1 | Some concerns | No concerns | No concerns | Major concerns | No concerns | Very Low | Reporting Bias; Imprecision; Small Number of studies | Not upgraded |
| CSF Aβ 1-42 | Crenezumab vs. Placebo | 1 | Some concerns | No concerns | No concerns | Major concerns | No concerns | Very Low | Reporting Bias; Imprecision; Small Number of studies | Not upgraded |
| CSF p-Tau | Crenezumab vs. Placebo | 1 | Some concerns | No concerns | No concerns | Major concerns | No concerns | Very Low | Reporting Bias; Imprecision; Small Number of studies | Not upgraded |
| Diarrhea | Crenezumab vs. Placebo | 1 | Some concerns | No concerns | No concerns | No concerns | No concerns | Very Low | Reporting Bias; Small Number of studies | Not upgraded |
| Dizziness | Crenezumab vs. Placebo | 1 | Some concerns | No concerns | No concerns | No concerns | No concerns | Very Low | Reporting Bias; Small Number of studies | Not upgraded |
| Fall | Crenezumab vs. Placebo | 1 | Some concerns | No concerns | No concerns | No concerns | No concerns | Very Low | Reporting Bias; Small Number of studies | Not upgraded |
| Headaches | Crenezumab vs. Placebo | 1 | Some concerns | No concerns | No concerns | No concerns | No concerns | Very Low | Reporting Bias; Small Number of studies | Not upgraded |
| MMSE | Crenezumab vs. Placebo | 1 | Some concerns | No concerns | No concerns | Major concerns | No concerns | Very Low | Reporting Bias; Imprecision; Small Number of studies | Not upgraded |
| Upper respiratory Infections | Crenezumab vs. Placebo | 1 | Some concerns | No concerns | No concerns | No concerns | No concerns | Very Low | Reporting Bias; Small Number of studies | Not upgraded |
| Amyloid Burden on PET | Lecanemab vs. Placebo | 1 | Some concerns | No concerns | No concerns | Major concerns | No concerns | Low | Reporting Bias; Imprecision; Small Number of studies | Not upgraded |
| ARIA-H | Lecanemab vs. Placebo | 1 | Some concerns | No concerns | No concerns | No concerns | No concerns | Low | Reporting Bias; Small Number of Studies | Not upgraded |
| Arthralgia | Lecanemab vs. Placebo | 1 | Some concerns | No concerns | No concerns | No concerns | No concerns | Low | Reporting Bias; Small Number of Studies | Not upgraded |
| Back Pain | Lecanemab vs. Placebo | 1 | Some concerns | No concerns | No concerns | No concerns | No concerns | Low | Reporting Bias; Small Number of Studies | Not upgraded |
| Diarrhea | Lecanemab vs. Placebo | 1 | Some concerns | No concerns | No concerns | No concerns | No concerns | Low | Reporting Bias; Small Number of Studies | Not upgraded |
| Dizziness | Lecanemab vs. Placebo | 1 | Some concerns | No concerns | No concerns | No concerns | No concerns | Low | Reporting Bias; Small Number of Studies | Not upgraded |
| Fall | Lecanemab vs. Placebo | 1 | Some concerns | No concerns | No concerns | No concerns | No concerns | Low | Reporting Bias; Imprecision | Not upgraded |
| Headaches | Lecanemab vs. Placebo | 1 | Some concerns | No concerns | No concerns | No concerns | No concerns | Low | Reporting Bias; Small Number of Studies | Not upgraded |
| Urinary Infections | Lecanemab vs. Placebo | 1 | Some concerns | No concerns | No concerns | No concerns | No concerns | Low | Reporting Bias; Small Number of Studies | Not upgraded |
| Arthralgia | Solanezumab vs. Placebo | 1 | Low | No concerns | No concerns | No concerns | No concerns | Moderate | Reporting Bias | Not upgraded |
| Headaches | Aducanumab vs. Bapineuzumab | 0 | Some concerns | No concerns | Major concerns | No concerns | No concerns | Low | Reporting Bias; Heterogeneity; Indirectness | Not upgraded |
| ARIA-E | Aducanumab vs. Bapineuzumab | 0 | Some concerns | No concerns | Major concerns | No concerns | No concerns | Low | Reporting Bias; Heterogeneity; Indirectness | Not upgraded |
| ARIA-E in APOE-ε4 Carriers | Aducanumab vs. Bapineuzumab | 0 | Some concerns | No concerns | Major concerns | No concerns | No concerns | Low | Reporting Bias; Imprecision; Indirectness | Not upgraded |
| ARIA-E in APOE-ε4 Non-Carriers | Aducanumab vs. Bapineuzumab | 0 | Some concerns | No concerns | Major concerns | No concerns | No concerns | Low | Reporting Bias; Imprecision; Indirectness | Not upgraded |
| Serious Side Effects | Aducanumab vs. Bapineuzumab | 0 | Some concerns | No concerns | Major concerns | No concerns | No concerns | Low | Reporting Bias; Heterogeneity; Indirectness | Not upgraded |
| Tolerability | Aducanumab vs. Bapineuzumab | 0 | Some concerns | No concerns | Major concerns | No concerns | No concerns | Low | Reporting Bias; Imprecision; Indirectness | Not upgraded |
| ADAS-Cog | Aducanumab vs. Bapineuzumab | 0 | Some concerns | No concerns | Major concerns | Major concerns | No concerns | Very Low | Reporting Bias; Imprecision; Indirectness | Not upgraded |
| CSF p-Tau | Aducanumab vs. Bapineuzumab | 0 | Some concerns | No concerns | Major concerns | Major concerns | No concerns | Low | Reporting Bias; Imprecision; Indirectness | Not upgraded |
| Diarrhea | Aducanumab vs. Bapineuzumab | 0 | Some concerns | No concerns | Major concerns | No concerns | No concerns | Low | Reporting Bias; Imprecision; Indirectness | Not upgraded |
| Dizziness | Aducanumab vs. Bapineuzumab | 0 | Some concerns | No concerns | Major concerns | No concerns | No concerns | Low | Reporting Bias; Imprecision; Indirectness | Not upgraded |
| Fall | Aducanumab vs. Bapineuzumab | 0 | Some concerns | No concerns | Major concerns | No concerns | No concerns | Low | Reporting Bias; Heterogeneity; Indirectness | Not upgraded |
| CDR-SB | Aducanumab vs. Bapineuzumab | 0 | Some concerns | No concerns | Major concerns | Major concerns | No concerns | Very Low | Reporting Bias; Imprecision; Indirectness | Not upgraded |
| MMSE | Aducanumab vs. Bapineuzumab | 0 | Some concerns | No concerns | Major concerns | Major concerns | No concerns | Very Low | Reporting Bias; Imprecision; Indirectness | Not upgraded |
| Nasopharynghitis | Aducanumab vs. Bapineuzumab | 0 | Some concerns | No concerns | Major concerns | Major concerns | No concerns | Very Low | Reporting Bias; Imprecision; Heterogeneity; Indirectness | Not upgraded |
| Amyloid Burden on PET | Aducanumab vs. Donanemab | 0 | Some concerns | Major concerns | Major concerns | Major concerns | No concerns | Very Low | Reporting Bias; Imprecision; Indirectness; Heterogeneity | Not upgraded |
| ARIA-E in APOE-ε4 Carriers | Aducanumab vs. Donanemab | 0 | Some concerns | No concerns | Major concerns | No concerns | No concerns | Low | Reporting Bias; Small Number of Studies; Indirectness | Not upgraded |
| ARIA-E in APOE-ε4 Non-Carriers | Aducanumab vs. Donanemab | 0 | Some concerns | No concerns | Major concerns | No concerns | No concerns | Low | Reporting Bias; Small Number of Patients; Indirectness | Not upgraded |
| Headaches | Aducanumab vs. Donanemab | 0 | Some concerns | No concerns | Major concerns | No concerns | No concerns | Low | Reporting Bias; Small Number of Studies; Indirectness | Not upgraded |
| Serious Side Effects | Aducanumab vs. Donanemab | 0 | Some concerns | No concerns | Major concerns | No concerns | No concerns | Low | Reporting Bias; Small Number of Studies; Indirectness | Not upgraded |
| Tolerability | Aducanumab vs. Donanemab | 0 | Some concerns | No concerns | Major concerns | No concerns | No concerns | Low | Reporting Bias; Small Number of Studies; Indirectness | Not upgraded |
| ARIA-E | Aducanumab vs. Donanemab | 0 | Some concerns | No concerns | Major concerns | No concerns | No concerns | Very Low | Reporting Bias; Small Number of Studies; Indirectness | Not upgraded |
| ARIA-H | Aducanumab vs. Donanemab | 0 | Some concerns | No concerns | Major concerns | No concerns | No concerns | Very Low | Reporting Bias; Small Number of Patients; Indirectness | Not upgraded |
| Diarrhea | Aducanumab vs. Donanemab | 0 | Some concerns | No concerns | Major concerns | No concerns | No concerns | Very Low | Reporting Bias; Indirectness; Small Number of Studies | Not upgraded |
| Dizziness | Aducanumab vs. Donanemab | 0 | Some concerns | No concerns | Major concerns | No concerns | No concerns | Very Low | Reporting Bias; Small Number of Studies; Indirectness | Not upgraded |
| MMSE | Aducanumab vs. Donanemab | 0 | Some concerns | No concerns | Major concerns | Major concerns | No concerns | Very Low | Reporting Bias; Imprecision; Indirectness | Not upgraded |
| Fall | Aducanumab vs. Donanemab | 0 | Some concerns | No concerns | Major concerns | No concerns | No concerns | Low | Reporting Bias; Small Number of Studies; Heterogeneity; Indirectness | Not upgraded |
| ADAS-Cog | Aducanumab vs. Donanemab | 0 | Some concerns | No concerns | Major concerns | Major concerns | No concerns | Very Low | Reporting Bias; Imprecision; Indirectness | Not upgraded |
| CDR-SB | Aducanumab vs. Donanemab | 0 | Some concerns | No concerns | Major concerns | Major concerns | No concerns | Very Low | Reporting Bias; Imprecision; Indirectness; Small Number of Studies | Not upgraded |
| Amyloid Burden on PET | Aducanumab vs. Lecanemab | 0 | Some concerns | No concerns | Major concerns | Major concerns | No concerns | Low | Reporting Bias; Imprecision; Indirectness | Not upgraded |
| ARIA-E | Aducanumab vs. Solanezumab | 0 | Some concerns | No concerns | Major concerns | No concerns | No concerns | Low | Reporting Bias; Heterogeneity; Indirectness | Not upgraded |
| Dizziness | Aducanumab vs. Solanezumab | 0 | Some concerns | No concerns | Major concerns | No concerns | No concerns | Low | Reporting Bias; Imprecision; Indirectness | Not upgraded |
| Headaches | Aducanumab vs. Solanezumab | 0 | Some concerns | No concerns | Major concerns | No concerns | No concerns | Low | Reporting Bias; Imprecision; Indirectness | Not upgraded |
| Serious Side Effects | Aducanumab vs. Solanezumab | 0 | Some concerns | No concerns | Major concerns | No concerns | No concerns | Low | Reporting Bias; Imprecision; Indirectness | Not upgraded |
| Tolerability | Aducanumab vs. Solanezumab | 0 | Some concerns | No concerns | Major concerns | No concerns | No concerns | Low | Reporting Bias; Imprecision; Indirectness | Not upgraded |
| ADAS-Cog | Aducanumab vs. Solanezumab | 0 | Some concerns | No concerns | Major concerns | Major concerns | No concerns | Very Low | Reporting Bias; Imprecision; Indirectness | Not upgraded |
| ARIA-H | Aducanumab vs. Solanezumab | 0 | Some concerns | No concerns | Major concerns | No concerns | No concerns | Very Low | Reporting Bias; Imprecision; Indirectness | Not upgraded |
| CSF Aβ 1-42 | Aducanumab vs. Solanezumab | 0 | Some concerns | No concerns | Major concerns | Major concerns | No concerns | Very Low | Reporting Bias; Imprecision; Indirectness | Not upgraded |
| MMSE | Aducanumab vs. Solanezumab | 0 | Some concerns | No concerns | Major concerns | Major concerns | No concerns | Very Low | Reporting Bias; Imprecision; Indirectness | Not upgraded |
| Nasopharynghitis | Aducanumab vs. Solanezumab | 0 | Some concerns | No concerns | Major concerns | Major concerns | No concerns | Very Low | Reporting Bias; Imprecision; Indirectness | Not upgraded |
| Diarrhea | Aducanumab vs. Solanezumab | 0 | Some concerns | No concerns | Major concerns | No concerns | No concerns | Low | Reporting Bias; Imprecision; Indirectness | Not upgraded |
| Fall | Aducanumab vs. Solanezumab | 0 | Some concerns | No concerns | Major concerns | No concerns | No concerns | Low | Reporting Bias; Heterogeneity; Indirectness | Not upgraded |
| CDR-SB | Aducanumab vs. Solanezumab | 0 | Some concerns | No concerns | Major concerns | Major concerns | No concerns | Very Low | Reporting Bias; Imprecision; Indirectness | Not upgraded |
| Nasopharynghitis | Crenezumab vs. Aducanumab | 0 | Some concerns | No concerns | Major concerns | No concerns | No concerns | Very Low | Reporting Bias; Imprecision; Indirectness; Small Number of studies; Heterogeneity | Not upgraded |
| ADAS-Cog | Crenezumab vs. Aducanumab | 0 | Some concerns | No concerns | Major concerns | Major concerns | No concerns | Very Low | Reporting Bias; Imprecision; Indirectness; Small Number of studies | Not upgraded |
| ARIA-E | Crenezumab vs. Aducanumab | 0 | Some concerns | No concerns | Major concerns | No concerns | No concerns | Very Low | Reporting Bias; Indirectness; Small Number of studies; Imprecision | Not upgraded |
| ARIA-H | Crenezumab vs. Aducanumab | 0 | Some concerns | No concerns | Major concerns | No concerns | No concerns | Very Low | Reporting Bias; Indirectness; Small Number of studies | Not upgraded |
| CSF Aβ 1-42 | Crenezumab vs. Aducanumab | 0 | Some concerns | No concerns | Major concerns | Major concerns | No concerns | Very Low | Reporting Bias; Imprecision; Indirectness; Small Number of studies | Not upgraded |
| Diarrhea | Crenezumab vs. Aducanumab | 0 | Some concerns | No concerns | Major concerns | No concerns | No concerns | Very Low | Reporting Bias; Indirectness; Small Number of studies | Not upgraded |
| Dizziness | Crenezumab vs. Aducanumab | 0 | Some concerns | No concerns | Major concerns | No concerns | No concerns | Very Low | Reporting Bias; Indirectness; Small Number of studies | Not upgraded |
| Headaches | Crenezumab vs. Aducanumab | 0 | Some concerns | No concerns | Major concerns | No concerns | No concerns | Very Low | Reporting Bias; Indirectness; Small Number of studies | Not upgraded |
| MMSE | Crenezumab vs. Aducanumab | 0 | Some concerns | No concerns | Major concerns | Major concerns | No concerns | Very Low | Reporting Bias; Imprecision; Indirectness; Small Number of studies | Not upgraded |
| Serious Side Effects | Crenezumab vs. Aducanumab | 0 | Some concerns | No concerns | Major concerns | No concerns | No concerns | Very Low | Reporting Bias; Indirectness; Small Number of studies | Not upgraded |
| Tolerability | Crenezumab vs. Aducanumab | 0 | Some concerns | No concerns | Major concerns | No concerns | No concerns | Very Low | Reporting Bias; Indirectness; Small Number of studies | Not upgraded |
| CDR-SB | Crenezumab vs. Aducanumab | 0 | Some concerns | No concerns | Major concerns | Major concerns | No concerns | Very Low | Reporting Bias; Imprecision; Indirectness; Small Number of studies | Not upgraded |
| CSF p-Tau | Crenezumab vs. Aducanumab | 0 | Some concerns | No concerns | Major concerns | Major concerns | No concerns | Very Low | Reporting Bias; Imprecision; Indirectness; Small Number of studies | Not upgraded |
| Fall | Crenezumab vs. Aducanumab | 0 | Some concerns | No concerns | Major concerns | No concerns | No concerns | Very Low | Reporting Bias; Indirectness; Small Number of studies; Heterogeneity | Not upgraded |
| Infusion-related reactions | Crenezumab vs. Bapineuzumab | 0 | Some concerns | No concerns | Major concerns | No concerns | No concerns | Low | Small Number of Studies, Imprecision | Not upgraded |
| Headaches | Crenezumab vs. Bapineuzumab | 0 | Some concerns | Major concerns | Major concerns | No concerns | No concerns | Very Low | Reporting Bias; Indirectness; Small Number of studies; Heterogeneity | Not upgraded |
| ADAS-Cog | Crenezumab vs. Bapineuzumab | 0 | Some concerns | No concerns | Major concerns | Major concerns | No concerns | Very Low | Reporting Bias; Imprecision; Indirectness; Small Number of studies | Not upgraded |
| ARIA-E | Crenezumab vs. Bapineuzumab | 0 | Some concerns | No concerns | Major concerns | No concerns | No concerns | Very Low | Reporting Bias; Indirectness; Small Number of studies; Imprecision | Not upgraded |
| Back Pain | Crenezumab vs. Bapineuzumab | 0 | Some concerns | No concerns | Major concerns | No concerns | No concerns | Very Low | Reporting Bias; Indirectness; Small Number of studies | Not upgraded |
| CDR-SB | Crenezumab vs. Bapineuzumab | 0 | Some concerns | No concerns | Major concerns | Major concerns | No concerns | Very Low | Reporting Bias; Imprecision; Indirectness; Small Number of studies | Not upgraded |
| CSF p-Tau | Crenezumab vs. Bapineuzumab | 0 | Some concerns | No concerns | Major concerns | Major concerns | No concerns | Very Low | Reporting Bias; Imprecision; Indirectness; Small Number of studies | Not upgraded |
| Fall | Crenezumab vs. Bapineuzumab | 0 | Some concerns | No concerns | Major concerns | No concerns | No concerns | Very Low | Reporting Bias; Indirectness; Small Number of studies | Not upgraded |
| Serious Side Effects | Crenezumab vs. Bapineuzumab | 0 | Some concerns | No concerns | Major concerns | No concerns | No concerns | Very Low | Reporting Bias; Indirectness; Small Number of studies; Heterogeneity | Not upgraded |
| Tolerability | Crenezumab vs. Bapineuzumab | 0 | Some concerns | No concerns | Major concerns | No concerns | No concerns | Very Low | Reporting Bias; Indirectness; Small Number of studies | Not upgraded |
| Upper respiratory Infections | Crenezumab vs. Bapineuzumab | 0 | Some concerns | No concerns | Major concerns | No concerns | No concerns | Very Low | Reporting Bias; Indirectness; Small Number of studies | Not upgraded |
| Diarrhea | Crenezumab vs. Bapineuzumab | 0 | Some concerns | No concerns | Major concerns | No concerns | No concerns | Very Low | Reporting Bias; Indirectness; Small Number of studies | Not upgraded |
| Dizziness | Crenezumab vs. Bapineuzumab | 0 | Some concerns | No concerns | Major concerns | No concerns | No concerns | Very Low | Reporting Bias; Indirectness; Small Number of studies | Not upgraded |
| MMSE | Crenezumab vs. Bapineuzumab | 0 | Some concerns | No concerns | Major concerns | Major concerns | No concerns | Very Low | Reporting Bias; Imprecision; Indirectness; Small Number of studies | Not upgraded |
| Nasopharynghitis | Crenezumab vs. Bapineuzumab | 0 | Some concerns | No concerns | Major concerns | No concerns | No concerns | Very Low | Reporting Bias; Imprecision; Indirectness; Small Number of studies; Heterogeneity | Not upgraded |
| Infusion-related reactions | Crenezumab vs. Donanemab | 0 | Some concerns | No concerns | Major concerns | No concerns | No concerns | Low | Small Number of Studies, Imprecision | Not upgraded |
| Nasopharynghitis | Crenezumab vs. Donanemab | 0 | Some concerns | No concerns | Major concerns | No concerns | No concerns | Very Low | Reporting Bias; Imprecision; Indirectness; Small Number of studies; Heterogeneity | Not upgraded |
| CDR-SB | Crenezumab vs. Donanemab | 0 | Some concerns | No concerns | Major concerns | Major concerns | No concerns | Very Low | Reporting Bias; Imprecision; Indirectness; Small Number of studies | Not upgraded |
| Diarrhea | Crenezumab vs. Donanemab | 0 | Some concerns | No concerns | Major concerns | No concerns | No concerns | Very Low | Reporting Bias; Indirectness; Small Number of studies | Not upgraded |
| Dizziness | Crenezumab vs. Donanemab | 0 | Some concerns | No concerns | Major concerns | No concerns | No concerns | Very Low | Reporting Bias; Indirectness; Small Number of studies | Not upgraded |
| MMSE | Crenezumab vs. Donanemab | 0 | Some concerns | No concerns | Major concerns | Major concerns | No concerns | Very Low | Reporting Bias; Imprecision; Indirectness; Small Number of studies | Not upgraded |
| ADAS-Cog | Crenezumab vs. Donanemab | 0 | Some concerns | No concerns | Major concerns | Major concerns | No concerns | Very Low | Reporting Bias; Imprecision; Indirectness; Small Number of studies | Not upgraded |
| ARIA-E | Crenezumab vs. Donanemab | 0 | Some concerns | No concerns | Major concerns | No concerns | No concerns | Very Low | Reporting Bias; Indirectness; Small Number of studies; Heterogeneity; Imprecision | Not upgraded |
| ARIA-H | Crenezumab vs. Donanemab | 0 | Some concerns | No concerns | Major concerns | No concerns | No concerns | Very Low | Reporting Bias; Indirectness; Small Number of studies | Not upgraded |
| CSF Aβ 1-42 | Crenezumab vs. Donanemab | 0 | Some concerns | No concerns | Major concerns | Major concerns | No concerns | Very Low | Reporting Bias; Imprecision; Indirectness; Small Number of studies | Not upgraded |
| Fall | Crenezumab vs. Donanemab | 0 | Some concerns | No concerns | Major concerns | No concerns | No concerns | Very Low | Reporting Bias; Indirectness; Small Number of studies | Not upgraded |
| Headaches | Crenezumab vs. Donanemab | 0 | Some concerns | No concerns | Major concerns | No concerns | No concerns | Very Low | Reporting Bias; Indirectness; Small Number of studies | Not upgraded |
| Serious Side Effects | Crenezumab vs. Donanemab | 0 | Some concerns | No concerns | Major concerns | No concerns | No concerns | Very Low | Reporting Bias; Indirectness; Small Number of studies | Not upgraded |
| Tolerability | Crenezumab vs. Donanemab | 0 | Some concerns | No concerns | Major concerns | No concerns | No concerns | Very Low | Reporting Bias; Indirectness; Small Number of studies | Not upgraded |
| Infusion-related reactions | Crenezumab vs. Lecanemab | 0 | Some concerns | No concerns | Major concerns | No concerns | No concerns | Low | Imprecision | Not upgraded |
| CSF Aβ 1-42 | Crenezumab vs. Lecanemab | 0 | Some concerns | No concerns | Major concerns | Major concerns | No concerns | Very Low | Reporting Bias; Imprecision; Indirectness; Small Number of studies; Heterogeneity | Not upgraded |
| CSF p-Tau | Crenezumab vs. Lecanemab | 0 | Some concerns | No concerns | Major concerns | Major concerns | No concerns | Very Low | Reporting Bias; Imprecision; Indirectness; Small Number of studies; Heterogeneity | Not upgraded |
| Nasopharynghitis | Crenezumab vs. Lecanemab | 0 | Some concerns | No concerns | Major concerns | No concerns | No concerns | Very Low | Reporting Bias; Imprecision; Indirectness; Small Number of studies; Heterogeneity | Not upgraded |
| ADAS-Cog | Crenezumab vs. Lecanemab | 0 | Some concerns | No concerns | Major concerns | Major concerns | No concerns | Very Low | Reporting Bias; Imprecision; Indirectness; Small Number of studies | Not upgraded |
| ARIA-E | Crenezumab vs. Lecanemab | 0 | Some concerns | No concerns | Major concerns | No concerns | No concerns | Very Low | Reporting Bias; Indirectness; Small Number of studies; Imprecision | Not upgraded |
| ARIA-H | Crenezumab vs. Lecanemab | 0 | Some concerns | No concerns | Major concerns | No concerns | No concerns | Very Low | Reporting Bias; Indirectness; Small Number of studies | Not upgraded |
| Back Pain | Crenezumab vs. Lecanemab | 0 | Some concerns | No concerns | Major concerns | No concerns | No concerns | Very Low | Reporting Bias; Indirectness; Small Number of studies | Not upgraded |
| CDR-SB | Crenezumab vs. Lecanemab | 0 | Some concerns | No concerns | Major concerns | Major concerns | No concerns | Very Low | Reporting Bias; Imprecision; Indirectness; Small Number of studies | Not upgraded |
| Diarrhea | Crenezumab vs. Lecanemab | 0 | Some concerns | No concerns | Major concerns | No concerns | No concerns | Very Low | Reporting Bias; Indirectness; Small Number of studies | Not upgraded |
| Dizziness | Crenezumab vs. Lecanemab | 0 | Some concerns | No concerns | Major concerns | No concerns | No concerns | Very Low | Reporting Bias; Indirectness; Small Number of studies | Not upgraded |
| Fall | Crenezumab vs. Lecanemab | 0 | Some concerns | No concerns | Major concerns | No concerns | No concerns | Very Low | Reporting Bias; Indirectness; Small Number of studies | Not upgraded |
| Headaches | Crenezumab vs. Lecanemab | 0 | Some concerns | No concerns | Major concerns | No concerns | No concerns | Very Low | Reporting Bias; Indirectness; Small Number of studies | Not upgraded |
| MMSE | Crenezumab vs. Lecanemab | 0 | Some concerns | No concerns | Major concerns | Major concerns | No concerns | Very Low | Reporting Bias; Imprecision; Indirectness; Small Number of studies | Not upgraded |
| Serious Side Effects | Crenezumab vs. Lecanemab | 0 | Some concerns | No concerns | Major concerns | No concerns | No concerns | Very Low | Reporting Bias; Indirectness; Small Number of studies | Not upgraded |
| Tolerability | Crenezumab vs. Lecanemab | 0 | Some concerns | No concerns | Major concerns | No concerns | No concerns | Very Low | Reporting Bias; Indirectness; Small Number of studies | Not upgraded |
| Nasopharynghitis | Crenezumab vs. Solanezumab | 0 | Some concerns | No concerns | Major concerns | No concerns | No concerns | Very Low | Reporting Bias; Imprecision; Indirectness; Small Number of studies | Not upgraded |
| Upper respiratory Infections | Crenezumab vs. Solanezumab | 0 | Some concerns | No concerns | Major concerns | No concerns | No concerns | Very Low | Reporting Bias; Indirectness; Small Number of studies; Heterogeneity | Not upgraded |
| ADAS-Cog | Crenezumab vs. Solanezumab | 0 | Some concerns | No concerns | Major concerns | Major concerns | No concerns | Very Low | Reporting Bias; Imprecision; Indirectness; Small Number of studies | Not upgraded |
| ARIA-E | Crenezumab vs. Solanezumab | 0 | Some concerns | No concerns | Major concerns | No concerns | No concerns | Very Low | Reporting Bias; Indirectness; Small Number of studies; Imprecision | Not upgraded |
| ARIA-H | Crenezumab vs. Solanezumab | 0 | Some concerns | No concerns | Major concerns | No concerns | No concerns | Very Low | Reporting Bias; Indirectness; Small Number of studies | Not upgraded |
| Back Pain | Crenezumab vs. Solanezumab | 0 | Some concerns | No concerns | Major concerns | No concerns | No concerns | Very Low | Reporting Bias; Indirectness; Small Number of studies | Not upgraded |
| CSF Aβ 1-42 | Crenezumab vs. Solanezumab | 0 | Some concerns | No concerns | Major concerns | Major concerns | No concerns | Very Low | Reporting Bias; Imprecision; Indirectness; Small Number of studies | Not upgraded |
| Diarrhea | Crenezumab vs. Solanezumab | 0 | Some concerns | No concerns | Major concerns | No concerns | No concerns | Very Low | Reporting Bias; Indirectness; Small Number of studies | Not upgraded |
| Dizziness | Crenezumab vs. Solanezumab | 0 | Some concerns | No concerns | Major concerns | No concerns | No concerns | Very Low | Reporting Bias; Indirectness; Small Number of studies | Not upgraded |
| Fall | Crenezumab vs. Solanezumab | 0 | Some concerns | No concerns | Major concerns | No concerns | No concerns | Very Low | Reporting Bias; Indirectness; Small Number of studies | Not upgraded |
| Headaches | Crenezumab vs. Solanezumab | 0 | Some concerns | No concerns | Major concerns | No concerns | No concerns | Very Low | Reporting Bias; Indirectness; Small Number of studies | Not upgraded |
| MMSE | Crenezumab vs. Solanezumab | 0 | Some concerns | No concerns | Major concerns | Major concerns | No concerns | Very Low | Reporting Bias; Imprecision; Indirectness; Small Number of studies | Not upgraded |
| Serious Side Effects | Crenezumab vs. Solanezumab | 0 | Some concerns | No concerns | Major concerns | No concerns | No concerns | Very Low | Reporting Bias; Indirectness; Small Number of studies | Not upgraded |
| Tolerability | Crenezumab vs. Solanezumab | 0 | Some concerns | No concerns | Major concerns | No concerns | No concerns | Very Low | Reporting Bias; Indirectness; Small Number of studies | Not upgraded |
| CDR-SB | Crenezumab vs. Solanezumab | 0 | Some concerns | No concerns | Major concerns | Major concerns | No concerns | Very Low | Reporting Bias; Imprecision; Indirectness; Small Number of studies | Not upgraded |
| Arthralgia | Donanemab vs. Bapineuzumab | 0 | Some concerns | No concerns | Major concerns | No concerns | No concerns | Low | Reporting Bias; Indirectness | Not upgraded |
| Infusion-related reactions | Donanemab vs. Bapineuzumab | 0 | Some concerns | No concerns | Major concerns | No concerns | No concerns | Low | Small Number of Studies, Imprecision | Not upgraded |
| Urinary Infections | Donanemab vs. Bapineuzumab | 0 | Some concerns | Some concerns | Major concerns | No concerns | No concerns | Low | Reporting Bias; Small Number of Studies; Heterogeneity; Indirectness | Not upgraded |
| Headaches | Donanemab vs. Bapineuzumab | 0 | Some concerns | Major concerns | Major concerns | No concerns | No concerns | Very Low | Reporting Bias; Small Number of Studies; Heterogeneity | Not upgraded |
| ARIA-E | Donanemab vs. Bapineuzumab | 0 | Some concerns | No concerns | Major concerns | No concerns | No concerns | Low | Reporting Bias; Small Number of Studies; Indirectness | Not upgraded |
| ARIA-E in APOE-ε4 Carriers | Donanemab vs. Bapineuzumab | 0 | Some concerns | No concerns | Major concerns | No concerns | No concerns | Low | Reporting Bias; Small Number of Patients; Indirectness | Not upgraded |
| ARIA-E in APOE-ε4 Non-Carriers | Donanemab vs. Bapineuzumab | 0 | Some concerns | No concerns | Major concerns | No concerns | No concerns | Low | Reporting Bias; Small Number of Patients; Indirectness | Not upgraded |
| Nausea | Donanemab vs. Bapineuzumab | 0 | Some concerns | No concerns | Major concerns | No concerns | No concerns | Low | Reporting Bias; Small Number of Studies; Indirectness | Not upgraded |
| Tolerability | Donanemab vs. Bapineuzumab | 0 | Some concerns | No concerns | Major concerns | No concerns | No concerns | Low | Reporting Bias; Small Number of Studies; Indirectness | Not upgraded |
| ADAS-Cog | Donanemab vs. Bapineuzumab | 0 | Some concerns | No concerns | Major concerns | Major concerns | No concerns | Very Low | Reporting Bias; Imprecision; Indirectness | Not upgraded |
| Back Pain | Donanemab vs. Bapineuzumab | 0 | Some concerns | No concerns | Major concerns | No concerns | No concerns | Very Low | Reporting Bias; Indirectness; Small Number of Studies | Not upgraded |
| CDR-SB | Donanemab vs. Bapineuzumab | 0 | Some concerns | No concerns | Major concerns | Major concerns | No concerns | Very Low | Reporting Bias; Imprecision; Indirectness; Small Number of Studies | Not upgraded |
| Fall | Donanemab vs. Bapineuzumab | 0 | Some concerns | No concerns | Major concerns | No concerns | No concerns | Very Low | Reporting Bias; Small Number of Studies; Indirectness | Not upgraded |
| Serious Side Effects | Donanemab vs. Bapineuzumab | 0 | Some concerns | No concerns | Major concerns | No concerns | No concerns | Very Low | Reporting Bias; Indirectness; Small Number of Studies; Heterogeneity | Not upgraded |
| Diarrhea | Donanemab vs. Bapineuzumab | 0 | Some concerns | No concerns | Major concerns | No concerns | No concerns | Low | Reporting Bias; Small Number of Studies; Indirectness | Not upgraded |
| Dizziness | Donanemab vs. Bapineuzumab | 0 | Some concerns | No concerns | Major concerns | No concerns | No concerns | Low | Reporting Bias; Small Number of Studies; Indirectness | Not upgraded |
| MMSE | Donanemab vs. Bapineuzumab | 0 | Some concerns | No concerns | Major concerns | Major concerns | No concerns | Very Low | Reporting Bias; Imprecision; Small Number of Studies | Not upgraded |
| Infusion-related reactions | Donanemab vs. Lecanemab | 0 | Some concerns | No concerns | Major concerns | No concerns | No concerns | Low | Imprecision | Not upgraded |
| Amyloid Burden on PET | Donanemab vs. Lecanemab | 0 | Some concerns | Major concerns | Major concerns | Major concerns | No concerns | Very Low | Reporting Bias; Imprecision; Indirectness; Heterogeneity | Not upgraded |
| ARIA-E | Donanemab vs. Solanezumab | 0 | Some concerns | No concerns | Major concerns | No concerns | No concerns | Low | Reporting Bias; Small Number of Studies; Indirectness | Not upgraded |
| Arthralgia | Donanemab vs. Solanezumab | 0 | Some concerns | No concerns | Major concerns | No concerns | No concerns | Low | Reporting Bias; Indirectness | Not upgraded |
| Dizziness | Donanemab vs. Solanezumab | 0 | Some concerns | No concerns | Major concerns | No concerns | No concerns | Low | Reporting Bias; Small Number of Studies; Indirectness | Not upgraded |
| Fall | Donanemab vs. Solanezumab | 0 | Some concerns | No concerns | Major concerns | No concerns | No concerns | Low | Reporting Bias; Small Number of Studies; Indirectness | Not upgraded |
| Headaches | Donanemab vs. Solanezumab | 0 | Some concerns | No concerns | Major concerns | No concerns | No concerns | Low | Reporting Bias; Small Number of Studies; Indirectness | Not upgraded |
| Nausea | Donanemab vs. Solanezumab | 0 | Some concerns | No concerns | Major concerns | No concerns | No concerns | Low | Reporting Bias; Small Number of Studies; Indirectness | Not upgraded |
| Serious Side Effects | Donanemab vs. Solanezumab | 0 | Some concerns | No concerns | Major concerns | No concerns | No concerns | Low | Reporting Bias; Imprecision; Indirectness | Not upgraded |
| Tolerability | Donanemab vs. Solanezumab | 0 | Some concerns | No concerns | Major concerns | No concerns | No concerns | Low | Reporting Bias; Small Number of Studies; Indirectness | Not upgraded |
| Urinary Infections | Donanemab vs. Solanezumab | 0 | Some concerns | No concerns | Major concerns | No concerns | No concerns | Low | Reporting Bias; Small Number of Studies; Heterogeneity; Indirectness | Not upgraded |
| ADAS-Cog | Donanemab vs. Solanezumab | 0 | Some concerns | No concerns | Major concerns | Major concerns | No concerns | Very Low | Reporting Bias; Imprecision; Indirectness | Not upgraded |
| ARIA-H | Donanemab vs. Solanezumab | 0 | Some concerns | No concerns | Major concerns | No concerns | No concerns | Very Low | Reporting Bias; Small Number of Studies; Indirectness | Not upgraded |
| Back Pain | Donanemab vs. Solanezumab | 0 | Some concerns | No concerns | Major concerns | No concerns | No concerns | Very Low | Reporting Bias; Indirectness Small Number of Studies | Not upgraded |
| MMSE | Donanemab vs. Solanezumab | 0 | Some concerns | No concerns | Major concerns | Major concerns | No concerns | Very Low | Reporting Bias; Imprecision; Small Number of Studies; Indirectness | Not upgraded |
| Diarrhea | Donanemab vs. Solanezumab | 0 | Some concerns | No concerns | Major concerns | No concerns | No concerns | Low | Reporting Bias; Small Number of Studies; Indirectness | Not upgraded |
| Fatigue | Donanemab vs. Solanezumab | 0 | Some concerns | No concerns | Major concerns | No concerns | No concerns | Low | Reporting Bias; Small Number of Studies; Heterogeneity; Indirectness | Not upgraded |
| CDR-SB | Donanemab vs. Solanezumab | 0 | Some concerns | No concerns | Major concerns | Major concerns | No concerns | Very Low | Reporting Bias; Imprecision; Indirectness; Small Number of Studies | Not upgraded |
| ADAS-Cog | Gantenerumab vs. Aducanumab | 0 | Some concerns | No concerns | Major concerns | Major concerns | No concerns | Very Low | Reporting Bias; Imprecision; Indirectness; Small Number of studies | Not upgraded |
| Amyloid Burden on PET | Gantenerumab vs. Aducanumab | 0 | Some concerns | No concerns | Major concerns | Major concerns | No concerns | Very Low | Reporting Bias; Imprecision; Indirectness; Small Number of studies | Not upgraded |
| ARIA-E in APOE-ε4 Carriers | Gantenerumab vs. Aducanumab | 0 | Some concerns | No concerns | Major concerns | No concerns | No concerns | Very Low | Reporting Bias; Indirectness; Small Number of Patients | Not upgraded |
| ARIA-H | Gantenerumab vs. Aducanumab | 0 | Some concerns | No concerns | Major concerns | No concerns | No concerns | Very Low | Reporting Bias; Indirectness; Small Number of studies; Heterogeneity | Not upgraded |
| Diarrhea | Gantenerumab vs. Aducanumab | 0 | Some concerns | No concerns | Major concerns | No concerns | No concerns | Very Low | Reporting Bias; Indirectness; Small Number of studies | Not upgraded |
| Dizziness | Gantenerumab vs. Aducanumab | 0 | Some concerns | No concerns | Major concerns | No concerns | No concerns | Very Low | Reporting Bias; Indirectness; Small Number of studies | Not upgraded |
| Headaches | Gantenerumab vs. Aducanumab | 0 | Some concerns | No concerns | Major concerns | No concerns | No concerns | Very Low | Reporting Bias; Indirectness; Small Number of studies | Not upgraded |
| Nasopharynghitis | Gantenerumab vs. Aducanumab | 0 | Some concerns | No concerns | Major concerns | No concerns | No concerns | Very Low | Reporting Bias; Imprecision; Indirectness; Small Number of studies | Not upgraded |
| Serious Side Effects | Gantenerumab vs. Aducanumab | 0 | Some concerns | No concerns | Major concerns | No concerns | No concerns | Very Low | Reporting Bias; Indirectness; Small Number of studies | Not upgraded |
| Tolerability | Gantenerumab vs. Aducanumab | 0 | Some concerns | No concerns | Major concerns | No concerns | No concerns | Very Low | Reporting Bias; Indirectness; Small Number of studies | Not upgraded |
| ARIA-E | Gantenerumab vs. Aducanumab | 0 | Some concerns | No concerns | Major concerns | No concerns | No concerns | Very Low | Reporting Bias; Indirectness; Small Number of studies; Heterogeneity | Not upgraded |
| ARIA-E in APOE-ε4 Non-Carriers | Gantenerumab vs. Aducanumab | 0 | Some concerns | No concerns | Major concerns | No concerns | No concerns | Very Low | Reporting Bias; Indirectness; Small Number of Patients | Not upgraded |
| CDR-SB | Gantenerumab vs. Aducanumab | 0 | Some concerns | No concerns | Major concerns | Major concerns | No concerns | Very Low | Reporting Bias; Imprecision; Indirectness; Small Number of studies | Not upgraded |
| Fall | Gantenerumab vs. Aducanumab | 0 | Some concerns | No concerns | Major concerns | No concerns | No concerns | Very Low | Reporting Bias; Indirectness; Small Number of studies; Heterogeneity | Not upgraded |
| Urinary Infections | Gantenerumab vs. Bapineuzumab | 0 | Some concerns | No concerns | Major concerns | No concerns | No concerns | Low | Reporting Bias; Imprecision; Heterogeneity; Indirectness | Not upgraded |
| Arthralgia | Gantenerumab vs. Bapineuzumab | 0 | Some concerns | No concerns | Major concerns | No concerns | No concerns | Very Low | Reporting Bias; Indirectness; Small Number of studies | Not upgraded |
| ADAS-Cog | Gantenerumab vs. Bapineuzumab | 0 | Some concerns | No concerns | Major concerns | Major concerns | No concerns | Very Low | Reporting Bias; Imprecision; Indirectness; Small Number of studies | Not upgraded |
| Amyloid Burden on PET | Gantenerumab vs. Bapineuzumab | 0 | Some concerns | No concerns | Major concerns | Major concerns | No concerns | Very Low | Reporting Bias; Imprecision; Indirectness; Small Number of studies | Not upgraded |
| ARIA-E in APOE-ε4 Carriers | Gantenerumab vs. Bapineuzumab | 0 | Some concerns | No concerns | Major concerns | No concerns | No concerns | Very Low | Reporting Bias; Indirectness; Small Number of Patients | Not upgraded |
| CDR-SB | Gantenerumab vs. Bapineuzumab | 0 | Some concerns | No concerns | Major concerns | Major concerns | No concerns | Very Low | Reporting Bias; Imprecision; Indirectness; Small Number of studies | Not upgraded |
| Fall | Gantenerumab vs. Bapineuzumab | 0 | Some concerns | No concerns | Major concerns | No concerns | No concerns | Very Low | Reporting Bias; Indirectness; Small Number of studies | Not upgraded |
| Headaches | Gantenerumab vs. Bapineuzumab | 0 | Some concerns | No concerns | Major concerns | No concerns | No concerns | Very Low | Reporting Bias; Indirectness; Small Number of studies | Not upgraded |
| Serious Side Effects | Gantenerumab vs. Bapineuzumab | 0 | Some concerns | No concerns | Major concerns | No concerns | No concerns | Very Low | Reporting Bias; Indirectness; Small Number of studies; Heterogeneity | Not upgraded |
| Tolerability | Gantenerumab vs. Bapineuzumab | 0 | Some concerns | No concerns | Major concerns | No concerns | No concerns | Very Low | Reporting Bias; Indirectness; Small Number of studies | Not upgraded |
| ARIA-E | Gantenerumab vs. Bapineuzumab | 0 | Some concerns | No concerns | Major concerns | No concerns | No concerns | Very Low | Reporting Bias; Indirectness; Small Number of studies; Heterogeneity | Not upgraded |
| ARIA-E in APOE-ε4 Non-Carriers | Gantenerumab vs. Bapineuzumab | 0 | Some concerns | No concerns | Major concerns | No concerns | No concerns | Very Low | Reporting Bias; Indirectness; Small Number of Patients | Not upgraded |
| Diarrhea | Gantenerumab vs. Bapineuzumab | 0 | Some concerns | No concerns | Major concerns | No concerns | No concerns | Very Low | Reporting Bias; Indirectness; Small Number of studies | Not upgraded |
| Dizziness | Gantenerumab vs. Bapineuzumab | 0 | Some concerns | No concerns | Major concerns | No concerns | No concerns | Very Low | Reporting Bias; Indirectness; Small Number of studies | Not upgraded |
| Nasopharynghitis | Gantenerumab vs. Bapineuzumab | 0 | Some concerns | No concerns | Major concerns | No concerns | No concerns | Very Low | Reporting Bias; Imprecision; Indirectness; Small Number of studies | Not upgraded |
| ADAS-Cog | Gantenerumab vs. Crenezumab | 0 | Some concerns | No concerns | Major concerns | Major concerns | No concerns | Very Low | Reporting Bias; Imprecision; Indirectness; Small Number of studies | Not upgraded |
| ARIA-H | Gantenerumab vs. Crenezumab | 0 | Some concerns | No concerns | Major concerns | No concerns | No concerns | Very Low | Reporting Bias; Indirectness; Small Number of studies; Heterogeneity | Not upgraded |
| CDR-SB | Gantenerumab vs. Crenezumab | 0 | Some concerns | No concerns | Major concerns | Major concerns | No concerns | Very Low | Reporting Bias; Imprecision; Indirectness; Small Number of studies | Not upgraded |
| Diarrhea | Gantenerumab vs. Crenezumab | 0 | Some concerns | No concerns | Major concerns | No concerns | No concerns | Very Low | Reporting Bias; Indirectness; Small Number of studies | Not upgraded |
| Dizziness | Gantenerumab vs. Crenezumab | 0 | Some concerns | No concerns | Major concerns | No concerns | No concerns | Very Low | Reporting Bias; Indirectness; Small Number of studies | Not upgraded |
| Fall | Gantenerumab vs. Crenezumab | 0 | Some concerns | No concerns | Major concerns | No concerns | No concerns | Very Low | Reporting Bias; Indirectness; Small Number of studies | Not upgraded |
| Headaches | Gantenerumab vs. Crenezumab | 0 | Some concerns | No concerns | Major concerns | No concerns | No concerns | Very Low | Reporting Bias; Indirectness; Small Number of studies | Not upgraded |
| Nasopharynghitis | Gantenerumab vs. Crenezumab | 0 | Some concerns | No concerns | Major concerns | No concerns | No concerns | Very Low | Reporting Bias; Imprecision; Indirectness; Small Number of studies | Not upgraded |
| Serious Side Effects | Gantenerumab vs. Crenezumab | 0 | Some concerns | No concerns | Major concerns | No concerns | No concerns | Very Low | Reporting Bias; Indirectness; Small Number of studies | Not upgraded |
| Tolerability | Gantenerumab vs. Crenezumab | 0 | Some concerns | No concerns | Major concerns | No concerns | No concerns | Very Low | Reporting Bias; Indirectness; Small Number of studies | Not upgraded |
| ARIA-E | Gantenerumab vs. Crenezumab | 0 | Some concerns | No concerns | Major concerns | No concerns | No concerns | Very Low | Reporting Bias; Indirectness; Small Number of studies; Heterogeneity | Not upgraded |
| Urinary Infections | Gantenerumab vs. Donanemab | 0 | Some concerns | No concerns | Major concerns | No concerns | No concerns | Low | Reporting Bias; Small Number of Studies; Indirectness; Heterogeneity | Not upgraded |
| Amyloid Burden on PET | Gantenerumab vs. Donanemab | 0 | Some concerns | Major concerns | Major concerns | Major concerns | No concerns | Very Low | Reporting Bias; Imprecision; Heterogeneity; Indirectness; Small Number of studies | Not upgraded |
| Arthralgia | Gantenerumab vs. Donanemab | 0 | Some concerns | No concerns | Major concerns | No concerns | No concerns | Very Low | Reporting Bias; Indirectedness | Not upgraded |
| CDR-SB | Gantenerumab vs. Donanemab | 0 | Some concerns | No concerns | Major concerns | Major concerns | No concerns | Very Low | Reporting Bias; Imprecision; Indirectness; Small Number of studies | Not upgraded |
| ADAS-Cog | Gantenerumab vs. Donanemab | 0 | Some concerns | No concerns | Major concerns | Major concerns | No concerns | Very Low | Reporting Bias; Imprecision; Indirectness; Small Number of studies | Not upgraded |
| ARIA-E | Gantenerumab vs. Donanemab | 0 | Some concerns | No concerns | Major concerns | No concerns | No concerns | Very Low | Reporting Bias; Indirectness; Small Number of studies; Heterogeneity | Not upgraded |
| ARIA-E in APOE-ε4 Carriers | Gantenerumab vs. Donanemab | 0 | Some concerns | No concerns | Major concerns | No concerns | No concerns | Very Low | Reporting Bias; Indirectness; Small Number of Patients | Not upgraded |
| ARIA-E in APOE-ε4 Non-Carriers | Gantenerumab vs. Donanemab | 0 | Some concerns | No concerns | Major concerns | No concerns | No concerns | Very Low | Reporting Bias; Indirectness; Small Number of Patients | Not upgraded |
| ARIA-H | Gantenerumab vs. Donanemab | 0 | Some concerns | No concerns | Major concerns | No concerns | No concerns | Very Low | Reporting Bias; Indirectness; Small Number of studies; Heterogeneity | Not upgraded |
| Diarrhea | Gantenerumab vs. Donanemab | 0 | Some concerns | No concerns | Major concerns | No concerns | No concerns | Very Low | Reporting Bias; Indirectness; Small Number of studies | Not upgraded |
| Dizziness | Gantenerumab vs. Donanemab | 0 | Some concerns | No concerns | Major concerns | No concerns | No concerns | Very Low | Reporting Bias; Indirectness; Small Number of studies | Not upgraded |
| Fall | Gantenerumab vs. Donanemab | 0 | Some concerns | No concerns | Major concerns | No concerns | No concerns | Very Low | Reporting Bias; Indirectness; Small Number of studies | Not upgraded |
| Headaches | Gantenerumab vs. Donanemab | 0 | Some concerns | No concerns | Major concerns | No concerns | No concerns | Very Low | Reporting Bias; Indirectness; Small Number of studies | Not upgraded |
| Nasopharynghitis | Gantenerumab vs. Donanemab | 0 | Some concerns | No concerns | Major concerns | No concerns | No concerns | Very Low | Reporting Bias; Imprecision; Indirectness; Small Number of studies | Not upgraded |
| Serious Side Effects | Gantenerumab vs. Donanemab | 0 | Some concerns | No concerns | Major concerns | No concerns | No concerns | Very Low | Reporting Bias; Indirectness; Small Number of studies | Not upgraded |
| Tolerability | Gantenerumab vs. Donanemab | 0 | Some concerns | No concerns | Major concerns | No concerns | No concerns | Very Low | Reporting Bias; Indirectness; Small Number of studies | Not upgraded |
| Arthralgia | Gantenerumab vs. Lecanemab | 0 | Some concerns | No concerns | Major concerns | No concerns | No concerns | Low | Reporting Bias; Indirectness; Small Number of studies | Not upgraded |
| ADAS-Cog | Gantenerumab vs. Lecanemab | 0 | Some concerns | No concerns | Major concerns | Major concerns | No concerns | Very Low | Reporting Bias; Imprecision; Indirectness; Small Number of studies | Not upgraded |
| ARIA-E in APOE-ε4 Carriers | Gantenerumab vs. Lecanemab | 0 | Some concerns | No concerns | Major concerns | No concerns | No concerns | Very Low | Reporting Bias; Indirectness; Small Number of Patients | Not upgraded |
| ARIA-H | Gantenerumab vs. Lecanemab | 0 | Some concerns | No concerns | Major concerns | No concerns | No concerns | Very Low | Reporting Bias; Indirectness; Small Number of studies; Heterogeneity | Not upgraded |
| Back Pain | Gantenerumab vs. Lecanemab | 0 | Some concerns | No concerns | Major concerns | No concerns | No concerns | Very Low | Reporting Bias; Indirectness; Small Number of studies | Not upgraded |
| CDR-SB | Gantenerumab vs. Lecanemab | 0 | Some concerns | No concerns | Major concerns | Major concerns | No concerns | Very Low | Reporting Bias; Imprecision; Indirectness; Small Number of studies | Not upgraded |
| Diarrhea | Gantenerumab vs. Lecanemab | 0 | Some concerns | No concerns | Major concerns | No concerns | No concerns | Very Low | Reporting Bias; Indirectness; Small Number of studies | Not upgraded |
| Dizziness | Gantenerumab vs. Lecanemab | 0 | Some concerns | No concerns | Major concerns | No concerns | No concerns | Very Low | Reporting Bias; Indirectness; Small Number of studies | Not upgraded |
| Fall | Gantenerumab vs. Lecanemab | 0 | Some concerns | No concerns | Major concerns | No concerns | No concerns | Very Low | Reporting Bias; Indirectness; Small Number of studies | Not upgraded |
| Headaches | Gantenerumab vs. Lecanemab | 0 | Some concerns | No concerns | Major concerns | No concerns | No concerns | Very Low | Reporting Bias; Indirectness; Small Number of studies | Not upgraded |
| Serious Side Effects | Gantenerumab vs. Lecanemab | 0 | Some concerns | No concerns | Major concerns | No concerns | No concerns | Very Low | Reporting Bias; Indirectness; Small Number of studies | Not upgraded |
| Tolerability | Gantenerumab vs. Lecanemab | 0 | Some concerns | No concerns | Major concerns | No concerns | No concerns | Very Low | Reporting Bias; Indirectness; Small Number of studies | Not upgraded |
| Amyloid Burden on PET | Gantenerumab vs. Lecanemab | 0 | Some concerns | No concerns | Major concerns | Major concerns | No concerns | Very Low | Reporting Bias; Imprecision; Indirectness; Small Number of studies | Not upgraded |
| ARIA-E | Gantenerumab vs. Lecanemab | 0 | Some concerns | No concerns | Major concerns | No concerns | No concerns | Very Low | Reporting Bias; Indirectness; Small Number of studies; Heterogeneity | Not upgraded |
| ARIA-E in APOE-ε4 Non-Carriers | Gantenerumab vs. Lecanemab | 0 | Some concerns | No concerns | Major concerns | No concerns | No concerns | Very Low | Reporting Bias; Indirectness; Small Number of Patients | Not upgraded |
| Urinary Infections | Gantenerumab vs. Solanezumab | 0 | Some concerns | No concerns | Major concerns | No concerns | No concerns | Low | Reporting Bias; Imprecision; Indirectness | Not upgraded |
| ADAS-Cog | Gantenerumab vs. Solanezumab | 0 | Some concerns | No concerns | Major concerns | Major concerns | No concerns | Very Low | Reporting Bias; Imprecision; Indirectness; Small Number of studies | Not upgraded |
| ARIA-H | Gantenerumab vs. Solanezumab | 0 | Some concerns | No concerns | Major concerns | No concerns | No concerns | Very Low | Reporting Bias; Indirectness; Small Number of studies; Heterogeneity | Not upgraded |
| Arthralgia | Gantenerumab vs. Solanezumab | 0 | Some concerns | No concerns | Major concerns | No concerns | No concerns | Very Low | Reporting Bias; Indirectness; Small Number of studies | Not upgraded |
| Dizziness | Gantenerumab vs. Solanezumab | 0 | Some concerns | No concerns | Major concerns | No concerns | No concerns | Very Low | Reporting Bias; Indirectness; Small Number of studies | Not upgraded |
| Fall | Gantenerumab vs. Solanezumab | 0 | Some concerns | No concerns | Major concerns | No concerns | No concerns | Very Low | Reporting Bias; Indirectness; Small Number of studies | Not upgraded |
| Headaches | Gantenerumab vs. Solanezumab | 0 | Some concerns | No concerns | Major concerns | No concerns | No concerns | Very Low | Reporting Bias; Indirectness; Small Number of studies | Not upgraded |
| Serious Side Effects | Gantenerumab vs. Solanezumab | 0 | Some concerns | No concerns | Major concerns | No concerns | No concerns | Very Low | Reporting Bias; Indirectness; Small Number of studies | Not upgraded |
| Tolerability | Gantenerumab vs. Solanezumab | 0 | Some concerns | No concerns | Major concerns | No concerns | No concerns | Very Low | Reporting Bias; Indirectness; Small Number of studies | Not upgraded |
| Amyloid Burden on PET | Gantenerumab vs. Solanezumab | 0 | Some concerns | No concerns | Major concerns | Major concerns | No concerns | Very Low | Reporting Bias; Imprecision; Indirectness; Small Number of studies | Not upgraded |
| ARIA-E | Gantenerumab vs. Solanezumab | 0 | Some concerns | No concerns | Major concerns | No concerns | No concerns | Very Low | Reporting Bias; Indirectness; Small Number of studies; Heterogeneity | Not upgraded |
| CDR-SB | Gantenerumab vs. Solanezumab | 0 | Some concerns | No concerns | Major concerns | Major concerns | No concerns | Very Low | Reporting Bias; Imprecision; Indirectness; Small Number of studies | Not upgraded |
| Diarrhea | Gantenerumab vs. Solanezumab | 0 | Some concerns | No concerns | Major concerns | No concerns | No concerns | Very Low | Reporting Bias; Indirectness; Small Number of studies | Not upgraded |
| Nasopharynghitis | Gantenerumab vs. Solanezumab | 0 | Some concerns | No concerns | Major concerns | No concerns | No concerns | Very Low | Reporting Bias; Imprecision; Indirectness; Small Number of studies | Not upgraded |
| CSF p-Tau | Lecanemab vs. Aducanumab | 0 | Some concerns | Some concerns | Major concerns | Major concerns | No concerns | Low | Reporting Bias; Imprecision; Heterogeneity; Indirectness | Not upgraded |
| CSF Aβ 1-42 | Lecanemab vs. Aducanumab | 0 | Some concerns | No concerns | Major concerns | Major concerns | No concerns | Very Low | Reporting Bias; Imprecision; Indirectness; Small Number of studies; Heterogeneity | Not upgraded |
| ARIA-E | Lecanemab vs. Aducanumab | 0 | Some concerns | No concerns | Major concerns | No concerns | No concerns | Low | Reporting Bias; Small Number of Studies; Indirectness | Not upgraded |
| ARIA-E in APOE-ε4 Carriers | Lecanemab vs. Aducanumab | 0 | Some concerns | No concerns | Major concerns | No concerns | No concerns | Low | Reporting Bias; Small Number of Patients; Indirectness | Not upgraded |
| ARIA-H | Lecanemab vs. Aducanumab | 0 | Some concerns | No concerns | Major concerns | No concerns | No concerns | Low | Reporting Bias; Small Number of Studies; Indirectness | Not upgraded |
| Diarrhea | Lecanemab vs. Aducanumab | 0 | Some concerns | No concerns | Major concerns | No concerns | No concerns | Low | Reporting Bias; Small Number of Studies; Indirectness | Not upgraded |
| Dizziness | Lecanemab vs. Aducanumab | 0 | Some concerns | No concerns | Major concerns | No concerns | No concerns | Low | Reporting Bias; Small Number of Studies; Indirectness | Not upgraded |
| Fall | Lecanemab vs. Aducanumab | 0 | Some concerns | No concerns | Major concerns | No concerns | No concerns | Low | Reporting Bias; Small Number of Studies; Indirectness | Not upgraded |
| Headaches | Lecanemab vs. Aducanumab | 0 | Some concerns | No concerns | Major concerns | No concerns | No concerns | Low | Reporting Bias; Small Number of Studies; Indirectness | Not upgraded |
| Nasopharynghitis | Lecanemab vs. Aducanumab | 0 | Some concerns | No concerns | Major concerns | Major concerns | No concerns | Low | Reporting Bias; Imprecision; Indirectness; Small Number of Studies | Not upgraded |
| Tolerability | Lecanemab vs. Aducanumab | 0 | Some concerns | No concerns | Major concerns | No concerns | No concerns | Low | Reporting Bias; Small Number of Studies; Indirectness | Not upgraded |
| ADAS-Cog | Lecanemab vs. Aducanumab | 0 | Some concerns | No concerns | Major concerns | Major concerns | No concerns | Very Low | Reporting Bias; Imprecision; Indirectness | Not upgraded |
| ARIA-E in APOE-ε4 Non-Carriers | Lecanemab vs. Aducanumab | 0 | Some concerns | No concerns | Major concerns | No concerns | No concerns | Very Low | Reporting Bias; Small Number of Patients; Indirectness | Not upgraded |
| CDR-SB | Lecanemab vs. Aducanumab | 0 | Some concerns | No concerns | Major concerns | Major concerns | No concerns | Very Low | Reporting Bias; Imprecision; Indirectness; Small Number of Studies | Not upgraded |
| Serious Side Effects | Lecanemab vs. Aducanumab | 0 | Some concerns | No concerns | Major concerns | No concerns | No concerns | Very Low | Reporting Bias; Small Number of Studies; Indirectness; Heterogeneity | Not upgraded |
| Infusion-related reactions | Lecanemab vs. Bapineuzumab | 0 | Some concerns | No concerns | Major concerns | No concerns | No concerns | Low | Small Number of Studies, Imprecision | Not upgraded |
| Arthralgia | Lecanemab vs. Bapineuzumab | 0 | Some concerns | No concerns | Major concerns | No concerns | No concerns | Very Low | Reporting Bias; Indirectness; Small Number of Studies | Not upgraded |
| Urinary Infections | Lecanemab vs. Bapineuzumab | 0 | Some concerns | Some concerns | Major concerns | No concerns | No concerns | Very Low | Reporting Bias; Small Number of studies; Indirectness; Heterogeneity | Not upgraded |
| ARIA-E | Lecanemab vs. Bapineuzumab | 0 | Some concerns | No concerns | Major concerns | No concerns | No concerns | Low | Reporting Bias; Small Number of Studies; Indirectness | Not upgraded |
| ARIA-E in APOE-ε4 Carriers | Lecanemab vs. Bapineuzumab | 0 | Some concerns | No concerns | Major concerns | No concerns | No concerns | Low | Reporting Bias; Small Number of Patients; Indirectness | Not upgraded |
| Diarrhea | Lecanemab vs. Bapineuzumab | 0 | Some concerns | No concerns | Major concerns | No concerns | No concerns | Low | Reporting Bias; Small Number of Studies; Indirectness | Not upgraded |
| Dizziness | Lecanemab vs. Bapineuzumab | 0 | Some concerns | No concerns | Major concerns | No concerns | No concerns | Low | Reporting Bias; Small Number of Studies; Indirectness | Not upgraded |
| Fall | Lecanemab vs. Bapineuzumab | 0 | Some concerns | No concerns | Major concerns | No concerns | No concerns | Low | Reporting Bias; Small Number of Studies; Indirectness | Not upgraded |
| Tolerability | Lecanemab vs. Bapineuzumab | 0 | Some concerns | No concerns | Major concerns | No concerns | No concerns | Low | Reporting Bias; Small Number of Studies; Indirectness | Not upgraded |
| ADAS-Cog | Lecanemab vs. Bapineuzumab | 0 | Some concerns | No concerns | Major concerns | Major concerns | No concerns | Very Low | Reporting Bias; Imprecision; Indirectness | Not upgraded |
| ARIA-E in APOE-ε4 Non-Carriers | Lecanemab vs. Bapineuzumab | 0 | Some concerns | No concerns | Major concerns | No concerns | No concerns | Very Low | Reporting Bias; Small Number of Patients; Indirectness | Not upgraded |
| Back Pain | Lecanemab vs. Bapineuzumab | 0 | Some concerns | No concerns | Major concerns | No concerns | No concerns | Very Low | Reporting Bias; Indirectness Small Number of Studies | Not upgraded |
| CDR-SB | Lecanemab vs. Bapineuzumab | 0 | Some concerns | No concerns | Major concerns | Major concerns | No concerns | Very Low | Reporting Bias; Imprecision; Indirectness; Small Number of Studies | Not upgraded |
| CSF p-Tau | Lecanemab vs. Bapineuzumab | 0 | Some concerns | No concerns | Major concerns | Major concerns | No concerns | Low | Reporting Bias; Imprecision; Indirectness | Not upgraded |
| Headaches | Lecanemab vs. Bapineuzumab | 0 | Some concerns | No concerns | Major concerns | No concerns | No concerns | Very Low | Reporting Bias; Heterogeneity; Indirectness; Small Number of Studies | Not upgraded |
| Serious Side Effects | Lecanemab vs. Bapineuzumab | 0 | Some concerns | No concerns | Major concerns | No concerns | No concerns | Very Low | Reporting Bias; Small Number of Studies; Indirectness; Heterogeneity | Not upgraded |
| ARIA-E | Lecanemab vs. Donanemab | 0 | Some concerns | No concerns | Major concerns | No concerns | No concerns | Low | Reporting Bias; Small Number of Studies; Indirectness | Not upgraded |
| ARIA-E in APOE-ε4 Carriers | Lecanemab vs. Donanemab | 0 | Some concerns | No concerns | Major concerns | No concerns | No concerns | Low | Reporting Bias; Small Number of Patients; Indirectness | Not upgraded |
| ARIA-H | Lecanemab vs. Donanemab | 0 | Some concerns | No concerns | Major concerns | No concerns | No concerns | Low | Reporting Bias; Small Number of Studies; Indirectness | Not upgraded |
| Diarrhea | Lecanemab vs. Donanemab | 0 | Some concerns | No concerns | Major concerns | No concerns | No concerns | Low | Reporting Bias; Small Number of Studies; Indirectness | Not upgraded |
| Dizziness | Lecanemab vs. Donanemab | 0 | Some concerns | No concerns | Major concerns | No concerns | No concerns | Low | Reporting Bias; Small Number of Studies; Indirectness | Not upgraded |
| Fall | Lecanemab vs. Donanemab | 0 | Some concerns | No concerns | Major concerns | No concerns | No concerns | Low | Reporting Bias; Small Number of Studies; Indirectness | Not upgraded |
| Headaches | Lecanemab vs. Donanemab | 0 | Some concerns | No concerns | Major concerns | No concerns | No concerns | Low | Reporting Bias; Small Number of Studies; Indirectness | Not upgraded |
| Tolerability | Lecanemab vs. Donanemab | 0 | Some concerns | No concerns | Major concerns | No concerns | No concerns | Low | Reporting Bias; Small Number of Studies; Indirectness | Not upgraded |
| Urinary Infections | Lecanemab vs. Donanemab | 0 | Some concerns | No concerns | Major concerns | No concerns | No concerns | Low | Reporting Bias; Small Number of Studies; Indirectness | Not upgraded |
| ARIA-E in APOE-ε4 Non-Carriers | Lecanemab vs. Donanemab | 0 | Some concerns | No concerns | Major concerns | No concerns | No concerns | Very Low | Reporting Bias; Small Number of Patients; Indirectness | Not upgraded |
| Arthralgia | Lecanemab vs. Donanemab | 0 | Some concerns | No concerns | Major concerns | No concerns | No concerns | Very Low | Reporting Bias; Indirectness;Small Number of Studies | Not upgraded |
| Back Pain | Lecanemab vs. Donanemab | 0 | Some concerns | No concerns | Major concerns | No concerns | No concerns | Very Low | Reporting Bias; Indirectness Small Number of Studies | Not upgraded |
| CDR-SB | Lecanemab vs. Donanemab | 0 | Some concerns | No concerns | Major concerns | Major concerns | No concerns | Very Low | Reporting Bias; Imprecision; Indirectness; Small Number of Studies | Not upgraded |
| Serious Side Effects | Lecanemab vs. Donanemab | 0 | Some concerns | No concerns | Major concerns | No concerns | No concerns | Very Low | Reporting Bias; Small Number of Studies; Indirectness; Heterogeneity | Not upgraded |
| ADAS-Cog | Lecanemab vs. Donanemab | 0 | Some concerns | No concerns | Major concerns | Major concerns | No concerns | Very Low | Reporting Bias; Imprecision; Indirectness | Not upgraded |
| Urinary Infections | Lecanemab vs. Gantenerumab | 0 | Some concerns | No concerns | Major concerns | No concerns | No concerns | Low | Reporting Bias; Small Number of Studies; Indirectness | Not upgraded |
| CSF Aβ 1-42 | Lecanemab vs. Solanezumab | 0 | Some concerns | Major concerns | Major concerns | Major concerns | No concerns | Very Low | Reporting Bias; Imprecision; Indirectness; Small Number of studies; Heterogeneity | Not upgraded |
| ARIA-E | Lecanemab vs. Solanezumab | 0 | Some concerns | No concerns | Major concerns | No concerns | No concerns | Low | Reporting Bias; Small Number of Studies; Indirectness | Not upgraded |
| Dizziness | Lecanemab vs. Solanezumab | 0 | Some concerns | No concerns | Major concerns | No concerns | No concerns | Low | Reporting Bias; Small Number of Studies; Indirectness | Not upgraded |
| Fall | Lecanemab vs. Solanezumab | 0 | Some concerns | No concerns | Major concerns | No concerns | No concerns | Low | Reporting Bias; Small Number of Studies; Indirectness | Not upgraded |
| Headaches | Lecanemab vs. Solanezumab | 0 | Some concerns | No concerns | Major concerns | No concerns | No concerns | Low | Reporting Bias; Small Number of Studies; Indirectness | Not upgraded |
| Tolerability | Lecanemab vs. Solanezumab | 0 | Some concerns | No concerns | Major concerns | No concerns | No concerns | Low | Reporting Bias; Small Number of Studies; Indirectness | Not upgraded |
| Urinary Infections | Lecanemab vs. Solanezumab | 0 | Some concerns | No concerns | Major concerns | No concerns | No concerns | Low | Reporting Bias; Small Number of Studies; Indirectness | Not upgraded |
| ADAS-Cog | Lecanemab vs. Solanezumab | 0 | Some concerns | No concerns | Major concerns | Major concerns | No concerns | Very Low | Reporting Bias; Imprecision; Indirectness | Not upgraded |
| ARIA-H | Lecanemab vs. Solanezumab | 0 | Some concerns | No concerns | Major concerns | No concerns | No concerns | Very Low | Reporting Bias; Small Number of Studies; Indirectness | Not upgraded |
| Arthralgia | Lecanemab vs. Solanezumab | 0 | Some concerns | No concerns | Major concerns | No concerns | No concerns | Very Low | Reporting Bias; Indirectness; Small Number of Studies | Not upgraded |
| Back Pain | Lecanemab vs. Solanezumab | 0 | Some concerns | No concerns | Major concerns | No concerns | No concerns | Very Low | Reporting Bias; Indirectness Small Number of Studies | Not upgraded |
| Serious Side Effects | Lecanemab vs. Solanezumab | 0 | Some concerns | No concerns | Major concerns | No concerns | No concerns | Very Low | Reporting Bias; Indirectness; Small Number of Studies; Heterogeneity | Not upgraded |
| Diarrhea | Lecanemab vs. Solanezumab | 0 | Some concerns | No concerns | Major concerns | No concerns | No concerns | Low | Reporting Bias; Small Number of Studies; Indirectness | Not upgraded |
| CDR-SB | Lecanemab vs. Solanezumab | 0 | Some concerns | No concerns | Major concerns | Major concerns | No concerns | Very Low | Reporting Bias; Imprecision; Indirectness; Small Number of Studies | Not upgraded |
| CDR-SB | Solanezumab vs. Aducanumab | 0 | Low | No concerns | No concerns | Major concerns | No concerns | Very Low | Reporting Bias; Indirectedness | Not upgraded |
| Arthralgia | Solanezumab vs. Bapineuzumab | 0 | Some concerns | No concerns | Major concerns | No concerns | No concerns | Low | Reporting Bias; Indirectness; Indirectness | Not upgraded |
| Upper respiratory Infections | Solanezumab vs. Bapineuzumab | 0 | Some concerns | No concerns | Major concerns | No concerns | No concerns | Low | Reporting Bias; Imprecision; Heterogeneity; Indirectness | Not upgraded |
| Back Pain | Solanezumab vs. Bapineuzumab | 0 | Some concerns | No concerns | Major concerns | No concerns | No concerns | Low | Reporting Bias; Indirectness; Indirectness | Not upgraded |
| Dizziness | Solanezumab vs. Bapineuzumab | 0 | Some concerns | No concerns | Major concerns | No concerns | No concerns | Low | Reporting Bias; Imprecision; Indirectness | Not upgraded |
| Fall | Solanezumab vs. Bapineuzumab | 0 | Some concerns | No concerns | Major concerns | No concerns | No concerns | Low | Reporting Bias; Imprecision; Indirectness | Not upgraded |
| Nasopharynghitis | Solanezumab vs. Bapineuzumab | 0 | Some concerns | No concerns | Major concerns | No concerns | No concerns | Low | Reporting Bias; Imprecision; Indirectness | Not upgraded |
| Nausea | Solanezumab vs. Bapineuzumab | 0 | Some concerns | No concerns | Major concerns | No concerns | No concerns | Low | Reporting Bias; Imprecision; Indirectness | Not upgraded |
| Tolerability | Solanezumab vs. Bapineuzumab | 0 | Some concerns | No concerns | Major concerns | No concerns | No concerns | Low | Reporting Bias; Imprecision; Indirectness | Not upgraded |
| Urinary Infections | Solanezumab vs. Bapineuzumab | 0 | Some concerns | No concerns | Major concerns | No concerns | No concerns | Low | Reporting Bias; Imprecision; Indirectness | Not upgraded |
| ADAS-Cog | Solanezumab vs. Bapineuzumab | 0 | Some concerns | No concerns | Major concerns | Major concerns | No concerns | Very Low | Reporting Bias; Imprecision; Indirectness | Not upgraded |
| Serious Side Effects | Solanezumab vs. Bapineuzumab | 0 | Some concerns | No concerns | Major concerns | No concerns | No concerns | Very Low | Reporting Bias; Indirectness; Small Number of Studies; Heterogeneity | Not upgraded |
| ARIA-E | Solanezumab vs. Bapineuzumab | 0 | Some concerns | No concerns | Major concerns | No concerns | No concerns | Low | Reporting Bias; Heterogeneity; Indirectness | Not upgraded |
| Diarrhea | Solanezumab vs. Bapineuzumab | 0 | Some concerns | No concerns | Major concerns | No concerns | No concerns | Low | Reporting Bias; Imprecision; Indirectness | Not upgraded |
| Headaches | Solanezumab vs. Bapineuzumab | 0 | Some concerns | No concerns | Major concerns | No concerns | No concerns | Low | Reporting Bias; Heterogeneity; Indirectness | Not upgraded |
| CDR-SB | Solanezumab vs. Bapineuzumab | 0 | Some concerns | No concerns | Major concerns | Major concerns | No concerns | Very Low | Reporting Bias; Imprecision; Indirectness | Not upgraded |
| MMSE | Solanezumab vs. Bapineuzumab | 0 | Some concerns | No concerns | Major concerns | Major concerns | No concerns | Very Low | Reporting Bias; Imprecision; Indirectness | Not upgraded |
| CDR-SB | Solanezumab vs. Donanemab | 0 | Some concerns | No concerns | Major concerns | Major concerns | No concerns | Very Low | Reporting Bias; Imprecision; Indirectness | Not upgraded |
| CDR-SB | Solanezumab vs. Lecanemab | 0 | Some concerns | No concerns | Major concerns | Major concerns | No concerns | Very Low | Reporting Bias; Imprecision; Indirectness | Not upgraded |
