# Supplementary material for: Clinical Benefits and Risks of Antiamyloid Antibodies in Sporadic Alzheimer Disease: Systematic Review and Network Meta-Analysis With a Web Application
Source: J Med Internet Res. 2025 Apr 7;27:e68454. doi: 10.2196/68454 (PMC12012406; doi:10.2196/68454)
Supplement: Multimedia Appendix 10 [file jmir_v27i1e68454_app10.docx]

### **Multimedia Appendix 10.** Surface under the cumulative ranking curve rank robustness.

| **Outcome** | **Interventions** | **Number (%) of studies displacing treatment’s rank by 1 rank** | **Number (%) of studies displacing treatment’s rank by 2 ranks** | **Number (%) of studies displacing treatment’s rank by 3 ranks** | **Number (%) of trials that did not change any treatment rank (%)** | **Rank robustness: Weighted Cohen's kappa: Median (Min; Max)** |
| --- | --- | --- | --- | --- | --- | --- |
| ADAS-Cog | Donanemab | 0 (0%) | 0 (0%) | 0 (0%) | 12 out of 18 (66.67%) | 97% (80%; 100%) |
|  | Gantenerumab | 2 (8.70%) | 0 (0%) | 0 (0%) |  |  |
|  | Lecanemab | 2 (8.70%) | 1 (4.35%) | 0 (0%) |  |  |
|  | Aducanumab | 1 (4.35%) | 1 (4.35%) | 0 (0%) |  |  |
|  | Solanezumab | 0 (0%) | 0 (0%) | 0 (0%) |  |  |
|  | Bapineuzumab | 2 (8.70%) | 0 (0%) | 0 (0%) |  |  |
|  | Crenezumab | 3 (13.04%) | 0 (0%) | 0 (0%) |  |  |
|  | Placebo | 0 (0%) | 0 (0%) | 0 (0%) |  |  |
| MMSE | Donanemab | 0 (0%) | 0 (0%) | 0 (0%) | 7 out of 13 (53.85%) | 94% (68%; 100%) |
|  | Solanezumab | 0 (0%) | 0 (0%) | 0 (0%) |  |  |
|  | Aducanumab | 1 (7.69%) | 0 (0%) | 0 (0%) |  |  |
|  | Bapineuzumab | 6 (45.15%) | 1 (7.69%) | 0 (0%) |  |  |
|  | Gantenerumab | 6 (45.15%) | 0 (0%) | 0 (0%) |  |  |
|  | Placebo | 1 (7.69%) | 0 (0%) | 0 (0%) |  |  |
|  | Crenezumab | 1 (7.69%) | 0 (0%) | 0 (0%) |  |  |
| CDR-SB | Donanemab | 0 (0%) | 0 (0%) | 0 (0%) | 12 out of 19 (63.16%) | 97.5% (85%; 99%) |
|  | Lecanemab | 1 (5.88%) | 1 (5.88%) | 0 (0%) |  |  |
|  | Aducanumab | 2 (11.76%) | 1 (5.88%) | 0 (0%) |  |  |
|  | Gantenerumab | 3 (17.64%) | 0 (0%) | 0 (0%) |  |  |
|  | Solanezumab | 2 (11.76%) | 0 (0%) | 0 (0%) |  |  |
|  | Placebo | 2 (11.76%) | 0 (0%) | 0 (0%) |  |  |
|  | Crenezumab | 5 (29.41%) | 0 (0%) | 0 (0%) |  |  |
|  | Bapineuzumab | 4 (23.53%) | 2 (11.76%) | 0 (0%) |  |  |
| ARIA-E | Donanemab | 3 (18.75%) | 3 (18.75%) | 0 (0%) | 8 out of 16 (50%) | 94% (-8.9%: 100%) |
|  | Gantenerumab | 1 (6.25%) | 0 (0%) | 0 (0%) |  |  |
|  | Lecanemab | 8 (50.0%) | 0 (0%) | 0 (0%) |  |  |
|  | Aducanumab | 6 (37.5%) | 1 (6.25%) | 0 (0%) |  |  |
|  | Solanezumab | 2 (12.5%) | 0 (0%) | 0 (0%) |  |  |
|  | Bapineuzumab | 0 (0%) | 0 (0%) | 0 (0%) |  |  |
|  | Crenezumab | 2 (12.5%) | 0 (0%) | 0 (0%) |  |  |
|  | Placebo | 0 (0%) | 0 (0%) | 0 (0%) |  |  |
| ARIA-H | Donanemab | 0 (0%) | 0 (0%) | 0 (0%) | 9 out of 11 (81.82%) | 97% (95%: 99%) |
|  | Gantenerumab | 0 (0%) | 0 (0%) | 0 (0%) |  |  |
|  | Lecanemab | 0 (0%) | 0 (0%) | 0 (0%) |  |  |
|  | Aducanumab | 0 (0%) | 0 (0%) | 0 (0%) |  |  |
|  | Solanezumab | 1 (9.09%) | 0 (0%) | 0 (0%) |  |  |
|  | Bapineuzumab | 0 (0%) | 0 (0%) | 0 (0%) |  |  |
|  | Crenezumab | 1 (9.09%) | 0 (0%) | 0 (0%) |  |  |
|  | Placebo | 0 (0%) | 0 (0%) | 0 (0%) |  |  |
| Tolerability: Treatment discontinuations due to adverse events | Donanemab | 0 (0%) | 0 (0%) | 0 (0%) | 17 out of 19 (89.47%) | 98% (92%: 99%) |
|  | Lecanemab | 0 (0%) | 0 (0%) | 0 (0%) |  |  |
|  | Aducanumab | 0 (0%) | 0 (0%) | 0 (0%) |  |  |
|  | Gantenerumab | 0 (0%) | 0 (0%) | 0 (0%) |  |  |
|  | Solanezumab | 1 (5.26%) | 0 (0%) | 0 (0%) |  |  |
|  | Placebo | 2 (10.53%) | 0 (0%) | 0 (0%) |  |  |
|  | Crenezumab | 1 (5.26%) | 0 (0%) | 0 (0%) |  |  |
|  | Bapineuzumab | 0 (0%) | 0 (0%) | 0 (0%) |  |  |
| Headaches | Donanemab | 2 (11.76%) | 0 (0%) | 0 (0%) | 11 out of 17 (64.71%) | 96% (-46%: 100%) |
|  | Lecanemab | 3 (17.65%) | 0 (0%) | 0 (0%) |  |  |
|  | Aducanumab | 2 (11.76%) | 0 (0%) | 0 (0%) |  |  |
|  | Gantenerumab | 2 (11.76%) | 1 (5.88%) | 0 (0%) |  |  |
|  | Solanezumab | 4 (23.53%) | 0 (0%) | 0 (0%) |  |  |
|  | Placebo | 4 (23.53%) | 1 (5.88%) | 0 (0%) |  |  |
|  | Crenezumab | 2 (11.76%) | 0 (0%) | 0 (0%) |  |  |
|  | Bapineuzumab | 1 (5.88%) | 0 (0%) | 1 (5.88%) |  |  |
| Infusion-related reactions | Donanemab | 1 (16.67%) | 0 (0%) | 0 (0%) | 4 out of 6 (66.67%) | -2.9% (-100%: 100%) |
|  | Lecanemab | 1 (16.67%) | 0 (0%) | 0 (0%) |  |  |
|  | Placebo | 0 (0%) | 0 (0%) | 0 (0%) |  |  |
|  | Crenezumab | 1 (16.67%) | 0 (0%) | 0 (0%) |  |  |
|  | Bapineuzumab | 2 (33.33%) | 0 (0%) | 0 (0%) |  |  |

| **Study Name (Year) Dose** | **Clinical Trial ID** | **Phase** | **Drug** |
| --- | --- | --- | --- |
| **Salloway et al [1] (2009) high dose** | **NCT00112073** | **II** | **Bapineuzumab** |
| **Salloway et al [2] 1 (2014) Study 301 low dose** | **NCT00574132** | **III** | **Bapineuzumab** |
| **Salloway et al [2] 2 (2014) Study 301 high dose** | **NCT00574132** | **III** | **Bapineuzumab** |
| **Salloway et al [2] 3 (2014) Study 302 low dose** | **NCT00575055** | **III** | **Bapineuzumab** |
| **Doody et al [3] 1 (2014) EXPEDITION 1** | **NCT00905372** | **III** | **Solanezumab** |
| **Doody et al [3] 2 (2014) EXPEDITION 2** | **NCT00904683** | **III** | **Solanezumab** |
| **Vandenberghe et al [4] 1 (2016) low dose** | **NCT00667810** | **III** | **Bapineuzumab** |
| **Vandenberghe et al [4] 2 (2016) high dose** | **NCT00667810** | **III** | **Bapineuzumab** |
| **Vandenberghe et al [4] 3 (2016) low dose** | **NCT00676143** | **III** | **Bapineuzumab** |
| **Honig et al [5] (2018) EXPEDITION 3** | **NCT01900665** | **III** | **Solanezumab** |
| **Haeberlein et al [6] (2022) EMERGE low dose** | **NCT02484547** | **III** | **Aducanumab** |
| **Haeberlein et al [6] (2022) EMERGE high dose** | **NCT02484547** | **III** | **Aducanumab** |
| **Haeberlein et al [6] (2022) ENGAGE low dose** | **NCT02477800** | **III** | **Aducanumab** |
| **Haeberlein et al [6] (2022) ENGAGE high dose** | **NCT02477800** | **III** | **Aducanumab** |
| **van Dyck et al [7] (2023) Clarity AD** | **NCT03887455** | **III** | **Lecanemab** |
| **Swanson et al [8] 1 (2021) high dose** | **NCT01767311** | **II** | **Lecanemab** |
| **Swanson et al [8] 2 (2021) low dose** | **NCT01767311** | **II** | **Lecanemab** |
| **Sims et al [9] (2023) TRAILBLAZER-ALZ 2 (pooled)** | **NCT04437511** | **III** | **Donanemab** |
| **Mintun et al [10] (2021) TRAILBLAZER-ALZ** | **NCT03367403** | **II** | **Donanemab** |
| **Bateman et al [11] (2023) GRADUATE I** | **NCT03444870** | **III** | **Gantenerumab** |
| **Bateman et al [11] (2023) GRADUATE II** | **NCT03443973** | **III** | **Gantenerumab** |
| **Ostrowitzki et al [12] (2022) CREAD** | **NCT02670083** | **III** | **Crenezumab** |
| **Salloway et al [13] (2018) BLAZE (pooled)** | **NCT01397578** | **II** | **Crenezumab** |
| **Ostrowitzki et al [14] (2017) SCarlet RoAD I** | **NCT01224106** | **III** | **Gantenerumab** |
| **Ostrowitzki et al [14] (2017) SCarlet RoAD II** | **NCT01224106** | **III** | **Gantenerumab** |

**References**

1. Salloway S, Sperling R, Gilman S, Fox NC, Blennow K, Raskind M, et al. A phase 2 multiple ascending dose trial of bapineuzumab in mild to moderate Alzheimer disease. Neurology. 2009 Dec 15;73(24):2061-70. PMID: 19923550. doi: 10.1212/WNL.0b013e3181c67808.

2. Salloway S, Sperling R, Fox NC, Blennow K, Klunk W, Raskind M, et al. Two phase 3 trials of bapineuzumab in mild-to-moderate Alzheimer's disease. N Engl J Med. 2014 Jan 23;370(4):322-33. PMID: 24450891. doi: 10.1056/NEJMoa1304839.

3. Doody RS, Thomas RG, Farlow M, Iwatsubo T, Vellas B, Joffe S, et al. Phase 3 trials of solanezumab for mild-to-moderate Alzheimer's disease. N Engl J Med. 2014 Jan 23;370(4):311-21. PMID: 24450890. doi: 10.1056/NEJMoa1312889.

4. Vandenberghe R, Rinne JO, Boada M, Katayama S, Scheltens P, Vellas B, et al. Bapineuzumab for mild to moderate Alzheimer's disease in two global, randomized, phase 3 trials. Alzheimers Res Ther. 2016 May 12;8(1):18. PMID: 27176461. doi: 10.1186/s13195-016-0189-7.

5. Honig LS, Vellas B, Woodward M, Boada M, Bullock R, Borrie M, et al. Trial of Solanezumab for Mild Dementia Due to Alzheimer's Disease. N Engl J Med. 2018 Jan 25;378(4):321-30. PMID: 29365294. doi: 10.1056/NEJMoa1705971.

6. Budd Haeberlein S, Aisen PS, Barkhof F, Chalkias S, Chen T, Cohen S, et al. Two Randomized Phase 3 Studies of Aducanumab in Early Alzheimer's Disease. J Prev Alzheimers Dis. 2022;9(2):197-210. PMID: 35542991. doi: 10.14283/jpad.2022.30.

7. van Dyck CH, Swanson CJ, Aisen P, Bateman RJ, Chen C, Gee M, et al. Lecanemab in Early Alzheimer's Disease. N Engl J Med. 2023 Jan 5;388(1):9-21. PMID: 36449413. doi: 10.1056/NEJMoa2212948.

8. Swanson CJ, Zhang Y, Dhadda S, Wang J, Kaplow J, Lai RYK, et al. A randomized, double-blind, phase 2b proof-of-concept clinical trial in early Alzheimer's disease with lecanemab, an anti-Aβ protofibril antibody. Alzheimers Res Ther. 2021 Apr 17;13(1):80. PMID: 33865446. doi: 10.1186/s13195-021-00813-

9. Sims JR, Zimmer JA, Evans CD, Lu M, Ardayfio P, Sparks J, et al. Donanemab in Early Symptomatic Alzheimer Disease: The TRAILBLAZER-ALZ 2 Randomized Clinical Trial. Jama. 2023 Aug 8;330(6):512-27. PMID: 37459141. doi: 10.1001/jama.2023.13239.

10. Mintun MA, Lo AC, Duggan Evans C, Wessels AM, Ardayfio PA, Andersen SW, et al. Donanemab in Early Alzheimer's Disease. N Engl J Med. 2021 May 6;384(18):1691-704. PMID: 33720637. doi: 10.1056/NEJMoa2100708.

11. Bateman RJ, Smith J, Donohue MC, Delmar P, Abbas R, Salloway S, et al. Two Phase 3 Trials of Gantenerumab in Early Alzheimer's Disease. N Engl J Med. 2023 Nov 16;389(20):1862-76. PMID: 37966285. doi: 10.1056/NEJMoa2304430.

12. Ostrowitzki S, Bittner T, Sink KM, Mackey H, Rabe C, Honig LS, et al. Evaluating the Safety and Efficacy of Crenezumab vs Placebo in Adults With Early Alzheimer Disease: Two Phase 3 Randomized Placebo-Controlled Trials. JAMA Neurol. 2022 Nov 1;79(11):1113-21. PMID: 36121669. doi: 10.1001/jamaneurol.2022.2909.

13. Salloway S, Honigberg LA, Cho W, Ward M, Friesenhahn M, Brunstein F, et al. Amyloid positron emission tomography and cerebrospinal fluid results from a crenezumab anti-amyloid-beta antibody double-blind, placebo-controlled, randomized phase II study in mild-to-moderate Alzheimer's disease (BLAZE). Alzheimers Res Ther. 2018 Sep 19;10(1):96. PMID: 30231896. doi: 10.1186/s13195-018-0424-5.

14. Ostrowitzki S, Lasser RA, Dorflinger E, Scheltens P, Barkhof F, Nikolcheva T, et al. A phase III randomized trial of gantenerumab in prodromal Alzheimer's disease. Alzheimers Res Ther. 2017 Dec 8;9(1):95. PMID: 29221491. doi: 10.1186/s13195-017-0318-y.
