# Supplementary material for: Clinical Benefits and Risks of Antiamyloid Antibodies in Sporadic Alzheimer Disease: Systematic Review and Network Meta-Analysis With a Web Application
Source: J Med Internet Res. 2025 Apr 7;27:e68454. doi: 10.2196/68454 (PMC12012406; doi:10.2196/68454)
Supplement: Multimedia Appendix 11 [file jmir_v27i1e68454_app11.docx]

### **Multimedia Appendix 11.** Reported impact of the COVID-19 pandemic on the primary studies.

| **Study** | **Number (%) of patients affected (Drug vs. Placebo)** | **Reported notes on the impact of COVID** |
| --- | --- | --- |
| van Dyck et al [1] (2023) Clarity AD | 64 (7.1) vs. 60 (6.7) | The COVID-19 pandemic caused missing doses during the trial, delayed assessments, and intercurrent illnesses. The dropout rate was 17.2%, and a sensitivity analysis that evaluated the effect of missed doses was consistent with the primary end-point analysis. The sample size was increased by 200 to account for participants who missed three or more consecutive doses during the initial 6-month peak period of coronavirus disease 2019 (Covid-19), in accordance with previous agreement with regulatory authorities. Sensitivity analyses of the CDR-SB score that evaluated the effect of Covid-19 (missed doses) and potential for bias from functional unblinding due to ARIA were generally consistent with the primary analysis |
| Sims et al [2] (2023) TRAILBLAZER-ALZ 2 | Low/medium-tau:  94 (16.1) vs. 106 (17.9)  High-tau population:  42 (15.7) vs. 48 (17.1) | The TRAILBLAZER-ALZ 2 trial was conducted during the COVID-19 pandemic, and COVID-19 was the most commonly reported adverse event across treatment groups |
| Mintun et al [3] (2021) TRAILBLAZER-ALZ | 3 (2.29) vs. 2 (1.6) | Due to the COVID-19 pandemic, investigative sites were allowed to replace on-site visits with telephone visits for any visit except the final visit at 76 weeks; efficacy data were not collected, and the trial drug was not dispensed at telephone visits. Because missed assessments were not allowed to occur at the final visit, the effect on interpretation of the analyses was considered to be minimal. |
| Bateman et al [4] (2023) GRADUATE I and II | Not reported | The double-blind period was extended from 104 to 116 weeks in response to delayed and missed visits due to the pandemic, due to the estimation that participants would miss a mean of 8 weeks (i.e., 2-4 visits during the double-blind period), which would reduce the power from 90% to 80%. The protocol was therefore amended to extend the double-blind period to a total of 116 weeks to mitigate the effect of missed visits. |

**References**

1. van Dyck CH, Swanson CJ, Aisen P, Bateman RJ, Chen C, Gee M, et al. Lecanemab in Early Alzheimer's Disease. N Engl J Med. 2023 Jan 5;388(1):9-21. PMID: 36449413. doi: 10.1056/NEJMoa2212948.

2. Sims JR, Zimmer JA, Evans CD, Lu M, Ardayfio P, Sparks J, et al. Donanemab in Early Symptomatic Alzheimer Disease: The TRAILBLAZER-ALZ 2 Randomized Clinical Trial. Jama. 2023 Aug 8;330(6):512-27. PMID: 37459141. doi: 10.1001/jama.2023.13239.

3. Mintun MA, Lo AC, Duggan Evans C, Wessels AM, Ardayfio PA, Andersen SW, et al. Donanemab in Early Alzheimer's Disease. N Engl J Med. 2021 May 6;384(18):1691-704. PMID: 33720637. doi: 10.1056/NEJMoa2100708.

4. Bateman RJ, Smith J, Donohue MC, Delmar P, Abbas R, Salloway S, et al. Two Phase 3 Trials of Gantenerumab in Early Alzheimer's Disease. N Engl J Med. 2023 Nov 16;389(20):1862-76. PMID: 37966285. doi: 10.1056/NEJMoa2304430.
