# Supplementary material for: Clinical Benefits and Risks of Antiamyloid Antibodies in Sporadic Alzheimer Disease: Systematic Review and Network Meta-Analysis With a Web Application
Source: J Med Internet Res. 2025 Apr 7;27:e68454. doi: 10.2196/68454 (PMC12012406; doi:10.2196/68454)
Supplement: Multimedia Appendix 12 [file jmir_v27i1e68454_app12.docx]

### **Multimedia Appendix 12.** Influential studies and their impacts on heterogeneity.

| **Method of detection:**  **Standard methods:** Leave-one-out, Baujat and Influence Diagnostics; **Machine Learning (ML) methods:** k-means clustering; DBSCAN and Gaussian mixture model (GMM) | **Influential Observation: Outcome** | **Intervention** | **Influential study (Arm)** | **Impact of exclusion on heterogeneity reduction** |
| --- | --- | --- | --- | --- |
| Both standard and ML methods | Amyloid burden on PET | Donanemab | Mintun et al., 2021 | Significant  (p < 0.05) |
| Both standard and ML methods | CSF Amyloid | Lecanemab | Swanson et al., 2021 | Significant  (p < 0.05) |
| Both standard and ML methods | CSF p-tau | Aducanumab | Budd Haeberlein et al., 2022 high dose | Significant  (p < 0.05) |
| Both standard and ML methods | Headaches | Bapineuzumab | Vandenberghe et al., 2016 | Significant  (p < 0.05) |
| Both standard and ML methods | Serious adverse events | Lecanemab | van Dyck et al 2023 Clarity AD | Significant  (p < 0.05) |
| Both standard and ML methods | Serious adverse events | Bapineuzumab | Salloway et al., 2014 | Significant  (p < 0.05) |
| Both standard and ML methods | Total ARIA-H | Gantenerumab | Bateman et al., 2023 GRADUATE II | Significant  (p < 0.05) |
| Both standard and ML methods | Urinary Infections | Bapineuzumab | Vandenberghe et al., 2016 | Significant  (p < 0.05) |
| Both standard and ML methods | ADAS-Cog | Bapineuzumab | Salloway et al., 2014 High dose | Not significant |
| Both standard and ML methods | ADAS-Cog | Lecanemab | Swanson et al., 2021 High dose | Not significant |
| ML methods | ADAS-Cog, CDR-SB | Aducanumab | Budd Haeberlein et al., 2022 High dose | Not significant |
| Both standard and ML methods | ADAS-Cog, CDR-SB | Donanemab | Sims et al., 2023 Low-medium tau | Not significant |
| Both standard and ML methods | Amyloid burden on PET | Lecanemab | Swanson et al., 2021 Low dose | Not significant |
| Standard methods | ARIA-E in APOE-ε4 carriers | Lecanemab | van Dyck et al., 2023 | Not significant |
| ML methods | ARIA-E in APOE-ε4 carriers and non-carriers | Donanemab | Mintun et al., 2021; Sims et al., 2023 | Not significant |
| ML methods | Arthralgia | Bapineuzumab | Salloway et al., 2014 Low and high dose study | Not significant |
| ML methods | CDR-SB | Lecanemab | Swanson et al., 2021 High dose | Not significant |
| Both standard and ML methods | CSF Amyloid | Solanezumab | Doody et al., 2014 | Not significant |
| Both standard and ML methods | Diarrhea | Bapineuzumab | Salloway et al., 2014 High Dose | Not significant |
| ML methods | Dizziness, Diarrhea, Arthralgia, Urinary infections | Bapineuzumab | Vandenberghe et al., 2016 | Not significant |
| ML methods | Fall | Aducanumab | Budd Haeberlein et al., 2022 ENGAGE low and high dose | Not significant |
| Standard methods | Fall | Aducanumab | Budd Haeberlein et al., 2022 ENGAGE low dose | Not significant |
| ML methods | Fatigue, Total ARIA-H, Upper respiratory infections | Solanezumab | Doody et al., 2014 | Not significant |
| Both standard and ML methods | Infusion-related reactions | Crenezumab | Ostrowitzki et al., 2022 | Not significant |
| ML methods | MMSE | Solanezumab | Honig et al., 2018 | Not significant |
| Both standard and ML methods | MMSE, CDR-SB | Bapineuzumab | Salloway et al., 2014 Low dose | Not significant |
| ML methods | Nausea | Donanemab | Mintun et al., 2021; Sims et al., 2023 High Tau | Not significant |
| Standard methods | Nausea | Donanemab | Sims et al., 2023 High-tau | Not significant |
| Both standard and ML methods | Nausea, Total ARIA-E and ARIA-E in APOE-ε4 carriers | Bapineuzumab | Salloway et al., 2014 Low Dose | Not significant |
| Both standard and ML methods | Nausea, Total ARIA-E, Total ARIA-H, Upper respiratory infections | Solanezumab | Doody et al., 2014 | Not significant |
| ML methods | Serious adverse events | Aducanumab | Budd Haeberlein et al., 2022 EMERGE low dose | Not significant |
| ML methods | Serious adverse events | Lecanemab | Swanson et al., 2021 | Not significant |
| Both standard and ML methods | Serious adverse events, Total ARIA-E, Fatigue | Solanezumab | Honig et al., 2018 | Not significant |
| ML methods | Dizziness, ARIA-E in APOE-e4 carriers | Lecanemab | van Dyck et al., 2023 | Not significant |
| Both standard and ML methods | Total ARIA-E | Aducanumab | Budd Haeberlein et al., 2022 ENGAGE low dose | Not significant |
| ML methods | Treatment discontinuation due to Adverse Events | Solanezumab | Honig et al., 2018 | Not significant |
| ML methods | Treatment Discontinuation due to Adverse Events, Total ARIA-H | Donanemab | Sims et al., 2023 Low-medium tau | Not significant |
| Standard methods | Treatment Discontinuation due to Adverse Events, Total ARIA-H, ARIA-E in APOE-ε4 carriers and non-carriers | Donanemab | Sims et al., 2023 Low-medium tau | Not significant |

| **Study Name (Year) Dose** | **Clinical Trial ID** | **Phase** | **Drug** |
| --- | --- | --- | --- |
| **Salloway et al [1] (2009) high dose** | **NCT00112073** | **II** | **Bapineuzumab** |
| **Salloway et al [2] 1 (2014) Study 301 low dose** | **NCT00574132** | **III** | **Bapineuzumab** |
| **Salloway et al [2] 2 (2014) Study 301 high dose** | **NCT00574132** | **III** | **Bapineuzumab** |
| **Salloway et al [2] 3 (2014) Study 302 low dose** | **NCT00575055** | **III** | **Bapineuzumab** |
| **Doody et al [3] 1 (2014) EXPEDITION 1** | **NCT00905372** | **III** | **Solanezumab** |
| **Doody et al [3] 2 (2014) EXPEDITION 2** | **NCT00904683** | **III** | **Solanezumab** |
| **Vandenberghe et al [4] 1 (2016) low dose** | **NCT00667810** | **III** | **Bapineuzumab** |
| **Vandenberghe et al [4] 2 (2016) high dose** | **NCT00667810** | **III** | **Bapineuzumab** |
| **Vandenberghe et al [4] 3 (2016) low dose** | **NCT00676143** | **III** | **Bapineuzumab** |
| **Honig et al [5] (2018) EXPEDITION 3** | **NCT01900665** | **III** | **Solanezumab** |
| **Haeberlein et al [6] (2022) EMERGE low dose** | **NCT02484547** | **III** | **Aducanumab** |
| **Haeberlein et al [6] (2022) EMERGE high dose** | **NCT02484547** | **III** | **Aducanumab** |
| **Haeberlein et al [6] (2022) ENGAGE low dose** | **NCT02477800** | **III** | **Aducanumab** |
| **Haeberlein et al [6] (2022) ENGAGE high dose** | **NCT02477800** | **III** | **Aducanumab** |
| **van Dyck et al [7] (2023) Clarity AD** | **NCT03887455** | **III** | **Lecanemab** |
| **Swanson et al [8] 1 (2021) high dose** | **NCT01767311** | **II** | **Lecanemab** |
| **Swanson et al [8] 2 (2021) low dose** | **NCT01767311** | **II** | **Lecanemab** |
| **Sims et al [9] (2023) TRAILBLAZER-ALZ 2 (pooled)** | **NCT04437511** | **III** | **Donanemab** |
| **Mintun et al [10] (2021) TRAILBLAZER-ALZ** | **NCT03367403** | **II** | **Donanemab** |
| **Bateman et al [11] (2023) GRADUATE I** | **NCT03444870** | **III** | **Gantenerumab** |
| **Bateman et al [11] (2023) GRADUATE II** | **NCT03443973** | **III** | **Gantenerumab** |
| **Ostrowitzki et al [12] (2022) CREAD** | **NCT02670083** | **III** | **Crenezumab** |
| **Salloway et al [13] (2018) BLAZE (pooled)** | **NCT01397578** | **II** | **Crenezumab** |
| **Ostrowitzki et al [14] (2017) SCarlet RoAD I** | **NCT01224106** | **III** | **Gantenerumab** |
| **Ostrowitzki et al [14] (2017) SCarlet RoAD II** | **NCT01224106** | **III** | **Gantenerumab** |

**References**

1. Salloway S, Sperling R, Gilman S, Fox NC, Blennow K, Raskind M, et al. A phase 2 multiple ascending dose trial of bapineuzumab in mild to moderate Alzheimer disease. Neurology. 2009 Dec 15;73(24):2061-70. PMID: 19923550. doi: 10.1212/WNL.0b013e3181c67808.

2. Salloway S, Sperling R, Fox NC, Blennow K, Klunk W, Raskind M, et al. Two phase 3 trials of bapineuzumab in mild-to-moderate Alzheimer's disease. N Engl J Med. 2014 Jan 23;370(4):322-33. PMID: 24450891. doi: 10.1056/NEJMoa1304839.

3. Doody RS, Thomas RG, Farlow M, Iwatsubo T, Vellas B, Joffe S, et al. Phase 3 trials of solanezumab for mild-to-moderate Alzheimer's disease. N Engl J Med. 2014 Jan 23;370(4):311-21. PMID: 24450890. doi: 10.1056/NEJMoa1312889.

4. Vandenberghe R, Rinne JO, Boada M, Katayama S, Scheltens P, Vellas B, et al. Bapineuzumab for mild to moderate Alzheimer's disease in two global, randomized, phase 3 trials. Alzheimers Res Ther. 2016 May 12;8(1):18. PMID: 27176461. doi: 10.1186/s13195-016-0189-7.

5. Honig LS, Vellas B, Woodward M, Boada M, Bullock R, Borrie M, et al. Trial of Solanezumab for Mild Dementia Due to Alzheimer's Disease. N Engl J Med. 2018 Jan 25;378(4):321-30. PMID: 29365294. doi: 10.1056/NEJMoa1705971.

6. Budd Haeberlein S, Aisen PS, Barkhof F, Chalkias S, Chen T, Cohen S, et al. Two Randomized Phase 3 Studies of Aducanumab in Early Alzheimer's Disease. J Prev Alzheimers Dis. 2022;9(2):197-210. PMID: 35542991. doi: 10.14283/jpad.2022.30.

7. van Dyck CH, Swanson CJ, Aisen P, Bateman RJ, Chen C, Gee M, et al. Lecanemab in Early Alzheimer's Disease. N Engl J Med. 2023 Jan 5;388(1):9-21. PMID: 36449413. doi: 10.1056/NEJMoa2212948.

8. Swanson CJ, Zhang Y, Dhadda S, Wang J, Kaplow J, Lai RYK, et al. A randomized, double-blind, phase 2b proof-of-concept clinical trial in early Alzheimer's disease with lecanemab, an anti-Aβ protofibril antibody. Alzheimers Res Ther. 2021 Apr 17;13(1):80. PMID: 33865446. doi: 10.1186/s13195-021-00813-

9. Sims JR, Zimmer JA, Evans CD, Lu M, Ardayfio P, Sparks J, et al. Donanemab in Early Symptomatic Alzheimer Disease: The TRAILBLAZER-ALZ 2 Randomized Clinical Trial. Jama. 2023 Aug 8;330(6):512-27. PMID: 37459141. doi: 10.1001/jama.2023.13239.

10. Mintun MA, Lo AC, Duggan Evans C, Wessels AM, Ardayfio PA, Andersen SW, et al. Donanemab in Early Alzheimer's Disease. N Engl J Med. 2021 May 6;384(18):1691-704. PMID: 33720637. doi: 10.1056/NEJMoa2100708.

11. Bateman RJ, Smith J, Donohue MC, Delmar P, Abbas R, Salloway S, et al. Two Phase 3 Trials of Gantenerumab in Early Alzheimer's Disease. N Engl J Med. 2023 Nov 16;389(20):1862-76. PMID: 37966285. doi: 10.1056/NEJMoa2304430.

12. Ostrowitzki S, Bittner T, Sink KM, Mackey H, Rabe C, Honig LS, et al. Evaluating the Safety and Efficacy of Crenezumab vs Placebo in Adults With Early Alzheimer Disease: Two Phase 3 Randomized Placebo-Controlled Trials. JAMA Neurol. 2022 Nov 1;79(11):1113-21. PMID: 36121669. doi: 10.1001/jamaneurol.2022.2909.

13. Salloway S, Honigberg LA, Cho W, Ward M, Friesenhahn M, Brunstein F, et al. Amyloid positron emission tomography and cerebrospinal fluid results from a crenezumab anti-amyloid-beta antibody double-blind, placebo-controlled, randomized phase II study in mild-to-moderate Alzheimer's disease (BLAZE). Alzheimers Res Ther. 2018 Sep 19;10(1):96. PMID: 30231896. doi: 10.1186/s13195-018-0424-5.

14. Ostrowitzki S, Lasser RA, Dorflinger E, Scheltens P, Barkhof F, Nikolcheva T, et al. A phase III randomized trial of gantenerumab in prodromal Alzheimer's disease. Alzheimers Res Ther. 2017 Dec 8;9(1):95. PMID: 29221491. doi: 10.1186/s13195-017-0318-y.
